# Hydroxytyrosol-Infused Extra Virgin Olive Oil: A Key to Minimizing Oxidation, Boosting Antioxidant Potential, and Enhancing Physicochemical Stability During Frying

**DOI:** 10.3390/antiox14030368

**Published:** 2025-03-20

**Authors:** Taha Mehany, José M. González-Sáiz, Consuelo Pizarro

**Affiliations:** Department of Chemistry, University of La Rioja, 26006 Logroño, Spain; taha.abdellatif@unirioja.es (T.M.); josemaria.gonzalez@unirioja.es (J.M.G.-S.)

**Keywords:** antioxidant activity, carotenoids, chlorophyll, principal component analysis, processing stabilization, polyphenolic extracts, refined olive oil, sunflower oil, quality indices

## Abstract

The current research aims to monitor the physicochemical changes in various varieties of extra virgin olive oils (EVOOs) supplemented with exogenous polyphenolic extract from olive fruit, enriched with hydroxytyrosol (HTyr) and its derivatives, compared to numerous refined olive oils, sunflower oil, and high oleic sunflower oil under different deep-frying conditions (170–210 °C for 3 to 6 h, with/without added HTyr. Acidity, K_232_, K_270_, ∆K, peroxide value (PV), anisidine value (AnV), TOTOX, refractive index (RI), carotenoids, chlorophyll, and antioxidant capacity using DPPH (2,2-diphenyl-1-picrylhydrazyl) approach were evaluated. The results show that EVOO varieties generally exhibit lower acidity and thermal degradation compared to refined olive oils, particularly when deep-fried at 170 °C for 3 h with exogenous HTyr (the best treatment). Royuela, Koroneiki, Empeltre, Manzanilla, and Arbosana EVOO varieties demonstrated lower K_232_ values (1.36, 1.67, 1.79, 1.82, and 1.81, respectively). Under optimal deep-frying conditions, all EVOO varieties fell within the standard K_232_ limit for EVOO (≤2.5), except for Cornicabra. Regarding K_270_, only Royuela (0.11) and Manzanilla (0.22) were below the standard limit of ≤0.22. These two varieties also exhibited the lowest ΔK values (0.00). The findings further revealed that Royuela, Koroneiki, and Manzanilla had the lowest TOTOX values, with 20.76, 23.38, and 23.85, respectively. Moreover, Koroneiki and Arbosana had the highest carotenoid ratios, with values of 17.5 mg/kg and 13.7 mg/kg, respectively. Koroneiki, Arbosana, and olive oil 1° also displayed the highest chlorophyll concentrations, with values of 50.2, 53.7, and 47.5 mg/kg, respectively. Furthermore, the findings from the best deep-frying treatment indicated that all olive oil categories exhibited high scavenging radical activity toward DPPH, even in refined olive oil categories and low-quality original olive oil due to the addition of HTyr. In conclusion, deep-fried EVOOs enriched with HTyr at 170 °C/3 h are thermally stable, exhibiting low hydrolysis, low oxidation, higher antioxidant potential, and stable chlorophyll and carotenoid levels. The addition of HTyr to deep-frying oils not only enhances the health benefits of EVOO, supporting EFSA health claims but also acts as a promising stabilizer for the olive oil industry, particularly under high-temperature processing conditions over prolonged periods. This highlights its potential for industrial use as a natural alternative to synthetic antioxidants, not only for olive oil but also for other edible oils, with practical applications in the food industry to improve the quality and stability of frying oils.

## 1. Introduction

Olive oil is highly valued for its unique bioactive components. Extra virgin olive oil (EVOO), a key part of the Mediterranean diet, promotes health through its high monounsaturated fats (MUFs) and polyphenols like oleocanthal, tyrosol (Tyr), hydroxytyrosol (HTyr), oleuropein (OLE), and carotenoids (β-carotene and lutein). These compounds provide antioxidant and anti-inflammatory benefits, counteracting oxidative stress and inflammation, which are critical in aging and chronic diseases. EVOO also enhances the body’s defenses by activating the Nrf-2 pathway and suppressing the pro-inflammatory NF-κB pathway [1]. Recent research highlights its significant health benefits, such as reducing the risk of cardiovascular disease, diabetes, and cancer. Additionally, olive oil consumption has been shown to improve biomarkers like inflammatory markers and lipid profiles, further supporting its role in promoting overall health [2]. EVOO may slow Alzheimer’s disease progression by inhibiting amyloid-β and tau aggregation, though its exact mechanisms are unclear. While individual phenolic compounds show protective effects, it is uncertain if the complex mixtures in dietary EVOO are equally effective, as their activity may be influenced by interactions and chemical modifications [3]. EVOO contains at least 36 identified phenolic compounds, ranging from 0.02 to 600 mg/kg, with compositions influenced by factors such as olive variety, genetics, environment, harvest timing, storage, and extraction methods [4].

Frying oils undergo significant changes during use, including water vapor transfer and degradation, especially with repeated use. Prolonged exposure to high temperatures can degrade unsaturated fatty acids and essential nutrients, trigger oxidation, and produce harmful compounds. Oils are essential in various culinary techniques, such as unheated applications in salads, high-heat stir-frying, pan-frying with moderate heat, and deep-frying at high temperatures (around 180 °C). However, these processes can impact both food quality and health by generating harmful compounds. Thus, improving the stability and quality of used oils is crucial [5,6]. Additionally, while endogenous phenolic compounds can significantly inhibit thermo-oxidative degradation of oils under deep-frying conditions, their concentrations in common vegetable oils are relatively low. As a result, their effectiveness during frying is limited [7,8]. Therefore, one promising solution to improve the stability and quality of frying oils is to enrich them with natural external plant extracts that are high in natural antioxidants.

HTyr is derived from oleuropein during olive maturation, oil storage, and table olive preparation, contributing to the complex flavor of olives and olive oil. HTyr and other phenolic compounds are found in olive oil byproducts, including pomace oil and wastewater. These byproducts are a major source of HTyr [9]. HTyr is also present in olive oil, with concentrations ranging from 1.4 to 5.6 mg/kg, and is found in olive leaves, where it coexists with compounds like OLE. As olives mature, OLE concentration decreases, while HTyr levels rise due to OLE hydrolysis [10].

A recent study found that both EVOO and its polyphenol HTyr have anti-diabetic effects in a rat model of type 2 diabetes (T2D). Treatment with EVOO and HTyr improved glucose tolerance and insulin receptor expression and reduced oxidative stress. EVOO reduced muscle lipids, while HTyr enhanced insulin sensitivity in adipose tissue and supported β-cell survival. Molecular studies also showed hydroxytyrosol’s strong binding to key diabetic proteins, indicating its potential to influence diabetic pathways [11]. Additionally, another study investigated the effects of hydroxytyrosol-rich olive mill wastewater (HTyr-OMWW) on diabetic retinopathy (DR) in Psammomys obesus, a model of DR. The results showed that HT-OMWW has anti-obesity, hypoglycemic, and hypolipidemic effects. Long-term administration of HT-OMWW reduces retinal glial reactivity and microglial count and restores glutamate homeostasis and synaptic function in diabetic animals. These findings suggested that HT-OMWW has promising anti-diabetic, anti-dyslipidemic, and neuroprotective potential for treating DR [12]. Furthermore, HTyr is a potent antioxidant that scavenges free radicals and protects DNA from oxidative damage by activating Nrf2. It also possesses analgesic and anti-inflammatory properties and inhibits colon and breast cancer cell growth by regulating gene expression and promoting pro-apoptotic effects [13].

The European Food Safety Authority (EFSA) has documented the health benefits of olive oil polyphenols, stating they help protect blood lipids from oxidative stress. The claim can be made for olive oil containing at least 5 mg of HTyr and its derivatives per 20 g of oil, with a daily intake of 20 g required [14]. In addition, HTyr is a powerful antioxidant primarily found in olive leaves and fruits [15]. It has various health benefits, including anti-proliferative, lipid-regulating, and anti-inflammatory properties, and prevents cardiovascular diseases. HTyr can be extracted from wastewater produced during olive oil production, with up to 98% of it found in the wastewater [16].

Based on the above discussion, the current research aims to monitor the physicochemical changes in various varieties of EVOOs supplemented with exogenous polyphenolic extracts, specifically olive fruit extract (OFE) enriched with HTyr and its derivatives. These oils were compared to various refined olive oils, sunflower oil, and high-oleic sunflower oil under different deep-frying conditions (170–210 °C for 3 to 6 h), with or without added HTyr. The addition of HTyr to deep-frying oils not only enhances the health benefits of EVOO and other olive oil types, supporting the EFSA health claims, but also serves as a promising stabilizer for the olive oil industry, particularly under high-temperature processing conditions for a prolonged time.

## 2. Materials and Methods

### 2.1. Reagents and Materials

Ethanol (with purity ≥ 99.93%), chloroform (with purity ≥ 99%), NaOH (0.1 N), and cyclohexane (≥99.8%) were purchased from VWR (VWR International, Fontenay-Sous-Bois Cedex, France). Glacial acetic acid (≥99%) was brought from Fisher Scientific Ltd. (Loughborough, UK). Diethyl ether (with purity ≥ 99.8%) was supplied by Honeywell Riedel-de Haen GmbH, Seelze, Germany. Isooctane (2,2,4-Trimethylpentane) with 99.8% purity was bought from Scharlab S.L., Gato Pérez, 33, Barcelona, Spain. Potassium iodide (≥99.0%), DPPH (2,2-diphenyl-1-picrylhydrazyl) with 99.13% purity, *p*-anisidine (99%), and sodium thiosulfate (Na_2_S_2_O_3_·5H_2_O) with 99% purity were purchased from Sigma-Aldrich (Saint Louis, MO, USA). Methanol with ≥99.9% purity was provided by Fisher Scientific Ltd. (Loughborough, UK). Ultrapure water was obtained from a MilliQ system (Millipore, Bedford, MA, USA).

### 2.2. Olive Fruit Extract (OFE) and Oil Sampling

OFE, containing 20% hydroxytyrosol, was obtained from Natac BioTech, Madrid, Spain. The OFE was stored at approximately 7 °C for further processing and analysis. Additionally, four different categories of olive oil and 2 classes of sunflower oil were studied in this research, as follows:

I. Nine different EVOO varieties, representing the widely consumed Spanish EVOOs, were selected for this study: (1) EVOO Picual (supplied by La casa del aceite, Navarra, Spain), (2) EVOO Cornicabra (supplied by COOSUR, Jaén, Spain), (3) EVOO Empeltre (supplied by La casa del aceite, Navarra, Spain), (4) EVOO Arbequina (supplied by COOSUR, Jaén, Spain), (5) EVOO Hojiblanca (supplied by COOSUR, Jaén, Spain), (6) EVOO Manzanilla Cacereña (supplied by Aceite Artajo, Navarra, Spain), (7) EVOO Royuela/Arróniz (supplied by Aceite Artajo, Navarra, Spain), (8) EVOO Koroneiki (supplied by Aceite Artajo, Navarra, Spain), (9) EVOO Arbosana (supplied by Aceite Artajo, Navarra, Spain);
II. One EVOO mixed with refined olive oil (ROO), e.g., olive oil 1° (with maximum %acidity 1) (La Masia, Oleo Masia, S.A. Sevilla, Spain);
III. One Pomace ROO mixed with EVOO, e.g., Pomace olive oil (known as Orujo olive oil in Spain), supplied by Simply, Spain);
IV. One virgin olive oil (VOO) mixed with ROO, e.g., olive oil 0.4° (with maximum %acidity 0.4) (purchased from La Española Oils, Seville, Spain);
V. One refined sunflower oil (RSO) (supplied by Abaco, Tarragona, Spain);
VI. One refined high oleic sunflower oil (RSOHO) (bought from Mercadona, Logroño, Spain).

Thus, 11 samples from each olive oil class were studied, including Control 1 (used as the control for Experiments 1–4), which refers to original, non-supplemented, non-deep-fried olive oil. Additionally, supplemented oil refers to non-deep-fried olive oil that has been enriched with olive fruit extract, which is also used in the preparation of Control 2. Control 2 (used as the control for Experiments 5–8) is a mixture of Control 1 and the supplemented oil, resulting in total polyphenol content of up to 650 mg/kg, and 8 samples subjected to deep-frying (D–F) experiments under varying conditions (time, temperature, and polyphenol addition) (see Table S1). Additionally, five samples were studied from each sunflower oil category, comprising one control and four different experiments (see Table S2). In total, 142 samples were collected for this study, including oils used in deep-frying (D–F) experiments and control samples. The analysis of each oil samples was carried out in triplicate.

### 2.3. Olive Oil Fortification Process with Hydroxytyrosol Extract

HTyr was employed to enhance olive oil varieties with OFE as an exogenic natural biophenol source, up to a concentration of 650 mg/kg, following the fortification method conducted by Mehany et al. [14]. This level was selected to replicate the polyphenol content of oils that are naturally rich in these compounds. OFE serves as a rich source of HTyr and its derivatives. Due to the polarity of HTyr, exogenous OFEs are water-soluble but only minimally soluble in oil. Therefore, OFE was initially dissolved in water (10% *w*/*v*); after that, the solution was stirred mechanically at room temperature using a magnetic stirrer (IKA-WERKE, Staufen, Germany). In addition, the resulting aqueous emulsion was used to fortify the olive oils (2 OFE: 5 olive oil *w*/*w*), and the oil/water solution was then stirred mechanically at room temperature for 60 min. Following centrifugation (9961× *g*/20 min using Sorvall RC-6 Plus, Thermo Scientific, Dreieich Germany), further 2 phases were obtained: the supernatant containing the enriched olive oil and the precipitate containing the aqueous phase. Finally, the supernatant, containing the fortified olive oil, was transferred into an amber container and stored at 7 ± 2 °C for further analysis.

### 2.4. Determination of Total Polyphenols in Olive Oils Supplemented with Hydroxytyrosol Extract by High-Performance Liquid Chromatography (HPLC)

The total phenolic content (TPC) of the original olive oil, the supplemented olive oil, and their mixtures (combining original and supplemented oils up to 650 mg/kg) were determined according to the method of the International Olive Council [17], using an HPLC system (Agilent Technologies, Waldbronn, Germany), equipped with a high-pressure gradient pump, a photodiode array detector (DAD), and a Spherisorb octadecyl silyl 2 (ODS) chromatographic column (250 mm × 4.6 mm ID, 5.0 μm particle size, supplied by Waters, Dublin, Ireland. Oil samples (20 µL) were injected and analyzed in triplicate at 25 °C using a mobile phase of 0.2% H_3_PO_4_/methanol/acetonitrile. A calibration standard (tyrosol and syringic acid) was used, and detection was performed at 280 nm. Total phenolic content was calculated by summing chromatographic peak areas. Additionally, Table S3 presents the total polyphenolic concentration of each original olive oil (Control 1), the supplemented olive oil used to enrich the olive oil with HTyr, and the mixed samples of supplemented and original oils (Control 2), up to 650 mg/kg of polyphenols.

The total phenolic content was calculated by summing the areas of the different chromatographic peaks using the following Equation (1):

(1) $$TPC\left(\frac{mg}{kg}\right)=\frac{\left(\Sigma areas\;of\;the\;peaks\right)\times 1.000\times \frac{RRF\;syringic\;acid}{tyrosol}\times \left(Weight\;of\;syringic\;acid\right)}{\left(Area\;of\;syringic\;acid\right)\times \left(Weight\;of\;the\;sample\right)}$$

where RRF syringic acid/tyrosol represents the multiplication coefficient to express the results in tyrosol.

**Figure 1:**
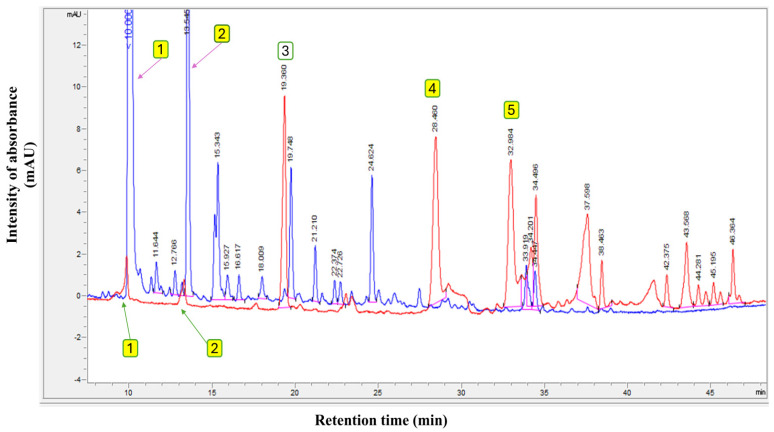
Polyphenol profile of original extra virgin olive oil (Control) (red chromatogram) and pure olive fruit extract used in this study (blue chromatogram). Where (**1**) Hydroxytyrosol; (**2**) Tyrosol; (**3**) Internal standard (syringic acid); (**4**) Oleuropein; and (**5**) Oleocanthal.

Furthermore, the total ion chromatograms (TICs) of the original EVOO (Control and presented in red chromatogram), compared with the olive fruit extract (blue chromatogram) used in this study to fortify the various olive oil types, are presented in Figure 1. The findings in Figure 1 show the polyphenol profiles of EVOO and pure olive fruit extract. The polyphenolic profile of this extract exhibits a qualitative composition comparable to that of EVOO but with a quantitatively higher concentration, measuring 1530.0 mg/kg compared to 262.9 mg/kg in the original EVOO. Based on these results, it can be confirmed that the extracts mainly contain large amounts of hydroxytyrosol and tyrosol, with contents of 904.6 mg/kg and 170.2 mg/kg, respectively, from the total polyphenol content.

**Figure 2:**
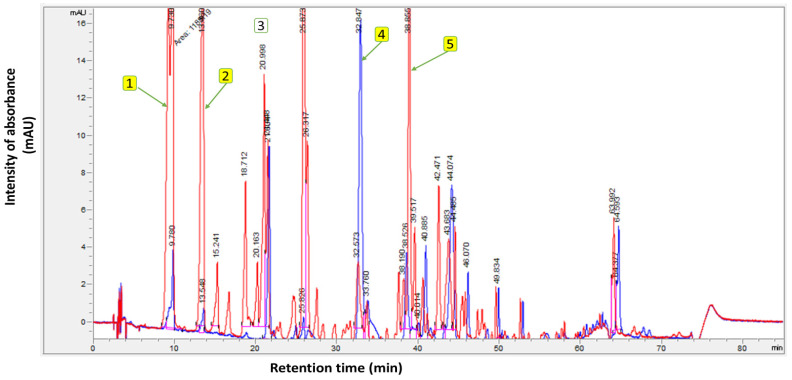
Polyphenol profile of unsupplemented extra virgin olive oil (Control) (blue chromatogram) and supplemented extra virgin olive oil with olive fruit extract (red chromatogram). Where (**1**): Hydroxytyrosol; (**2**): Tyrosol; (**3**): Internal standard (syringic acid); (**4**): Oleuropein; and (**5**): Oleocanthal.

Meanwhile, the TICs of the unsupplemented EVOO (Control), compared with the supplemented EVOO containing OFE, are presented in Figure 2. The polyphenolic analysis was carried out in a randomized order. From the findings in Figure 2, the total phenolic content of the unsupplemented EVOO (control) was 400.6 mg/kg, while the supplemented EVOO exhibited 1211.1 mg/kg. The chromatograms highlight the main components of the supplemented EVOO with OFE—namely, hydroxytyrosol and tyrosol.

### 2.5. The Experimental Design

This study employed a full factorial experimental design, with a 2^3^ design used for olive oil classes and a 2^2^ design used for sunflower oil classes, following the methodology described by Box et al. [18]. This design enabled the evaluation of both the direct effects of investigated variables and their potential interactions. The experimental setup, including the factors and their respective levels for each olive oil type, is detailed in Table S1, while Table S2 provides the design for sunflower oil categories. For olive oil experiments, three factors were selected: frying time (x_1_), frying temperature (x_2_), and the addition of a natural extract enriched with polyphenols, specifically HTyr (x_3_). For sunflower oil experiments, only frying time and temperature were included as factors, as refined sunflower oil lacks natural phenolic content. Each factor was tested at two coded levels (−1 and +1): 3 and 6 h for frying time, 170 °C and 210 °C for frying temperature, and 0 mg/kg and 650.0 mg/kg (supplemented oil) for polyphenol addition. Additionally, a polyphenol level of 0 mg/kg indicates that no exogenous OFE was added during the experiment. In such cases, the samples contained only their intrinsic polyphenol content, which varied widely across samples, as shown in Table S3.

### 2.6. Deep-Frying Process

Different categories of olive oil and sunflower oil, as illustrated in Table S3, were heated using a Soxhlet heating instrument (SELECTA, Barcelona, Spain). The setup included an ELECTTEMP-BASIC (SELECTA, Barcelona, Spain) to control the heating duration and monitor the temperature with a thermometer throughout the experiments. For each deep-frying (D–F) experiment, 0.4 L of each oil type was placed in the fryer and heated continuously at two temperature ranges: 170 ± 10 °C for 3 and 6 h, and 210 ± 10 °C for 3 and 6 h. Following the D–F process, conducted under various conditions (oil type, frying time, frying temperature, and added natural antioxidants), 400 mL of the oil was collected in amber glass vials. These samples were used to monitor oil degradation and analyze the dynamic changes in rancid oil. The samples were kept cold at 5 °C in an amber glass to avoid advanced rancidity before the assessments. Additionally, the physicochemical changes and antioxidant potential of the deep-fried oils were assessed for both control (non-fried) and fried samples across all oil categories.

### 2.7. Physicochemical Characteristics of Deep-Fried Vegetable Oils

The quality indices of the deep-fried edible oils, such as free acidity (% oleic acid), PV, and UV–Vis spectrophotometric characteristics at 270 nm, 232 nm, and ΔK, were measured [19].

#### 2.7.1. Acidity

The free acidity was measured as follows:

(2) $$Acidity\;(\%\;of\;oleic\;acid)=\frac{V\times N\times 28.2}{m}$$

where V is the volume of NaOH consumed (mL); N is the normality of NaOH, and m is the mass of the oil sample (g).

#### 2.7.2. Peroxide Value

PV was measured according to the following equation:

(3) $$PV(mEq\;O_{2}/kg)=\frac{V\times N\times 1000}{m}$$

where V is the volume (mL) of Na_2_S_2_O_3_·5H_2_O (blank corrected); N is the normality of sodium thiosulfate (0.1 N), and m is the mass of the oil sample (g).

#### 2.7.3. Conjugated Trienes and Conjugated Dienes

The absorption coefficients for conjugated trienes (K_270_) and conjugated dienes (K_232_) were measured using a UV–VIS spectrophotometer (Hewlett Packard, Waldbronn, Germany) and expressed as specific extinction coefficients, calculated using the following equation:

(4) $$K\lambda =\frac{Ab\lambda }{C\times L}$$

where Kλ = spectrophotometric absorption in the ultraviolet region (232 or 270); Ab = absorbance; C = concentration of the prepared solution in g/100 mL (1.0%), and L = cuvette path length (10 mm).

The spectrophotometric analysis of olive and sunflowers oil involves determining ΔK, defined by the following equation:

(5) $$\Delta K=\frac{K270-((K266)+(K274))}{2}$$

### 2.8. Anisidine Value (AnV)

The anisidine value was determined according to AOCS Official Method Cd 18–90 (AOCS) [20] by mixing 0.5 g of each oil sample with 25 mL of isooctane containing 1 mL of *p*-anisidine reagent. The absorbance of the mixture was then measured at 350 nm using a spectrophotometer, with the *p*-anisidine reagent in the reference cuvette as the blank.

The values of *p*-anisidine value are expressed in mg/kg and calculated using the following equation:

(6) $$p-anisidine\;value=\frac{25\times \left(1.2As-Ab\right)}{m}$$

where

As = Absorbance of the oil solution after reaction with the *p*-anisidine reagent;
Ab = Absorbance of the oil solution in the solvent (isooctane);
m = Mass of the sample in grams.

### 2.9. TOTOX

The TOTOX value is estimated according to Aşkın & Kaya [21] using the following equation to determine the total oxidation value of the oil samples:

(7) TOTOX value=AnV+2PV

where TOTOX: Total oxidation; AnV: Anisidine value; PV: Peroxide value.

### 2.10. Refractive Index

The refractive index (RI) of the examined olive and sunflower oils was measured as described by El Sohaimy et al. [22]. The oil was placed on the refractometer’s prism and tempered at a specified temperature (below 40 °C) for 2 min. The refraction of light by the oil was then measured and converted to the refractive index. For the calculations, a correction factor of 0.0003385 was applied for oils. The formula for determining the RI is as follows:

(8) $$RI=R\prime +K\left(T\prime -T\right)$$

where RI = Refractive index; R′ = Refractive index (RI) read from the instrument; K factor = 0.0003385 at (20 °C–40 °C).

### 2.11. Carotenoids and Chlorophyll

The total contents of carotenoids and chlorophyll in several vegetable oil samples were measured using the absorption spectrophotometry technique, following the procedure of Cayuela et al. [23], at 472 nm and 670 nm for carotenoids and chlorophylls, respectively, from the pigment extract in cyclohexane. Briefly, 7.5 g of the oil sample was weighed and dissolved in 25 mL of cyclohexane in a volumetric flask, then stirred to ensure homogenization. The sample was then analyzed using the spectrophotometer. The results are expressed in mg/kg of oil. All samples were measured in triplicate. The formula for determining the pigment content is as follows:

(9) $$Carotenoides(k470)=\frac{Ab\times \frac{250}{2000}\times 1000}{P}$$

(10) $$Chlorophylls(K670)=\frac{Ab\times \frac{250}{613}\times 1000}{P}$$

where Ab = The absorbance provided by the wavelength spectrophotometer at 470 or 670 nm; P = Sample weight.

### 2.12. Extraction of Phenolic Compounds

Firstly, 2 g from each oil sample treated under thermal stress and non-fried oil (controls) were weighed in a test tube. After that, 5 mL of 80/20 (*v*/*v*) methanol/water was added to each sample, and the mixtures were stirred vigorously (Heidolph, Schwabach, Germany) for 1 min. Next, the shacked samples were sonicated for 15 min at 30 °C (SELECTA, Barcelona, Spain). Afterward, the sonicated samples were centrifuged (Eppendorf GmbH, Hamburg, Germany) at 4193× *g* for 25 min. An aliquot of the supernatant was then filtered before being analyzed for DPPH scavenging activity [17].

### 2.13. Antioxidant Activity (% DPPH Scavenging Activity)

The antioxidant capacity of oil samples was determined by DPPH assay [22]. Firstly, 0.15 mM DPPH methanolic solution was prepared by dissolving 0.00394 g of DPPH in 100 mL methanol. For the antioxidant activity analyses, 1 mL of each oil methanolic extract was added to 0.5 mL of DPPH and mixed vigorously. The mixture was incubated for 30 min at an ambient temperature in the dark. The absorption was measured at 517 against a control prepared with 1 mL methanol plus 0.5 mL of DPPH, and the antioxidant capacity was calculated from the following Equation (11). Before measuring the control and samples in the DPPH analysis, a blank cuvette containing 1.5 mL of methanol was used to zero the baseline background. Results are expressed as percentages of scavenging, considering that the higher the percentage of inhibition, the greater the antioxidant power of the sample. Also, the results are expressed in the effective concentration of the oil sample to show 50% scavenging (IC_50_). All measurements were carried out in triplicate.

(11) $$DPPH\;scavenging\;activity\left(\%\right)=\frac{A0-A1}{A0}\times 100$$

where A0 = Absorbance of the control; A1 = Absorbance of the sample.

### 2.14. Statistical Analyses

The experimental data were evaluated by SPSS (V28, IBM SPSS Statistics, Chicago, IL, USA), and the mean ± SD was calculated. A one-way analysis of variance (ANOVA) was carried out to measure statistical significance with a *p*-value of less than 0.05. Furthermore, principal component analysis (PCA) was performed by Origin 2022 software (OriginLab, Northampton, MA, USA). To evaluate the significant effects of the independent coded factors on the response for the full factorial experimental designs, factors or combinations of interaction factors with values lower than or equal to the absolute value of (b_123_) were considered non-significant, while values higher than the absolute value of (b_123_) were considered significant. The mean data (responses or physicochemical properties and the oxidation indicators) from the experimental designs of the deep-fried oil samples were analyzed via the Nemrod-W package (version 2000-D, NEMROD-W, Marseille, France) following the method of Mathieu et al. [24]. This was performed to approximate and visualize the interactions between the independent variables and the dependent variables, i.e., physicochemical properties and the oxidation indicators (responses) of each investigated oil.

In addition, to replicate this study with comparable findings, we recommend conducting analyses using different EVOO cultivars from various countries worldwide. This would provide broader insights into the impact of regional and genetic variations on oil stability, antioxidant properties, and overall quality under deep-frying conditions.

## 3. Results

### 3.1. Changes in the Evolution of Acidity in EVOO Varieties, Olive Oils, and Sunflwoer Oils After Deep-Frying

**Figure 3:**
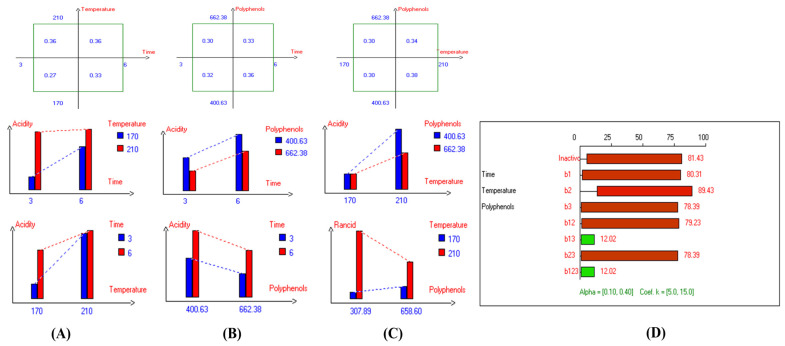
Two-way table illustrating the joined relations between the independent variables and the dependent variable (acidity%) in EVOO Royuela under D–F: (**A**) Time and Temperature, (**B**) Time and Polyphenols, (**C**) Temperature and Polyphenols, and (**D**) Results showing the significant impact of each independent variable and the interactions among the experimental variables on acidity; b signifies a significant change when be >b_123_, while b signifies no significant change when be ≤b_123_; b_1_, b_2_, b_3_ are the main effects of the independent variables, while b_12_, b_13_, b_23_, and b_123_ represent the interaction effects of the independent variables in the experimental design of the D–F process.

The results show that acidity values vary depending on frying conditions, with increases observed as time extended from 3 to 6 h, and temperature rose from 170 °C to 210 °C. The lowest acidity values in most EVOO varieties were achieved at lower temperatures, shorter frying times, and with added olive fruit extract. For instance, in Picual oil, the lowest acidity value (0.32%) was observed with a 3-hour frying time and HTyr extract (658.60 mg/kg) (Figure S1). In Cornicabra, the best acidity values (0.41%) resulted from a combination of 170 °C for 3 h or the addition of polyphenols (up to 658.15 mg/kg) at the same temperature (Figure S2). Empeltre showed its lowest acidity (0.33%) at 170 °C for 3 h, with or without polyphenols (Figure S3), while Arbequina showed 0.36% under the same conditions with added polyphenols (Figure S4). Hojiblanca, despite its generally higher acidity, achieved 0.58% through a combination of 170 °C and polyphenols (655.73 mg/kg) (Figure S5). Manzanilla exhibited the lowest acidity among EVOO varieties, recording 0.22% at 170 °C for 3 h and maintaining stability even without supplementation due to its low initial acidity (0.17%) (Figure S6). Similarly, Royuela demonstrated high thermal stability, with the lowest value (0.27%) at 170 °C for 3 h, attributed to its initial low acidity (0.17%) and high polyphenolic content (400.63 mg/kg) (Figure 3). Pomace olive oil recorded 0.33% acidity under similar conditions or when polyphenols (652.25 mg/kg) were added at 170 °C (Figure S7). Koroneiki achieved 0.27% acidity at 170 °C for 3 h with HTyr extract (663.93 mg/kg) (Figure S8), while Arbosana showed reduced acidity with exogenous polyphenols (666.95 mg/kg) compared to unsupplemented samples (393.00 mg/kg) (Figure S9). A similar trend was noted in olive oils 1° (Figure S10) and 0.4° (Figure S11). High oleic sunflower oil displayed lower acidity (0.17%) compared to regular sunflower oil (0.28%) under the same conditions (Figure S12), thanks to its high oleic acid content, which offers protection against hydrolysis. Overall, the findings indicate that EVOO varieties generally have lower acidity and thermal degradation than refined olive oils, especially when deep-fried at 170 °C for 3 h with exogenous HTyr from olive fruit extract.

### 3.2. Changes in the Evolution of Oxidation Indicators in EVOO Varieties, Olive Oils, and Sunflwoer Oils After Deep-Frying

The results show that the spectrophotometric characteristics (K_232_, K_270_, and ΔK) vary depending on frying conditions, with increases observed as time extended from 3 to 6 h and temperature rose from 170 °C to 210 °C. They also vary depending on the EVOO varieties. The findings on the effect of combined independent variables on the spectrophotometric characteristics and other oxidation indicators (i.e., K_232_, K2_70_, ΔK, PV, AnV, TOTOX, and RI) of Picual oil are shown in Figures S13–S19. Figures S20–S26 present the results for Cornicabra, Figures S27–S33 for Empeltre, Figures S34–S40 for Arbequina, Figures S41–S47 for Hojiblanca, Figures S48–S54 for Manzanilla, Figures S55–S61 for Pomace olive oil, Figures S62–S68 for Koroneiki, Figures S69–S75 for Arbosana, Figures S76–S82 for Olive oil 1°, Figures S83–S89 for Olive oil 0.4°, and Figures S90–S96 for sunflower oil and high oleic sunflower oil.

**Figure 4:**
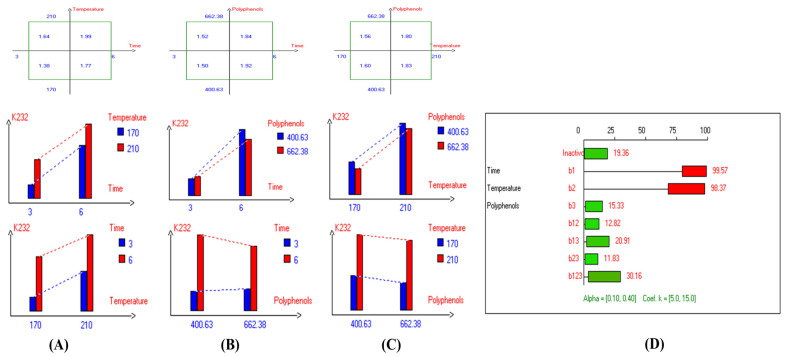
Two-way table illustrating the joined relations between the independent variables and the dependent variable (K_232_) in EVOO Royuela under D–F: (**A**) Time and Temperature, (**B**) Time and Polyphenols, (**C**) Temperature and Polyphenols, and (**D**) Results showing the significant impact of each independent variable and the interactions among combined independent variables on K_232_; b signifies a significant change when be >b_123_, while b signifies no significant change when be ≤b_123_; b_1_, b_2_, b_3_ are the main effects of the independent variables, while b_12_, b_13_, b_23_, and b_123_ correspond to the interaction effects of the independent variables in the experimental design of the D–F process.

**Figure 5:**
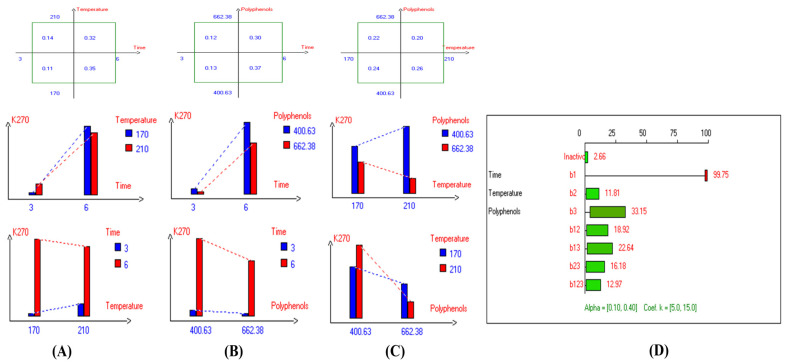
Two-way table illustrating the joined relations between the independent variables and the dependent variable (K_270_) in EVOO Royuela under D–F: (**A**) Time and Temperature, (**B**) Time and Polyphenols, (**C**) Temperature and Polyphenols, and (**D**) Results showing the significant impact of each independent variable and the interactions among combined independent variables on K_270_; b signifies a significant change when be >b_123_, while b signifies no significant change when be ≤b_123_; b_1_, b_2_, b_3_ are the main effects of the independent variables, while b_12_, b_13_, b_23_, and b_123_ represent the interaction effects of the independent variables in the experimental design of the D–F process.

**Figure 6:**
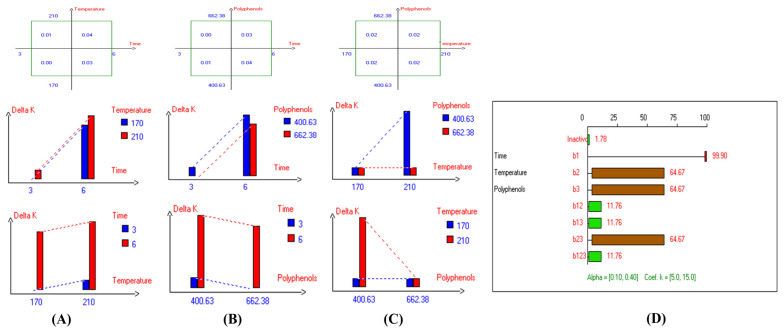
Two-way table illustrating the joined relations between the independent variables and the dependent variable (∆K) in EVOO Royuela under D–F: (**A**) Time and Temperature, (**B**) Time and Polyphenols, (**C**) Temperature and Polyphenols, and (**D**) Results showing the significant impact of each independent variable and the interactions among combined independent variables on ∆K; b signifies a significant change when be >b_123_, while b signifies no significant change when be ≤b_123_; b_1_, b_2_, b_3_ are the main effects of the independent variables, while b_12_, b_13_, b_23_, and b_123_ represent the interaction effects of the independent variables in the experimental design of the D–F process.

The present results indicate that the lowest primary and secondary oxidation compound values in most EVOO varieties were achieved at lower temperatures, shorter frying times, and with or without the addition of olive fruit extract. Notably, remarkable stability was observed when the oils were supplemented with hydroxytyrosol (HTyr). Moreover, at high temperatures and prolonged frying times, the oils fortified with HTyr exhibited greater stability compared to non-supplemented ones under the same conditions. For instance, Royuela, Koroneiki, Empeltre, Manzanilla, and Arbosana EVOO varieties showed lower K_232_ values (1.36, 1.67, 1.79, 1.82, and 1.81, respectively) (Table 1). Under the best deep-frying conditions, as shown in Table 1, all EVOO varieties fall within the standard limit of K_232_ for EVOO (≤2.5), except for Cornicabra. Regarding K_270_ (Table 1), only Royuela (0.11) and Manzanilla (0.22) fall under the standard limits: ≤0.22. Similarly, these two varieties exhibited the lowest ΔK value (0.00). In summary, Royuela demonstrated noticeable and remarkable thermal oxidation stability in terms of K_232_, K_270_, and ΔK values (Figure 4, Figure 5, and Figure 6, respectively).

In sum, the spectrophotometric parameters K_232_, K_270_, and ΔK are key indicators of EVOO’s oxidative stability and compliance with quality standards. Maintaining these values within regulatory limits ensures higher stability and prolonged usability in frying. Among the tested varieties, Royuela exhibited exceptional thermal oxidation resistance, reinforcing its suitability for high-temperature applications. The results show that the lowest primary and secondary oxidation compound values in most EVOO varieties were achieved at lower temperatures, shorter frying times, and with added olive fruit extract. In this regard, the findings (Table 1) indicate that Royuela, Koroneiki, and Manzanilla exhibited the lowest TOTOX values, measuring 20.76, 23.38, and 23.85, respectively.

**Figure 7:**
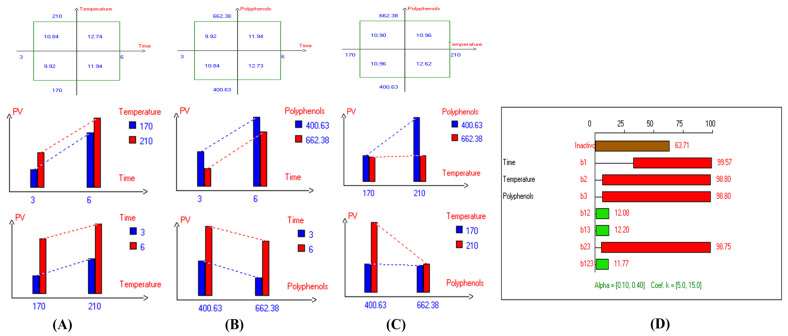
Two-way table illustrating the joined relations between the independent variables and the dependent variable (peroxide value (mEqO_2_/kg)) in EVOO Royuela under D–F: (**A**) Time and Temperature, (**B**) Time and Polyphenols, (**C**) Temperature and Polyphenols, and (**D**) Results showing the significant impact of each independent variable and the interactions among combined independent variables on peroxide value; b signifies a significant change when be >b_123_, while b signifies no significant change when be ≤b_123_; b_1_, b_2_, b_3_ are the main effects of the independent variables, while b_12_, b_13_, b_23_, and b_123_ represent the interaction effects of the independent variables in the experimental design of the D–F process.

**Figure 8:**
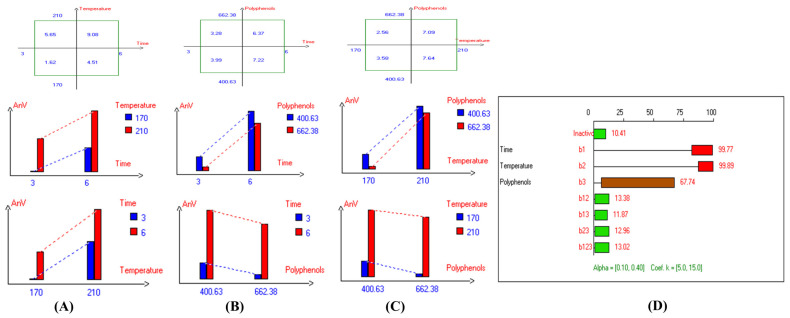
Two-way table illustrating the joined relations between the independent variables and the dependent variable (anisidine value (mg/kg)) in EVOO Royuela under D–F: (**A**) Time and Temperature, (**B**) Time and Polyphenols, (**C**) Temperature and Polyphenols, and (**D**) Results showing the significant impact of each independent variable and the interactions among combined independent variables on anisidine value; b signifies a significant change when be >b_123_, while b signifies no significant change when be ≤b_123_; b_1_, b_2_, b_3_ are the main effects of the independent variables, while b_12_, b_13_, b_23_, and b_123_ represent the interaction effects of the independent variables in the experimental design of the D–F process.

**Figure 9:**
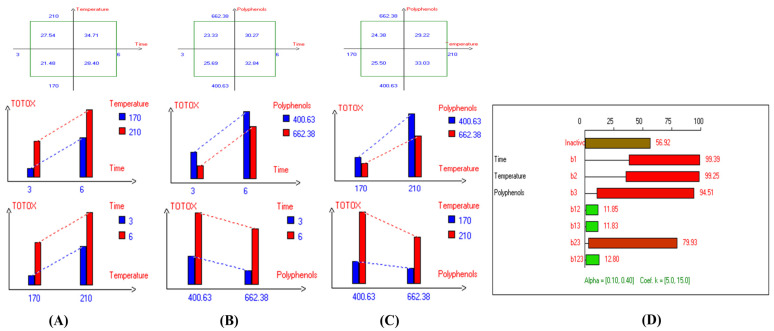
Two-way table illustrating the joined relations between the independent variables and the dependent variable (TOTOX) in EVOO Royuela under D–F: (**A**) Time and Temperature, (**B**) Time and Polyphenols, (**C**) Temperature and Polyphenols, and (**D**) Results showing the significant impact of each independent variable and the interactions among combined independent variables on TOTOX; b signifies a significant change when be >b_123_, while b signifies no significant change when be ≤b_123_; b_1_, b_2_, b_3_ are the main effects of the independent variables, while b_12_, b_13_, b_23_, and b_123_ represent the interaction effects of the independent variables in the experimental design of the D–F process.

**Figure 10:**
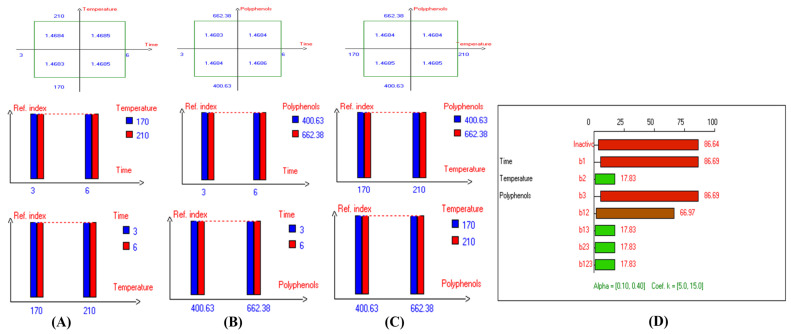
Two-way table illustrating the joined relations between the independent variables and the dependent variable (refractive index) in EVOO Royuela under D–F: (**A**) Time and Temperature, (**B**) Time and Polyphenols, (**C**) Temperature and Polyphenols, and (**D**) Results showing the significant impact of each independent variable and the interactions among combined independent variables on refractive index; b signifies a significant change when be >b_123_, while b signifies no significant change when be ≤b_123_; b_1_, b_2_, b_3_ are the main effects of the independent variables, while b_12_, b_13_, b_23_, and b_123_ represent the interaction effects of the independent variables in the experimental design of the D–F process.

In addition, the refractive index (RI) reflects changes in the composition of EVOO due to rancidity, the hydrolysis of fatty acids, and the formation of oxidation products. High RI values can indicate oxidation or degradation, making it a valuable complementary quality measurement tool. Furthermore, the present findings (Table 1) show that the lowest RI was recorded in Arbosana oil deep-fried at 170 °C for 3 h with added HTyr, with a value of 1.4679, while other EVOOs exhibited relatively stable RI values ranging from 1.4682 to 1.4684. On the other hand, Hojiblanca recorded the highest RI (1.4691), which could be attributed to the lower quality of this original variety compared to other varieties. This indicates that the EVOO variety and the initial quality of the oil before deep-frying play a crucial role in determining oil stability under high thermal processes. Additionally, Pomace olive oil exhibited an RI of 1.4690, reflecting the low quality of this oil. Moreover, sunflower oil showed high RI values of 1.4709 and 1.4731 for high oleic sunflower oil and regular sunflower oil, respectively, indicating that the high oleic acid category demonstrated greater stability. Overall, the findings indicate that EVOO varieties generally have lower oxidation and thermal degradation than refined olive oils, especially when deep-fried at 170 °C for 3 h with exogenous HTyr from olive fruit extract. Additionally, amongst all EVOO varieties, Manzanilla and Royuela exhibited great stability. In summary, Royuela demonstrated noticeable and remarkable thermal oxidation stability in terms of PV, AnV, TOTOX, and RI values (Figure 7, Figure 8, Figure 9, and Figure 10, respectively).

### 3.3. Changes in the Carotenoids and Chlorophyll in EVOO Varieties, Olive Oils, and Sunflwoer Oils After Deep-Frying

The findings reveal that carotenoid concentrations depend on frying conditions, showing an increase as frying time extended from 3 to 6 h and temperature rose from 170 °C to 210 °C. In most EVOO varieties, the highest carotenoid concentrations were achieved under lower temperatures, shorter frying durations, and with the addition of olive fruit extract. For instance, in Picual oil, the highest carotenoid concentration (9.1 mg/kg) was observed after 3 h of frying with HTyr extract (658.6 mg/kg) (Figure S97). Additionally, the maximum chlorophyll concentration (29.1 mg/kg) was recorded at 170 °C after 3 h of frying (Figure S98). In Cornicabra oil, the highest carotenoid concentration (8.1 mg/kg) was recorded at 170 °C after 3 h of frying (Figure S99). Similarly, the maximum chlorophyll concentration (29.8%) was observed under the same conditions (Figure S100). In Empeltre oil, the highest carotenoid concentration (9.4 mg/kg) was observed after 3 h of frying with HTyr extract (647.2 mg/kg) (Figure S101). Similarly, the maximum chlorophyll concentration (29.8 mg/kg) was recorded under the same deep-frying conditions (Figure S102). Similar to the Empeltre variety, Arbequina also demonstrated the highest carotenoid (Figure S103) and chlorophyll (Figure S104) ratios under the same frying conditions. This indicates that the combined effect of shorter frying times and higher phenolic content (663.9 mg/kg) contributes to the enhanced stability of bioactive compounds. Likewise, the Hojiblanca variety exhibited high concentrations of carotenoids (Figure S105) and chlorophyll (Figure S106) after 3 h of frying with the addition of exogenous polyphenols (655.7 mg/kg).

Moreover, the highest carotenoid concentration in the Manzanilla variety (6.65 mg/kg) was recorded after 3 h of frying with added HTyr (661.2 mg/kg) (Figure S107). On the other hand, the maximum chlorophyll concentration (28.7%) was observed under the same conditions (Figure S108). In Pomace olive oil, the highest carotenoid concentration (8.7 mg/kg) was observed at 170 °C after frying with HTyr extract (652.2 mg/kg) (Figure S109). Similarly, the maximum chlorophyll concentration (19.5 mg/kg) was recorded under the same deep-frying conditions (Figure S110). In Koroneiki oil, the highest carotenoid concentration (12.3 mg/kg) was observed at 170 °C after 3 h of frying (Figure S111). Additionally, the maximum chlorophyll concentration (49.5 mg/kg) was recorded at 170 °C of frying with added HTyr (663.9 mg/kg) (Figure S112).

Additionally, Arbosana oil showed the highest carotenoid concentration (17.3 mg/kg) when deep-fried for 3 h with polyphenol content up to 666.9 mg/kg (Figure S113). Meanwhile, the maximum chlorophyll concentration (53.5 mg/kg) was recorded under the same conditions (Figure S114). Therefore, HTyr extract improved the stability of these minor bioactive substances.

In Olive oil 1°, the highest carotenoid concentration (10.6 mg/kg) was observed at 170 °C during deep frying with HTyr extract (653.1 mg/kg) (Figure S115). However, the highest chlorophyll concentration (44.7 mg/kg) was recorded at 170 °C after 3 h of frying (Figure S116). In the case of Olive oil 0.4°, the highest chlorophyll concentration (9.7 mg/kg) was recorded at 170 °C after 3 h of frying (Figure S117). Meanwhile, the highest carotenoid concentration (21.6 mg/kg) was recorded under the same frying conditions (Figure S118).

**Figure 11:**
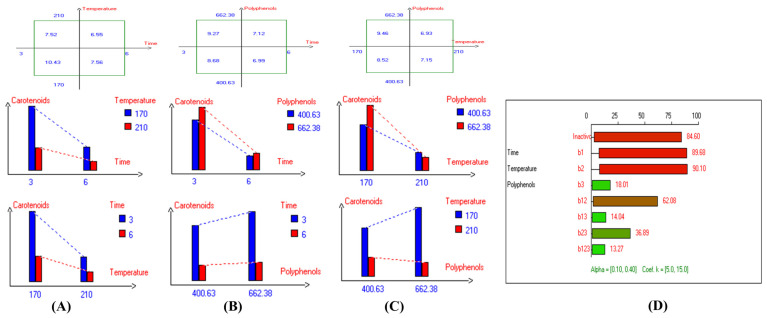
Two-way table illustrating the joined relations between the independent variables and the dependent variable (carotenoids (mg/kg)) in EVOO Royuela under D–F: (**A**) Time and Temperature, (**B**) Time and Polyphenols, (**C**) Temperature and Polyphenols, and (**D**) Results showing the significant impact of each independent variable and the interactions among combined independent variables on carotenoids; b signifies a significant change when be >b_123_, while b signifies no significant change when be ≤b_123_; b_1_, b_2_, b_3_ are the main effects of the independent variables, while b_12_, b_13_, b_23_, and b_123_ represent the interaction effects of the independent variables in the experimental design of the D–F process.

**Figure 12:**
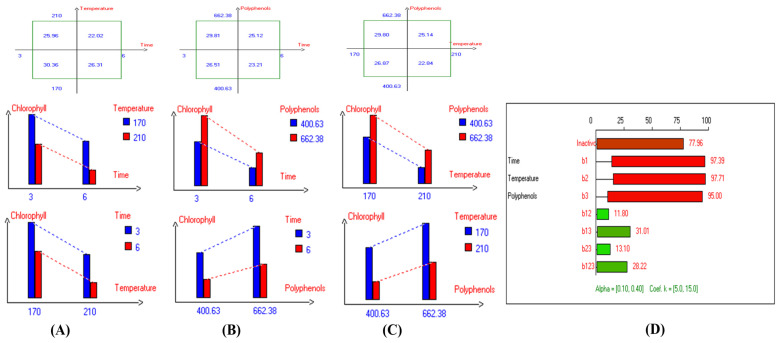
Two-way table illustrating the joined relations between the independent variables and the dependent variable (chlorophyll (mg/kg)) in EVOO Royuela under D–F: (**A**) Time and Temperature, (**B**) Time and Polyphenols, (**C**) Temperature and Polyphenols, and (**D**) Results showing the significant impact of each independent variable and the interactions among combined independent variables on chlorophyll; b signifies a significant change when be >b_123_, while b signifies no significant change when be ≤b_123_; b_1_, b_2_, b_3_ are the main effects of the independent variables, while b_12_, b_13_, b_23_, and b_123_ represent the interaction effects of the independent variables in the experimental design of the D–F process.

Regarding sunflower oil, the highest carotenoid concentration (6.6 mg/kg) was recorded at 210 °C after 3 h of frying (Figure S119A). Meanwhile, the highest carotenoid concentration in high oleic sunflower oil (8.7 mg/kg) was observed under the same frying conditions at 210 °C after 3 h (Figure S119D). Moreover, the highest chlorophyll concentration (15.6 mg/kg) was recorded at 170 °C after 3 h of frying (Figure S120A). Similarly, the highest chlorophyll concentration in high oleic sunflower oil (21.3 mg/kg) was recorded under the same conditions (170 °C after 3 h of frying) (Figure S120D). In summary, and based on the high stability of the Royuela variety compared to other EVOOs, the combined effects of interacting independent variables on carotenoid and chlorophyll concentrations were illustrated in Figure 11 and Figure 12, respectively.

The results indicate that the highest values of minor bioactive compounds, including chlorophyll and carotenoids, in most EVOO varieties were achieved at lower temperatures, shorter frying times, and with the addition of olive fruit extract. As shown in Table 2, Koroneiki and Arbosana EVOO varieties exhibited the highest carotenoid concentrations, with values of 17.5 mg/kg and 13.7 mg/kg, respectively. Additionally, Koroneiki, Arbosana, and Olive oil 1° displayed the highest chlorophyll concentrations, with values of 50.2 mg/kg, 53.7 mg/kg, and 47.5 mg/kg, respectively.

### 3.4. DPPH Scavenging Activity in EVOO Varieties, Olive Oils, and Sunflwoer Oils After Deep-Frying

**Figure 13:**
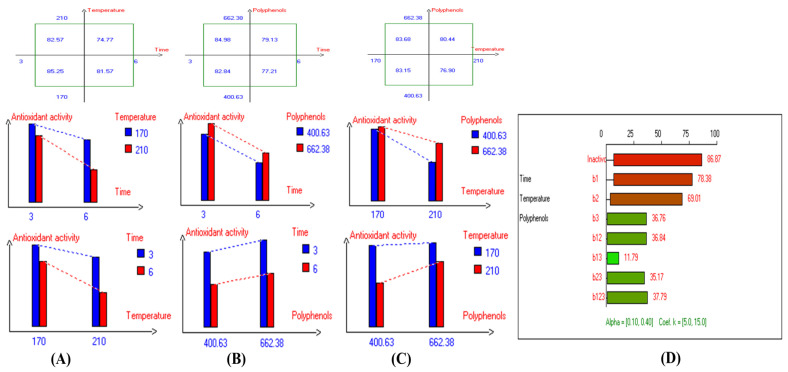
Two-way table illustrating the joined relations between the independent variables and the dependent variable (antioxidant activity %) in EVOO Royuela under D–F: (**A**) Time and Temperature, (**B**) Time and Polyphenols, (**C**) Temperature and Polyphenols, and (**D**) Results showing the significant impact of each independent variable and the interactions among combined independent variables on antioxidant activity; b signifies a significant change when be >b_123_, while b signifies no significant change when be ≤b_123_; b_1_, b_2_, b_3_ are the main effects of the independent variables, while b_12_, b_13_, b_23_, and b_123_ represent the interaction effects of the independent variables in the experimental design of the D–F process.

The findings on the impact of combined independent variables i.e., D–F time, D–F temperature, and polyphenols supplementation on antioxidant activity of oil i.e., Picual, Cornicabra, Empeltre, Arbequina, Hojiblanca, Manzanilla, Pomace olive oil, Koroneiki, Arbosana, Olive oil 1°, Olive oil 0.4°, sunflower oil, and high oleic sunflower oil are shown in Figures S121–S132, respectively. The results also indicate that the lowest primary and secondary oxidation compound values in most EVOO varieties were achieved at lower temperatures, shorter frying times, and with added olive fruit extract. In addition, based on the high stability of the Royuela variety compared to other EVOOs, the combined effects of interacting independent variables on antioxidant activity are illustrated in Figure 13.

The findings of the best deep-frying treatment (Table 2) show that all olive oil categories exhibited high radical scavenging activity toward DPPH, thanks to the added exogenous polyphenols, even in refined olive oil categories and low-quality original olive oil due to the addition of HTyr. However, sunflower oil types showed minimal antioxidant potential, as they were free from the added olive fruit extract. Overall, the findings indicate that EVOO varieties generally exhibit lower oxidation and thermal degradation compared to refined olive oils, such as Olive oil 0.4°, Olive oil 1°, and Pomace olive oil, especially when deep-fried at 170 °C for 3 h with exogenous HTyr from olive fruit extract. Additionally, among all the EVOO varieties, Manzanilla, Royuela, Koroneiki, and Arbosana demonstrated excellent physicochemical stability. Overall, the findings indicate that EVOO varieties generally have lower acidity and thermal degradation than refined olive oils, especially when deep-fried at 170 °C for 3 h with exogenous HTyr from olive fruit extract.

## 4. Discussion

The combined effects of deep-frying (three factors: time, temperature, and polyphenol content) and EVOO variety (nine types) were studied on the essential quality parameters, oxidation indicators, and antioxidant activity of olive oil from the most consumed Spanish EVOO varieties, including Picual, Cornicabra, Empeltre, Arbequina, Hojiblanca, Manzanilla, Royuela, Koroneiki, and Arbosana. These were compared to refined olive oils blended with EVOO or VOO such as (Pomace olive oil, Olive oil 1°, Olive oil 0.4°), sunflower oil, and high oleic acid sunflower oil. Significant interactions were observed between EVOO variety, frying time, frying temperature, and polyphenol content. After deep-frying, acidity values increased significantly in all samples compared to the non-fried oil. This increase results from the transformation of oxidation products into carboxylic acids, with oleic acid being the primary final product. The rise in acidity in the oil samples is attributed to thermal oxidation resulting from the continuous deep-frying process.

The results also indicate that acidity values in EVOO are influenced by frying conditions, particularly time and temperature. Lower acidity values were observed with shorter frying times, lower temperatures, and the addition of olive fruit extract (identified as the best frying conditions). In addition, most EVOO varieties exhibited the best acidity levels under these conditions. For instance, Picual, Cornicabra, Empeltre, Arbequina, and Hojiblanca oils showed the lowest acidity when fried at 170 °C for 3 h, with the addition of exogenous polyphenols. Moreover, Manzanilla exhibited the lowest acidity among EVOOs, maintaining stability even without supplementation due to their initial low acidity. Royuela also demonstrated high thermal stability, attributed to its low initial acidity, high-quality oil, and rich polyphenolic content. In contrast, refined olive oils showed higher acidity and greater thermal degradation. High oleic sunflower oil exhibited lower acidity than sunflower oil, highlighting the protective effect of oleic acid against hydrolysis. Overall, EVOO varieties demonstrated better acidity and thermal stability, particularly when supplemented with exogenous HTyr from olive fruit extract. This is primarily attributed to the antioxidant potential of HTyr, as indicated in the recent literature [9,25].

Recent reports indicate that natural antioxidants from olive stuffs play a significant role in reducing and/or inhibiting oxidation and degradation levels in edible oil. For instance, Harzalli et al. [26] conducted a study in which potatoes were fried in sunflower oil, with and without enrichment, using extracts from Chetoui olive byproducts (leaves and olive mill wastewater). The oils were analyzed before and after in vitro digestion using ¹H NMR spectroscopy. This study found that non-enriched oils generated more aldehydes during frying, while digested potatoes fried in these oils exhibited greater linoleic acid degradation and increased levels of primary oxidation products. In contrast, enriched oils slightly enhanced lipolysis during digestion, indicating improved breakdown. These findings suggest that Chetoui olive byproduct extracts may function as antioxidants, enhancing the quality, safety, and nutritional profile of fried foods. Another study investigated the effect of olive mill wastewater (OMW) extract (100 ppm) as an additive in refined sunflower oil and olive oil to reduce oxidation during the deep-frying of chicken and potatoes [27]. The results showed that OMW extract reduced the formation of polymerized triacylglycerols (pTAG) and aldehydes in sunflower oil but slightly increased these compounds in olive oil. Frying chicken produced more oxidation products compared to frying potatoes. Overall, OMW extract shows potential as a bioactive additive to reduce oxidation in frying oils, with olive oil without additives remaining a healthier choice.

The spectrophotometric characteristics, i.e., K_232_, K_270_, and ΔK, are indices used to measure the oxidation level of olive oil. K_232_ indicates early oxidation or the presence of primary oxidation molecules, while K_270_ reflects advanced oxidation or secondary oxidation compounds. ΔK helps evaluate oil quality; a high ΔK value often suggests blending with refined oils, the presence of degraded oils, or high thermal degradation [28]. Low values of these spectrophotometric indices are typical of high-quality extra virgin olive oil. In this regard, the present results demonstrate that spectrophotometric characteristics, including K_232_, K_270_, and ΔK—indicators of oxidation levels in olive oil—are influenced by frying conditions such as temperature, time, and the presence of olive fruit extract. Lower temperatures (170 °C), shorter frying times (3 h), and the addition of olive fruit extract resulted in lower values of primary and secondary oxidation compounds in most EVOO varieties. The addition of HTyr further enhanced the stability of the oils, particularly at high temperatures and during prolonged frying, compared to non-enriched oils under the same frying conditions. Thus, oils enriched with HTyr exhibited reduced oxidation and degradation. These findings align with the known free radical scavenging capacity of HTyr [29,30].

The results also show that Royuela, Koroneiki, Empeltre, Manzanilla, and Arbosana EVOO varieties exhibited notable thermal stability, with significantly lower oxidation values compared to other EVOOs, especially when supplemented with HTyr. Among these, Royuela demonstrated exceptional thermal oxidation stability, maintaining values within standard limits for K_232_, K_270_, and ΔK. The alterations in free acidity, PV, ultraviolet absorption indicators, TPC, pigment levels, and oxidative stability of olive oil were significantly influenced by the combined effects of variety, geographic origin, and crop season [31]. Moreover, the differences in the stability of EVOOs are likely attributed to variations in minor bioactive compounds concentration, harvest time, oleic acid content, or other environmental factors. High levels of phenolic compounds, particularly from oily sources such as olive fruits, contribute to the greater stability of edible oils [32]. A recent study also demonstrated variations between Empeltre and Arbequina olive oils. The variety was identified as the main factor driving the variation in palmitic, palmitoleic, and linolenic fatty acid content, while the geographic origin primarily influenced the differences in oleic and linoleic fatty acids in Arbequina and Empeltre olive oils. Although the variety significantly affected sterol composition, the production area also had a considerable impact on these oils [33]. Our results align with those of recent reports, which stated that the nutritional and quality characteristics of olive oil are influenced by factors such as the olive tree genotype, fruit maturity stage, agronomic practices, extraction methods, and storage conditions [11]. Thus, this study highlights the importance of choosing olive oil from a specific variety with high polyphenol content, either in its endogenous form or supplemented with external natural antioxidants, as well as ensuring low initial oxidation indicators before using it for deep-frying.

One of the most interesting findings is that olive fruit extract, due to its polyphenolic profile, improved the thermal oxidation stability, in terms of acidity and spectroscopic characteristics, of EVOOs compared to non-enriched ones, when fried at the same time and temperatures. The main groups of phenolic compounds in olive fruit extract that contribute to its antioxidant potential include HTyr, tyrosol (Tyr), verbascoside, pinoresinol, 1-acetoxy-pinoresinol, vanillic acid, chlorogenic acid, *p*-coumaric acid, cinnamic acid, homovanillic acid, caffeic acid, and 4-dihydroxybenzoic acid, Additionally, lignans, secoiridoids, and phenolic derivatives also play a significant role [34]. In addition, phenolic compounds have antioxidant properties determined by their chemical structure. Research indicates they are more effective than butylated hydroxytoluene (BHT), butylated hydroxyanisole (BHA), and tocopherols as stabilizers for frying oils due to their lower volatility during deep-frying. They also inhibit tocopherol oxidation, significantly enhancing its retention in frying oils [32,35].

The peroxide value (PV) is one of the key quality parameters used to monitor lipid oxidation and evaluate the overall quality of EVOO. PV is a primary test for assessing autoxidation, also known as oxidative rancidity. Peroxides act as intermediates in the autoxidation process. Lipid oxidation becomes a significant concern during cooking, especially when oils are reused, as it can impact both the nutritional value and the flavor and aroma of the product [36]. In addition, TOTOX values (total oxidation values) in olive oil measure its thermal–oxidative stability and quality, reflecting its exposure to degradation over time. They are calculated from PV and AnV, where the PV measures primary oxidation products like hydroperoxides, and the AnV measures secondary oxidation products such as aldehydes. Low TOTOX values indicate high-quality thermal stability under deep-frying, fresh oil with minimal oxidation, while high values suggest degradation due to factors like prolonged storage, heat, or light exposure. Monitoring Totox values ensures the oil’s quality, flavor, and compliance with industry standards [37,38].

In this regard, the results show that the TOTOX value progressively increased with longer frying times and higher frying temperatures, primarily due to elevated AnV and PV resulting from continuous deep frying. The current study revealed that the Royuela, Koroneiki, and Manzanilla EVOO varieties had the lowest TOTOX values, highlighting their superior quality and greater resistance to oxidation during frying. A lower TOTOX value reflects better oil quality. Moreover, the TOTOX value demonstrated a linear correlation with PV and AnV, consistent with findings reported by Mohammadi et al. [39], who confirmed significant changes in PV, oxidative indices, TOTOX values, and AnV with increasing heating temperatures (*p* < 0.05).

The refractive index (RI), a distinctive property of each type of oil within specific limits, also served as an important indicator of oil stability [40], with lower values observed in Arbosana oil deep-fried with HTyr and higher RI values in Hojiblanca and Pomace olive oils, indicating lower initial quality. Moreover, the RI values were relatively consistent among EVOOs. The findings further reveal that EVOO varieties, particularly those supplemented with HTyr, exhibit superior oxidation and thermal stability compared to refined olive oils. Additionally, oils such as Arbosana, Koroneiki, Manzanilla, and Royuela demonstrated exceptional stability, highlighting the importance of variety and initial oil quality in determining the stability of EVOO during high-heat processes. The differences in RI values are primarily attributed to variations in the degree of unsaturation, as well as other factors like free fatty acid concentration, oxidation products, and heat treatment conditions [41]. Additionally, EVOOs with high polyphenolic content exhibited greater stability. This is primarily because fried olive oils enriched with HTyr have a higher antioxidant capacity compared to those without supplementation. Recent studies have also reported that HTyr provides several significant technological and health benefits, including antioxidant, anticancer, anti-inflammatory, cartilage-protective, and antiosteoporotic properties. As a potent antioxidant, HTyr demonstrates a 2.45 times greater ability to scavenge DPPH radicals compared to the antioxidant 2,6-di-tert-butyl-4-methylphenol (BHT) [40], making it a viable alternative to traditional chemical antioxidants [42].

Furthermore, the present findings demonstrate that carotenoid concentrations in EVOO are influenced by frying conditions, decreasing as frying time and temperature rise. The highest carotenoid concentrations were observed at lower temperatures, shorter frying durations, and with the addition of olive fruit extract. Among the various EVOO varieties tested, Koroneiki and Arbosana exhibited the highest carotenoid concentrations, while Koroneiki, Arbosana, and Olive oil 1° showed the highest chlorophyll concentrations. Moreover, for specific varieties, Picual, Cornicabra, Empeltre, Arbequina, and Hojiblanca oils showed stable carotenoid and chlorophyll concentrations with HTyr addition, indicating that the combined effect of higher phenolic content and shorter frying durations enhanced bioactive compound stability. Furthermore, sunflower oil, particularly the high oleic acid variant, also showed improved stability of carotenoids and chlorophyll when subjected to deep-frying conditions. Therefore, based on the current investigation, oils supplemented with HTyr extract are of great interest and present a promising strategy for improving stability and protecting minor bioactive compounds, such as pigments, under deep-frying conditions. In this regard, a polyphenolic extract from olive vegetation water (OVW) was tested in ROO to reduce frying-related oxidation [43]. ROO enriched with OVW was compared to ROO with BHT and high-polyphenol EVOO. The polyphenolic extract, at a concentration of 400 mg/kg, effectively reduced tocopherol oxidation and aldehyde emissions during frying at 180 °C, matching the performance of EVOO and surpassing that of BHT. In conclusion, the present study emphasizes the positive impact of lower temperatures, shorter frying times, and olive fruit extract supplementation on maintaining the stability of carotenoids and chlorophyll in various EVOO varieties.

Regarding the antioxidant activity, the results show that lower temperatures, shorter frying durations, and the addition of OFE resulted in lower primary and secondary oxidation compound levels in most EVOO varieties. Among these oils, the Royuela variety demonstrated remarkable stability, with antioxidant activity significantly enhanced by the addition of HTyr. The deep-frying treatments revealed that all EVOO varieties exhibited strong radical scavenging activity toward DPPH, particularly when supplemented with exogenous polyphenols. This activity was observed even in lower-quality oils, such as refined olive oils and those with lower initial quality, thanks to HTyr supplementation and other phenolic compound derivatives in OFE. In contrast, sunflower oil varieties, which lacked olive fruit extract, showed minimal antioxidant potential. Therefore, the addition of HTyr extract to EVOO during deep-frying is of great interest not only for its potent several health benefits but also as a natural antioxidant. This suggests its potential for industrial use as an alternative to synthetic antioxidants, not only for olive oil but also for other edible oils.

In this regard, numerous studies have shown that HTyr, in comparison to other phenolic compounds in olive oil, demonstrates much stronger antioxidant properties, e.g., scavenging free radicals, breaking peroxidative chain reactions, preventing lipid peroxidation, and inhibiting radicals derived from hypochlorous acid [44]. In addition, HTyr treatment has been shown to decrease reactive oxygen species (ROS) production triggered by iron- or copper-induced oxidation of low-density lipoprotein (LDL) in an in vitro model, indicating its metal-chelating properties. Its ability to scavenge or reduce ROS generation was further validated using chemiluminescence methods in both 12-myristate 13-phorbol acetate (PMA)-treated leukocytes and the hypoxanthine/xanthine oxidase cell-free system [45]. Recently, Rubio-Senent et al. [30] illustrated the antioxidant properties of HTyr by describing its effects on free radicals in two primary ways: (1) it has scavenging properties that break the chains of free radicals and (2) it acts as a metal chelator, reducing the harmful effects of free radicals and converting them into stable molecules. Additionally, HTyr exhibits a high oxygen radical absorbance capacity (ORAC) value, highlighting its potent antioxidant potential.

**Figure 14:**
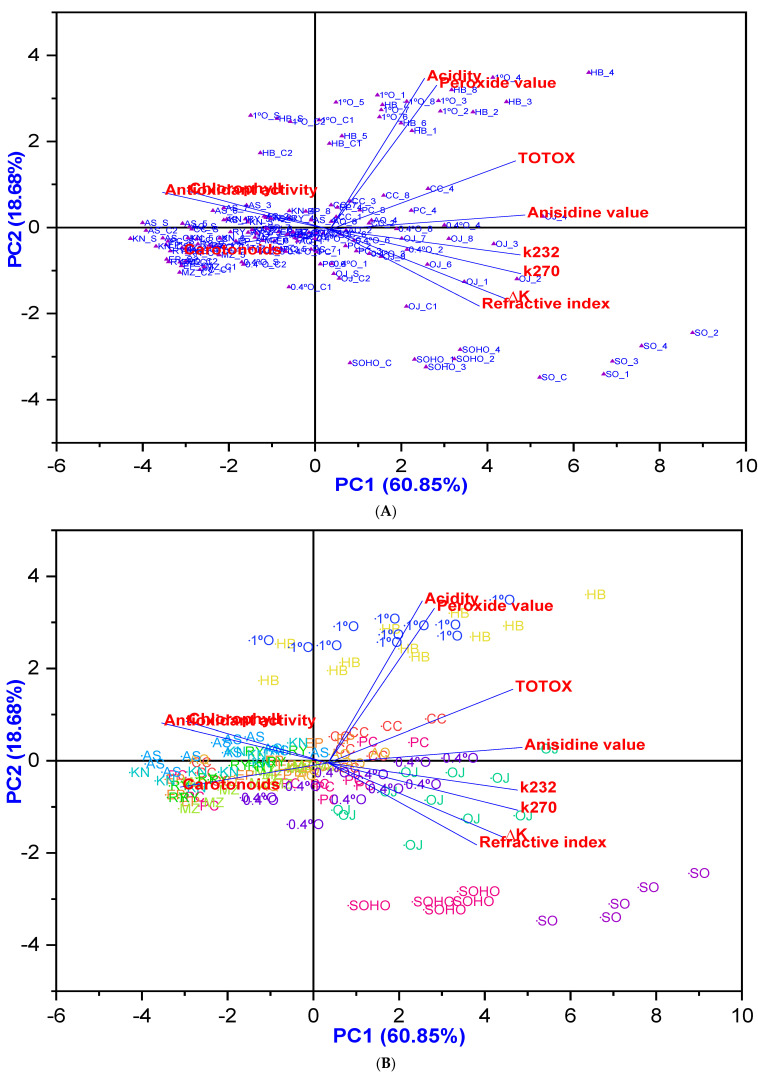
(**A**) PCA biplot (combining score and loading plots) of nine EVOO varieties, compared with various refined olive oils blended with EVOO or VOO, sunflower oil, and high oleic sunflower oil, illustrating the potential clustering of oil samples based on physicochemical and antioxidant activity changes under various deep-frying conditions as well as before the deep-frying. (**B**) PCA clustering analysis based on different oil categories: The PCA clustering analysis categorizes different oils based on their response variables, including acidity, K_232_, K_270_, ∆K, peroxide value (PV), anisidine value (AnV), TOTOX, refractive index (RI), carotenoids, chlorophyll, and antioxidant activity. Each category is represented in a distinct color, allowing for clear differentiation. The oil categories include Picual (PC), Cornicabra (CC), Empeltre (EP), Arbequina (AQ), Hojiblanca (HB), Manzanilla Cacereña (MZ), Royuela/Arróniz (RY), Koroneiki (KN), Arbosana (AS), Olive oil 1° (1°O), Pomace olive oil (or Orujo) (OJ), Olive oil 0.4° (0.4°), sunflower oil (SO), and high oleic sunflower oil (SOHO). Where PC_C1: Picual_Control 1; PC_S: Picual_Supplemented; PC_C2: Picual_Control 2; PC_1: Picual_Exp 1; PC_2: Picual_Exp 2; PC_3: Picual_Exp 3; PC_4: Picual_Exp 4; PC_5: Picual_Exp 5; PC_6: Picual_Exp 6; PC_7: Picual_Exp 7; PC_8: Picual_Exp 8; CC_C1: Cornicabra_Control 1; CC_S: Cornicabra_Supplemented; CC_C2: Cornicabra_Control 2; CC_1: Cornicabra_Exp 1; CC_2: Cornicabra_Exp 2; CC_3: Cornicabra_Exp 3; CC_4: Cornicabra_Exp 4; CC_5: Cornicabra_Exp 5; CC_6: Cornicabra_Exp 6; CC_7: Cornicabra_Exp 7; CC_8: Cornicabra_Exp 8; EP_C1: Empeltre_Control 1; EP_S: Empeltre_Supplemented; EP_C2: Empeltre_ Control 2; EP_1: Empeltre_Exp 1; EP_2: Empeltre_Exp 2; EP_3: Empeltre_Exp 3; EP_4: Empeltre_Exp 4; EP_5: Empeltre_Exp 5; EP_6: Empeltre_Exp 6; EP_7: Empeltre_Exp 7; EP_8: Empeltre_Exp 8; AQ_C1: Arbequina_Control 1; AQ_S: Arbequina_Supplemented; AQ_C2: Arbequina_ Control 2; AQ_1: Arbequina_Exp 1; AQ_2: Arbequina_Exp 2; AQ_3: Arbequina_Exp 3; AQ_4: Arbequina_Exp 4; AQ_5: Arbequina_Exp 5; AQ_6: Arbequina_Exp 6; AQ_7: Arbequina_Exp 7; AQ_8: Arbequina_Exp 8; HB_C1: Hojiblanca_Control 1; Hojiblanca_Supplemented; Hojiblanca_Control 2; HB_1: Hojiblanca_Exp 1; HB_2: Hojiblanca_Exp 2; HB_3: Hojiblanca_Exp 3; HB_4: Hojiblanca_Exp 4; HB_5: Hojiblanca_Exp 5; HB_6: Hojiblanca_Exp 6; HB_7: Hojiblanca_Exp 7; HB_8: Hojiblanca_Exp 8; MZ_C1: Manzanilla_ Control 1; MZ_S: Manzanilla_Supplemented; MZ_C2: Manzanilla_Control 2; MZ_1: Manzanilla_Exp 1; MZ_2: Manzanilla_Exp 2; MZ_3: Manzanilla_Exp 3; MZ_4: Manzanilla_Exp 4; MZ_5: Manzanilla_Exp 5; MZ_6: Manzanilla_Exp 6; MZ_7: Manzanilla_Exp 7; MZ_8: Manzanilla_Exp 8; RY_C1: Royuela_ Control 1; RY_S: Royuela_Supplemented; RY_C2: Royuela_ Control 2; RY_1: Royuela_Exp 1; RY_2: Royuela_Exp 2; RY_3: Royuela_Exp 3; RY_4: Royuela_Exp 4; RY_5: Royuela_Exp 5; RY_6: Royuela_Exp 6; RY_7: Royuela_Exp 7; RY_8: Royuela_Exp 8; OJ_C1: Pomace_Control 1; OJ_S: Pomace_ Supplemented; OJ_C2: Pomace_ Control 2; OJ_1: Pomace_Exp 1; OJ_2: Pomace_Exp 2; OJ_3: Pomace_Exp 3; OJ_4: Pomace_Exp 4; OJ_5: Pomace_Exp 5; OJ_6: Pomace_Exp 6; OJ_7: Pomace_Exp 7; OJ_8: Pomace_Exp 8; KN_C1: Koroneiki_Control 1; KN_S: Koroneiki_Supplemented; KN_C2: Koroneiki_Control 2; KN_1: Koroneiki_Exp 1; KN_2: Koroneiki_Exp 2; KN_3: Koroneiki_Exp 3; KN_4: Koroneiki_Exp 4; KN_5: Koroneiki_Exp 5; KN_6: Koroneiki_Exp 6; KN_7: Koroneiki_Exp 7; KN_8: Koroneiki_Exp 8; AS_C1: Arbosana_Control 1; AS_S: Arbosana_Supplemented; AS_C2: Arbosana_Control 2; AS_1: Arbosana_Exp 1; AS_2: Arbosana_Exp 2; AS_3: Arbosana_Exp 3; AS_4: Arbosana_Exp 4; AS_5: Arbosana_Exp 5; AS_6: Arbosana_Exp 6; AS_7: Arbosana_Exp 7; AS_8: Arbosana_Exp 8; 1°O_C1: Olive 1°_ Control 1; 1°O_S: Olive 1°_Supplemented; 1°O_C2: Olive 1°_ Control 2; 1°O_1: Olive 1°_Exp 1; 1°O_2: Olive 1°_Exp 2; 1°O_3: Olive 1°_Exp 3; 1°O_4: Olive 1°_Exp 4; 1°O_5: Olive 1°_Exp 5; 1°O_6: Olive 1°_Exp 6; 1°O_7: Olive 1°_Exp 7; 1°O_8: Olive 1°_Exp 8; 0.4°O_C1: Olive 0.4°_ Control 1; 0.4°O_S: Olive 0.4°_Supplemented; 0.4°O_C2: Olive 0.4°_ Control 2; 0.4°O_1: Olive 0.4°_Exp 1; 0.4°O_2: Olive 0.4°_Exp 2; 0.4°O_3: Olive 0.4°_Exp 3; 0.4°O_4: Olive 0.4°_Exp 4; 0.4°O_5: Olive 0.4°_Exp 5; 0.4°O_6: Olive 0.4°_Exp 6; 0.4°O_7: Olive 0.4°_Exp 7; 0.4°O_8: Olive 0.4°_Exp 8; SO_C: Sunflower oil_Control; SO_1: Sunflower oil_Exp 1; SO_2: Sunflower oil_Exp 2; SO_3: Sunflower oil_Exp 3; SO_4: Sunflower oil_Exp 4; SOHO_C: Sunflower oil_high oleic acid_Control; SOHO_1: Sunflower oil_ high oleic acid _Exp 1; SOHO_2: Sunflower oil_ high oleic acid_Exp 2; SOHO_3: Sunflower oil_ high oleic acid_Exp 3; SOHO_4: Sunflower oil_ high oleic acid_Exp 4. In addition, for the olive oil categories, Control 1 (used as the control for Experiments 1–4) refers to original, non-deep-fried olive oil. Supplemented oil refers to non-deep-fried olive oil that has been enriched with olive fruit extract, which is also used in the preparation of Control 2. Control 2 (used as the control for Experiments 5–8) is a mixture of Control 1 and the supplemented oil, resulting in a total polyphenol content of up to 650 mg/kg. Exp.1: olive oil deep-fried at 170 °C for 3 h without polyphenol supplementation, Exp.2: olive oil deep-fried at 170 °C for 6 h without polyphenol supplementation, Exp.3: olive oil deep-fried at 210 °C for 3 h without polyphenol supplementation, Exp.4: olive oil deep-fried at 210 °C for 6 h without polyphenol supplementation, Exp.5: olive oil deep-fried at 170 °C for 3 h with polyphenol supplementation, Exp.6: olive oil deep-fried at 170 °C for 6 h with polyphenol supplementation, Exp.7: olive oil deep-fried at 210 °C for 3 h with polyphenol supplementation, Exp.8: olive oil deep-fried at 210 °C for 6 h with polyphenol supplementation. Moreover, for sunflower oil categories: Control refers to original, non-deep-fried oil. Exp.1: oil deep-fried at 170 °C for 3 h, Exp.2: oil deep-fried at 170 °C for 6 h, Exp.3: oil deep-fried at 210 °C for 3 h, Exp.4: oil deep-fried at 210 °C for 6 h.

Furthermore, the principal component analysis (PCA) findings presented in Figure 14 indicate that most EVOOs, except Hojiblanca, along with Olive oil 1°, Pomace olive oil, and Olive oil 0.4°, are clustered around higher antioxidant potential with low degradation, particularly at lower temperatures and shorter frying times, especially with added HTyr. Conversely, most olive oil samples from Experiment 5 (fried at 170 °C for 3 h with HTyr) exhibited significantly lower degradation. In addition, sunflower oils are clustered around a higher refractive index, indicating relatively low stability.

Overall, EVOO varieties exhibited lower oxidation and thermal degradation compared to refined olive oils, particularly when deep-fried at 170 °C for 3 h with added HTyr. Notably, Manzanilla, Royuela, Koroneiki, and Arbosana oils showed excellent physicochemical stability under these conditions. Therefore, EVOOs, especially those supplemented with HTyr, demonstrated superior antioxidant properties and stability during deep-frying compared to refined olive oils and refined sunflower oils.

## 5. The Limitation and Commercial Implications

This study highlights that supplementing various olive oil categories (e.g., nine EVOO varieties, EVOO mixed with ROO, refined Pomace olive oil mixed with EVOO, and VOO mixed with ROO) with HTyr enhances thermal stability and antioxidant potential, making it a promising natural stabilizer for high-temperature processing. This has significant commercial implications for olive oil producers, offering an alternative to synthetic antioxidants while extending shelf life and improving oil quality. The findings also support EVOO’s compliance with EFSA health claims, reinforcing its market value as a premium, health-promoting product. Encouraging HTyr enrichment could help producers differentiate their products and appeal to consumers seeking healthier, high-performance cooking/and or frying edible oils.

To reproduce these findings across diverse conditions, we recommend further studies using different EVOO cultivars from various countries. This would provide broader insights into regional and genetic influences on oil stability, antioxidant properties, and overall quality under deep-frying conditions.

Commercially, these findings demonstrate HTyr’s potential to enhance frying oil stability and nutritional quality, positioning it as a natural alternative to synthetic antioxidants. The improved oxidative resistance of HTyr-supplemented EVOO extends shelf life and usability in the food industry, adding value to EVOO, promoting its adoption in commercial frying operations, and aligning with consumer demand for healthier cooking oils. Additionally, HTyr-enriched olive oil not only adds value for producers but also benefits consumers by providing food products enriched with bioactive compounds that offer various health benefits. This is particularly relevant for non-fried olive oil supplemented with HTyr, which retains its antioxidant properties. Furthermore, HTyr-enriched olive oil holds potential for commercial applications in the food supplement industry, catering to the growing demand for functional and health-promoting products.

## 6. Conclusions

This study examined the physicochemical changes in various varieties of extra virgin olive oils (EVOOs) supplemented with exogenous polyphenolic extract, particularly olive fruit extract (OFE) enriched with hydroxytyrosol (HTyr). The results were compared with several refined olive oils, sunflower oil, and high-oleic sunflower oil under different deep-frying conditions (170–210 °C for 3 to 6 h, with or without added HTyr. The findings revealed that EVOO varieties generally exhibited lower acidity and less thermal degradation compared to refined olive oils, especially when deep-fried at 170 °C for 3 h with added HTyr. EVOO varieties such as Royuela, Koroneiki, Empeltre, Manzanilla, and Arbosana showed lower rancidity values, indicating better stability against oxidation. Additionally, the Koroneiki and Arbosana varieties had the highest carotenoid and chlorophyll levels. The current study also found that all EVOO varieties, even those of lower quality when supplemented with OFE, exhibited strong radical scavenging activity toward DPPH. Therefore, deep-fried EVOOs enriched with HTyr at 170 °C for 3 h demonstrated exceptional thermal stability, showing low hydrolysis, low oxidation, enhanced antioxidant activity, and elevated levels of chlorophyll and carotenoids. These conditions were selected as the best for deep-frying most EVOO varieties. In conclusion, the addition of HTyr extract to EVOO during deep-frying is of significant interest, both for its potent health benefits and as a natural antioxidant. The addition of HTyr to deep-frying oils not only enhances the health benefits of EVOO, supporting EFSA health claims, but also acts as a promising stabilizer for the olive oil industry, particularly under high-temperature processing conditions over prolonged periods. This highlights its potential for industrial use as a natural alternative to synthetic antioxidants, not only for olive oil but also for other edible oils, with practical applications in the food industry to improve the quality and stability of cooking and/or frying oils.

## Figures and Tables

**Table 1:** An illustration of the best deep-frying conditions of the physicochemical indices and oxidation markers of numerous olive oil classes along with sunflower oil and high oleic sunflower oil *.

Vegetable Oil	Acidity	K_232_	K_270_	∆K	PV	AnV	TOTOX	RI
EVOO Picual	0.32 ± 0.01 ^d^	2.50 ± 0.06 ^d^	0.84 ± 0.02 ^d^	0.05 ± 0.02 ^d^	9.51 ± 0.13 ^c^	17.76 ± 0.39 ^d^	36.77 ± 0.51 ^e^	1.4684 ± 0.0001 ^d^
EVOO Cornicabra	0.38 ± 0.02 ^c^	2.64 ± 0.09 ^cd^	1.12 ± 0.00 ^c^	0.07 ± 0.00 ^c^	11.52 ± 0.37 ^b^	17.54 ± 0.21^d^	40.73 ± 0.64 ^e^	1.4683 ± 0.0001 ^d^
EVOO Empeltre	0.33 ± 0.01 ^d^	1.79 ± 0.24 ^f^	0.35 ± 0.00 ^f^	0.03 ± 0.00 ^e^	9.71 ± 0.19 ^c^	7.76 ± 0.36 ^e^	27.06 ± 0.56 ^f^	1.4684 ± 0.0001 ^d^
EVOO Arbequina	0.34 ± 0.01 ^d^	2.21 ± 0.18 ^e^	0.69 ± 0.01 ^e^	0.05 ± 0.01 ^d^	9.36 ± 0.53 ^c^	9.00 ± 0.13 ^e^	27.73 ± 0.97 ^f^	1.4684 ± 0.0001 ^d^
EVOO Hojiblanca	0.56 ± 0.02 ^b^	2.31 ± 0.04 ^e^	0.68 ± 0.01 ^e^	0.01 ± 0.00 ^f^	19.83 ± 0.70 ^a^	26.18 ± 0.15 ^c^	65.84 ± 1.59 ^c^	1.4691 ± 0.0001^c^
EVOO Manzanilla	0.22 ± 0.00 ^f^	1.81 ± 0.06 ^f^	0.22 ± 0.00 ^h^	0.00 ± 0.00 ^g^	9.41 ± 0.24 ^c^	4.86 ± 0.33 ^fg^	23.85 ± 0.50 ^g^	1.4683 ± 0.0001 ^d^
EVOO Royuela	0.27 ± 0.01 ^e^	1.36 ± 0.09 ^g^	0.11 ± 0.00 ^i^	0.00 ± 0.00 ^g^	9.86 ± 0.20 ^c^	1.03 ± 0.28 ^h^	20.76 ± 0.16 ^g^	1.4683 ± 0.0001 ^d^
Pomace olive oil	0.33 ± 0.01 ^d^	3.03 ± 0.07 ^bc^	1.36 ± 0.03 ^b^	0.13 ± 0.03 ^b^	11.67 ± 0.51 ^b^	38.36 ± 0.26 ^b^	61.28 ± 1.13 ^c^	1.4690 ± 0.0001 ^c^
EVOO Koroneiki	0.27 ± 0.02 ^e^	1.67 ± 0.10 ^f^	0.25 ± 0.01 ^h^	0.01 ± 0.00 ^f^	9.87 ± 0.19 ^a^	3.63 ± 0.41 ^fg^	23.38 ± 0.70 ^g^	1.4682 ± 0.0001 ^de^
EVOO Arbosana	0.28 ± 0.01 ^e^	1.82 ± 0.09 ^f^	0.31 ± 0.00 ^g^	0.01 ± 0.00 ^f^	11.40 ± 0.41 ^b^	5.46 ± 0.29 ^f^	28.27 ± 1.05 ^f^	1.4679 ± 0.0001 ^f^
Olive oil 1°	0.62 ± 0.01 ^a^	2.83 ± 0.13 ^c^	0.74 ± 0.00 ^e^	0.03 ± 0.00 ^e^	21.59 ± 0.41 ^a^	29.24 ± 0.28 ^c^	72.43 ± 0.69 ^b^	1.4682 ± 0.0000 ^de^
Olive oil 0.4°	0.28 ± 0.01 ^e^	2.36 ± 0.24 ^de^	0.83 ± 0.01 ^d^	0.07 ± 0.01 ^c^	9.87 ± 0.14 ^c^	20.34 ± 0.04 ^d^	40.07 ± 0.41 ^e^	1.4676 ± 0.0000 ^fg^
Sunflower oil	0.28 ± 0.01 ^e^	4.41 ± 0.02 ^a^	2.55 ± 0.01 ^a^	0.39 ± 0.01 ^a^	13.85 ± 0.20 ^b^	52.04 ± 0.01 ^a^	79.59 ± 0.48 ^a^	1.4732 ± 0.0000 ^a^
Sunflower oil—high oleic acid	0.17 ± 0.01 ^g^	3.25 ± 0.09 ^b^	1.21 ± 0.00 ^c^	0.14 ± 0.00 ^b^	5.95 ± 0.23 ^d^	40.99 ± 0.82 ^b^	52.50 ± 0.65 ^d^	1.4709 ± 0.0001 ^b^

* Experiment 5 was identified as the superior stable treatment for numerous olive oil types, whereas Experiment 1 was deemed the best for sunflower oils. ^a,b,c,d,e,f,^ and ^g^: Data in the same column followed by different small letters vary significantly (*p* < 0.05).

**Table 2:** An illustration of the best deep-frying conditions of the physicochemical indices and oxidation markers of several olive oil classes along with sunflower oil and high oleic sunflower oil *.

Vegetable Oil	Carotenoids	Chlorophyll	Antioxidant Activity % (DPPH Scavenging Activity)
EVOO Picual	9.3 ± 0.0 ^d^	31.4 ± 0.7 ^b^	87.0 ± 2.2 ^ab^
EVOO Cornicabra	8.1 ± 0.2 ^e^	30.3 ± 0.7 ^b^	84.3 ± 2.8 ^b^
EVOO Empeltre	9.9 ± 0.2 ^cd^	30.8 ± 0.5 ^b^	86.3 ± 1.0 ^ab^
EVOO Arbequina	10.1 ± 0.0 ^c^	29.6 ± 0.9 ^b^	86.1 ± 0.3 ^ab^
EVOO Hojiblanca	6.4 ± 1.2 ^f^	29.8 ± 1.3 ^b^	87.6 ± 1.7 ^ab^
EVOO Manzanilla	8.0 ± 0.5 ^e^	31.1 ± 1.5 ^b^	87.6 ± 0.2 ^ab^
EVOO Royuela	10.9 ± 0.1 ^c^	31.8 ± 0.7 ^b^	86.7 ± 1.7 ^ab^
Pomace olive oil	8.8 ± 0.5 ^de^	21.5 ± 0.5 ^c^	83.3 ± 1.0 ^b^
EVOO Koroneiki	13.7 ± 0.4 ^b^	50.2 ± 2.1 ^a^	86.8 ± 0.9 ^ab^
EVOO Arbosana	17.5 ± 0.5 ^a^	53.7 ± 0.7 ^a^	90.8 ± 1.0 ^a^
Olive oil 1°	10.9 ± 0.2 ^c^	47.5 ± 1.1 ^a^	82.7 ± 1.9 ^b^
Olive oil 0.4°	9.7 ± 0.2 ^d^	21.9 ± 0.4 ^c^	83.8 ± 1.1 ^b^
Sunflower oil	6.6 ± 0.4 ^f^	15.7 ± 0.1 ^d^	26.0 ± 0.3 ^c^
Sunflower oil—high oleic acid	8.7 ± 0.0 ^e^	21.3 ± 0.2 ^c^	26.6 ± 1.1 ^c^

* Experiment 5 was identified as the superior stable treatment for numerous olive oil types, whereas Experiment 1 was deemed the best for sunflower oils. ^a,b,c,d,e^ and ^f^: Data in the same column followed by different small letters vary significantly (*p* < 0.05).

## Data Availability

Data will be available upon reasonable request.

## Supplementary Materials

The following supporting information can be downloaded at: https://www.mdpi.com/article/10.3390/antiox14030368/s1, Table S1. Experimental design (2^3^) methodology of olive oils supplemented with hydroxytyrosol under deep-frying stress. This experiment was conducted separately for each olive oil category, comprising nine EVOOs and three lower-quality olive oils (i.e., Pomace olive oil, Olive oil 1°, and Olive oil 0.4°). Table S2. Experimental design (2^2^) methodology for sunflower oil and high oleic sunflower oil under deep-frying stress. Table S3. Total phenolic content (TPC) of various olive oils before thermal processing, as used in the experimental design for the deep-frying process. Figures S1–S132: Each figure illustrates the results showing the effect of the independent variables—time and temperature, time and polyphenols, and temperature and polyphenols—on physicochemical indicators, e.g., acidity, K_232_, K_270_, ∆K, peroxide value (PV), anisidine value (AnV), TOTOX, refractive index (RI), carotenoids, chlorophyll, and antioxidant activity. The results highlight the significant impact of each independent variable, and the interactions between the combined variables on physicochemical indicators are also highlighted. Each corresponding title for each Figure was provided in detailed captions in the Supplementary Materials file of this article.
