# Supplementary material for: Hydroxytyrosol-Infused Extra Virgin Olive Oil: A Key to Minimizing Oxidation, Boosting Antioxidant Potential, and Enhancing Physicochemical Stability During Frying
Source: Antioxidants (Basel). 2025 Mar 20;14(3):368. doi: 10.3390/antiox14030368 (PMC11939150; doi:10.3390/antiox14030368)
Supplement: Supplementary file 1 [file antioxidants-14-00368-s001.zip › antioxidants-3496684-supplementary.pdf]

<u>Supplementary materials for the article:</u>

Hydroxytyrosol-Infused Extra Virgin Olive Oil: A Key to Minimizing Oxidation, Boosting Antioxidant Potential, and Enhancing Physicochemical Stability During Frying

Taha Mehany, José M. González-Sáiz and Consuelo Pizarro *

Department of Chemistry, University of La Rioja, 26006 Logroño, Spain; taha.abdellatif@unirioja.es (T.M.); josemaria.gonzalez@unirioja.es (J.M.G.-S.)

* Correspondence: consuelo.pizarro@unirioja.es; Tel.: +34-941299626

Table S1. Experimental design (2^3) methodology of olive oils supplemented with hydroxytyrosol under deep-frying stress. The experiment was conducted separately for each olive oil category, comprising nine EVOOs and three lower-quality olive oils (i.e., Pomace olive oil, Olive oil 1°, and Olive oil 0.4°).

**Experiment**	**Design Matrix**			**Independent Variables**			**Response**
	**X_1**	**X_2**	**X_3**	**Time (h)**	**Temp. (°C)**	**Polyphenols (mg/kg)**	
1	-1	-1	-1	3	170	-	Acidity, K_{232}, K_{270}, ΔK, peroxide value, anisidine value, TOTOX, refractive index, carotenoids, chlorophyll, and antioxidant activity
2	+1	-1	-1	6	170	-	
3	-1	+1	-1	3	210	-	
4	+1	+1	-1	6	210	-	
5	-1	-1	+1	3	170	650	
6	+1	-1	+1	6	170	650	
7	-1	+1	+1	3	210	650	
8	+1	+1	+1	6	210	650	

Level	Time (h)	Temp. (°C)	Polyphenols (mg/kg)
-1	3	170	Original concentration (0 addition)
+1	6	210	650

Table S2. Experimental design (2^2) Methodology for sunflower oil and sunflower oil-high oleic acid under deep-frying stress.

Experiment	X_1	X_2	Independent Variables		Response
			Time (h)	Temp.(°C)	
1	-1	-1	3	170	Acidity, K_{232}, K_{270}, ΔK, peroxide value, anisidine value, TOTOX, refractive index, carotenoids, chlorophyll, and antioxidant activity
2	+1	-1	6	170	
3	-1	+1	3	210	
4	+1	+1	6	210	

Level	Time (h)	Temp. (°C)
-1	3	170
+1	6	210

Table S3. Total phenolic content (TPC) by determined HPLC of various olive oils before thermal processing, as used in the experimental design for the deep-frying process.

Olive Oil Type	Original Olive Oil (Control 1)	Supplemented Olive Oil	Supplemented Olive Oil and Original Olive Oil Mix (Control 2)
Picual	307.9 ± 8.7	1524.3 ± 2.4	658.6 ± 4.4
Cornicabra	275.6 ± 9.3	1683.0 ± 32.0	658.1 ± 17.4
Empeltre	337.9 ± 11.5	1468.8 ± 47.3	647.2 ± 5.2
Arbequina	227.7 ± 6.3	1384.9 ± 31.4	663.9 ± 6.5
Hojiblanca	209.5 ± 9.2	1208.6 ± 41.6	655.7 ± 13.1
Manzanilla	309.0 ± 16.7	1255.2 ± 28.1	661.2 ± 7.7
Royuela	400.6 ± 10.1	1211.1 ± 30.8	662.3 ± 10.8
Pomace olive oil	3.9 ± 0.3	1259.0 ± 20.4	652.2 ± 5.5
Koroneiki	327.7 ± 12.1	1230.9 ± 18.2	663.9 ± 3.1
Arbosana	393.0 ± 6.6	1434.5 ± 27.8	666.9 ± 16.5
Olive oil 1°	181.8 ± 7.2	1152.2 ± 45.1	653.1 ± 17.6
Olive oil 0.4°	26.5 ± 2.4	1113.4 ± 14.7	650.8 ± 13.1
Sunflower oil *	0	-	-
Sunflower oil-high oleic acid *	0	-	-

*: non-applicable for the supplementation process with polyphenols.

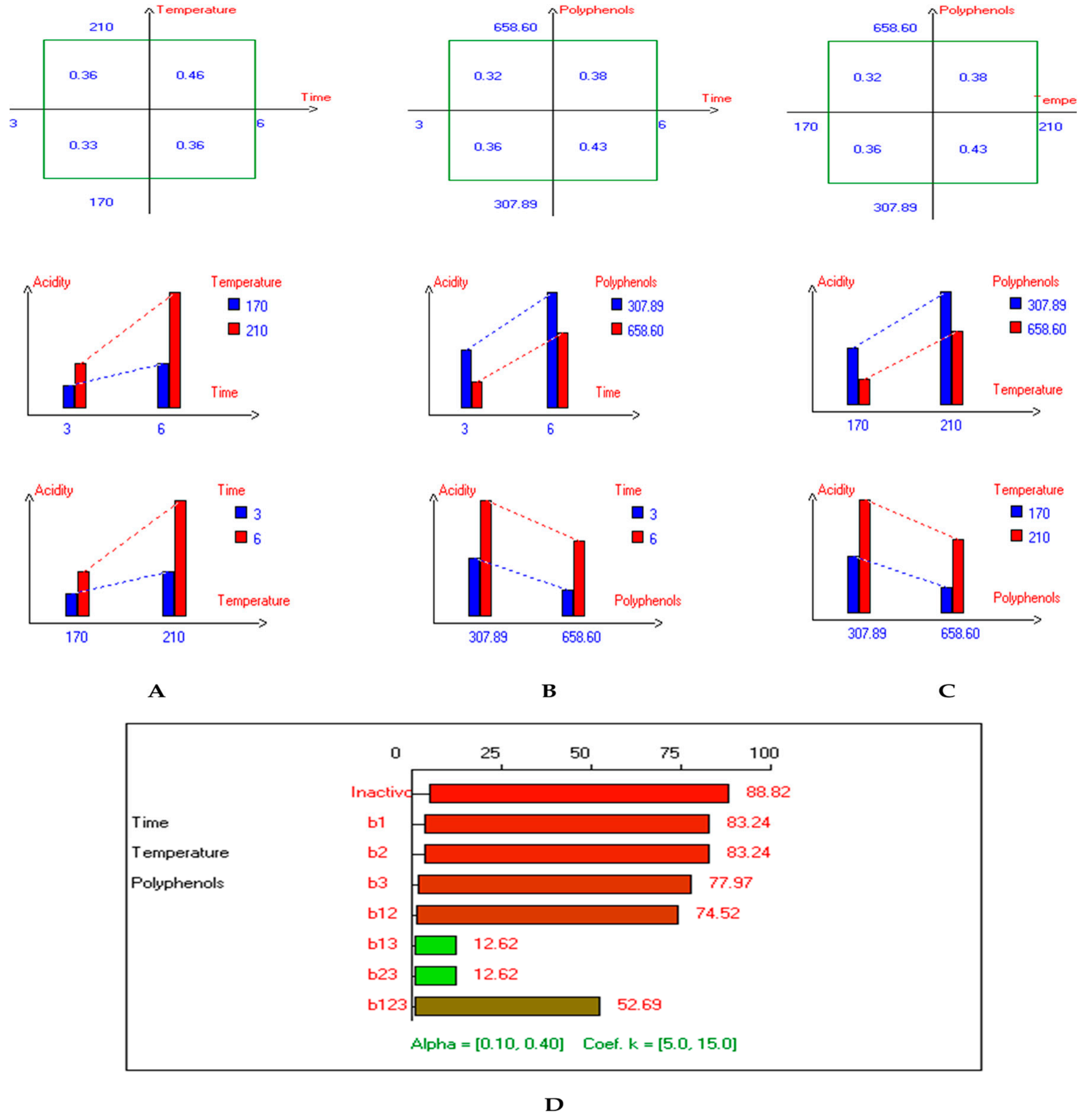

Figure S1. Combined interactions between the independent variables on a response variable (**acidity%**) in EVOO **Picual** under D-F: (**A**) x_1 and x_2, (**B**) x_1 and x_3, (**C**) x_2 and x_3, and (**D**) the results of the variance analysis of the regression equation model and the significance of changes in each individual independent variable, as well as the interactions between the combined independent variables on acidity; b represents a significant difference when be $>b_{123}$, while b represents no significant difference when be $\leq b_{123}$; b_1, b_2, b_3 are the main effects of the independent variables, while b_{12}, b_{13}, b_{23}, and b_{123} are the interaction effects of the independent variables. Moreover, x_1, x_2, and x_3 are coded variables (time, temperature, and polyphenols addition, respectively) for the experimental design in D-F process.

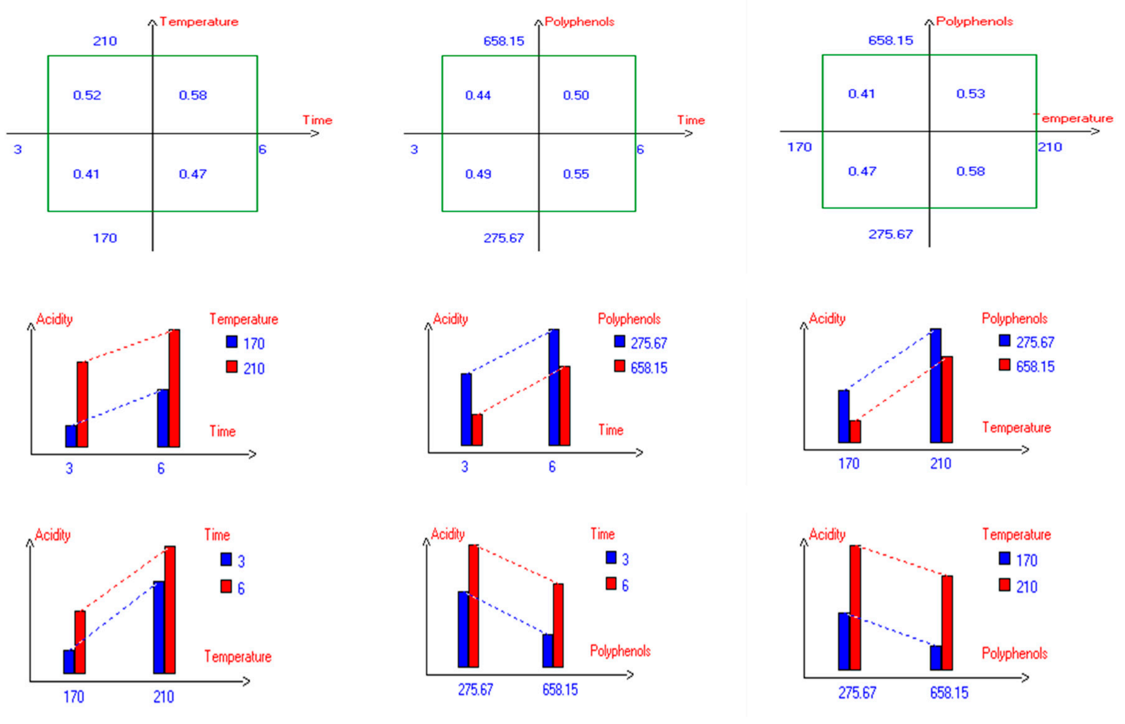

A **B** **C**

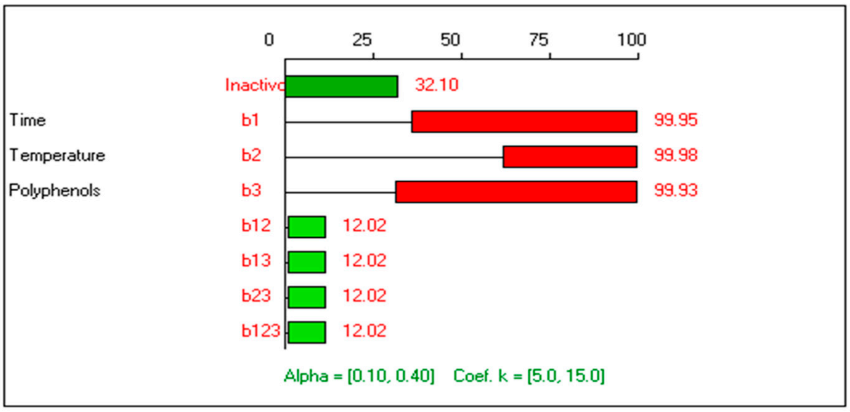

D

Figure S2. Combined interactions between the independent variables on a response variable (**acidity%**) in EVOO **Cornicabra** under D-F: (**A**) x_1 and x_2, (**B**) x_1 and x_3, (**C**) x_2 and x_3, and (**D**) results of variance analysis of regression equation model and the significance changes in each individual independent variable and interaction between the combined independent variables on acidity; b represents a significant difference when be $>b_{123}$, while b represents no significant difference when be $\leq b_{123}$; b_1, b_2, b_3 are the main effects of the independent variables, while b_{12}, b_{13}, b_{23}, and b_{123} are the interaction effects of the independent variables. Moreover, x_1, x_2, and x_3 are coded variables (time, temperature, and polyphenols addition, respectively) for the experimental design in D-F process.

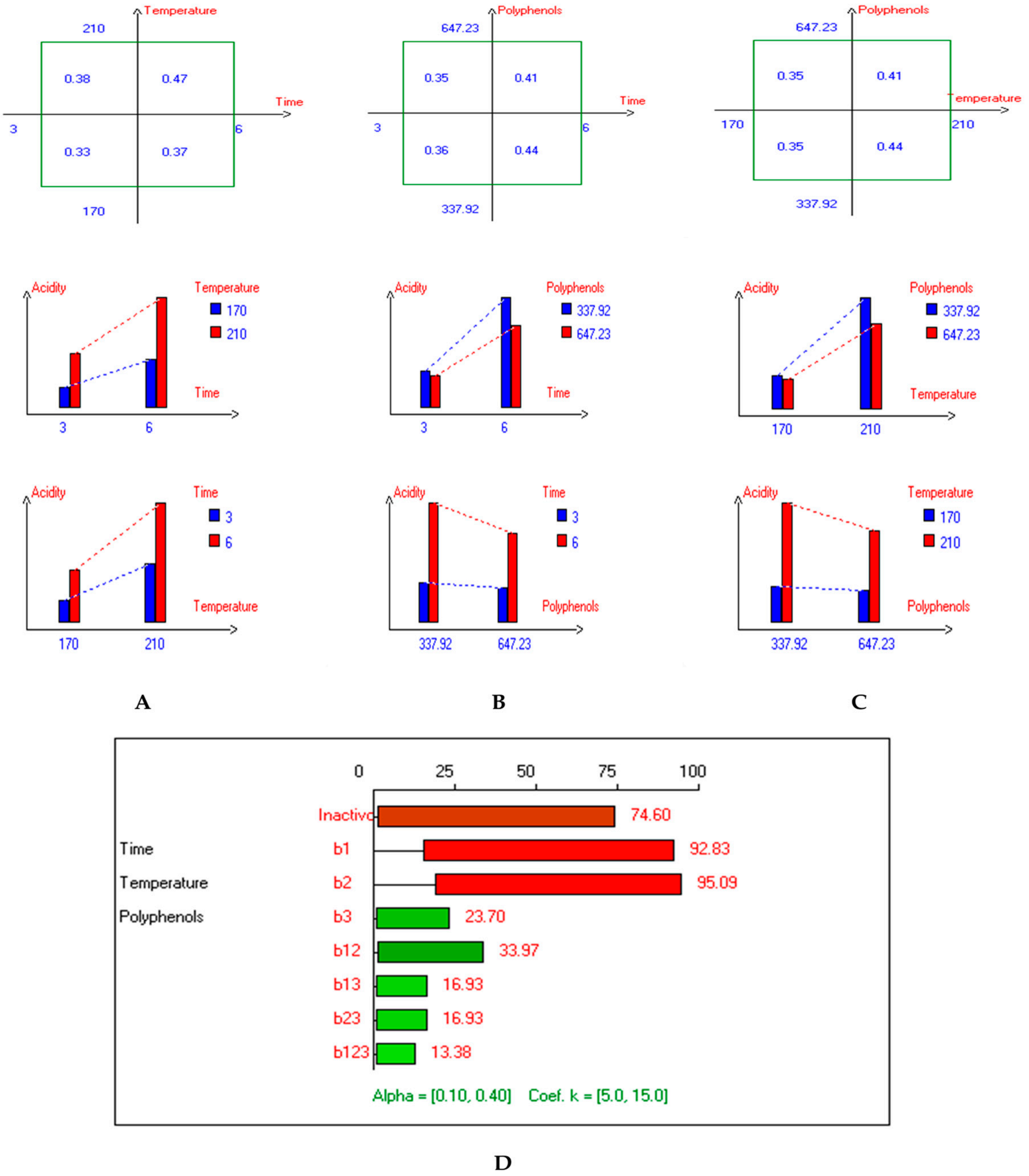

Figure S3. Combined interactions between the independent variables on a response variable (**acidity%**) in EVOO **Empeltre** under D-F: (**A**) x_1 and x_2, (**B**) x_1 and x_3, (**C**) x_2 and x_3, and (**D**) results of variance analysis of regression equation model and the significance changes in each individual independent variable and interaction between the combined independent variables on acidity; b represents a significant difference when be $>b_{123}$, while b represents no significant difference when be $\leq b_{123}$; b_1, b_2, b_3 are the main effects of the independent variables, while b_{12}, b_{13}, b_{23}, and b_{123} are the interaction effects of the independent variables. Moreover, x_1, x_2, and x_3 are coded variables (time, temperature, and polyphenols addition, respectively) for the experimental design in D-F process.

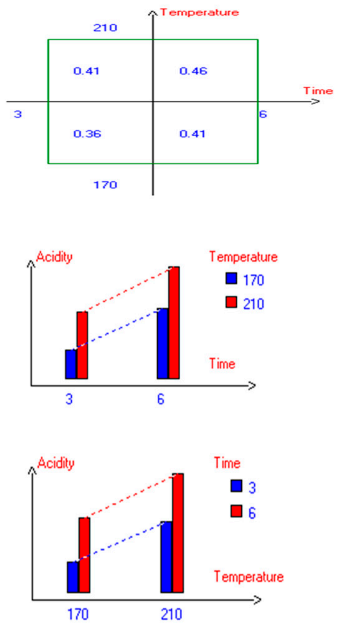

A

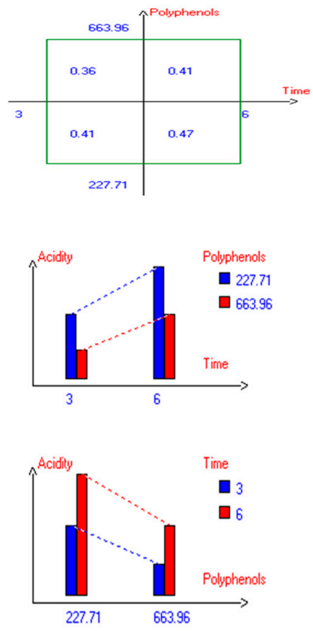

B

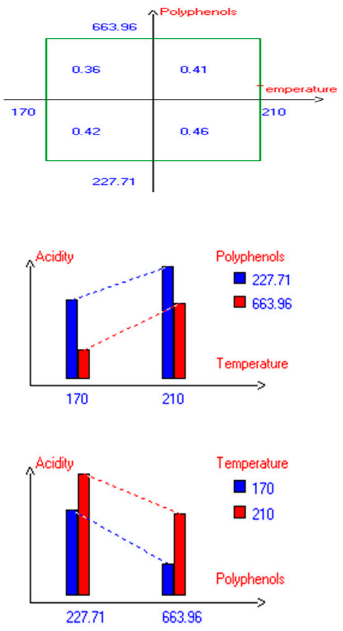

C

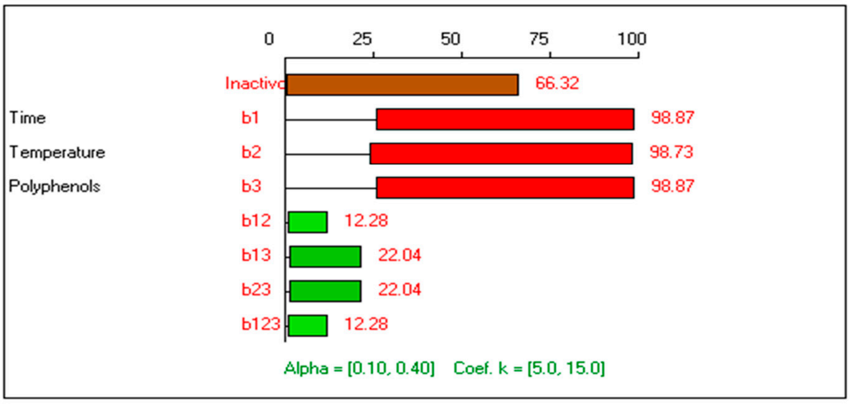

D

Figure S4. Combined interactions between the independent variables on a response variable (**acidity%**) in EVOO **Arbequina** under D-F: (**A**) x_1 and x_2, (**B**) x_1 and x_3, (**C**) x_2 and x_3, and (**D**) results of variance analysis of regression equation model and the significance changes in each individual independent variable and interaction between the combined independent variables on acidity; b represents a significant difference when be $>b_{123}$, while b represents no significant difference when be $\leq b_{123}$; b_1, b_2, b_3 are the main effects of the independent variables, while b_{12}, b_{13}, b_{23}, and b_{123} are the interaction effects of the independent variables. Moreover, x_1, x_2, and x_3 are coded variables (time, temperature, and polyphenols addition, respectively) for the experimental design in D-F process.

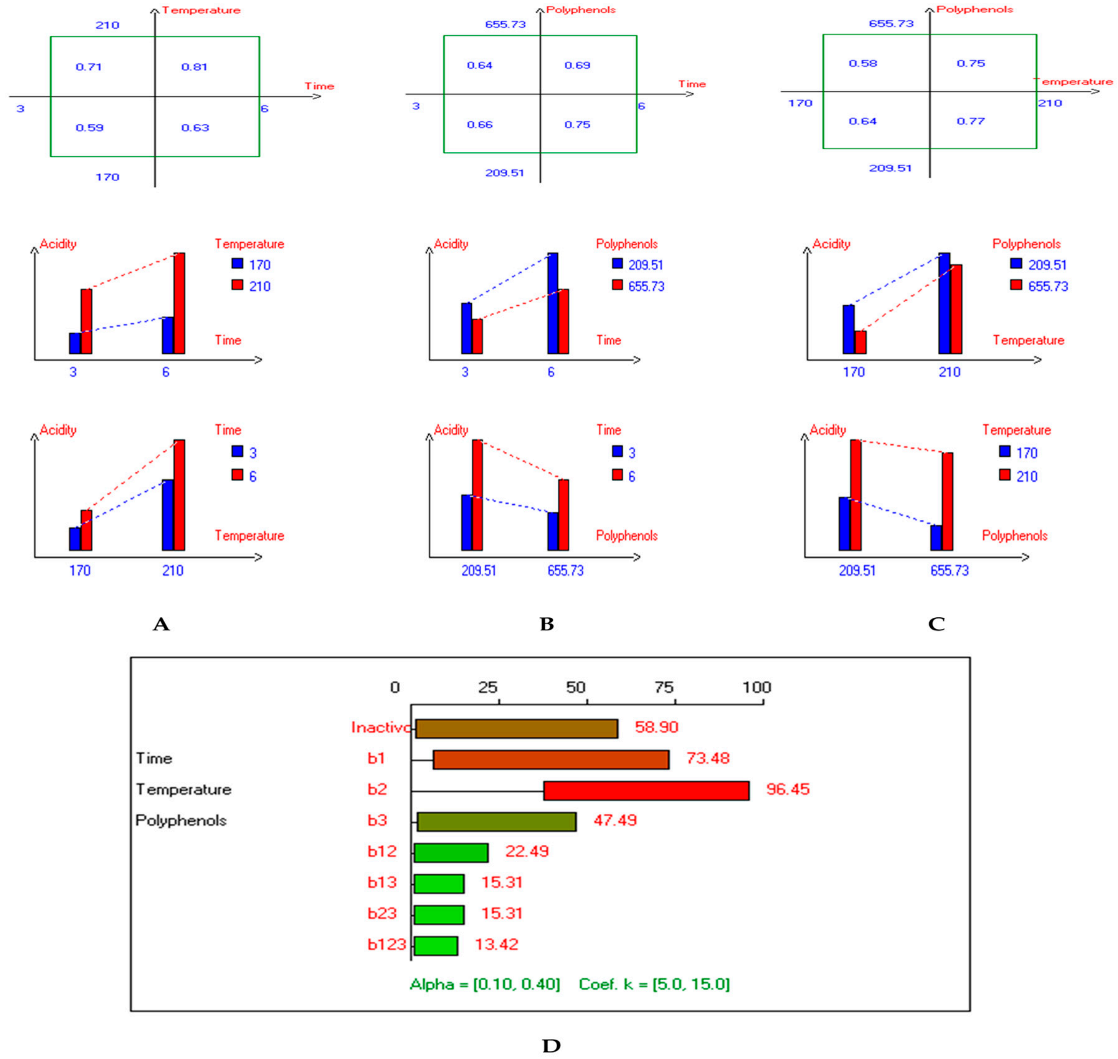

Figure S5. Combined interactions between the independent variables on a response variable (**acidity%**) in EVOO **Hojiblanca** under D-F: (**A**) x_1 and x_2, (**B**) x_1 and x_3, (**C**) x_2 and x_3, and (**D**) results of variance analysis of regression equation model and the significance changes in each individual independent variable and interaction between the combined independent variables on acidity; b represents a significant difference when be $>b_{123}$, while b represents no significant difference when be $\leq b_{123}$; b_1, b_2, b_3 are the main effects of the independent variables, while b_{12}, b_{13}, b_{23}, and b_{123} are the interaction effects of the independent variables. Moreover, x_1, x_2, and x_3 are coded variables (time, temperature, and polyphenols addition, respectively) for the experimental design in D-F process.

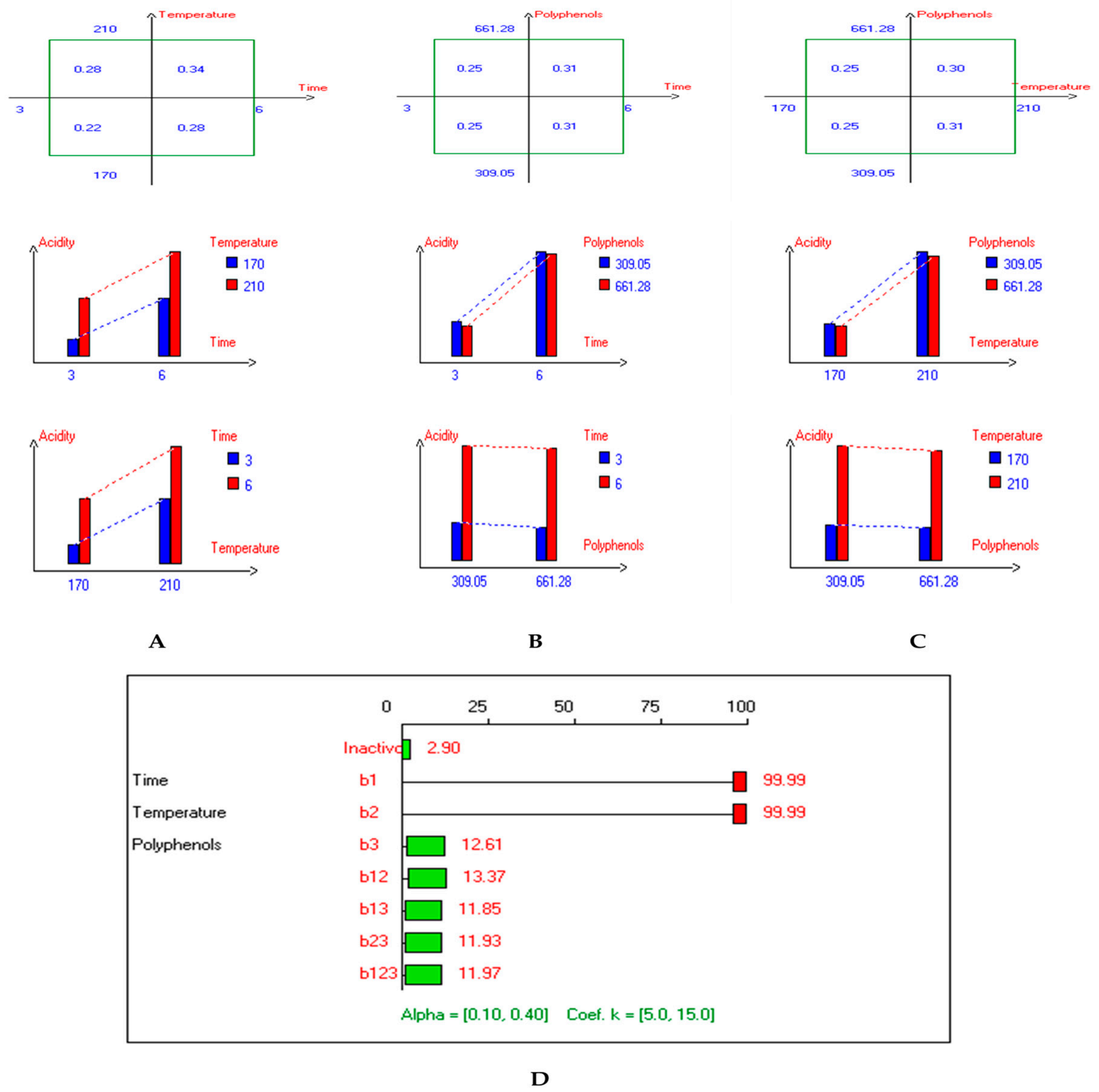

Figure S6. Combined interactions between the independent variables on a response variable (**acidity%**) in EVOO **Manzanilla** under D-F: (**A**) x_1 and x_2, (**B**) x_1 and x_3, (**C**) x_2 and x_3, and (**D**) results of variance analysis of regression equation model and the significance changes in each individual independent variable and interaction between the combined independent variables on acidity; b represents a significant difference when be $>b_{123}$, while b represents no significant difference when be $\leq b_{123}$; b_1, b_2, b_3 are the main effects of the independent variables, while b_{12}, b_{13}, b_{23}, and b_{123} are the interaction effects of the independent variables. Moreover, x_1, x_2, and x_3 are coded variables (time, temperature, and polyphenols addition, respectively) for the experimental design in D-F process.

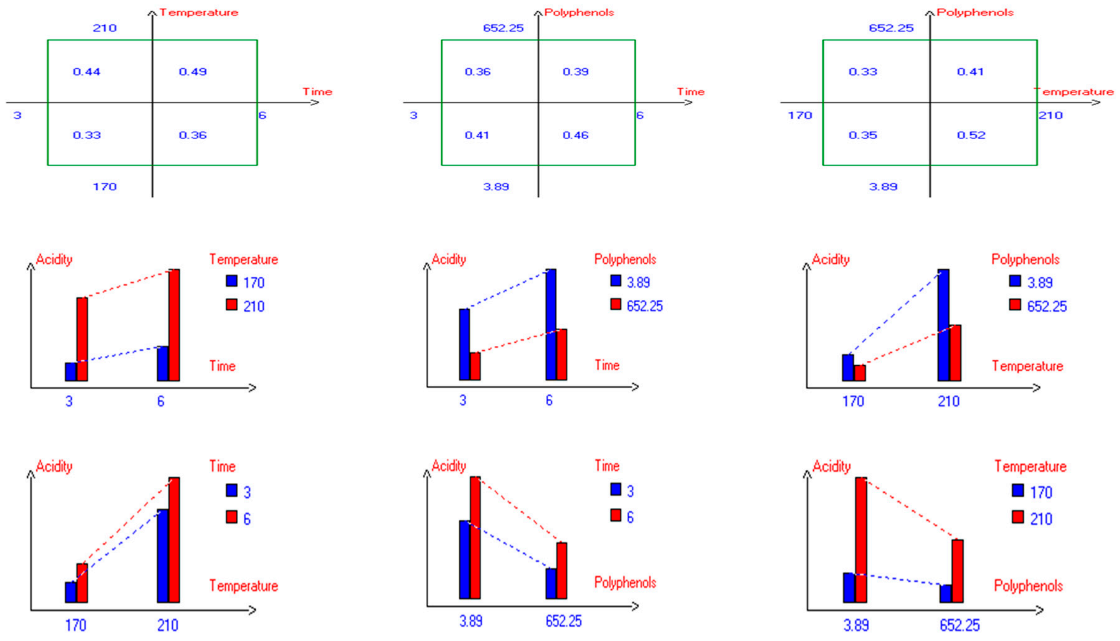

A **B** **C**

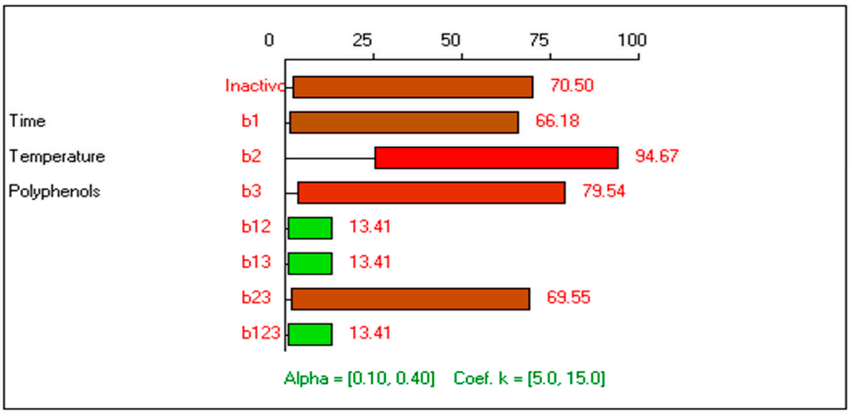

D

Figure S7. Combined interactions between the independent variables on a response variable (**acidity%**) in **Pomace** olive oil under D-F: (**A**) x_1 and x_2, (**B**) x_1 and x_3, (**C**) x_2 and x_3, and (**D**) results of variance analysis of regression equation model and the significance changes in each individual independent variable and interaction between the combined independent variables on acidity; b represents a significant difference when be $>b_{123}$, while b represents no significant difference when be $\leq b_{123}$; b_1, b_2, b_3 are the main effects of the independent variables, while b_{12}, b_{13}, b_{23}, and b_{123} are the interaction effects of the independent variables. Moreover, x_1, x_2, and x_3 are coded variables (time, temperature, and polyphenols addition, respectively) for the experimental design in D-F process.

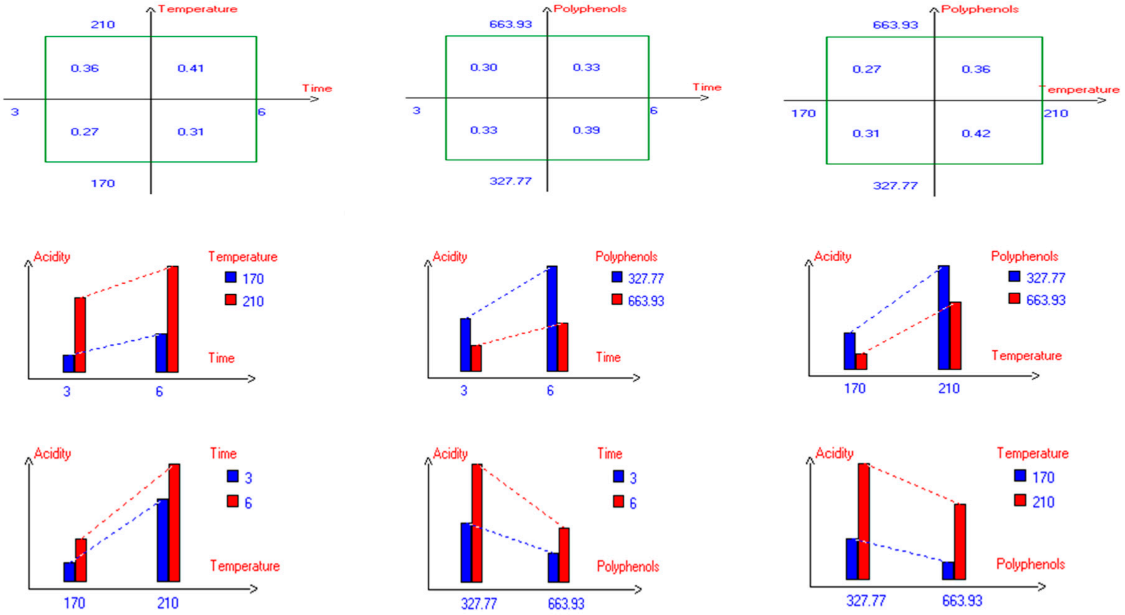

A **B** **C**

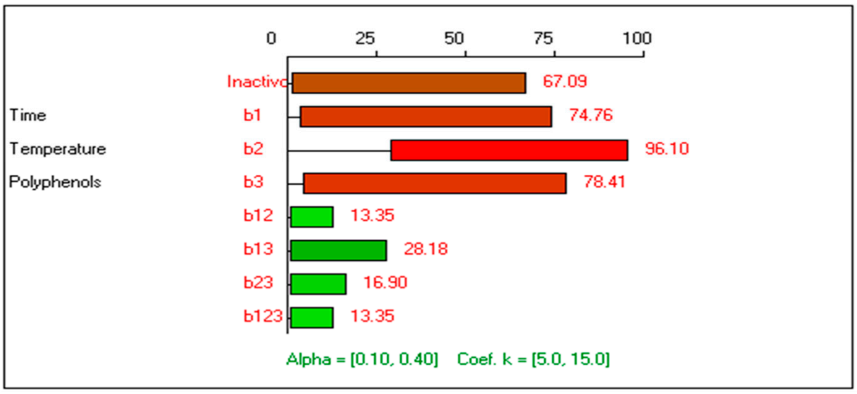

D

Figure S8. Combined interactions between the independent variables on a response variable (**acidity%**) in EVOO **Koroneiki** under D-F: (**A**) x_1 and x_2, (**B**) x_1 and x_3, (**C**) x_2 and x_3, and (**D**) results of variance analysis of regression equation model and the significance changes in each individual independent variable and interaction between the combined independent variables on acidity; b represents a significant difference when be >b_{123}, while b represents no significant difference when be ≤b_{123}; b_1, b_2, b_3 are the main effects of the independent variables, while b_{12}, b_{13}, b_{23}, and b_{123} are the interaction effects of the independent variables. Moreover, x_I, x_2, and x_3 are coded variables (time, temperature, and polyphenols addition, respectively) for the experimental design in D-F process.

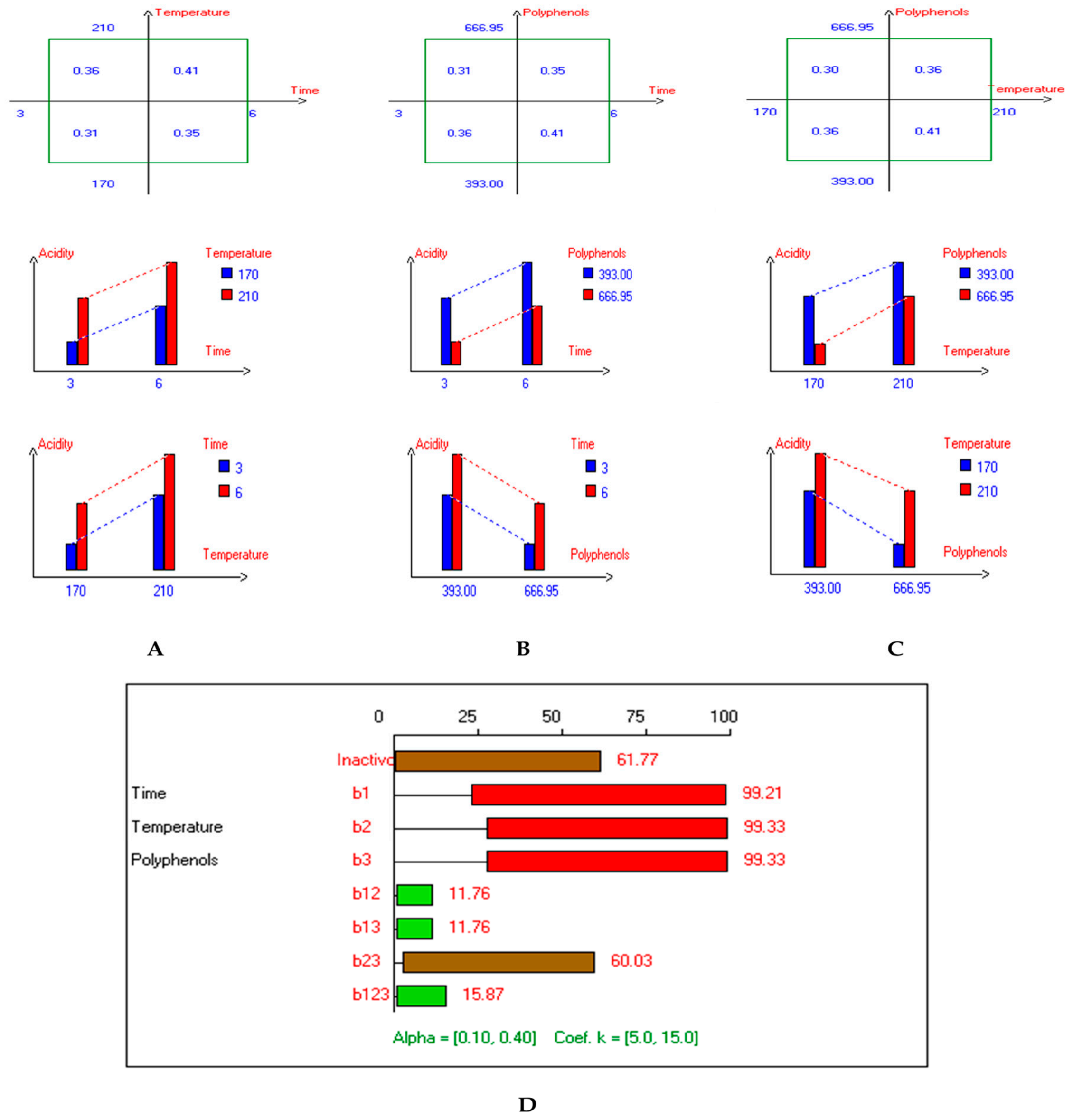

Figure S9. Combined interactions between the independent variables on a response variable (**acidity%**) in EVOO **Arbosana** under D-F: (**A**) x_1 and x_2, (**B**) x_1 and x_3, (**C**) x_2 and x_3, and (**D**) results of variance analysis of regression equation model and the significance changes in each individual independent variable and interaction between the combined independent variables on acidity; b represents a significant difference when be $>b_{123}$, while b represents no significant difference when be $\leq b_{123}$; b_1, b_2, b_3 are the main effects of the independent variables, while b_{12}, b_{13}, b_{23}, and b_{123} are the interaction effects of the independent variables. Moreover, x_1, x_2, and x_3 are coded variables (time, temperature, and polyphenols addition, respectively) for the experimental design in D-F process.

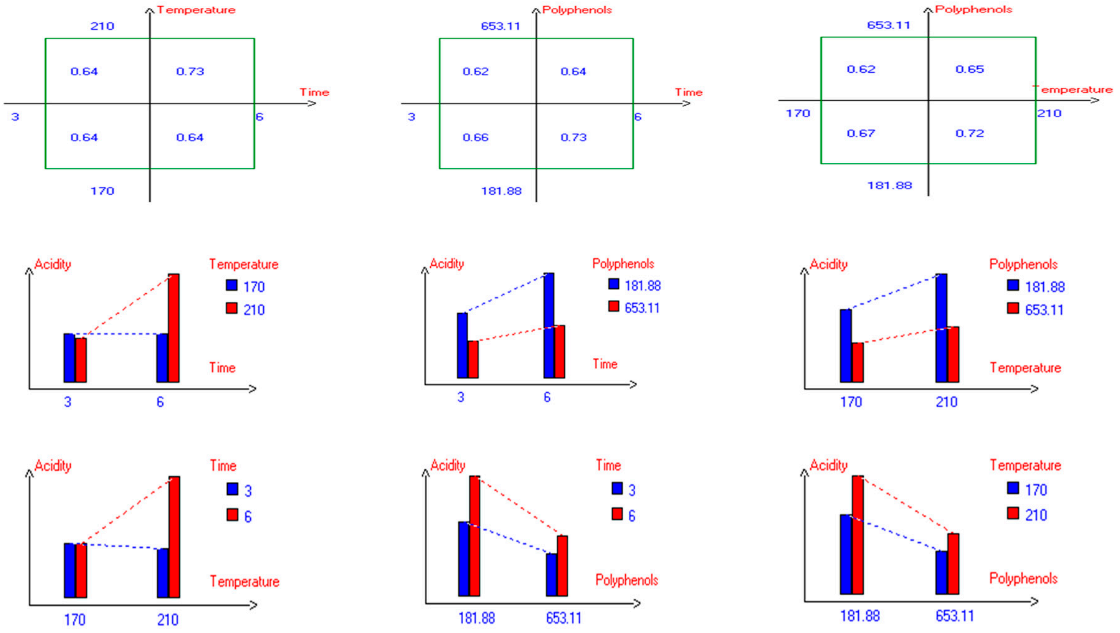

A B C

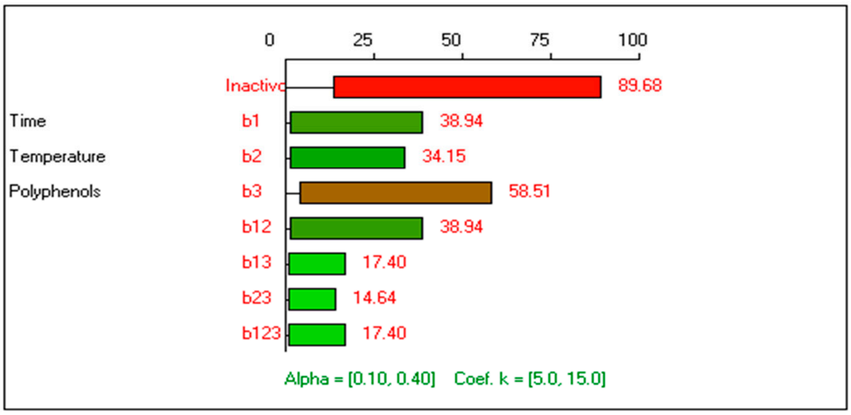

D

Figure S10. Combined interactions between the independent variables on a response variable (**acidity%**) in **Olive oil 1°** under D-F: (**A**) x_1 and x_2, (**B**) x_1 and x_3, (**C**) x_2 and x_3, and (**D**) results of variance analysis of regression equation model and the significance changes in each individual independent variable and interaction between the combined independent variables on acidity; b represents a significant difference when be $>b_{123}$, while b represents no significant difference when be $\leq b_{123}$; b_1, b_2, b_3 are the main effects of the independent variables, while b_{12}, b_{13}, b_{23}, and b_{123} are the interaction effects of the independent variables. Moreover, x_1, x_2, and x_3 are coded variables (time, temperature, and polyphenols addition, respectively) for the experimental design in D-F process.

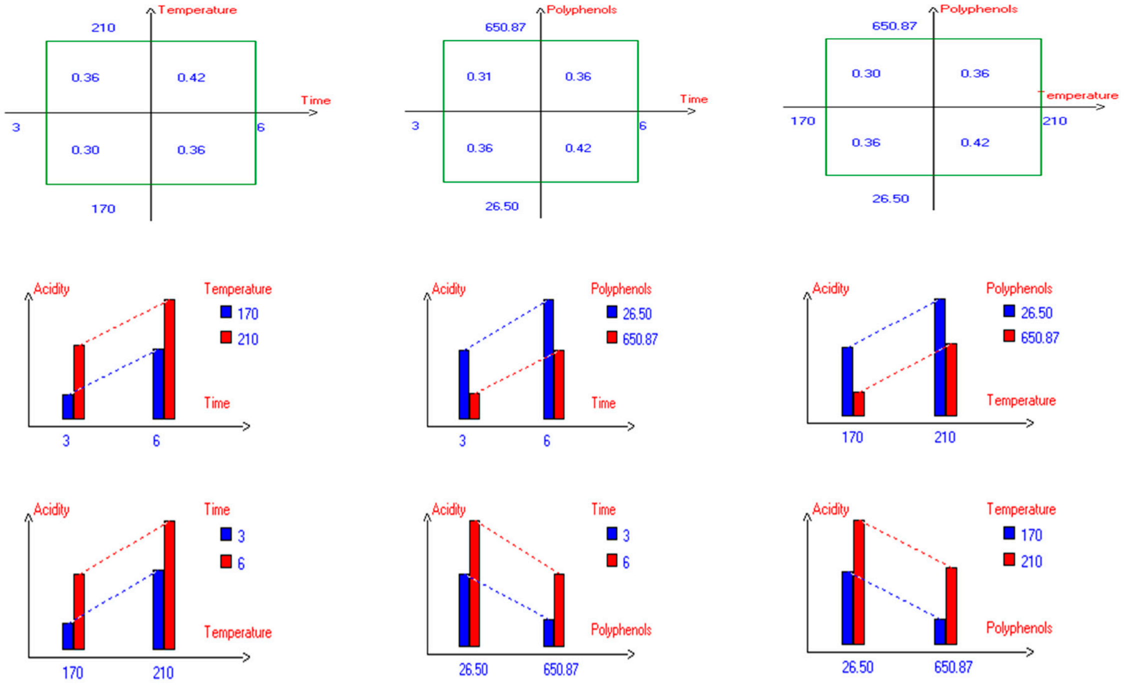

A B C

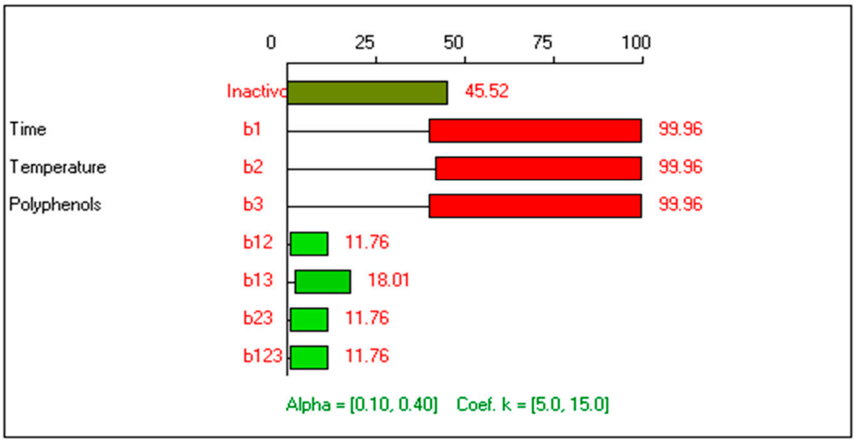

D

Figure S11. Combined interactions between the independent variables on a response variable (**acidity%**) in **Olive oil 0.4°** under D-F: (**A**) x_1 and x_2, (**B**) x_1 and x_3, (**C**) x_2 and x_3, and (**D**) results of variance analysis of regression equation model and the significance changes in each individual independent variable and interaction between the combined independent variables on acidity; b represents a significant difference when be $>b_{123}$, while b represents no significant difference when be $\leq b_{123}$; b_1, b_2, b_3 are the main effects of the independent variables, while b_{12}, b_{13}, b_{23}, and b_{123} are the interaction effects of the independent variables. Moreover, x_1, x_2, and x_3 are coded variables (time, temperature, and polyphenols addition, respectively) for the experimental design in D-F process.

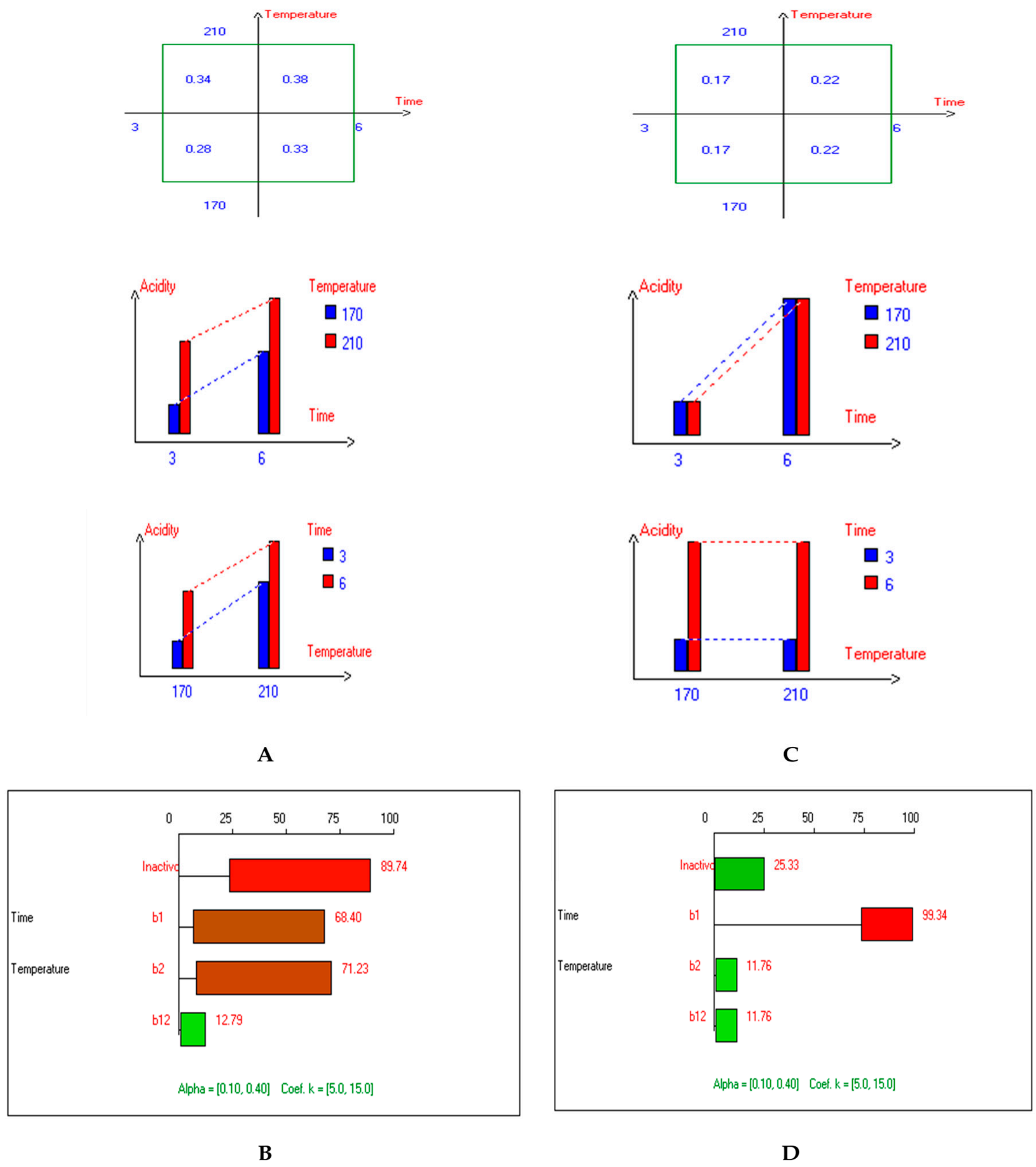

Figure S12. (A) Combined interactions between the independent variables (x_1 and x_2) on (**acidity%**) in **sunflower oil**, **(B)** Results of variance analysis of regression equation model and the significance changes of each individual independent variable and interaction between the combined independent variables on acidity in sunflower oil. **(C)** Combined interactions between the independent variables (x_1 and x_2) on (**acidity%**) in **sunflower oil-high oleic acid**, **(D)** Results of variance analysis of regression equation model and the significance changes of each individual independent variable and interaction between the combined independent variables on rancid score in sunflower oil-high oleic acid. Where, b represents significant difference when be $>b_{12}$; while b represents no significant difference when be $\leq b_{12}$. Moreover, b_1 and b_2 are the main effects of the independent variables, while b_{12} is the interaction effect of the independent variables. Additionally, x_1: time, x_2: temperature.

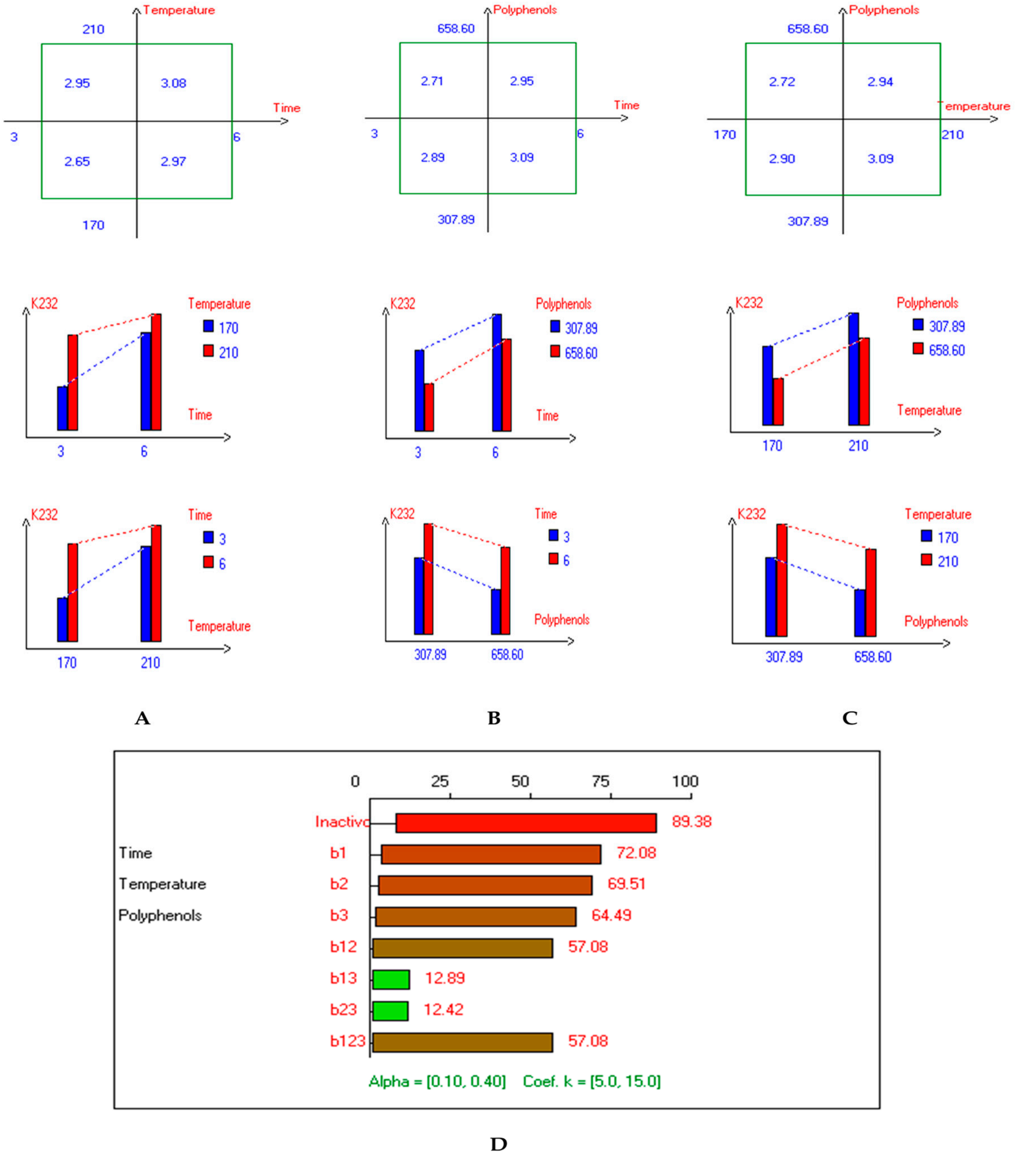

Figure S13. Combined interactions between the independent variables on a response variable (**K_{232}**) in EVOO **Picual** under D-F: (**A**) x_1 and x_2, (**B**) x_1 and x_3, (**C**) x_2 and x_3, and (**D**) results of variance analysis of regression equation model and the significance changes in each individual independent variable and interaction between the combined independent variables on K_{232}; b represents a significant difference when be $>b_{123}$, while b represents no significant difference when be $\leq b_{123}$; b_1, b_2, b_3 are the main effects of the independent variables, while b_{12}, b_{13}, b_{23}, and b_{123} are the interaction effects of the independent variables. Moreover, x_1, x_2, and x_3 are coded variables (time, temperature, and polyphenols addition, respectively) for the experimental design in D-F process.

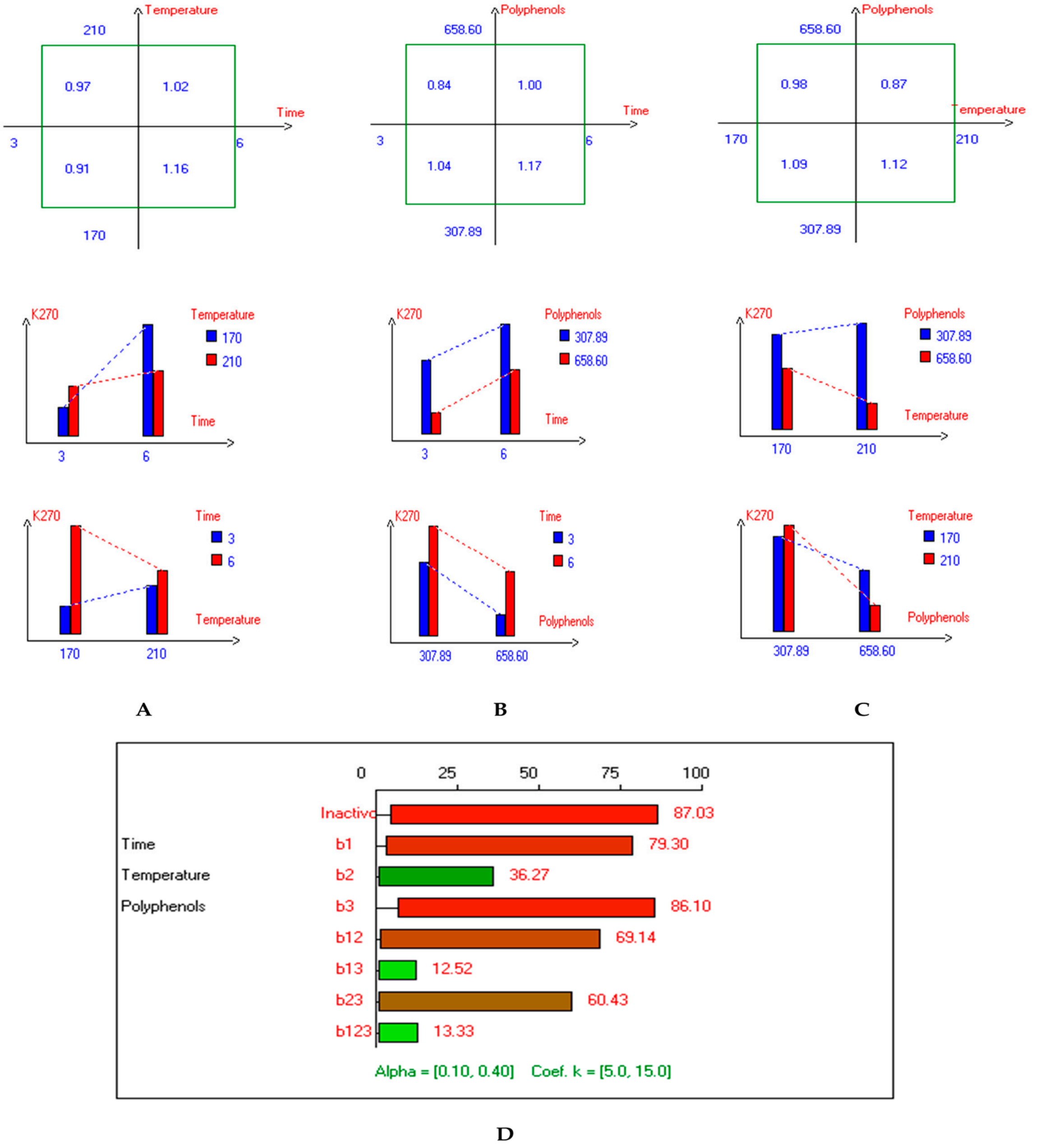

Figure S14. Combined interactions between the independent variables on a response variable (**K_{270}**) in EVOO **Picual** under D-F: (**A**) x_1 and x_2, (**B**) x_1 and x_3, (**C**) x_2 and x_3, and (**D**) results of variance analysis of regression equation model and the significance changes in each individual independent variable and interaction between the combined independent variables on K_{270}; b represents a significant difference when be $>b_{123}$, while b represents no significant difference when be $\leq b_{123}$; b_1, b_2, b_3 are the main effects of the independent variables, while b_{12}, b_{13}, b_{23}, and b_{123} are the interaction effects of the independent variables. Moreover, x_1, x_2, and x_3 are coded variables (time, temperature, and polyphenols addition, respectively) for the experimental design in D-F process.

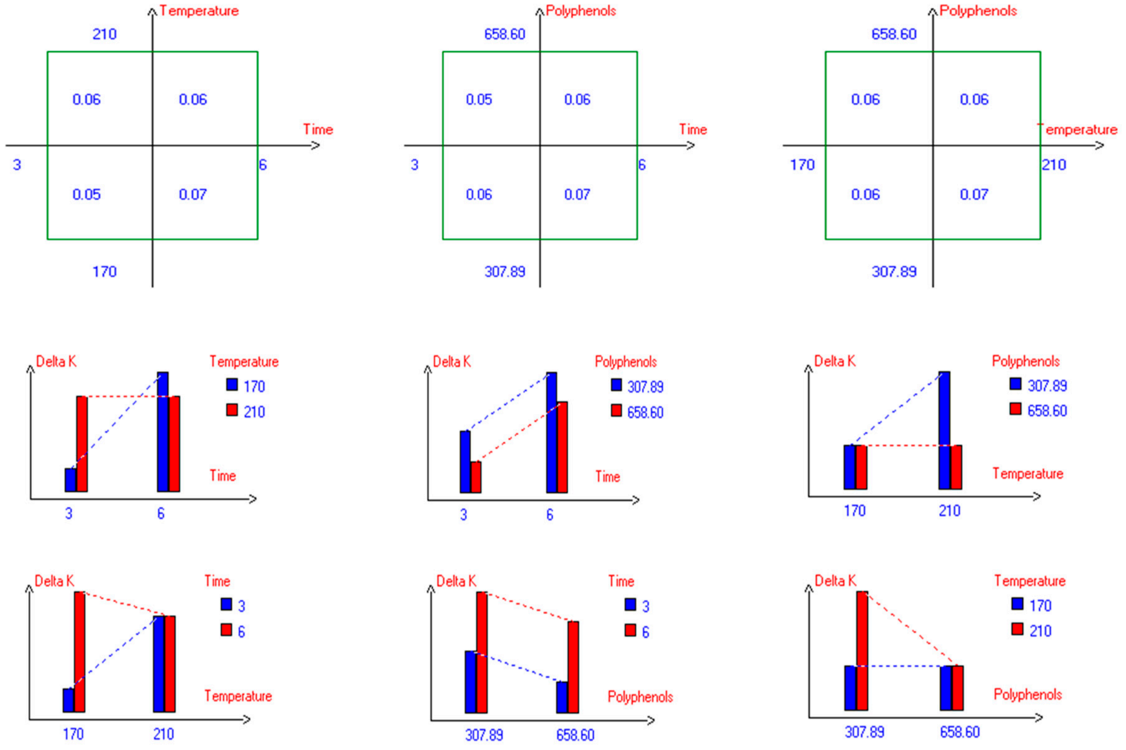

A **B** **C**

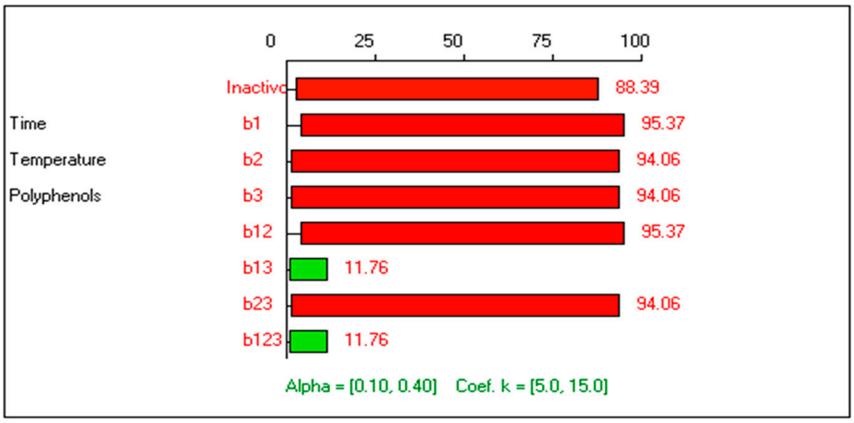

D

Figure S15. Combined interactions between the independent variables on a response variable (ΔK) in EVOO **Picual** under D-F: (**A**) x_1 and x_2, (**B**) x_1 and x_3, (**C**) x_2 and x_3, and (**D**) results of variance analysis of regression equation model and the significance changes in each individual independent variable and interaction between the combined independent variables on ΔK; b represents a significant difference when be $>b_{123}$, while b represents no significant difference when be $\leq b_{123}$; b_1, b_2, b_3 are the main effects of the independent variables, while b_{12}, b_{13}, b_{23}, and b_{123} are the interaction effects of the independent variables. Moreover, x_1, x_2, and x_3 are coded variables (time, temperature, and polyphenols addition, respectively) for the experimental design in D-F process.

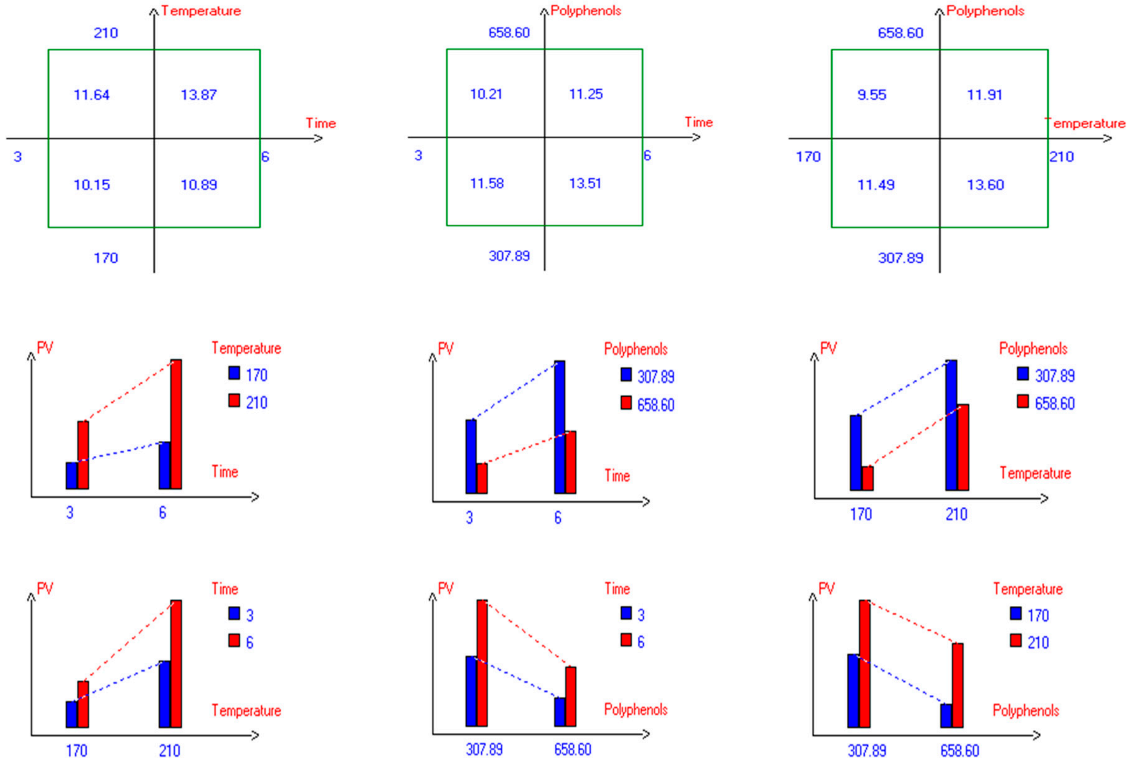

A **B** **C**

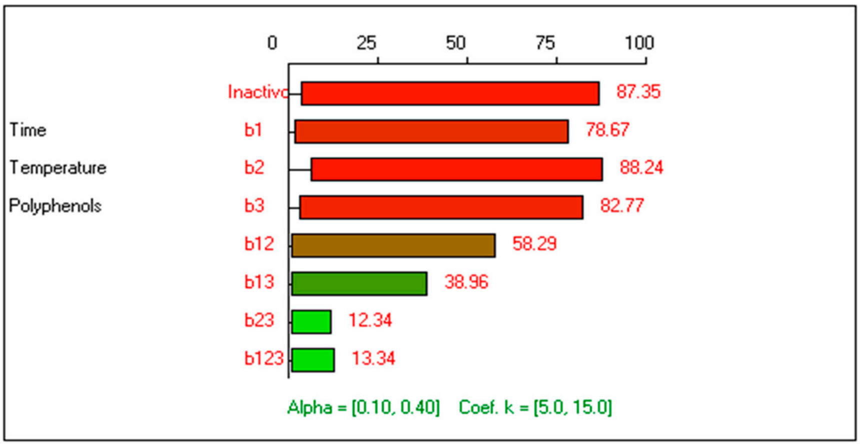

D

Figure S16. Combined interactions between the independent variables on a response variable (**peroxide value** (mEqO_2/kg)) in EVOO **Picual** under D-F: (**A**) x_1 and x_2, (**B**) x_1 and x_3, (**C**) x_2 and x_3, and (**D**) results of variance analysis of regression equation model and the significance changes in each individual independent variable and interaction between the combined independent variables on peroxide value; b represents a significant difference when be $>b_{123}$, while b represents no significant difference when be $\leq b_{123}$; b_1, b_2, b_3 are the main effects of the independent variables, while b_{12}, b_{13}, b_{23}, and b_{123} are the interaction effects of the independent variables. Moreover, x_1, x_2, and x_3 are coded variables (time, temperature, and polyphenols addition, respectively) for the experimental design in D-F process.

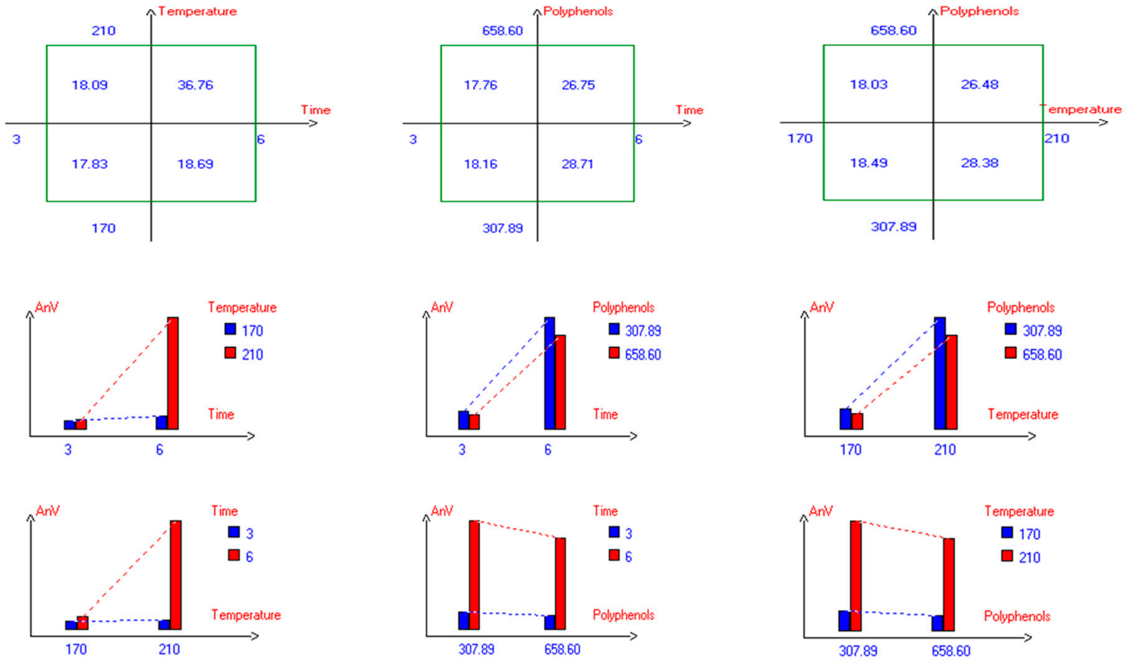

A **B** **C**

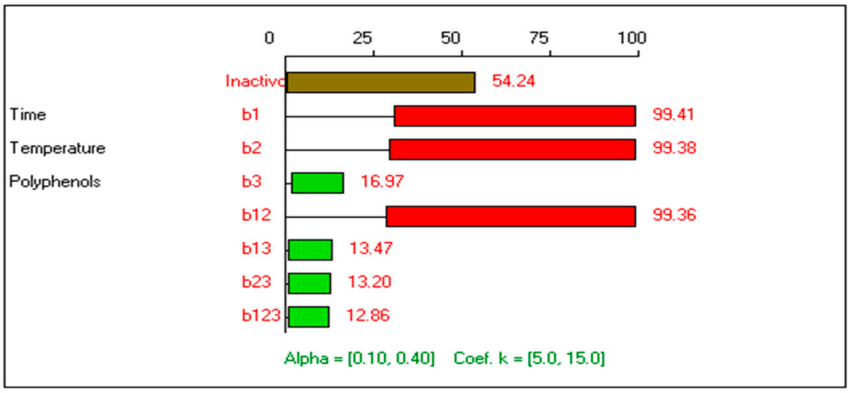

D

Figure S17. Combined interactions between the independent variables on a response variable (**anisidine value** (mg/kg)) in EVOO **Picual** under D-F: (**A**) x_1 and x_2, (**B**) x_1 and x_3, (**C**) x_2 and x_3, and (**D**) results of variance analysis of regression equation model and the significance changes in each individual independent variable and interaction between the combined independent variables on anisidine value; b represents a significant difference when be $>b_{123}$, while b represents no significant difference when be $\leq b_{123}$; b_1, b_2, b_3 are the main effects of the independent variables, while b_{12}, b_{13}, b_{23}, and b_{123} are the interaction effects of the independent variables. Moreover, x_1, x_2, and x_3 are coded variables (time, temperature, and polyphenols addition, respectively) for the experimental design in D-F process.

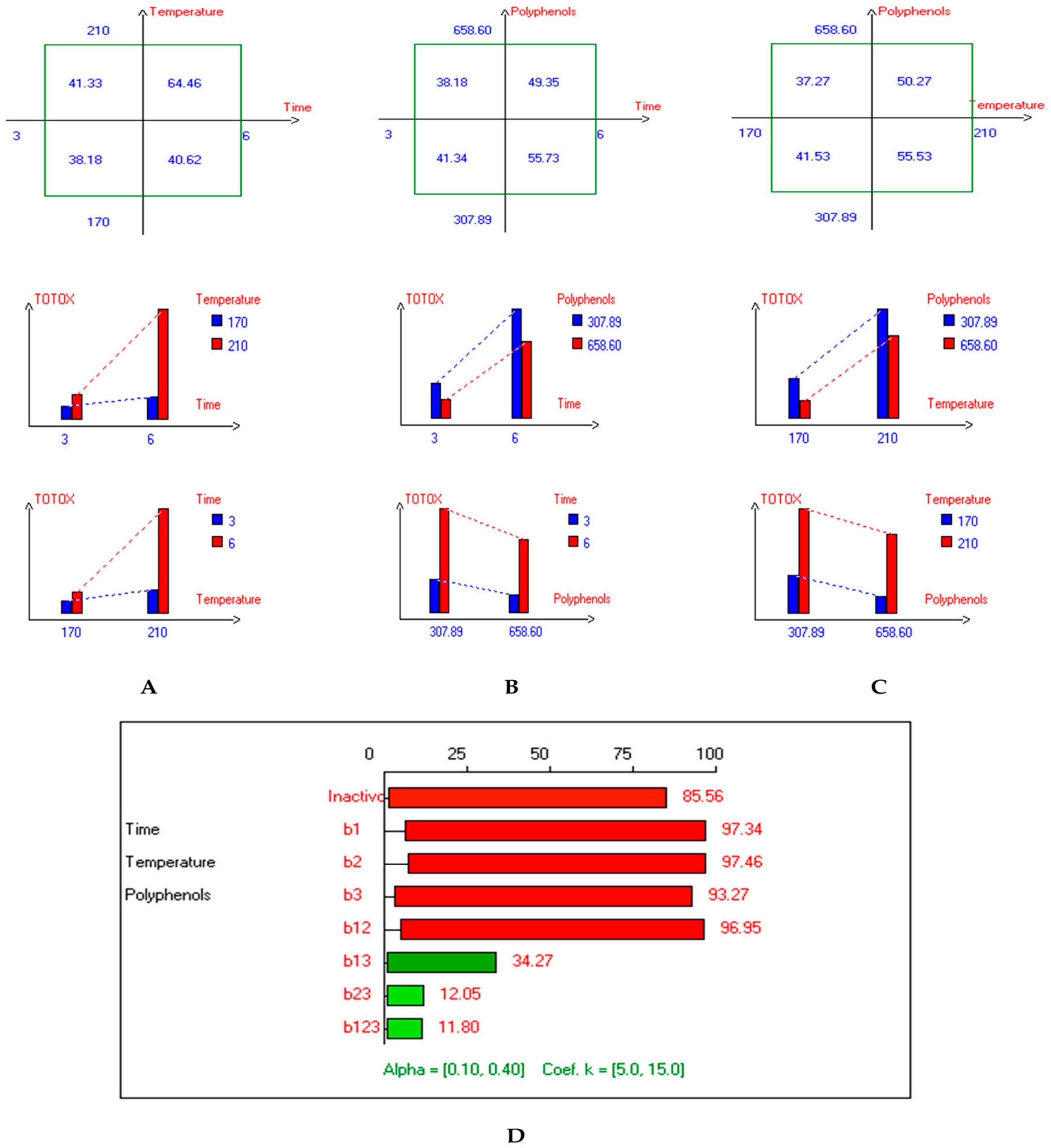

Figure S18. Combined interactions between the independent variables on a response variable (**TOTOX**) in EVOO **Picual** under D-F: (**A**) x_1 and x_2, (**B**) x_1 and x_3, (**C**) x_2 and x_3, and (**D**) results of variance analysis of regression equation model and the significance changes in each individual independent variable and interaction between the combined independent variables on TOTOX; b represents a significant difference when be $>b_{123}$, while b represents no significant difference when be $\leq b_{123}$; b_1, b_2, b_3 are the main effects of the independent variables, while b_{12}, b_{13}, b_{23}, and b_{123} are the interaction effects of the independent variables. Moreover, x_1, x_2, and x_3 are coded variables (time, temperature, and polyphenols addition, respectively) for the experimental design in D-F process.

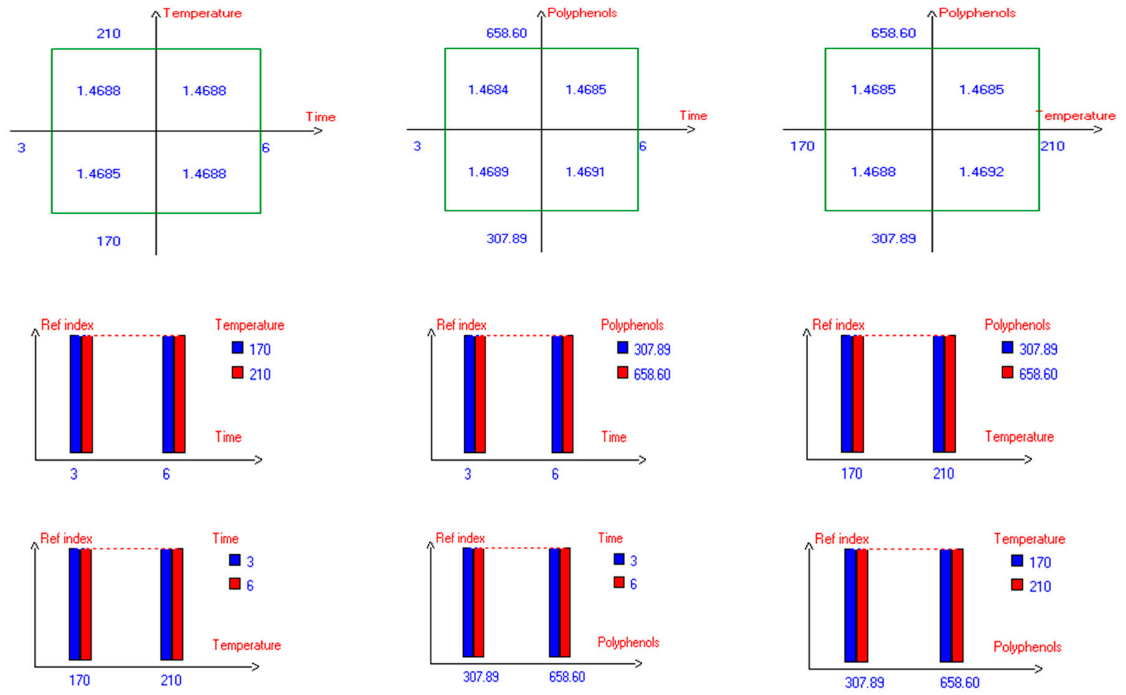

A B C

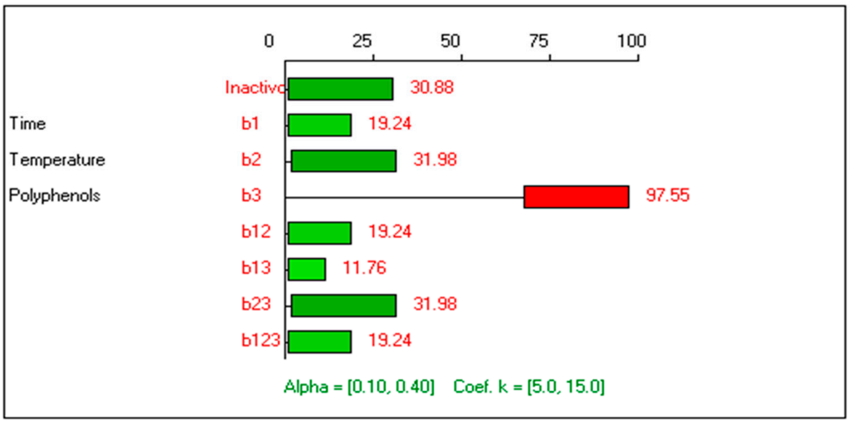

D

Figure S19. Combined interactions between the independent variables on a response variable (**refractive index**) in EVOO **Picual** under D-F: (**A**) x_1 and x_2, (**B**) x_1 and x_3, (**C**) x_2 and x_3, and (**D**) results of variance analysis of regression equation model and the significance changes in each individual independent variable and interaction between the combined independent variables on refractive index; b represents a significant difference when be $>b_{123}$, while b represents no significant difference when be $\leq b_{123}$; b_1, b_2, b_3 are the main effects of the independent variables, while b_{12}, b_{13}, b_{23}, and b_{123} are the interaction effects of the independent variables. Moreover, x_1, x_2, and x_3 are coded variables (time, temperature, and polyphenols addition, respectively) for the experimental design in D-F process.

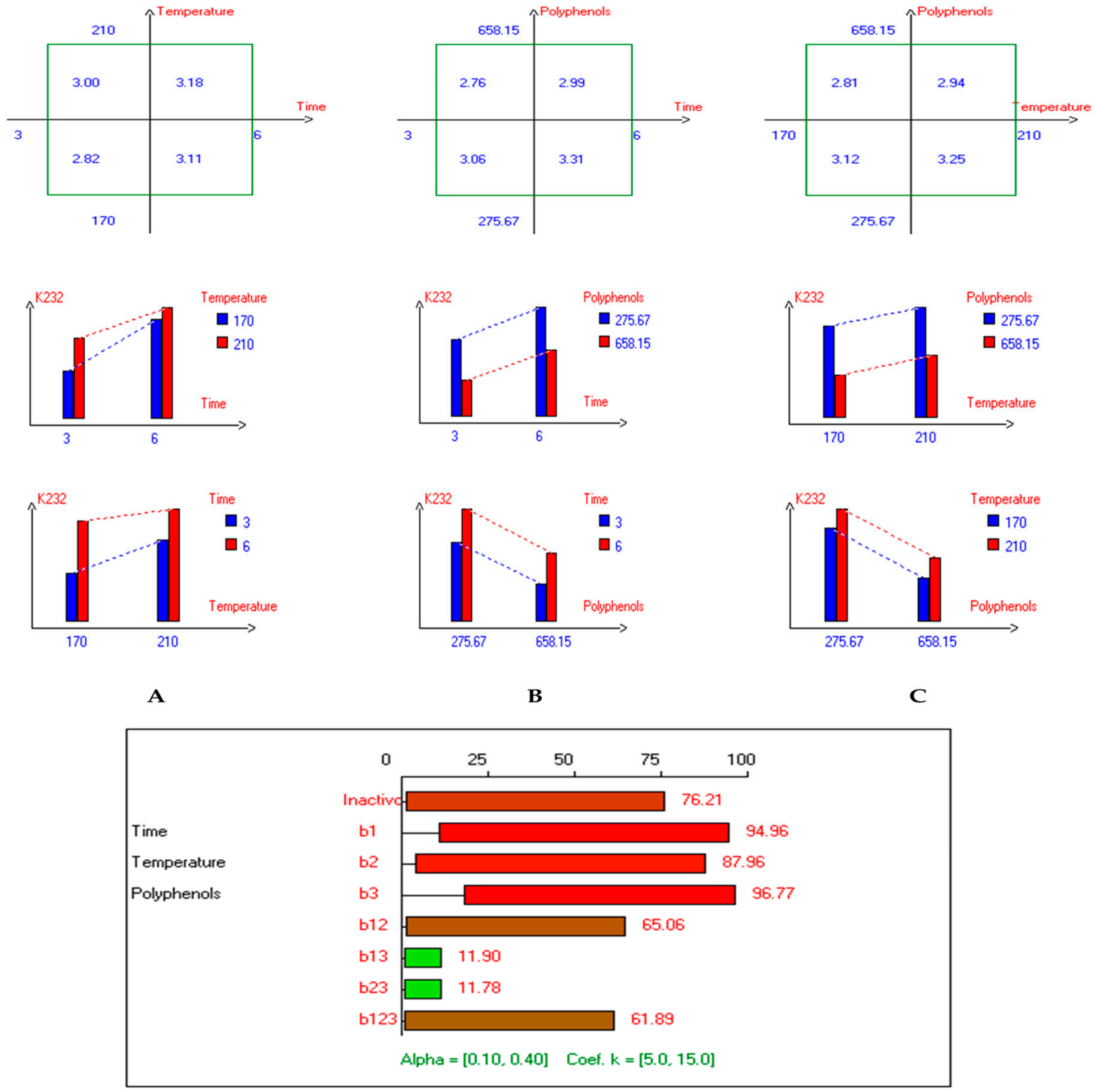

D

Figure S20. Combined interactions between the independent variables on a response variable (**K_{232}**) in EVOO **Cornicabra** under D-F: (**A**) x_1 and x_2, (**B**) x_1 and x_3, (**C**) x_2 and x_3, and (**D**) results of variance analysis of regression equation model and the significance changes in each individual independent variable and interaction between the combined independent variables on K_{232}; b represents a significant difference when be $>b_{123}$, while b represents no significant difference when be $\leq b_{123}$; b_1, b_2, b_3 are the main effects of the independent variables, while b_{12}, b_{13}, b_{23}, and b_{123} are the interaction effects of the independent variables. Moreover, x_1, x_2, and x_3 are coded variables (time, temperature, and polyphenols addition, respectively) for the experimental design in D-F process.

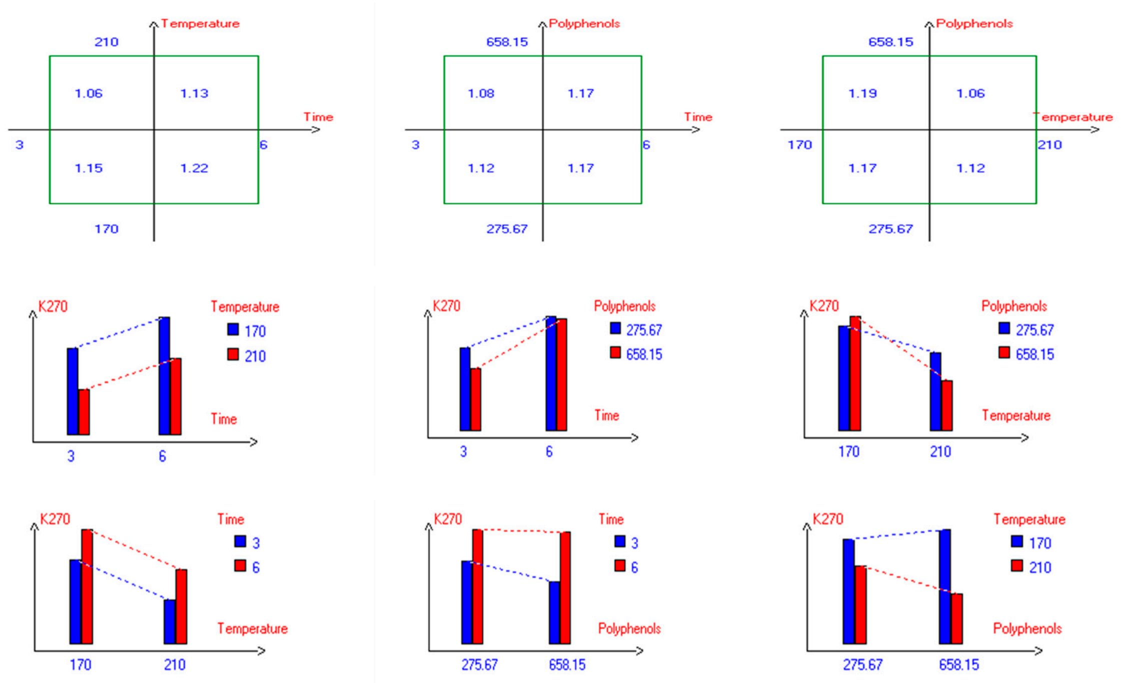

A B C

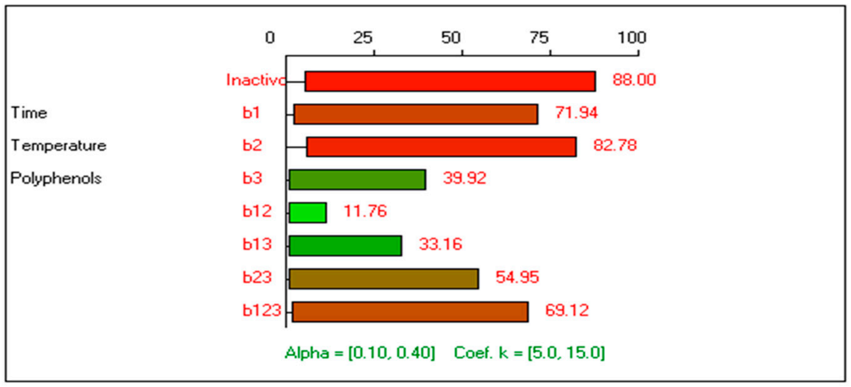

D

Figure S21. Combined interactions between the independent variables on a response variable (**K_{270}**) in EVOO **Cornicabra** under D-F: (**A**) x_1 and x_2, (**B**) x_1 and x_3, (**C**) x_2 and x_3, and (**D**) results of variance analysis of regression equation model and the significance changes in each individual independent variable and interaction between the combined independent variables on K_{270}; b represents a significant difference when be $>b_{123}$, while b represents no significant difference when be $\leq b_{123}$; b_1, b_2, b_3 are the main effects of the independent variables, while b_{12}, b_{13}, b_{23}, and b_{123} are the interaction effects of the independent variables. Moreover, x_1, x_2, and x_3 are coded variables (time, temperature, and polyphenols addition, respectively) for the experimental design in D-F process.

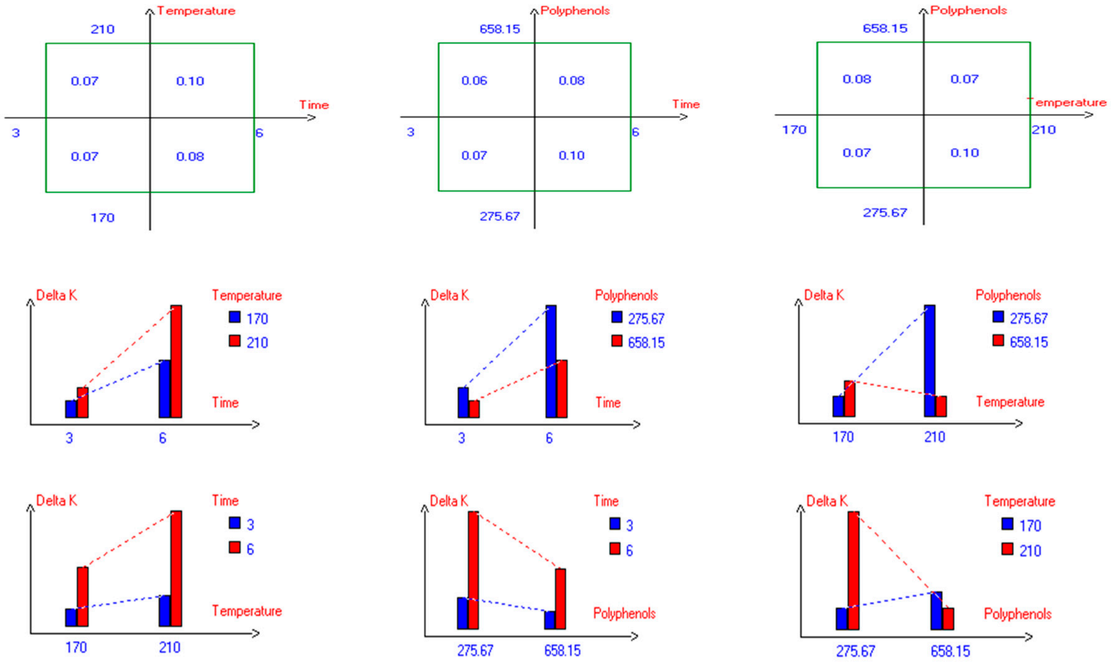

A **B** **C**

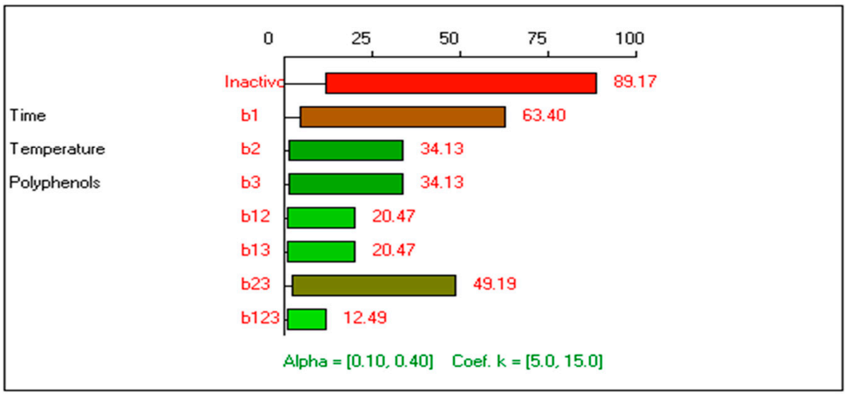

D

Figure S22. Combined interactions between the independent variables on a response variable (**ΔK**) in EVOO **Cornicabra** under D-F: (**A**) x_1 and x_2, (**B**) x_1 and x_3, (**C**) x_2 and x_3, and (**D**) results of variance analysis of regression equation model and the significance changes in each individual independent variable and interaction between the combined independent variables on ΔK; b represents a significant difference when be $>b_{123}$, while b represents no significant difference when be $\leq b_{123}$; b_1, b_2, b_3 are the main effects of the independent variables, while b_{12}, b_{13}, b_{23}, and b_{123} are the interaction effects of the independent variables. Moreover, x_1, x_2, and x_3 are coded variables (time, temperature, and polyphenols addition, respectively) for the experimental design in D-F process.

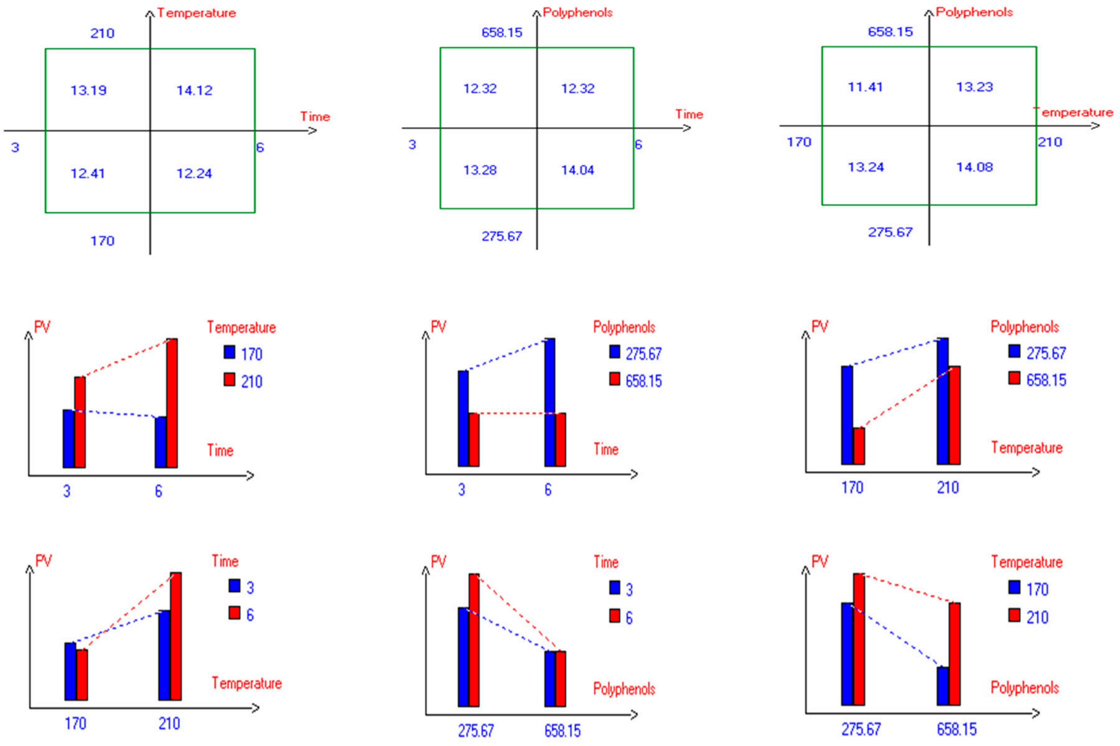

A **B** **C**

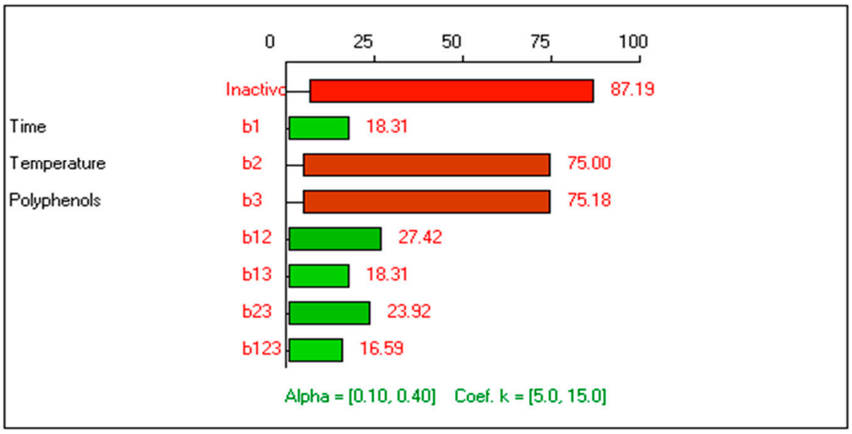

D

Figure S23. Combined interactions between the independent variables on a response variable (**peroxide value** (mEqO_2/kg)) in EVOO **Cornicabra** under D-F: (**A**) x_1 and x_2, (**B**) x_1 and x_3, (**C**) x_2 and x_3, and (**D**) results of variance analysis of regression equation model and the significance changes in each individual independent variable and interaction between the combined independent variables on peroxide value; b represents a significant difference when be >b_{123}, while b represents no significant difference when be ≤b_{123}; b_1, b_2, b_3 are the main effects of the independent variables, while b_{12}, b_{13}, b_{23}, and b_{123} are the interaction effects of the independent variables. Moreover, x_l, x_2, and x_3 are coded variables (time, temperature, and polyphenols addition, respectively) for the experimental design in D-F process.

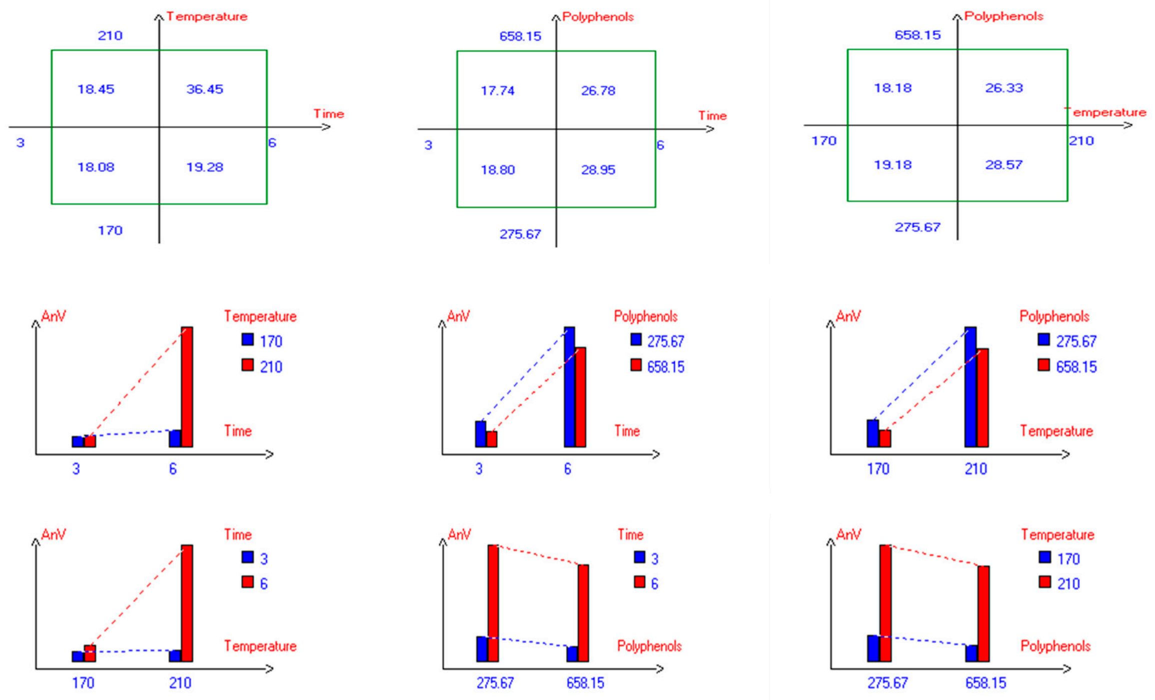

A B C

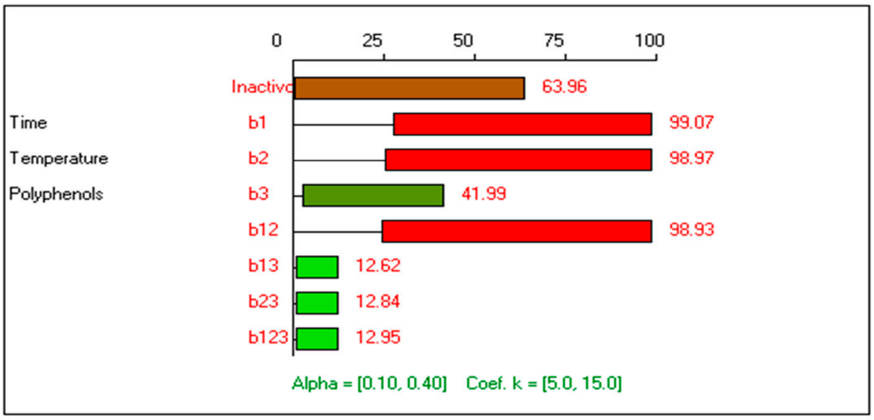

D

Figure S24. Combined interactions between the independent variables on a response variable (**anisidine value** (mg/kg)) in EVOO **Cornicabra** under D-F: (**A**) x_1 and x_2, (**B**) x_1 and x_3, (**C**) x_2 and x_3, and (**D**) results of variance analysis of regression equation model and the significance changes in each individual independent variable and interaction between the combined independent variables on anisidine value; b represents a significant difference when be $>b_{123}$, while b represents no significant difference when be $\leq b_{123}$; b_1, b_2, b_3 are the main effects of the independent variables, while b_{12}, b_{13}, b_{23}, and b_{123} are the interaction effects of the independent variables. Moreover, x_1, x_2, and x_3 are coded variables (time, temperature, and polyphenols addition, respectively) for the experimental design in D-F process.

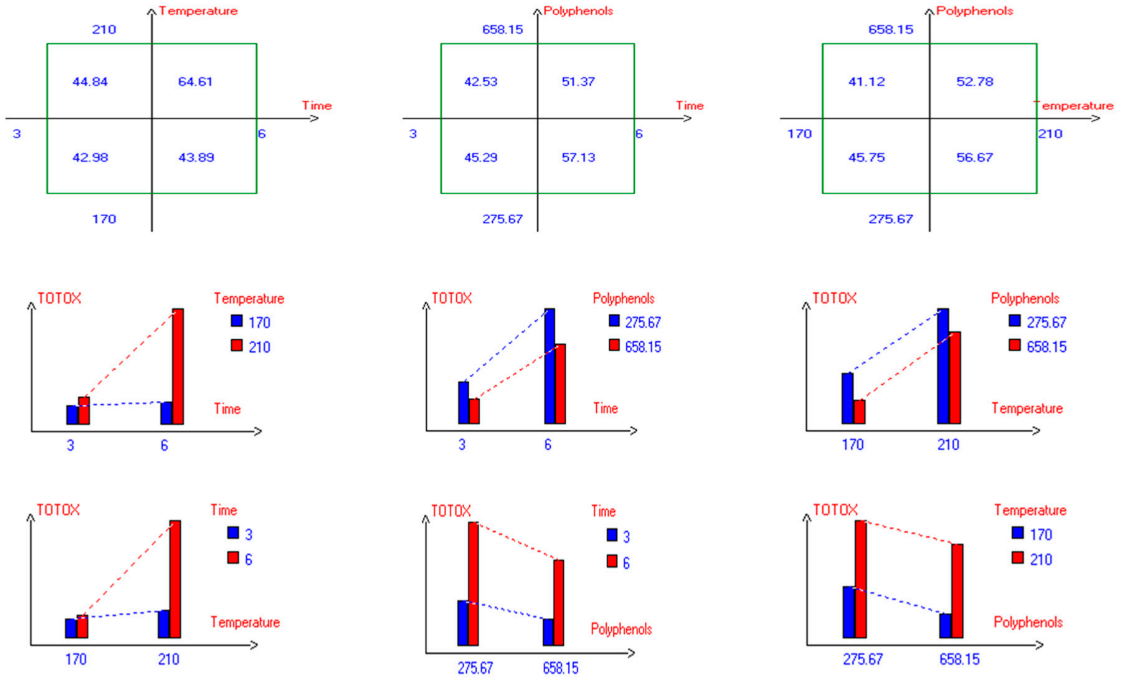

A **B** **C**

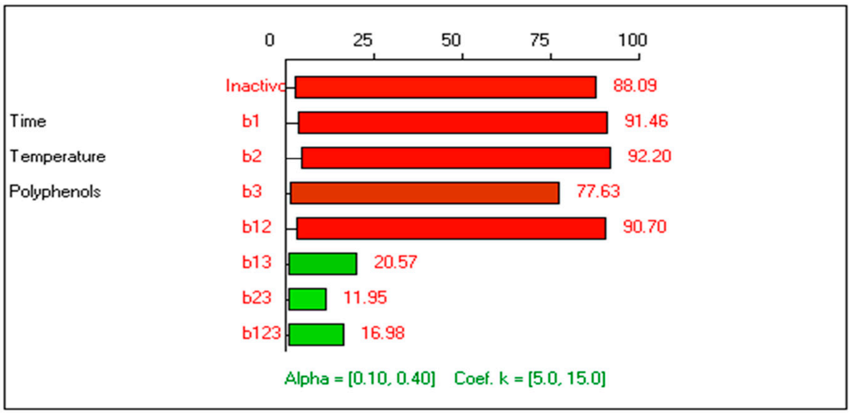

D

Figure S25. Combined interactions between the independent variables on a response variable (**TOTOX**) in EVOO **Cornicabra** under D-F: (**A**) x_1 and x_2, (**B**) x_1 and x_3, (**C**) x_2 and x_3, and (**D**) results of variance analysis of regression equation model and the significance changes in each individual independent variable and interaction between the combined independent variables on TOTOX; b represents a significant difference when be $>b_{123}$, while b represents no significant difference when be $\leq b_{123}$; b_1, b_2, b_3 are the main effects of the independent variables, while b_{12}, b_{13}, b_{23}, and b_{123} are the interaction effects of the independent variables. Moreover, x_1, x_2, and x_3 are coded variables (time, temperature, and polyphenols addition, respectively) for the experimental design in D-F process.

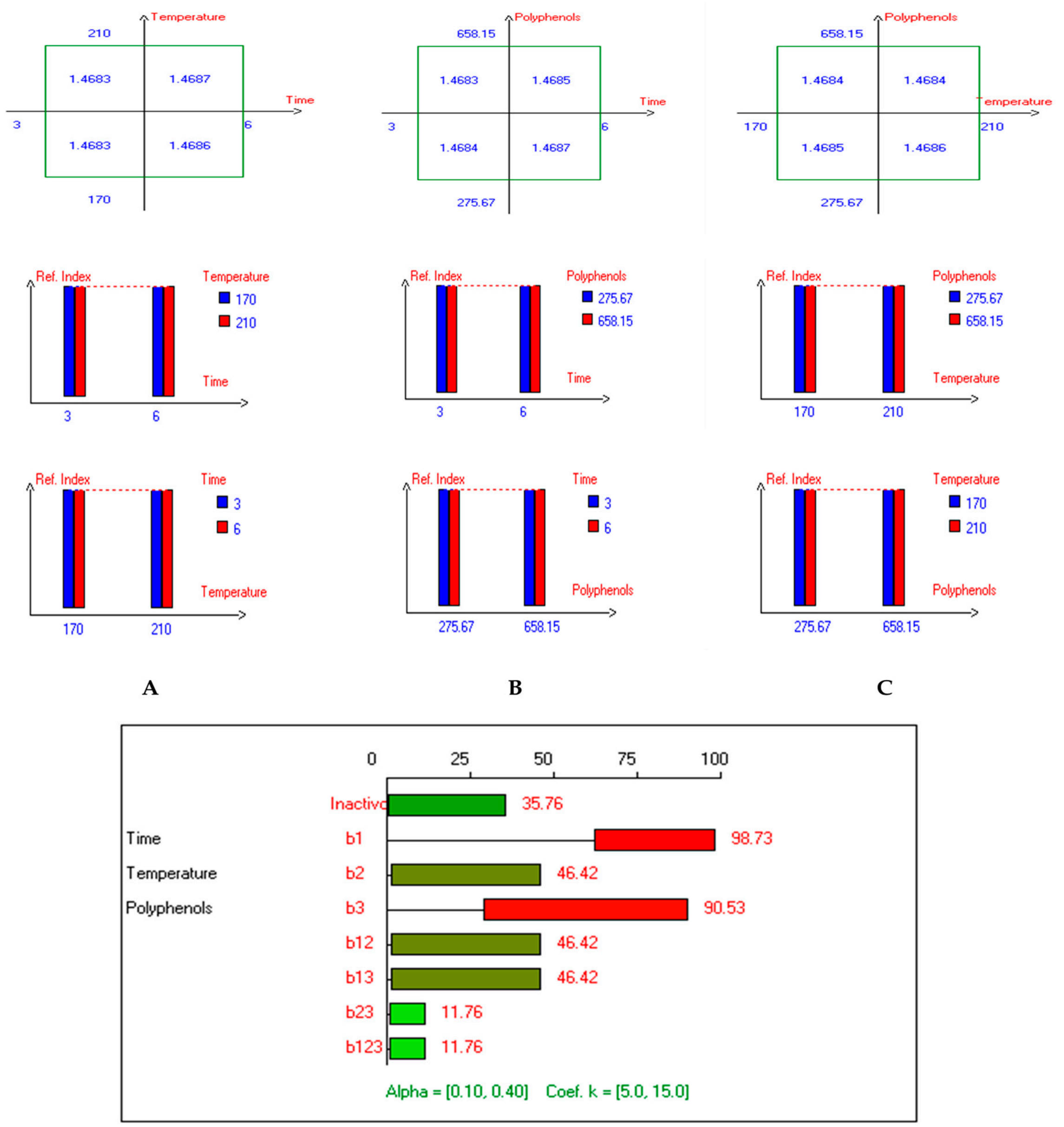

D

Figure S26. Combined interactions between the independent variables on a response variable (**refractive index**) in EVOO **Cornicabra** under D-F: (**A**) x_1 and x_2, (**B**) x_1 and x_3, (**C**) x_2 and x_3, and (**D**) results of variance analysis of regression equation model and the significance changes in each individual independent variable and interaction between the combined independent variables on refractive index; b represents a significant difference when be $>b_{123}$, while b represents no significant difference when be $\leq b_{123}$; b_1, b_2, b_3 are the main effects of the independent variables, while b_{12}, b_{13}, b_{23}, and b_{123} are the interaction effects of the independent variables. Moreover, x_l, x_2, and x_3 are coded variables (time, temperature, and polyphenols addition, respectively) for the experimental design in D-F process.

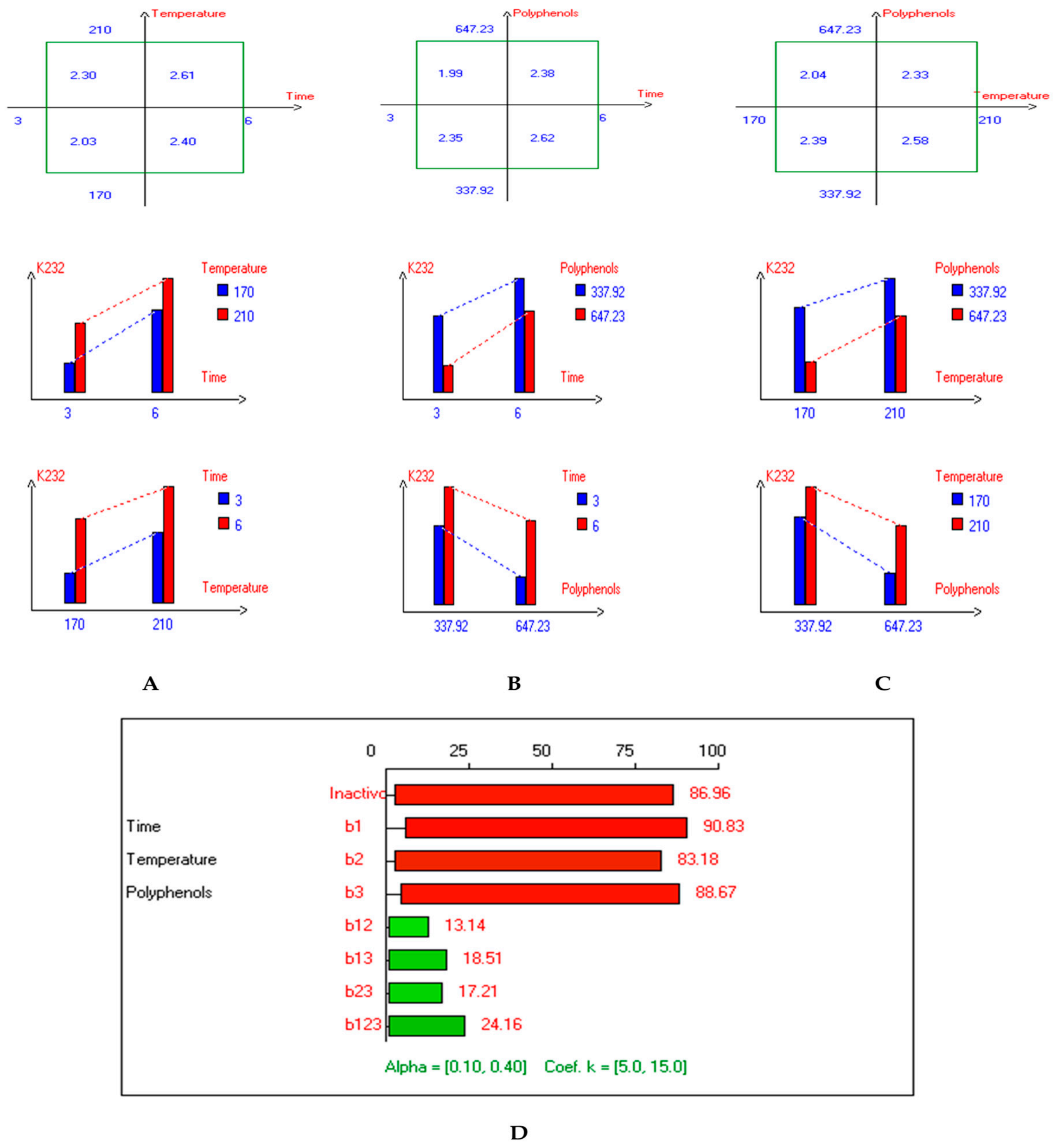

Figure S27. Combined interactions between the independent variables on a response variable (**K$_{232}$**) in EVOO **Empeltre** under D-F: (**A**) x_1 and x_2, (**B**) x_1 and x_3, (**C**) x_2 and x_3, and (**D**) results of variance analysis of regression equation model and the significance changes in each individual independent variable and interaction between the combined independent variables on K$_{232}$; b represents a significant difference when be >b_{123}, while b represents no significant difference when be ≤b_{123}; b_1, b_2, b_3 are the main effects of the independent variables, while b_{12}, b_{13}, b_{23}, and b_{123} are the interaction effects of the independent variables. Moreover, x_I, x_2, and x_3 are coded variables (time, temperature, and polyphenols addition, respectively) for the experimental design in D-F process.

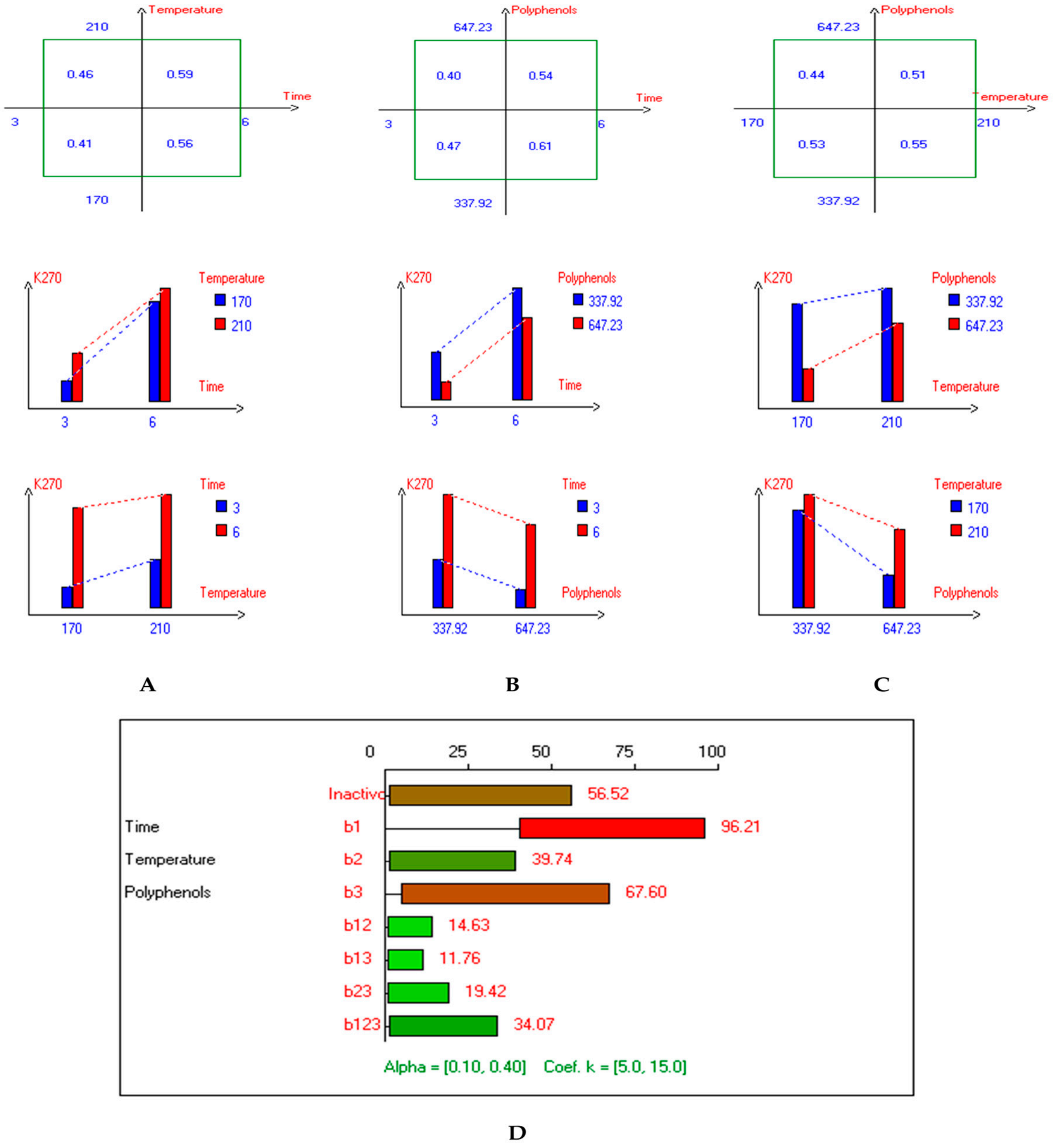

Figure S28. Combined interactions between the independent variables on a response variable (**K270**) in EVOO **Empeltre** under D-F: (**A**) x_1 and x_2, (**B**) x_1 and x_3, (**C**) x_2 and x_3, and (**D**) results of variance analysis of regression equation model and the significance changes in each individual independent variable and interaction between the combined independent variables on K_{270}; b represents a significant difference when be >b_{123}, while b represents no significant difference when be ≤b_{123}; b_1, b_2, b_3 are the main effects of the independent variables, while b_{12}, b_{13}, b_{23}, and b_{123} are the interaction effects of the independent variables. Moreover, x_1, x_2, and x_3 are coded variables (time, temperature, and polyphenols addition, respectively) for the experimental design in D-F process.

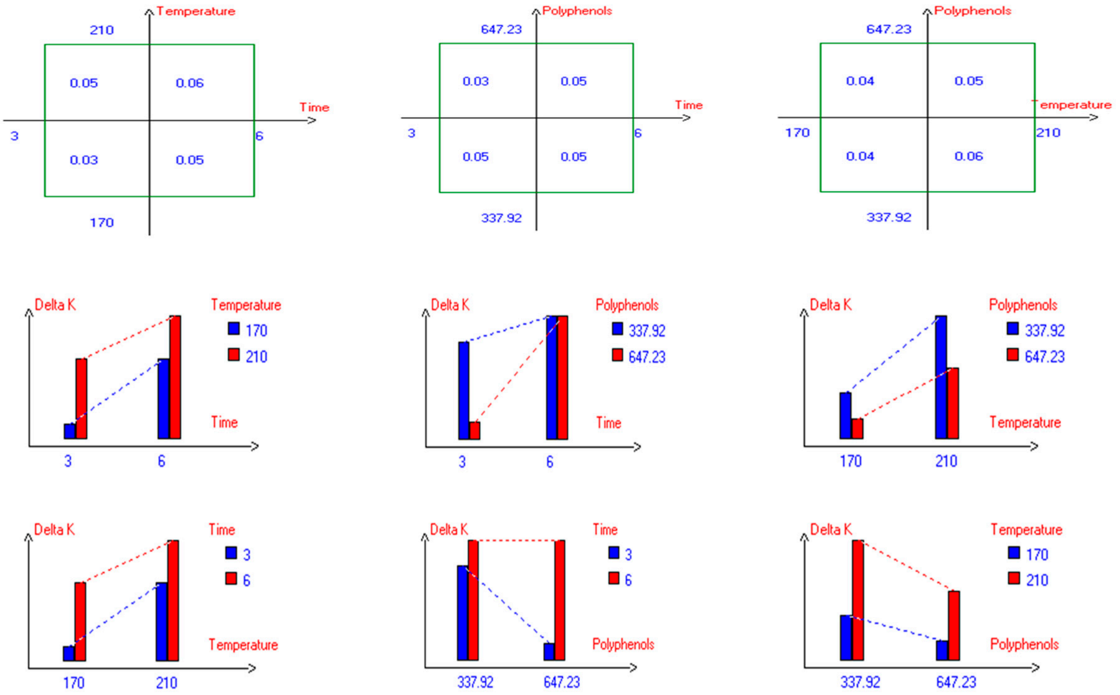

A **B** **C**

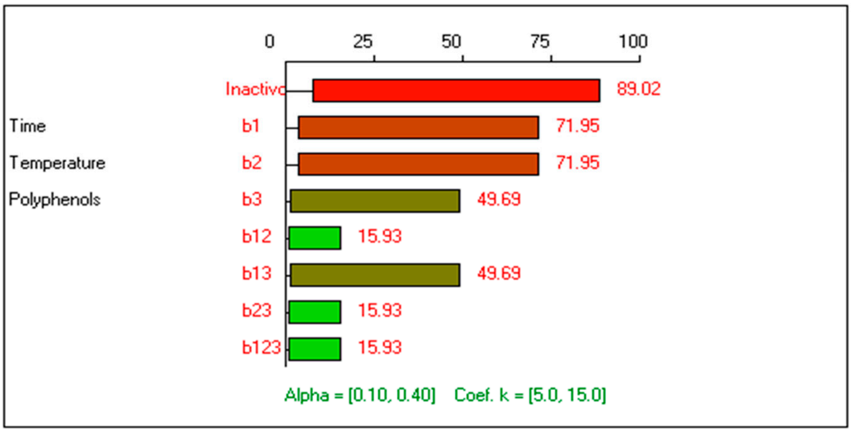

D

Figure S29. Combined interactions between the independent variables on a response variable (ΔK) in EVOO **Empeltre** under D-F: (**A**) x_1 and x_2, (**B**) x_1 and x_3, (**C**) x_2 and x_3, and (**D**) results of variance analysis of regression equation model and the significance changes in each individual independent variable and interaction between the combined independent variables on ΔK; b represents a significant difference when be $>b_{123}$, while b represents no significant difference when be $\leq b_{123}$; b_1, b_2, b_3 are the main effects of the independent variables, while b_{12}, b_{13}, b_{23}, and b_{123} are the interaction effects of the independent variables. Moreover, x_1, x_2, and x_3 are coded variables (time, temperature, and polyphenols addition, respectively) for the experimental design in D-F process.

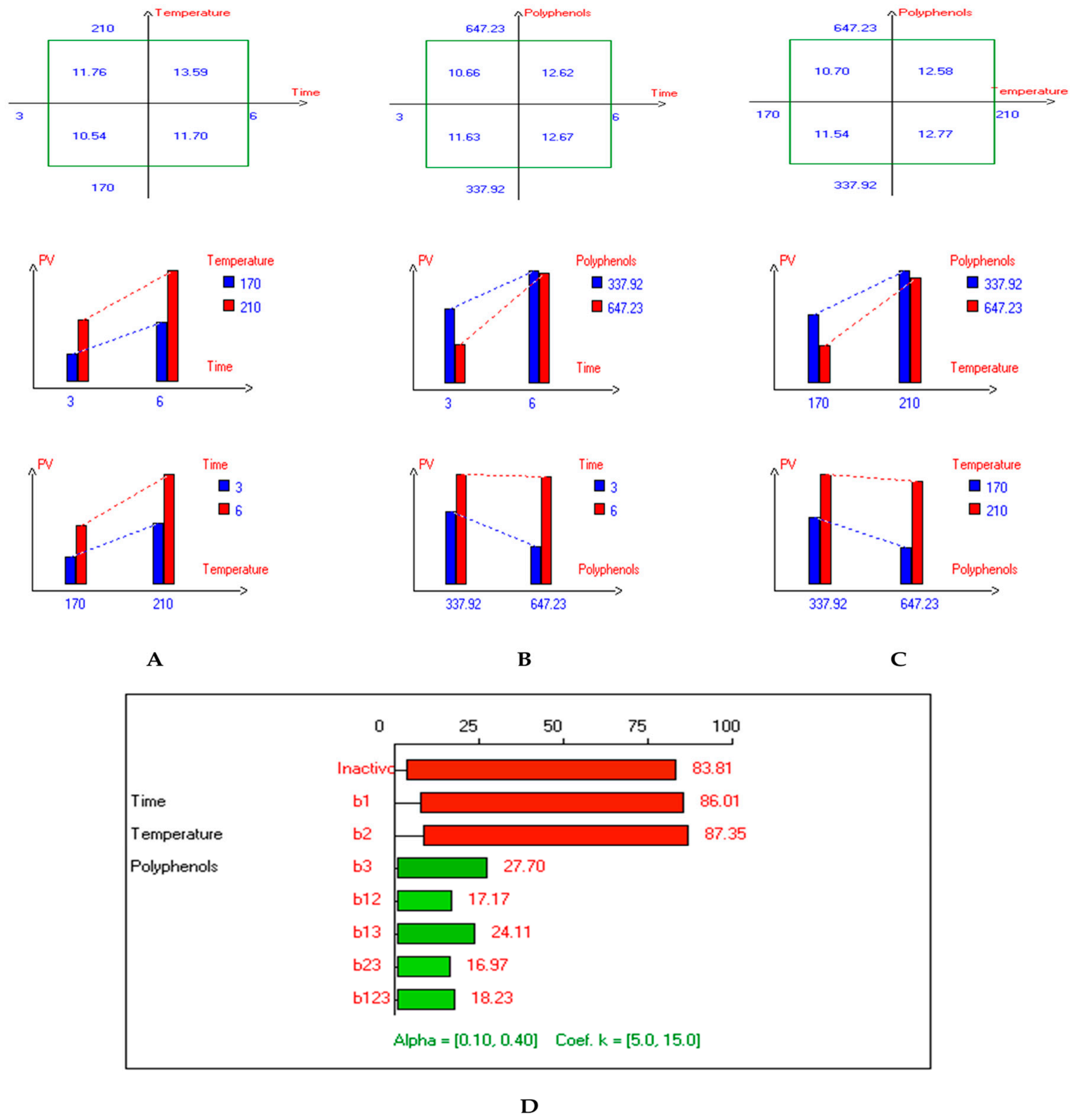

Figure S30. Combined interactions between the independent variables on a response variable (**peroxide value** (mEqO$_2$/kg)) in EVOO **Empeltre** under D-F: (**A**) x_1 and x_2, (**B**) x_1 and x_3, (**C**) x_2 and x_3, and (**D**) results of variance analysis of regression equation model and the significance changes in each individual independent variable and interaction between the combined independent variables on peroxide value; b represents a significant difference when be >b_{123}, while b represents no significant difference when be ≤b_{123}; b_1, b_2, b_3 are the main effects of the independent variables, while b_{12}, b_{13}, b_{23}, and b_{123} are the interaction effects of the independent variables. Moreover, x_1, x_2, and x_3 are coded variables (time, temperature, and polyphenols addition, respectively) for the experimental design in D-F process.

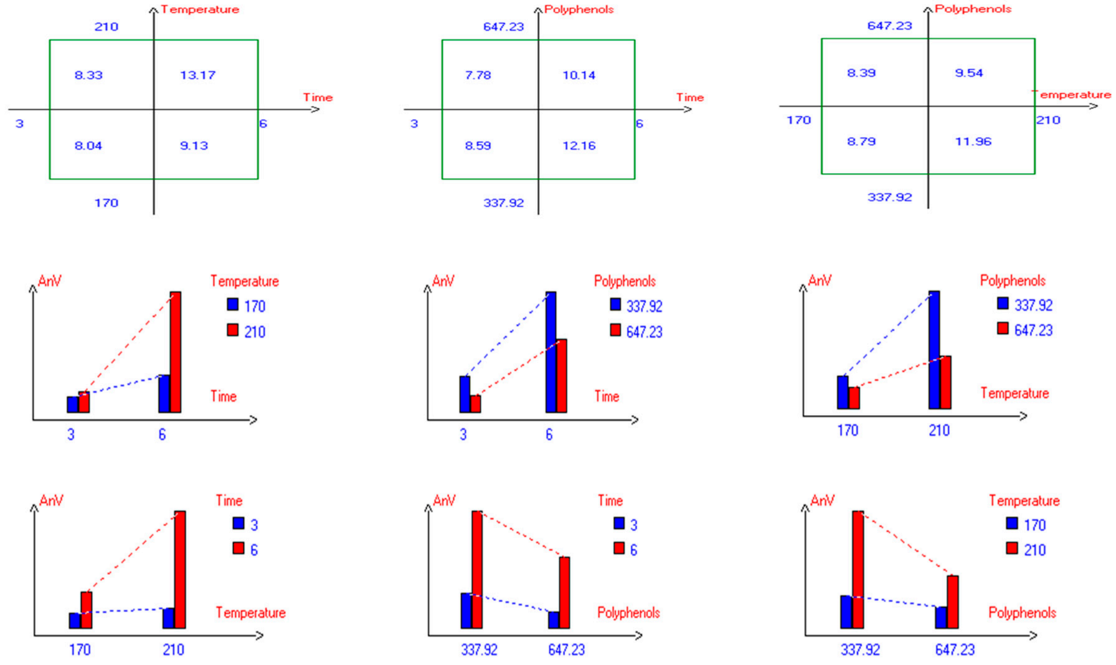

A **B** **C**

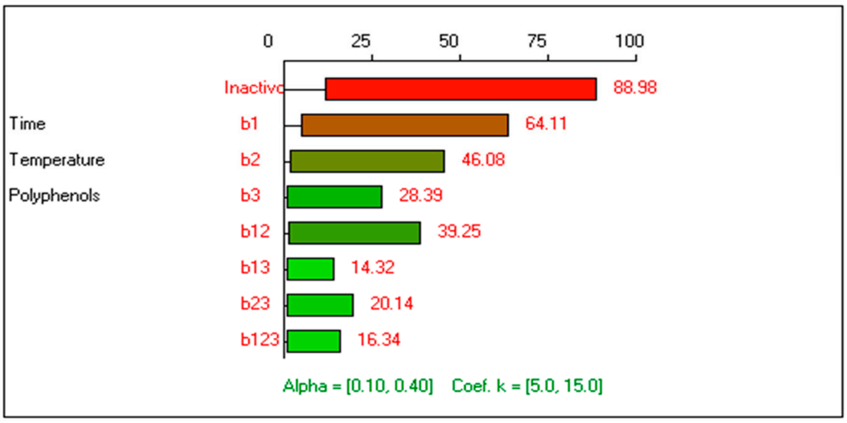

D

Figure S31. Combined interactions between the independent variables on a response variable (**anisidine value** (mg/kg)) in EVOO **Empeltre** under D-F: (**A**) x_1 and x_2, (**B**) x_1 and x_3, (**C**) x_2 and x_3, and (**D**) results of variance analysis of regression equation model and the significance changes in each individual independent variable and interaction between the combined independent variables on anisidine value; b represents a significant difference when be >b_{123}, while b represents no significant difference when be ≤b_{123}; b_1, b_2, b_3 are the main effects of the independent variables, while b_{12}, b_{13}, b_{23}, and b_{123} are the interaction effects of the independent variables. Moreover, x_1, x_2, and x_3 are coded variables (time, temperature, and polyphenols addition, respectively) for the experimental design in D-F process.

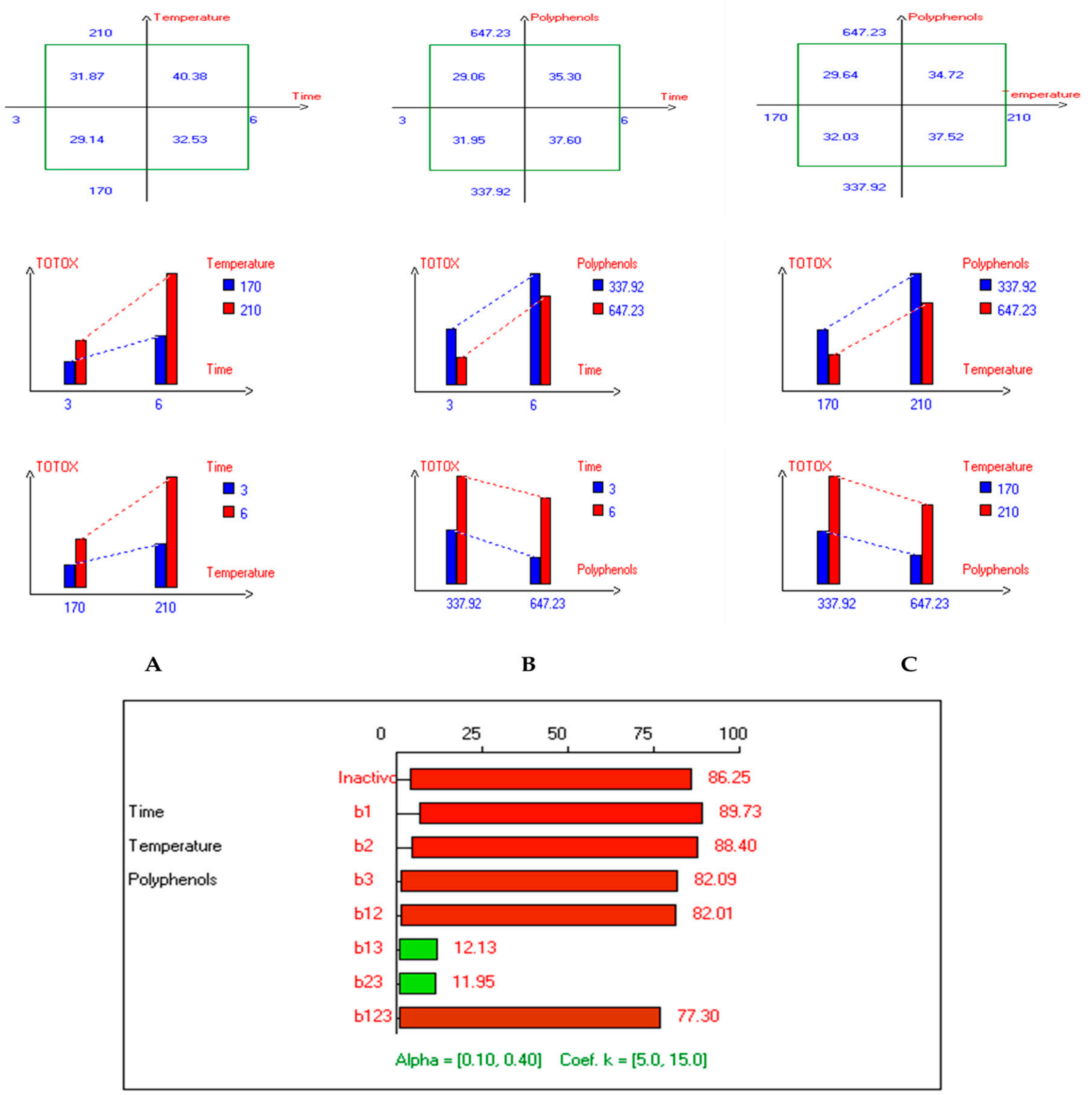

D

Figure S32. Combined interactions between the independent variables on a response variable (**TOTOX**) in EVOO **Empeltre** under D-F: (**A**) x_1 and x_2, (**B**) x_1 and x_3, (**C**) x_2 and x_3, and (**D**) results of variance analysis of regression equation model and the significance changes in each individual independent variable and interaction between the combined independent variables on TOTOX; b represents a significant difference when be >b_{123}, while b represents no significant difference when be ≤b_{123}; b_1, b_2, b_3 are the main effects of the independent variables, while b_{12}, b_{13}, b_{23}, and b_{123} are the interaction effects of the independent variables. Moreover, x_1, x_2, and x_3 are coded variables (time, temperature, and polyphenols addition, respectively) for the experimental design in D-F process.

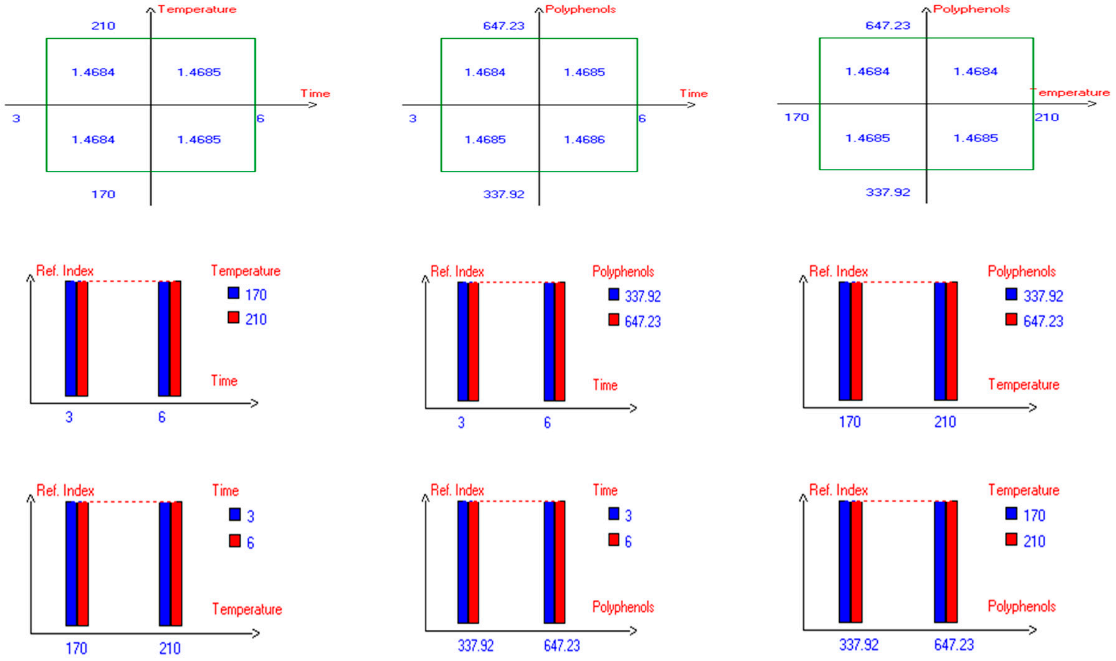

A **B** **C**

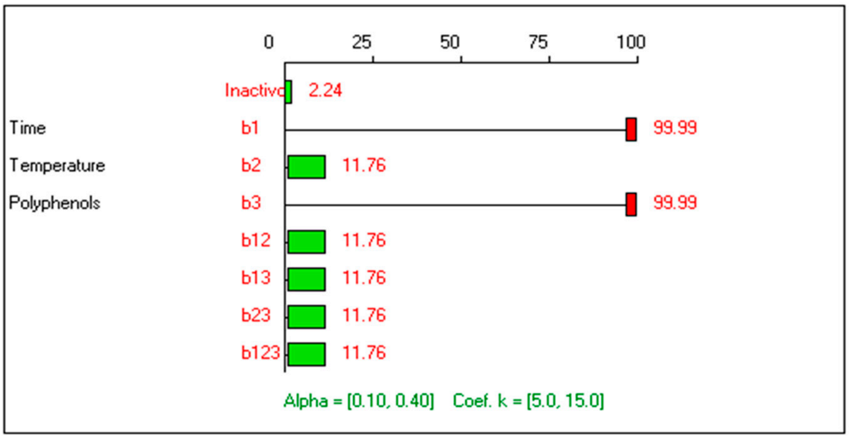

D

Figure S33. Combined interactions between the independent variables on a response variable (**refractive index**) in EVOO **Empeltre** under D-F: (**A**) x_1 and x_2, (**B**) x_1 and x_3, (**C**) x_2 and x_3, and (**D**) results of variance analysis of regression equation model and the significance changes in each individual independent variable and interaction between the combined independent variables on refractive index; b represents a significant difference when be >b_{123}, while b represents no significant difference when be ≤b_{123}; b_1, b_2, b_3 are the main effects of the independent variables, while b_{12}, b_{13}, b_{23}, and b_{123} are the interaction effects of the independent variables. Moreover, x_1, x_2, and x_3 are coded variables (time, temperature, and polyphenols addition, respectively) for the experimental design in D-F process.

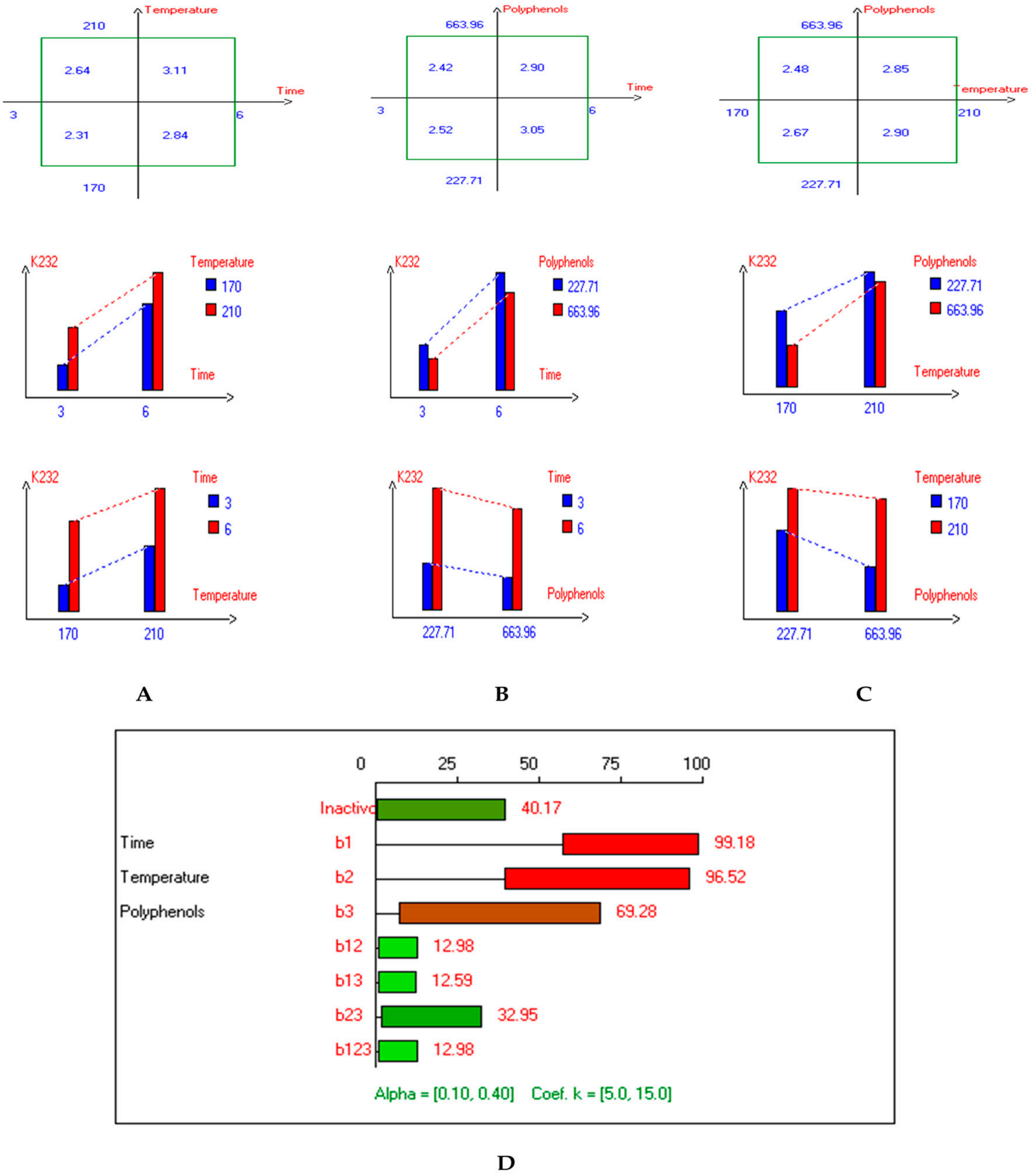

Figure S34. Combined interactions between the independent variables on a response variable (**K_{232}**) in EVOO **Arbequina** under D-F: (**A**) x_1 and x_2, (**B**) x_1 and x_3, (**C**) x_2 and x_3, and (**D**) results of variance analysis of regression equation model and the significance changes in each individual independent variable and interaction between the combined independent variables on K_{232}; b represents a significant difference when be >b_{123}, while b represents no significant difference when be ≤b_{123}; b_1, b_2, b_3 are the main effects of the independent variables, while b_{12}, b_{13}, b_{23}, and b_{123} are the interaction effects of the independent variables. Moreover, x_1, x_2, and x_3 are coded variables (time, temperature, and polyphenols addition, respectively) for the experimental design in D-F process.

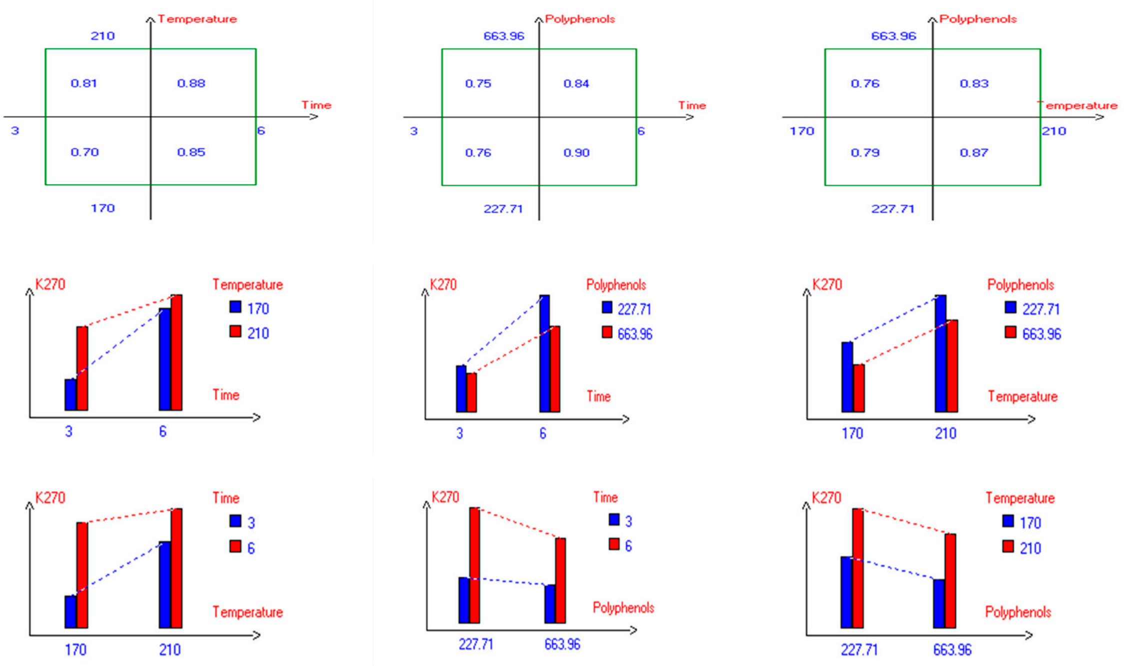

A **B** **C**

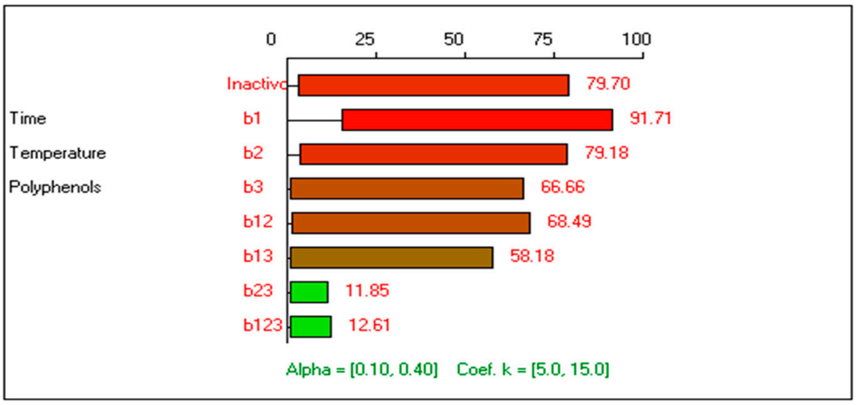

D

Figure S35. Combined interactions between the independent variables on a response variable (**K$_{270}$**) in EVOO **Arbequina** under D-F: (**A**) x_1 and x_2, (**B**) x_1 and x_3, (**C**) x_2 and x_3, and (**D**) results of variance analysis of regression equation model and the significance changes in each individual independent variable and interaction between the combined independent variables on K$_{270}$; b represents a significant difference when be >b_{123}, while b represents no significant difference when be ≤b_{123}; b_1, b_2, b_3 are the main effects of the independent variables, while b_{12}, b_{13}, b_{23}, and b_{123} are the interaction effects of the independent variables. Moreover, x_1, x_2, and x_3 are coded variables (time, temperature, and polyphenols addition, respectively) for the experimental design in D-F process.

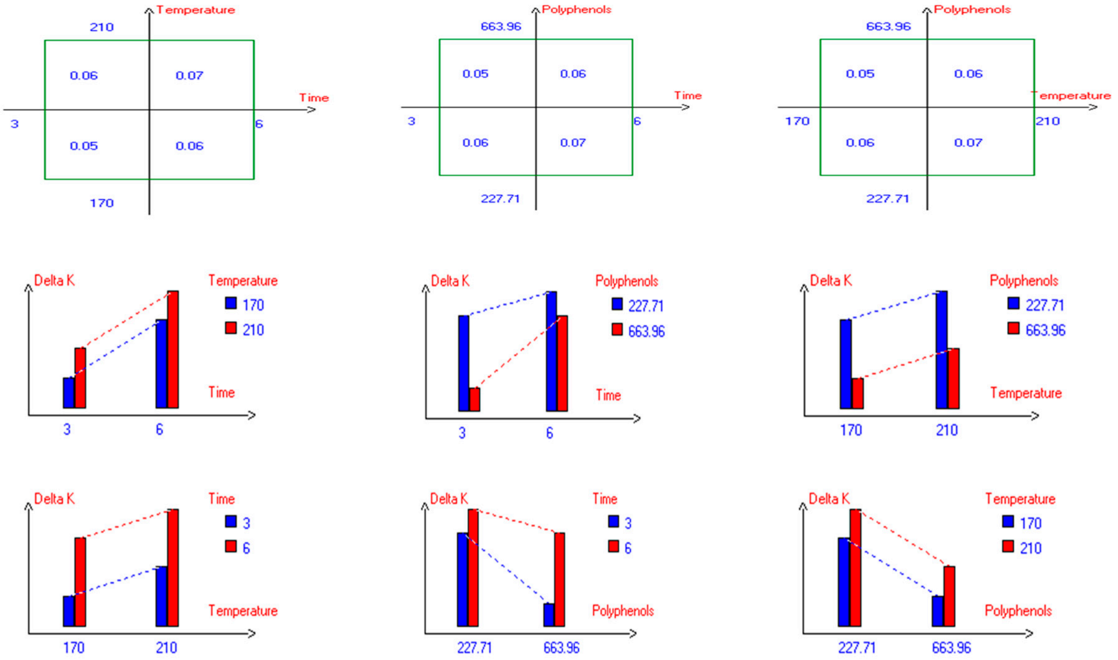

A **B** **C**

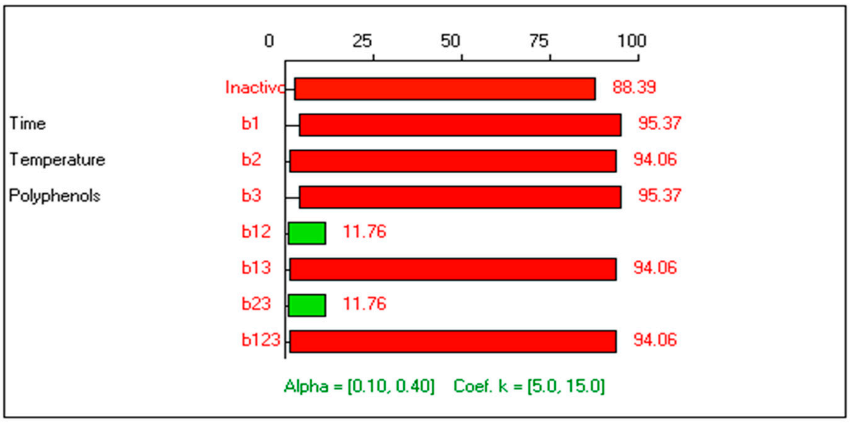

D

Figure S36. Combined interactions between the independent variables on a response variable (**ΔK**) in EVOO **Arbequina** under D-F: (**A**) x_1 and x_2, (**B**) x_1 and x_3, (**C**) x_2 and x_3, and (**D**) results of variance analysis of regression equation model and the significance changes in each individual independent variable and interaction between the combined independent variables on ΔK; b represents a significant difference when be $>b_{123}$, while b represents no significant difference when be $\leq b_{123}$; b_1, b_2, b_3 are the main effects of the independent variables, while b_{12}, b_{13}, b_{23}, and b_{123} are the interaction effects of the independent variables. Moreover, x_1, x_2, and x_3 are coded variables (time, temperature, and polyphenols addition, respectively) for the experimental design in D-F process.

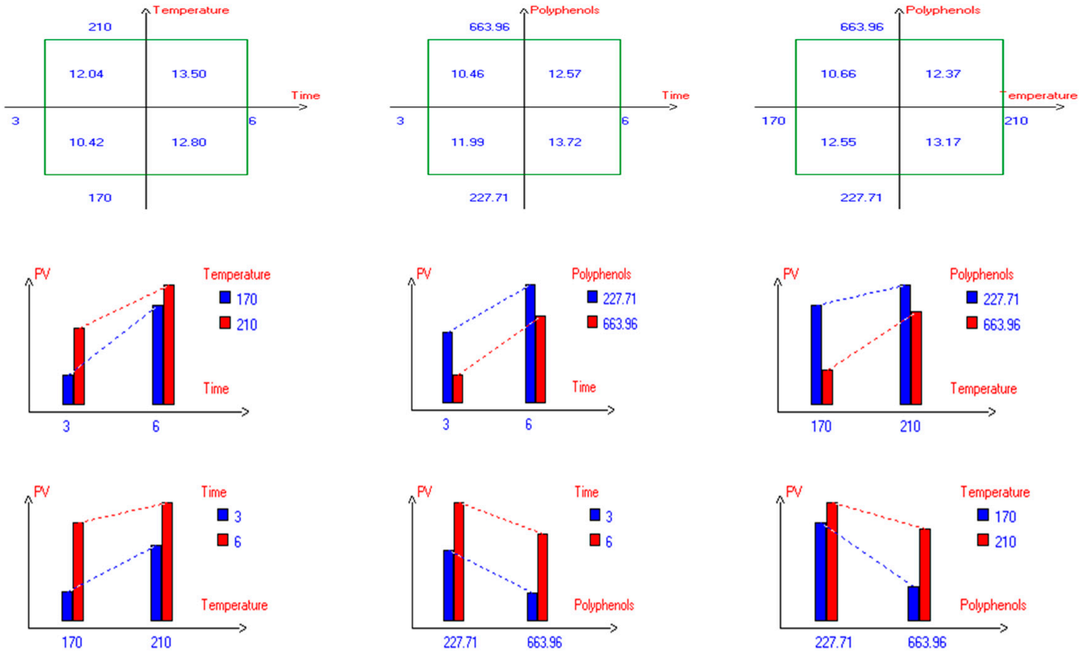

A B C

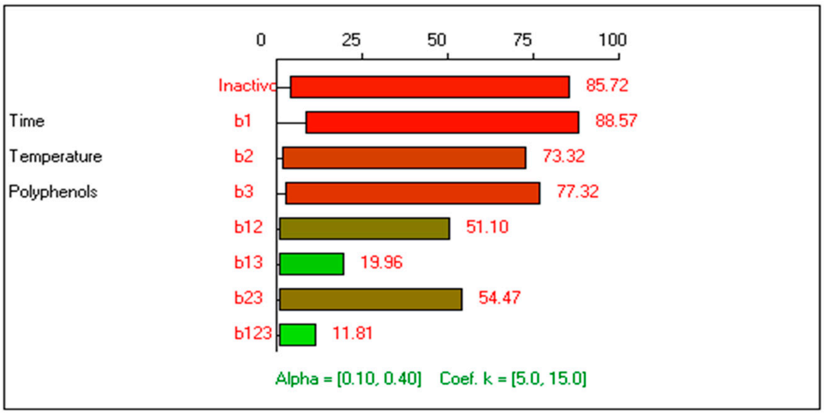

D

Figure S37. Combined interactions between the independent variables on a response variable (**peroxide value** ($mEqO_2/kg$)) in EVOO **Arbequina** under D-F: (**A**) x_1 and x_2, (**B**) x_1 and x_3, (**C**) x_2 and x_3, and (**D**) results of variance analysis of regression equation model and the significance changes in each individual independent variable and interaction between the combined independent variables on peroxide value; b represents a significant difference when be $>b_{123}$, while b represents no significant difference when be $\leq b_{123}$; b_1, b_2, b_3 are the main effects of the independent variables, while b_{12}, b_{13}, b_{23}, and b_{123} are the interaction effects of the independent variables. Moreover, x_1, x_2, and x_3 are coded variables (time, temperature, and polyphenols addition, respectively) for the experimental design in D-F process.

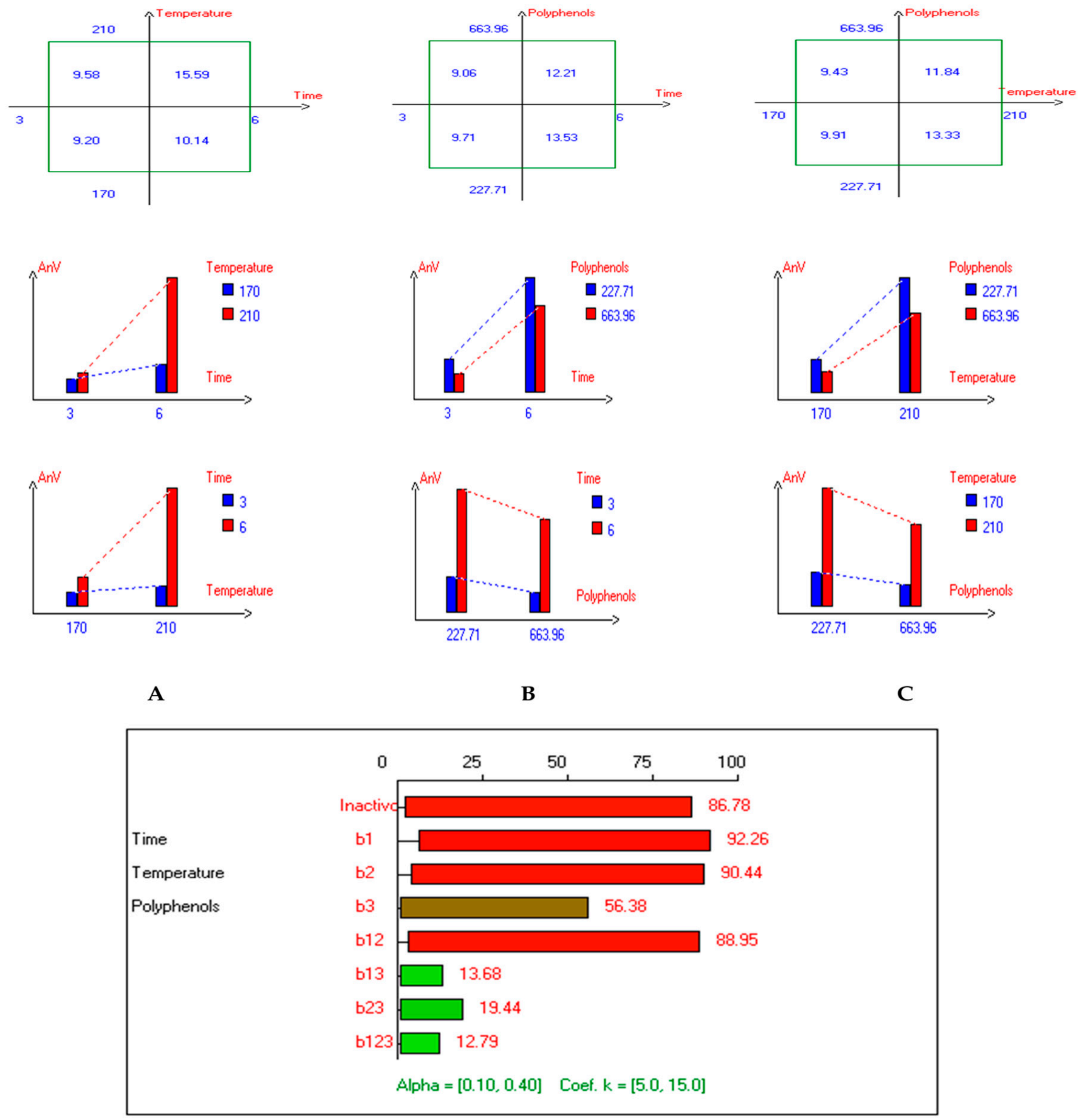

D

Figure S38. Combined interactions between the independent variables on a response variable (**anisidine value** (mg/kg)) in EVOO **Arbequina** under D-F: (**A**) x_1 and x_2, (**B**) x_1 and x_3, (**C**) x_2 and x_3, and (**D**) results of variance analysis of regression equation model and the significance changes in each individual independent variable and interaction between the combined independent variables on anisidine value; b represents a significant difference when be $>b_{123}$, while b represents no significant difference when be $\leq b_{123}$; b_1, b_2, b_3 are the main effects of the independent variables, while b_{12}, b_{13}, b_{23}, and b_{123} are the interaction effects of the independent variables. Moreover, x_1, x_2, and x_3 are coded variables (time, temperature, and polyphenols addition, respectively) for the experimental design in D-F process.

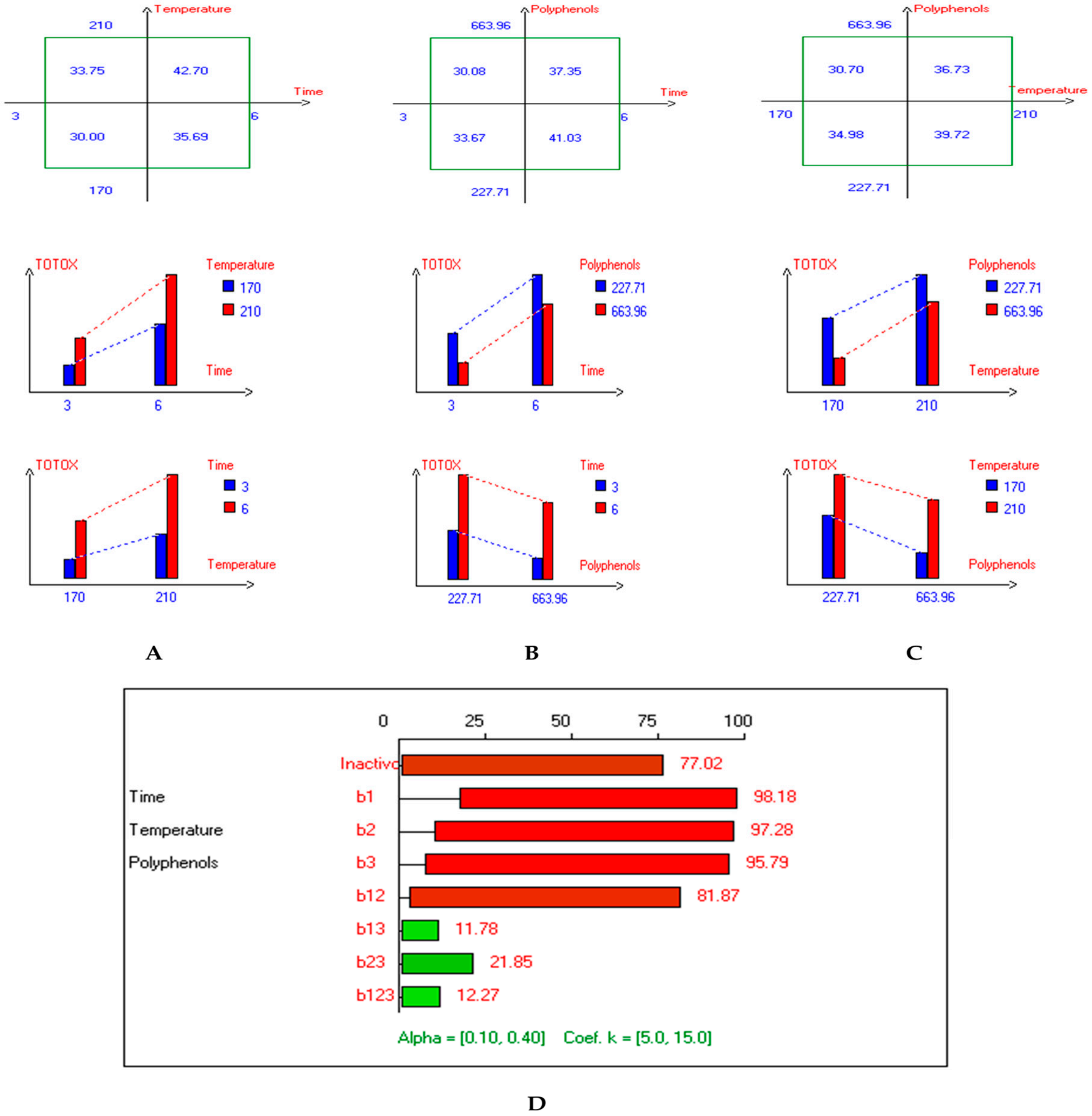

Figure S39. Combined interactions between the independent variables on a response variable (**TOTOX**) in EVOO **Arbequina** under D-F: (**A**) x_1 and x_2, (**B**) x_1 and x_3, (**C**) x_2 and x_3, and (**D**) results of variance analysis of regression equation model and the significance changes in each individual independent variable and interaction between the combined independent variables on TOTOX; b represents a significant difference when be $>b_{123}$, while b represents no significant difference when be $\leq b_{123}$; b_1, b_2, b_3 are the main effects of the independent variables, while b_{12}, b_{13}, b_{23}, and b_{123} are the interaction effects of the independent variables. Moreover, x_1, x_2, and x_3 are coded variables (time, temperature, and polyphenols addition, respectively) for the experimental design in D-F process.

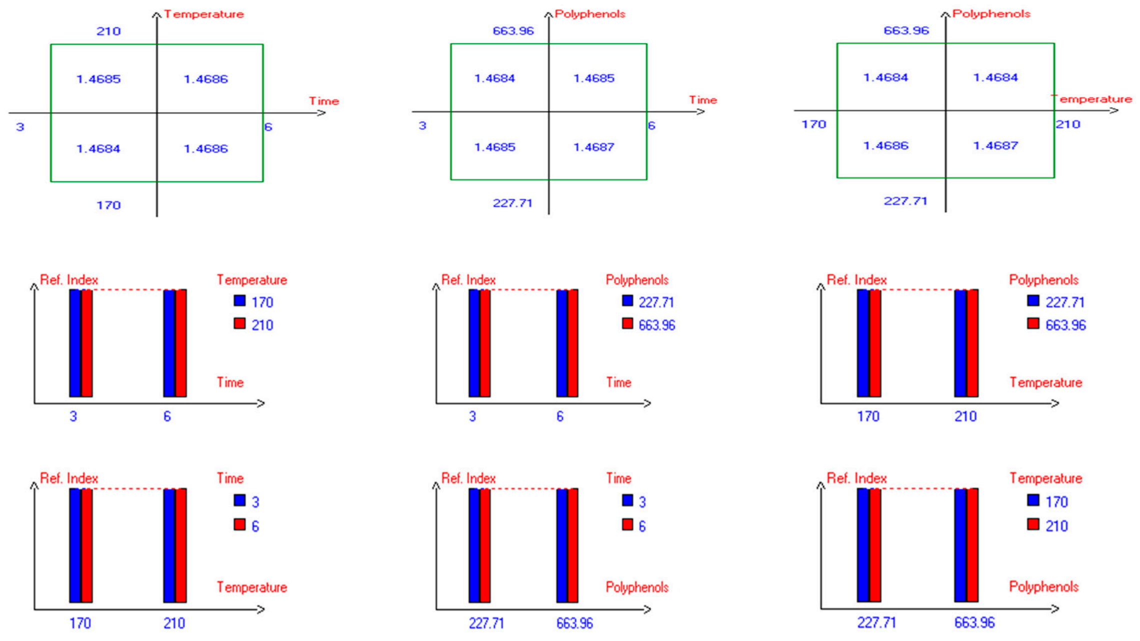

A **B** **C**

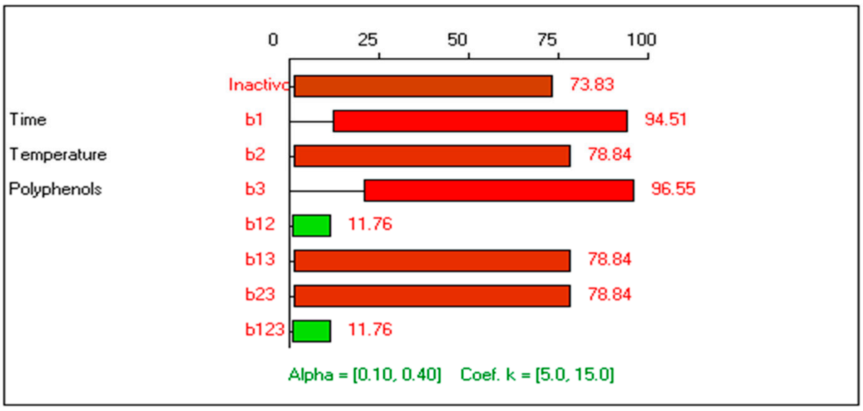

D

Figure S40. Combined interactions between the independent variables on a response variable (**refractive index**) in EVOO **Arbequina** under D-F: (**A**) x_1 and x_2, (**B**) x_1 and x_3, (**C**) x_2 and x_3, and (**D**) results of variance analysis of regression equation model and the significance changes in each individual independent variable and interaction between the combined independent variables on refractive index; b represents a significant difference when be >b_{123}, while b represents no significant difference when be ≤b_{123}; b_1, b_2, b_3 are the main effects of the independent variables, while b_{12}, b_{13}, b_{23}, and b_{123} are the interaction effects of the independent variables. Moreover, x_1, x_2, and x_3 are coded variables (time, temperature, and polyphenols addition, respectively) for the experimental design in D-F process.

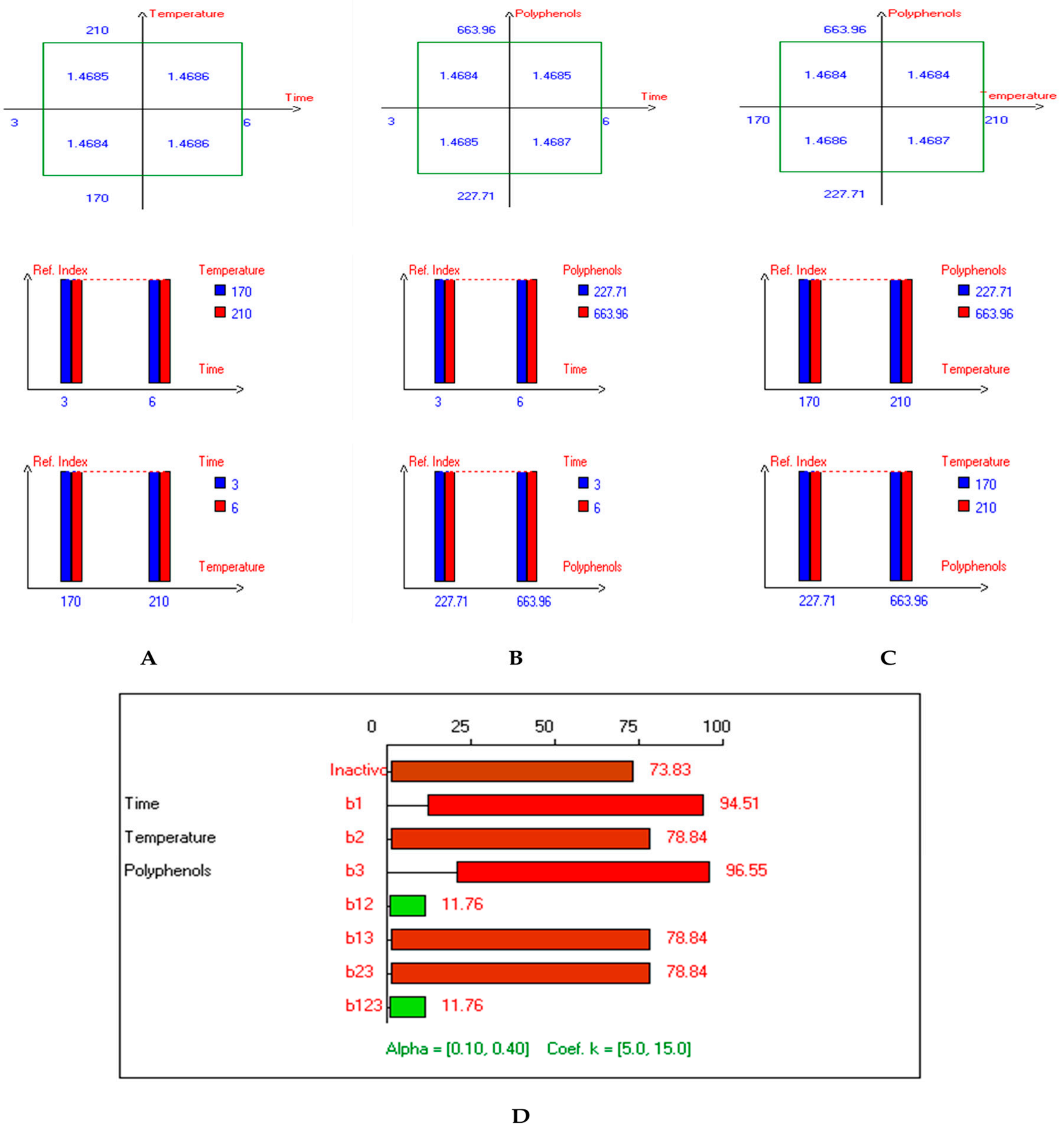

Figure S41. Combined interactions between the independent variables on a response variable (**K_{232}**) in EVOO **Hojiblanca** under D-F: (**A**) x_1 and x_2, (**B**) x_1 and x_3, (**C**) x_2 and x_3, and (**D**) results of variance analysis of regression equation model and the significance changes in each individual independent variable and interaction between the combined independent variables on K_{232}; b represents a significant difference when be $>b_{123}$, while b represents no significant difference when be $\leq b_{123}$; b_1, b_2, b_3 are the main effects of the independent variables, while b_{12}, b_{13}, b_{23}, and b_{123} are the interaction effects of the independent variables. Moreover, x_1, x_2, and x_3 are coded variables (time, temperature, and polyphenols addition, respectively) for the experimental design in D-F process.

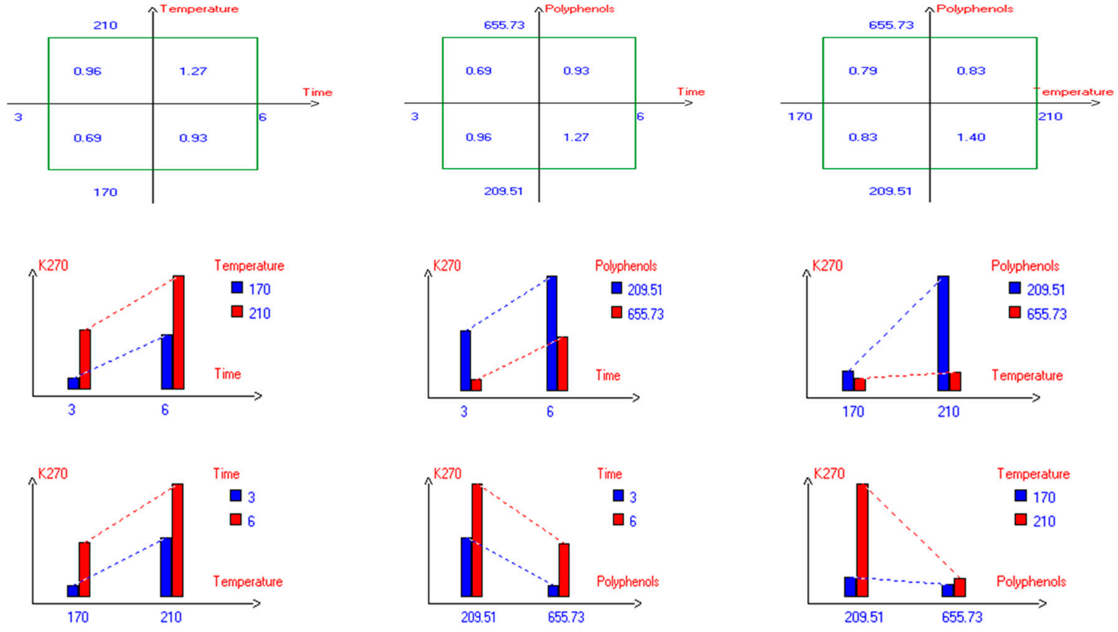

A **B** **C**

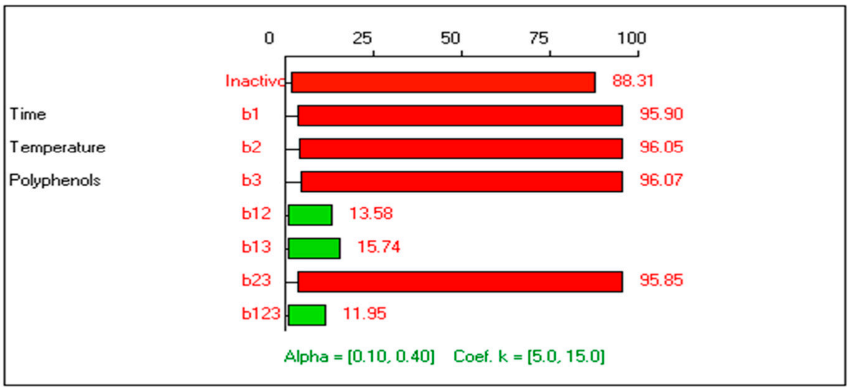

D

Figure S42. Combined interactions between the independent variables on a response variable (**K_{270}**) in EVOO **Hojiblanca** under D-F: (**A**) x_1 and x_2, (**B**) x_1 and x_3, (**C**) x_2 and x_3, and (**D**) results of variance analysis of regression equation model and the significance changes in each individual independent variable and interaction between the combined independent variables on K_{270}; b represents a significant difference when be $>b_{123}$, while b represents no significant difference when be $\leq b_{123}$; b_1, b_2, b_3 are the main effects of the independent variables, while b_{12}, b_{13}, b_{23}, and b_{123} are the interaction effects of the independent variables. Moreover, x_1, x_2, and x_3 are coded variables (time, temperature, and polyphenols addition, respectively) for the experimental design in D-F process.

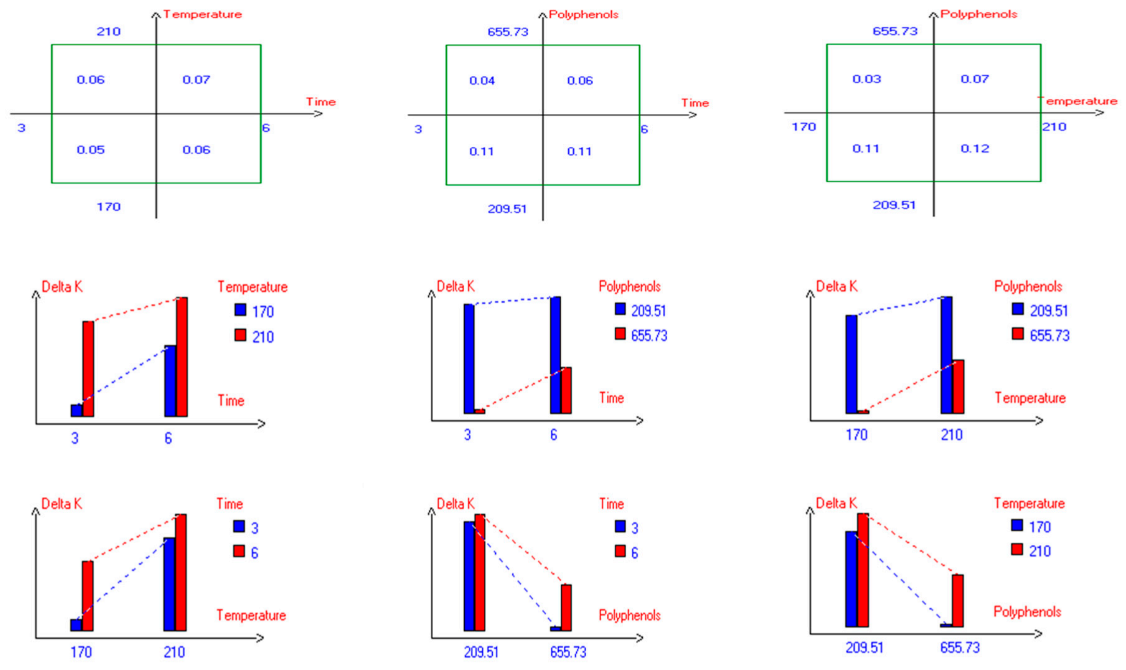

A **B** **C**

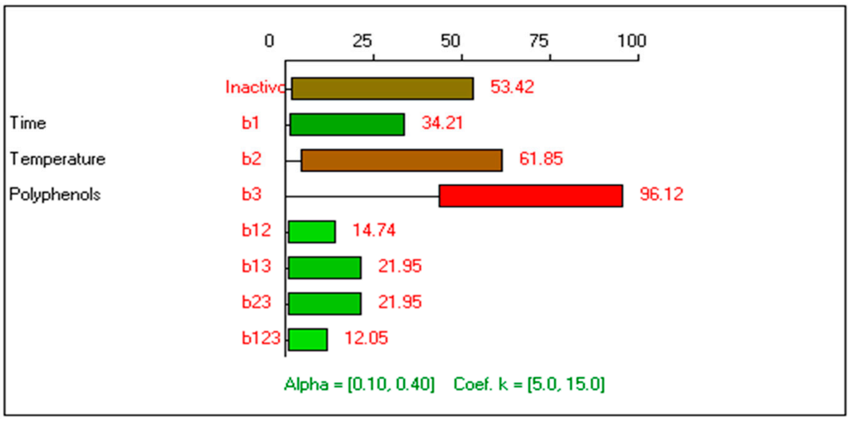

D

Figure S43. Combined interactions between the independent variables on a response variable (**ΔK**) in EVOO **Hojiblanca** under D-F: (**A**) x_1 and x_2, (**B**) x_1 and x_3, (**C**) x_2 and x_3, and (**D**) results of variance analysis of regression equation model and the significance changes in each individual independent variable and interaction between the combined independent variables on ΔK; b represents a significant difference when be $>b_{123}$, while b represents no significant difference when be $\leq b_{123}$; b_1, b_2, b_3 are the main effects of the independent variables, while b_{12}, b_{13}, b_{23}, and b_{123} are the interaction effects of the independent variables. Moreover, x_1, x_2, and x_3 are coded variables (time, temperature, and polyphenols addition, respectively) for the experimental design in D-F process.

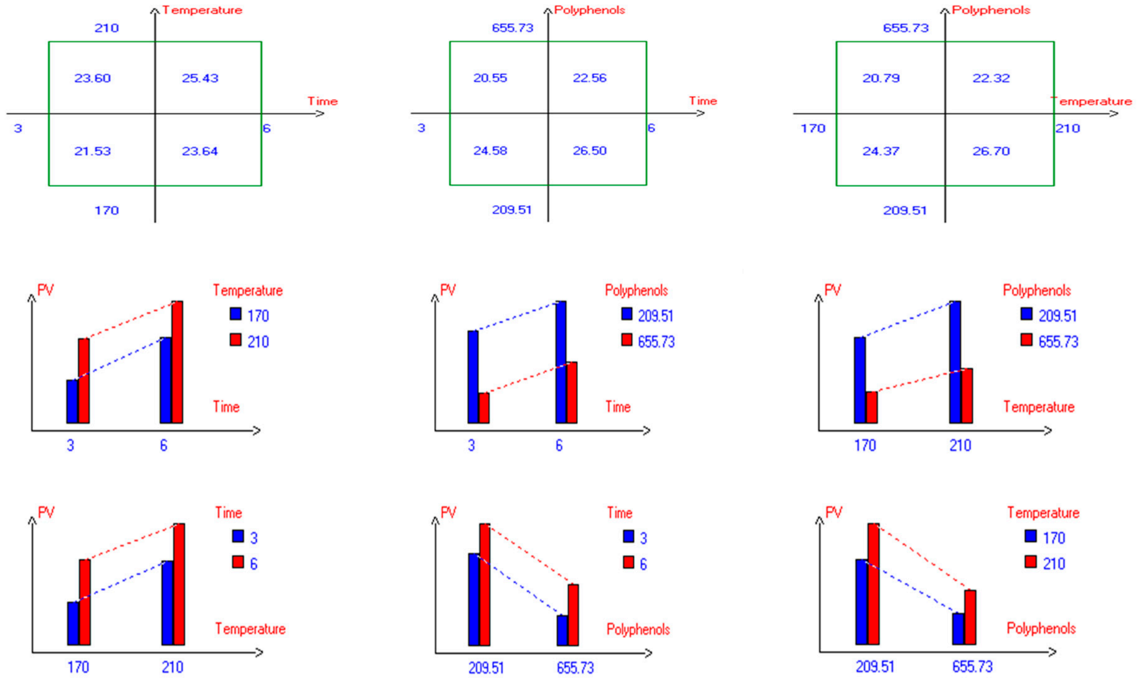

A **B** **C**

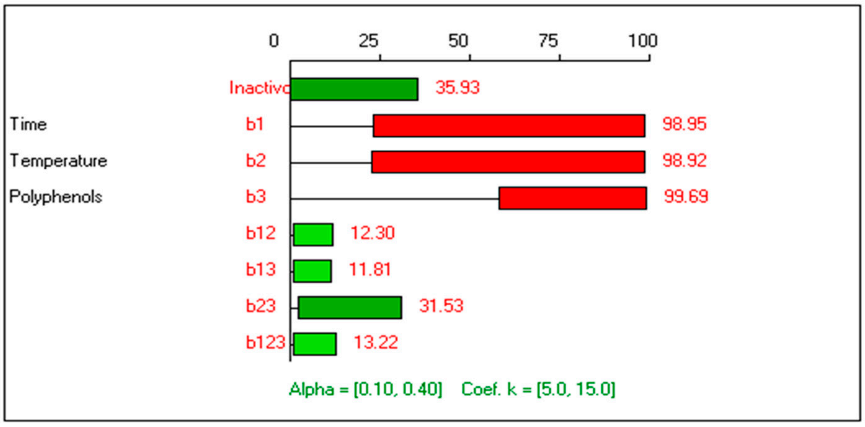

D

Figure S44. Combined interactions between the independent variables on a response variable (**peroxide value** (mEqO$_2$/kg)) in EVOO **Hojiblanca** under D-F: (**A**) x_1 and x_2, (**B**) x_1 and x_3, (**C**) x_2 and x_3, and (**D**) results of variance analysis of regression equation model and the significance changes in each individual independent variable and interaction between the combined independent variables on peroxide value; b represents a significant difference when be >b_{123}, while b represents no significant difference when be ≤b_{123}; b_1, b_2, b_3 are the main effects of the independent variables, while b_{12}, b_{13}, b_{23}, and b_{123} are the interaction effects of the independent variables. Moreover, x_1, x_2, and x_3 are coded variables (time, temperature, and polyphenols addition, respectively) for the experimental design in D-F process.

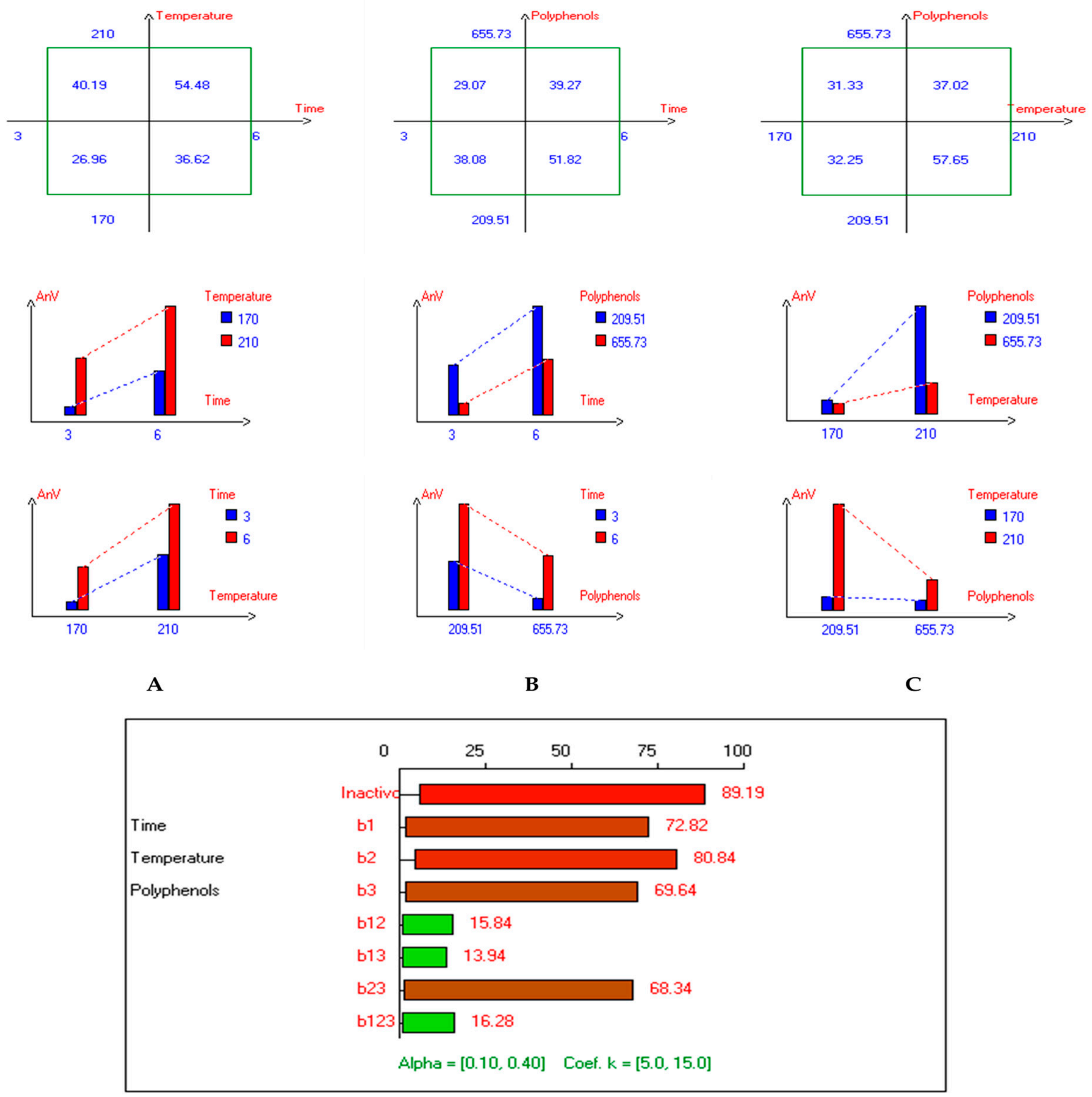

D

Figure S45. Combined interactions between the independent variables on a response variable (**anisidine value** (mg/kg)) in EVOO **Hojiblanca** under D-F: (**A**) x_1 and x_2, (**B**) x_1 and x_3, (**C**) x_2 and x_3, and (**D**) results of variance analysis of regression equation model and the significance changes in each individual independent variable and interaction between the combined independent variables on anisidine value; b represents a significant difference when be $>b_{123}$, while b represents no significant difference when be $\leq b_{123}$; b_1, b_2, b_3 are the main effects of the independent variables, while b_{12}, b_{13}, b_{23}, and b_{123} are the interaction effects of the independent variables. Moreover, x_1, x_2, and x_3 are coded variables (time, temperature, and polyphenols addition, respectively) for the experimental design in D-F process.

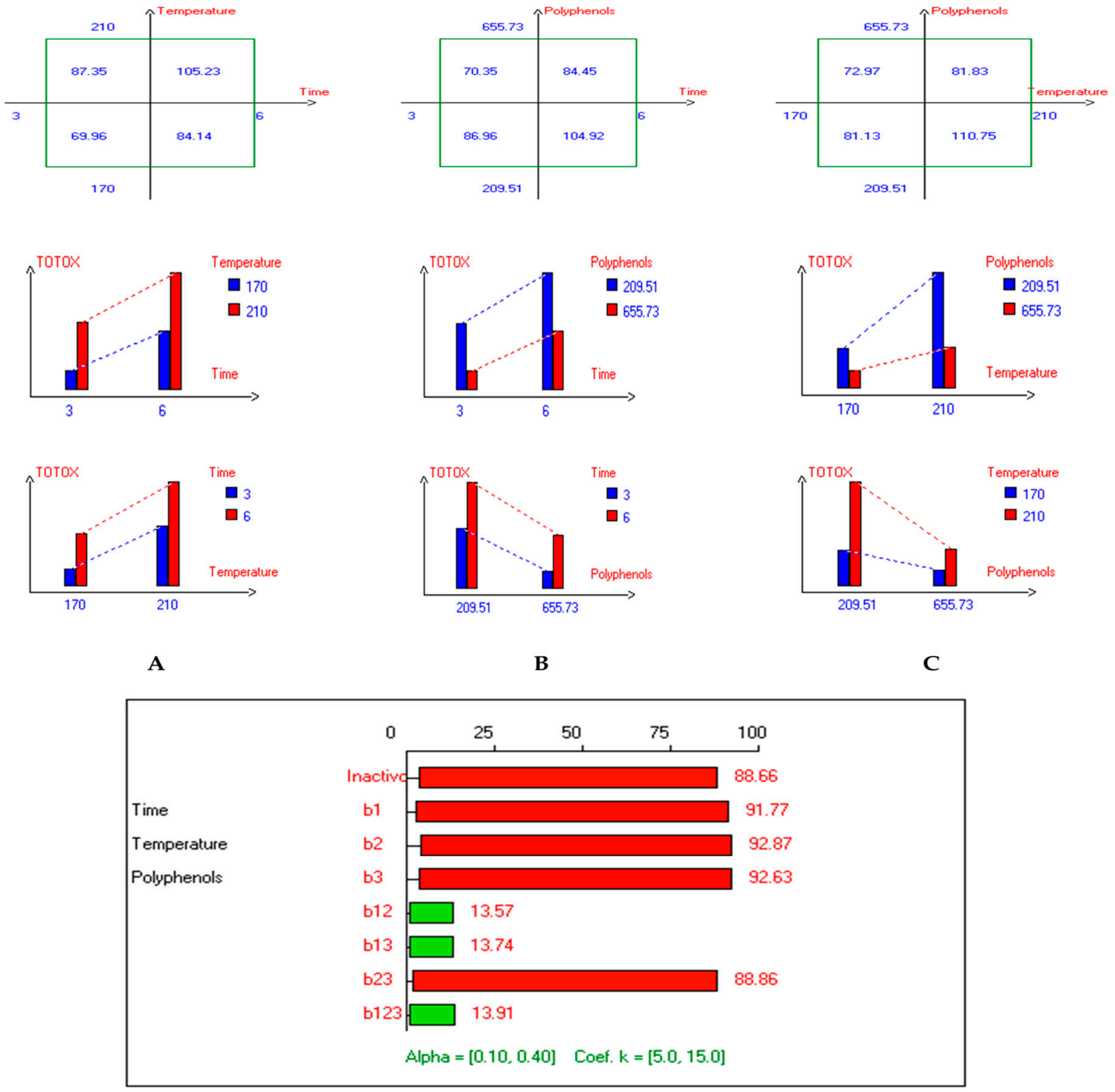

D

Figure S46. Combined interactions between the independent variables on a response variable (**TOTOX**) in EVOO **Hojiblanca** under D-F: (**A**) x_1 and x_2, (**B**) x_1 and x_3, (**C**) x_2 and x_3, and (**D**) results of variance analysis of regression equation model and the significance changes in each individual independent variable and interaction between the combined independent variables on TOTOX; b represents a significant difference when be $>b_{123}$, while b represents no significant difference when be $\leq b_{123}$; b_1, b_2, b_3 are the main effects of the independent variables, while b_{12}, b_{13}, b_{23}, and b_{123} are the interaction effects of the independent variables. Moreover, x_1, x_2, and x_3 are coded variables (time, temperature, and polyphenols addition, respectively) for the experimental design in D-F process.

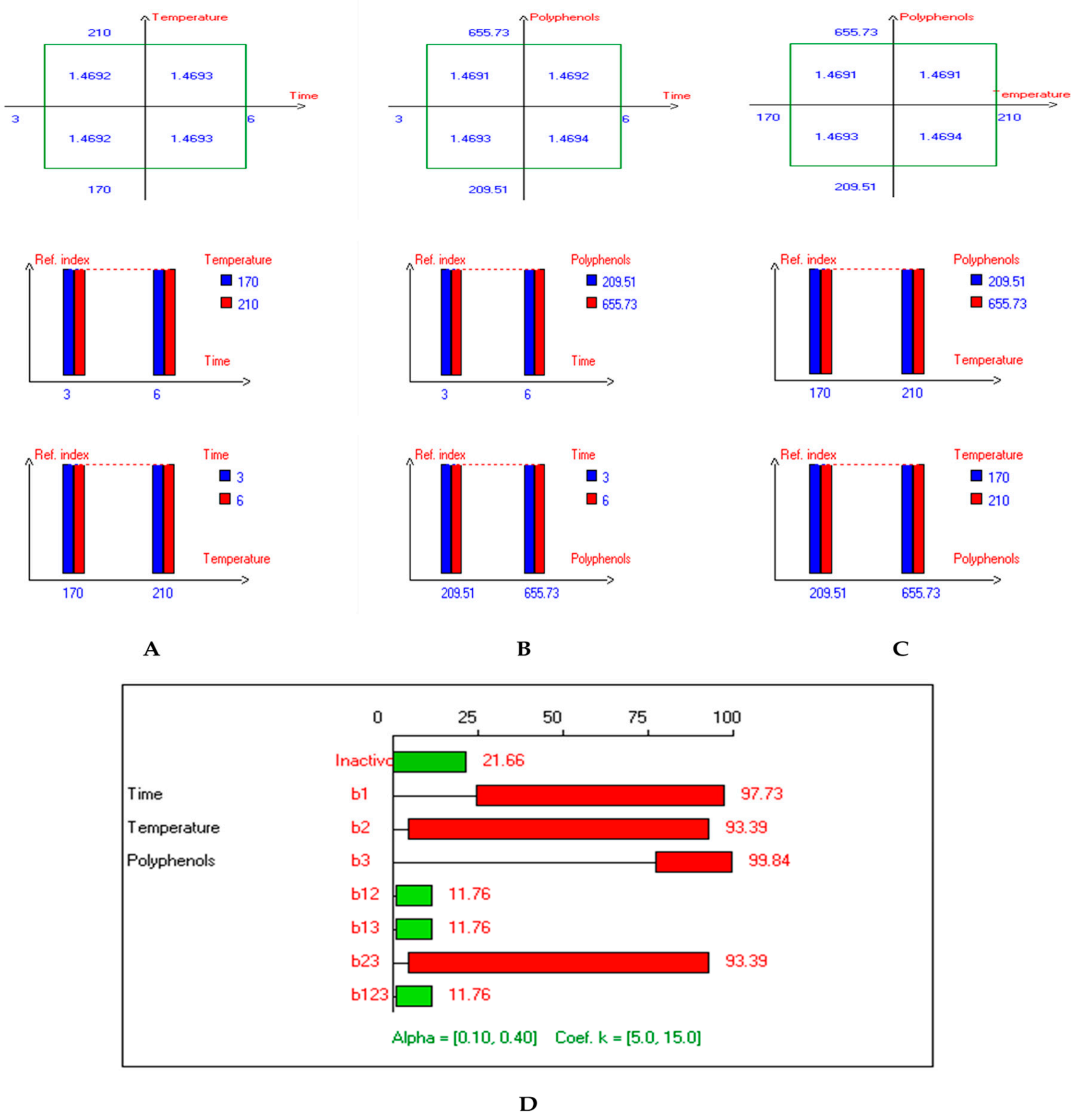

Figure S47. Combined interactions between the independent variables on a response variable (**refractive index**) in EVOO **Hojiblanca** under D-F: (**A**) x_1 and x_2, (**B**) x_1 and x_3, (**C**) x_2 and x_3, and (**D**) results of variance analysis of regression equation model and the significance changes in each individual independent variable and interaction between the combined independent variables on refractive index; b represents a significant difference when be $>b_{123}$, while b represents no significant difference when be $\leq b_{123}$; b_1, b_2, b_3 are the main effects of the independent variables, while b_{12}, b_{13}, b_{23}, and b_{123} are the interaction effects of the independent variables. Moreover, x_1, x_2, and x_3 are coded variables (time, temperature, and polyphenols addition, respectively) for the experimental design in D-F process.

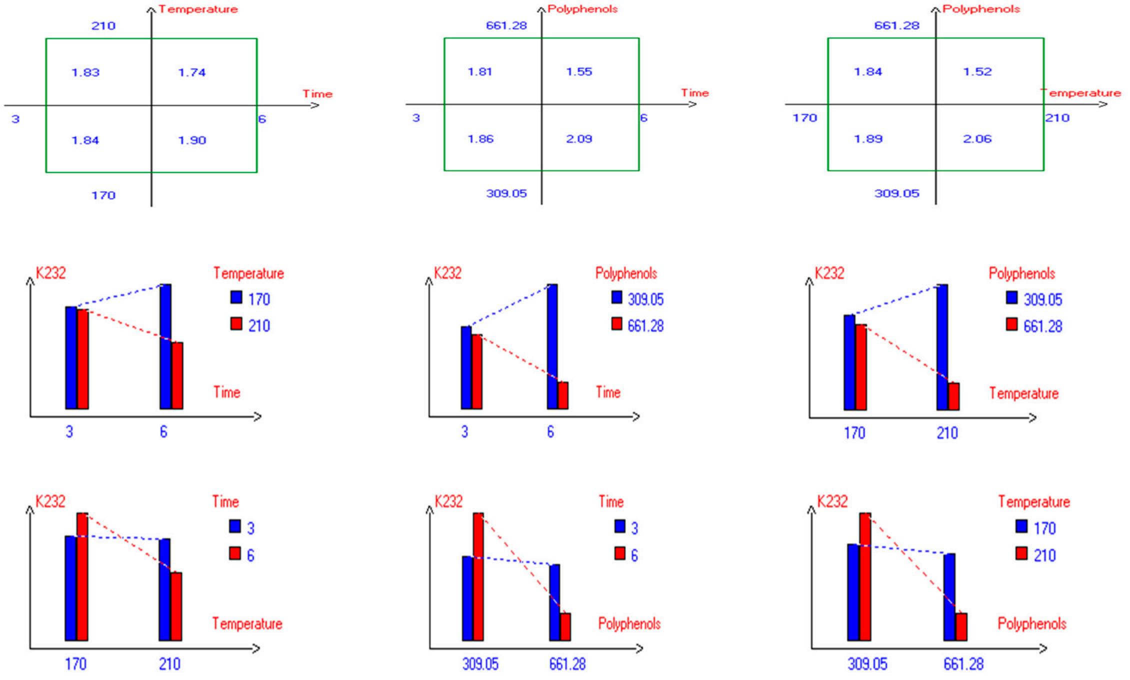

A B C

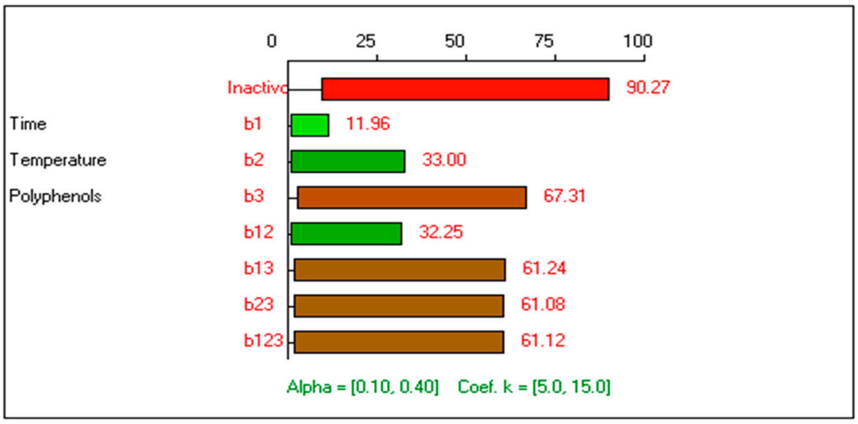

D

Figure S48. Combined interactions between the independent variables on a response variable (**K_{232}**) in EVOO **Manzanilla** under D-F: (**A**) x_1 and x_2, (**B**) x_1 and x_3, (**C**) x_2 and x_3, and (**D**) results of variance analysis of regression equation model and the significance changes in each individual independent variable and interaction between the combined independent variables on K_{232}; b represents a significant difference when be $>b_{123}$, while b represents no significant difference when be $\leq b_{123}$; b_1, b_2, b_3 are the main effects of the independent variables, while b_{12}, b_{13}, b_{23}, and b_{123} are the interaction effects of the independent variables. Moreover, x_1, x_2, and x_3 are coded variables (time, temperature, and polyphenols addition, respectively) for the experimental design in D-F process.

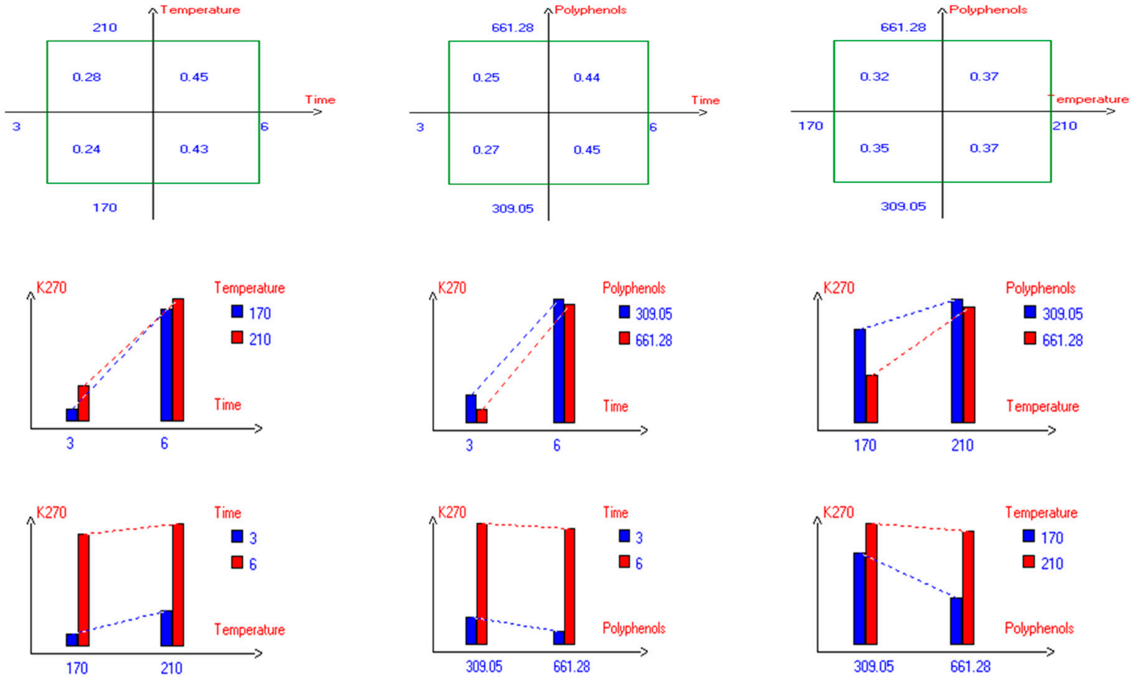

A **B** **C**

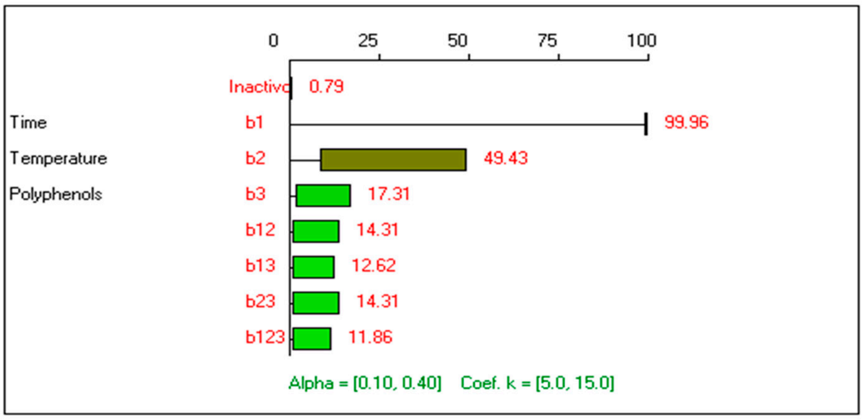

D

Figure S49. Combined interactions between the independent variables on a response variable (**K_{270}**) in EVOO **Manzanilla** under D-F: (**A**) x_1 and x_2, (**B**) x_1 and x_3, (**C**) x_2 and x_3, and (**D**) results of variance analysis of regression equation model and the significance changes in each individual independent variable and interaction between the combined independent variables on K_{270}; b represents a significant difference when be $>b_{123}$, while b represents no significant difference when be $\leq b_{123}$; b_1, b_2, b_3 are the main effects of the independent variables, while b_{12}, b_{13}, b_{23}, and b_{123} are the interaction effects of the independent variables. Moreover, x_1, x_2, and x_3 are coded variables (time, temperature, and polyphenols addition, respectively) for the experimental design in D-F process.

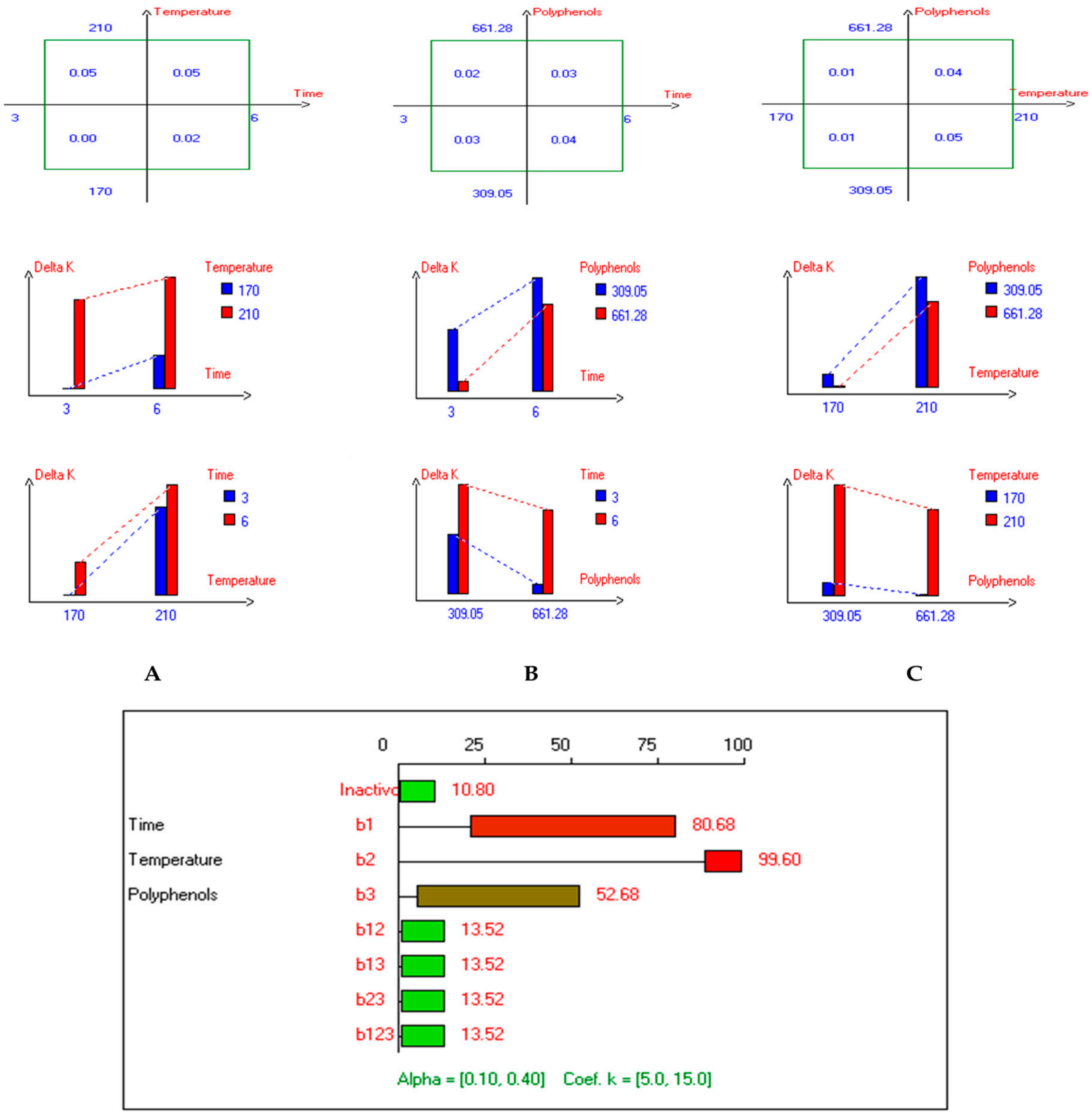

D

Figure S50. Combined interactions between the independent variables on a response variable (ΔK) in EVOO **Manzanilla** under D-F: (**A**) x_1 and x_2, (**B**) x_1 and x_3, (**C**) x_2 and x_3, and (**D**) results of variance analysis of regression equation model and the significance changes in each individual independent variable and interaction between the combined independent variables on ΔK; b represents a significant difference when be $>b_{123}$, while b represents no significant difference when be $\leq b_{123}$; b_1, b_2, b_3 are the main effects of the independent variables, while b_{12}, b_{13}, b_{23}, and b_{123} are the interaction effects of the independent variables. Moreover, x_1, x_2, and x_3 are coded variables (time, temperature, and polyphenols addition, respectively) for the experimental design in D-F process.

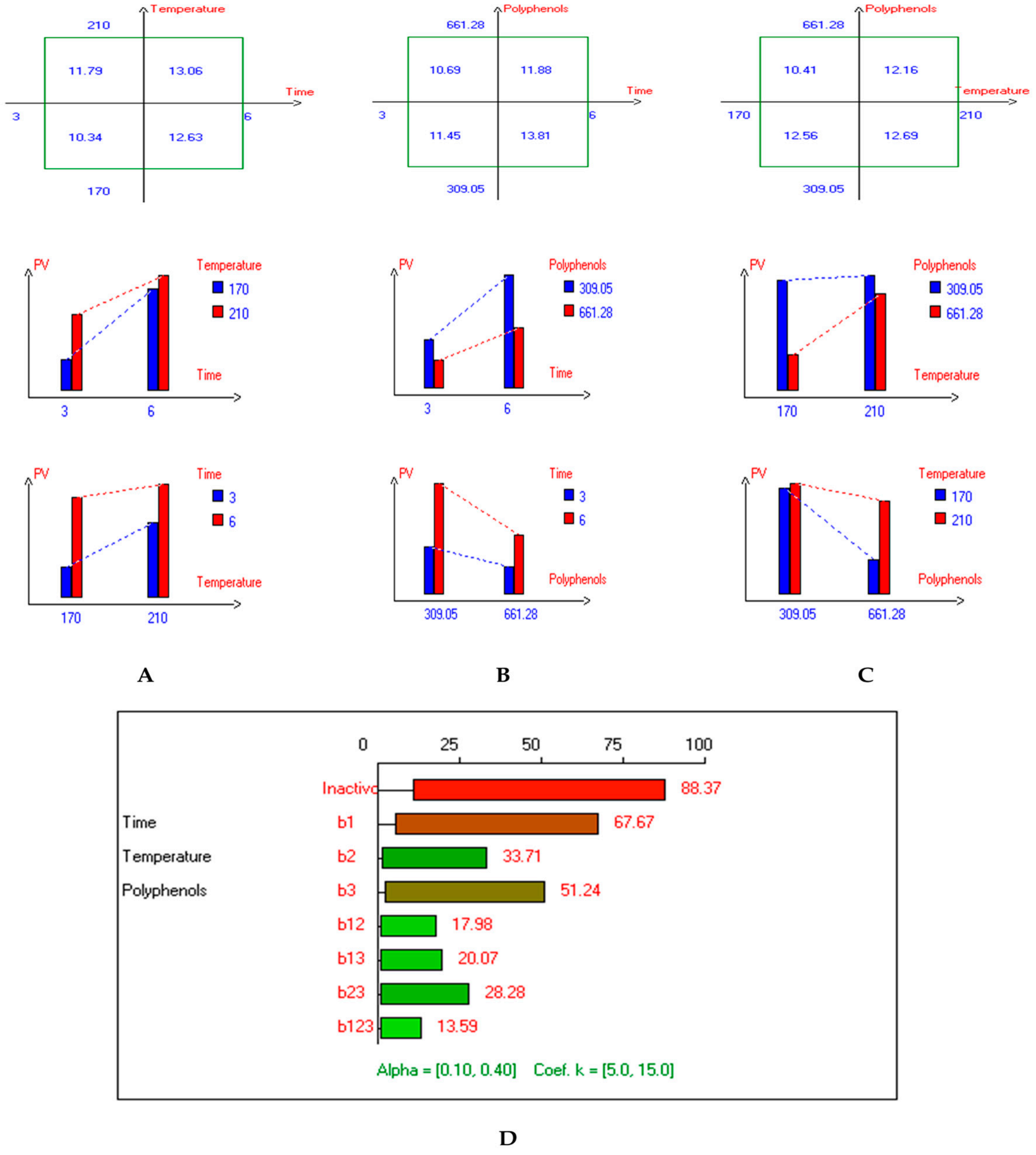

Figure S51. Combined interactions between the independent variables on a response variable (**peroxide value** (mEqO_2/kg)) in EVOO **Manzanilla** under D-F: (**A**) x_1 and x_2, (**B**) x_1 and x_3, (**C**) x_2 and x_3, and (**D**) results of variance analysis of regression equation model and the significance changes in each individual independent variable and interaction between the combined independent variables on peroxide value; b represents a significant difference when be >b_{123}, while b represents no significant difference when be ≤b_{123}; b_1, b_2, b_3 are the main effects of the independent variables, while b_{12}, b_{13}, b_{23}, and b_{123} are the interaction effects of the independent variables. Moreover, x_l, x_2, and x_3 are coded variables (time, temperature, and polyphenols addition, respectively) for the experimental design in D-F process.

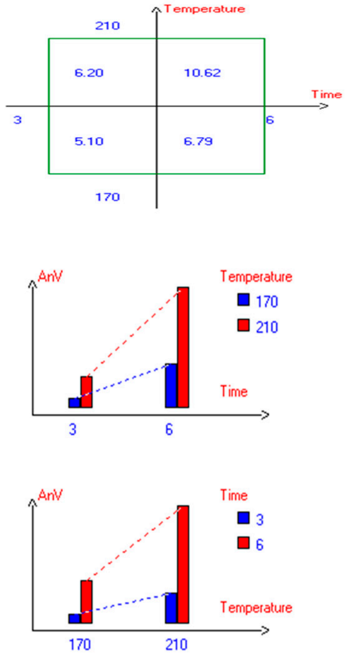

A

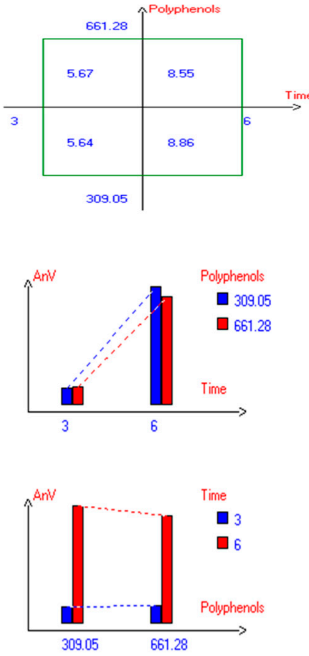

B

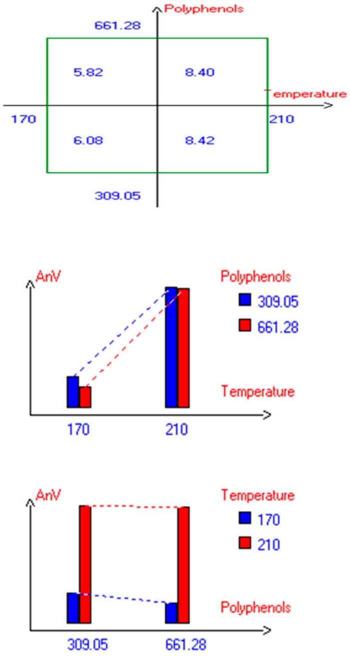

C

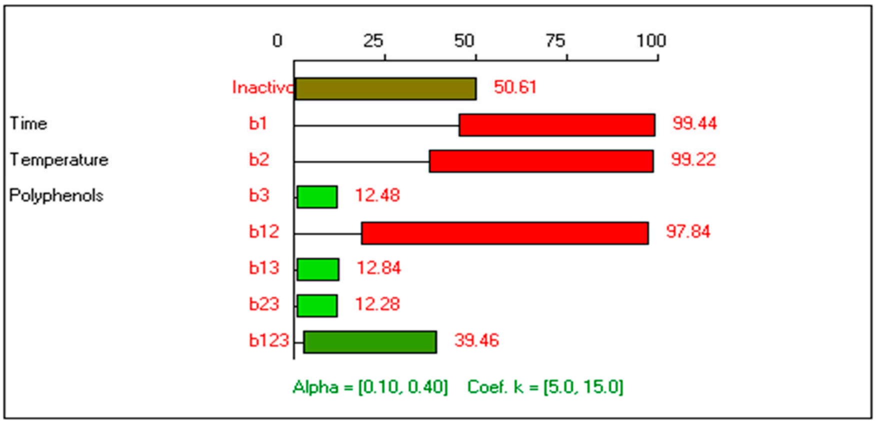

D

Figure S52. Combined interactions between the independent variables on a response variable (**anisidine value** (mg/kg)) in EVOO **Manzanilla** under D-F: (**A**) x_1 and x_2, (**B**) x_1 and x_3, (**C**) x_2 and x_3, and (**D**) results of variance analysis of regression equation model and the significance changes in each individual independent variable and interaction between the combined independent variables on anisidine value; b represents a significant difference when be $>b_{123}$, while b represents no significant difference when be $\leq b_{123}$; b_1, b_2, b_3 are the main effects of the independent variables, while b_{12}, b_{13}, b_{23}, and b_{123} are the interaction effects of the independent variables. Moreover, x_1, x_2, and x_3 are coded variables (time, temperature, and polyphenols addition, respectively) for the experimental design in D-F process.

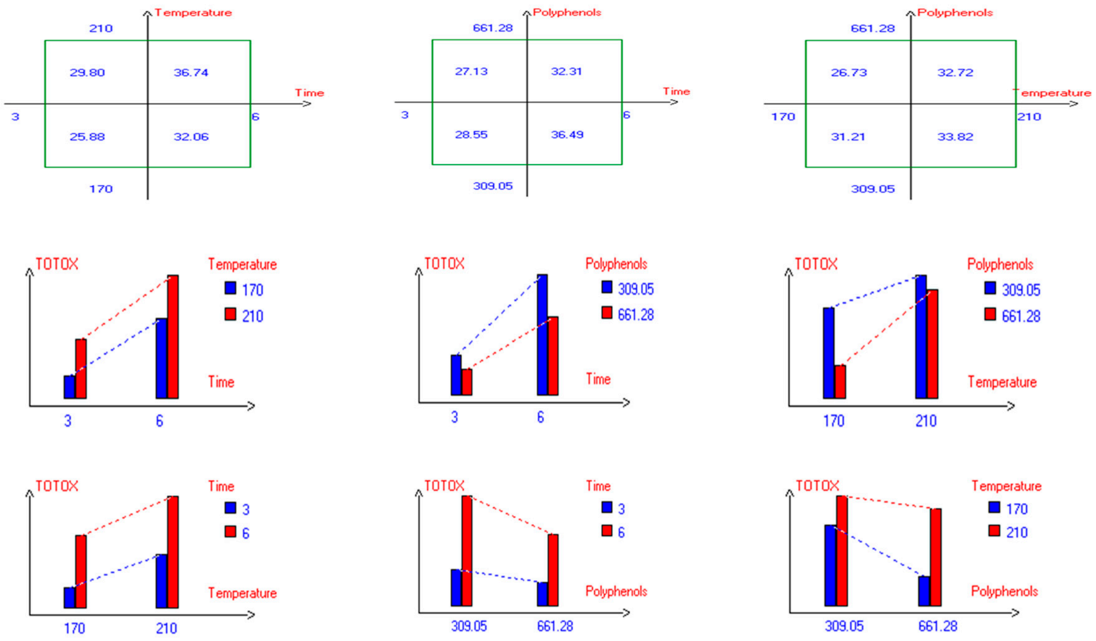

A B C

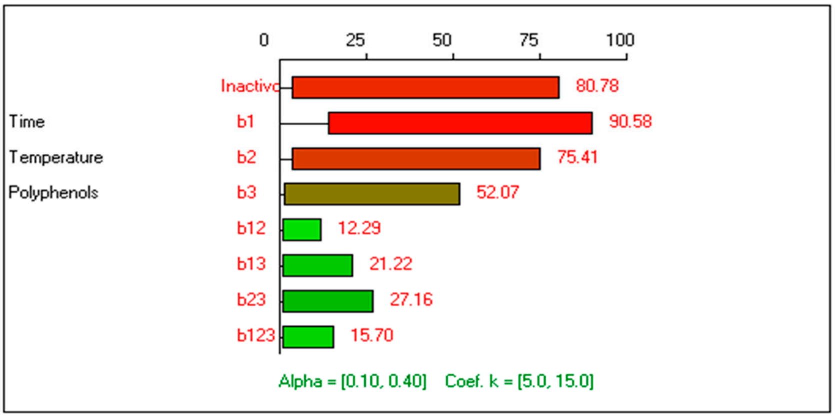

D

Figure S53. Combined interactions between the independent variables on a response variable (**TOTOX**) in EVOO **Manzanilla** under D-F: (**A**) x_1 and x_2, (**B**) x_1 and x_3, (**C**) x_2 and x_3, and (**D**) results of variance analysis of regression equation model and the significance changes in each individual independent variable and interaction between the combined independent variables on TOTOX; b represents a significant difference when be $>b_{123}$, while b represents no significant difference when be $\leq b_{123}$; b_1, b_2, b_3 are the main effects of the independent variables, while b_{12}, b_{13}, b_{23}, and b_{123} are the interaction effects of the independent variables. Moreover, x_1, x_2, and x_3 are coded variables (time, temperature, and polyphenols addition, respectively) for the experimental design in D-F process.

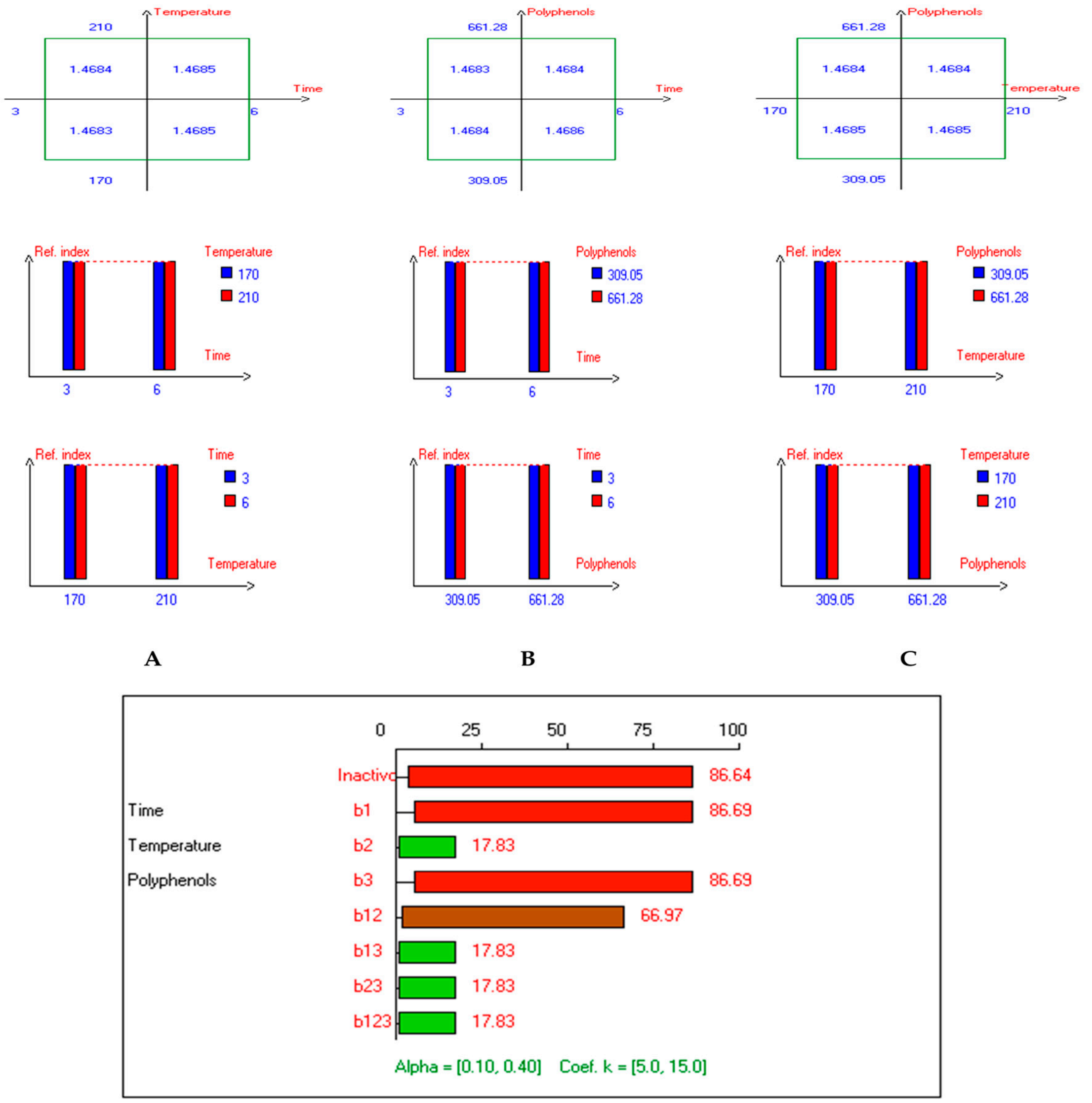

D

Figure S54. Combined interactions between the independent variables on a response variable (**refractive index**) in EVOO **Manzanilla** under D-F: (**A**) x_1 and x_2, (**B**) x_1 and x_3, (**C**) x_2 and x_3, and (**D**) results of variance analysis of regression equation model and the significance changes in each individual independent variable and interaction between the combined independent variables on refractive index; b represents a significant difference when be $>b_{123}$, while b represents no significant difference when be $\leq b_{123}$; b_1, b_2, b_3 are the main effects of the independent variables, while b_{12}, b_{13}, b_{23}, and b_{123} are the interaction effects of the independent variables. Moreover, x_1, x_2, and x_3 are coded variables (time, temperature, and polyphenols addition, respectively) for the experimental design in D-F process.

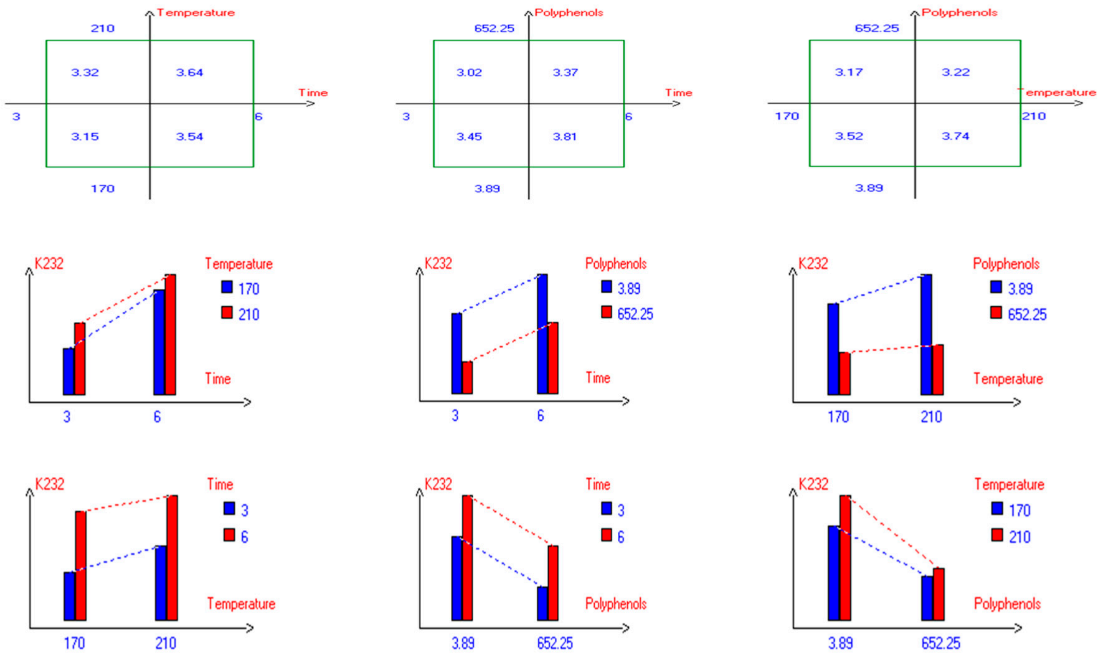

A **B** **C**

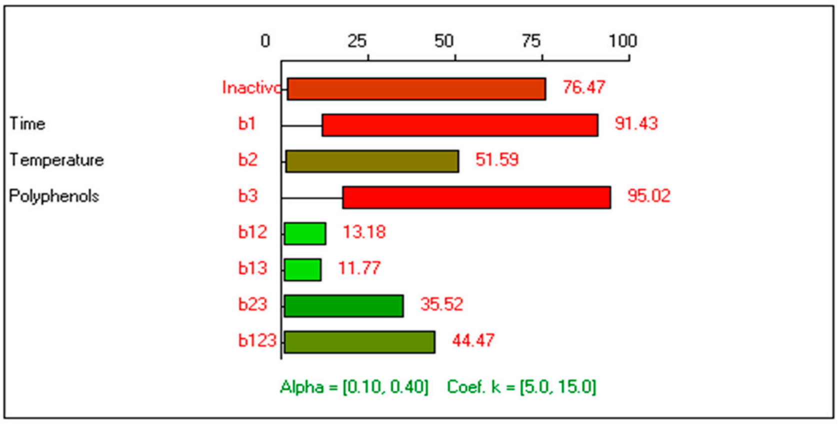

D

Figure S55. Combined interactions between the independent variables on a response variable (**K_{232}**) in **Pomace** olive oil under D-F: (**A**) x_1 and x_2, (**B**) x_1 and x_3, (**C**) x_2 and x_3, and (**D**) results of variance analysis of regression equation model and the significance changes in each individual independent variable and interaction between the combined independent variables on K_{232}; b represents a significant difference when be $>b_{123}$, while b represents no significant difference when be $\leq b_{123}$; b_1, b_2, b_3 are the main effects of the independent variables, while b_{12}, b_{13}, b_{23}, and b_{123} are the interaction effects of the independent variables. Moreover, x_1, x_2, and x_3 are coded variables (time, temperature, and polyphenols addition, respectively) for the experimental design in D-F process.

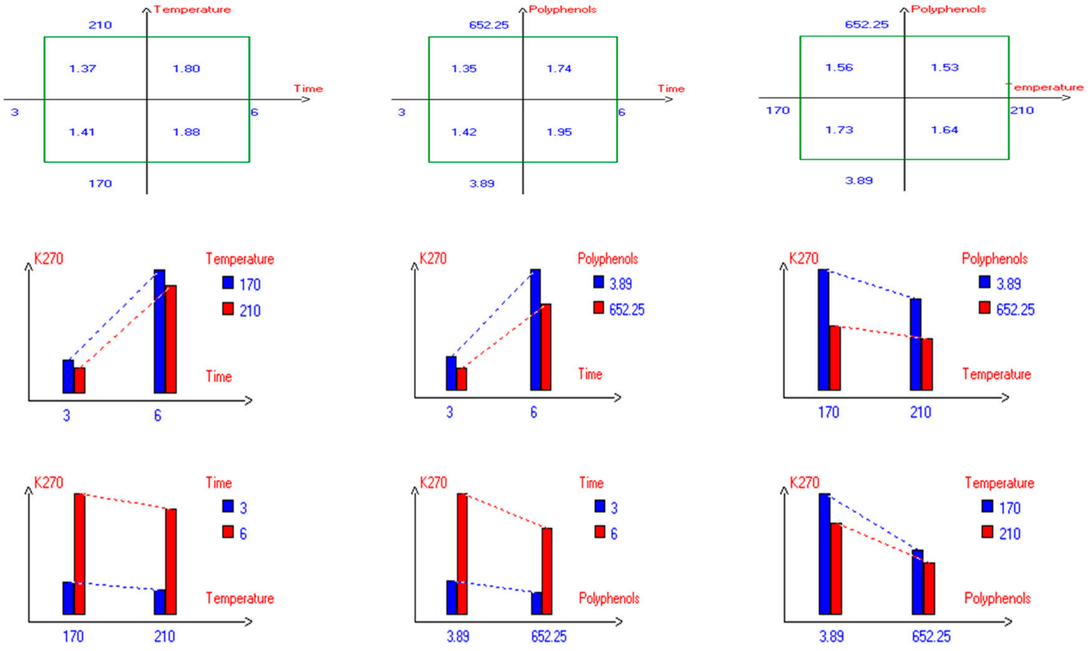

A **B** **C**

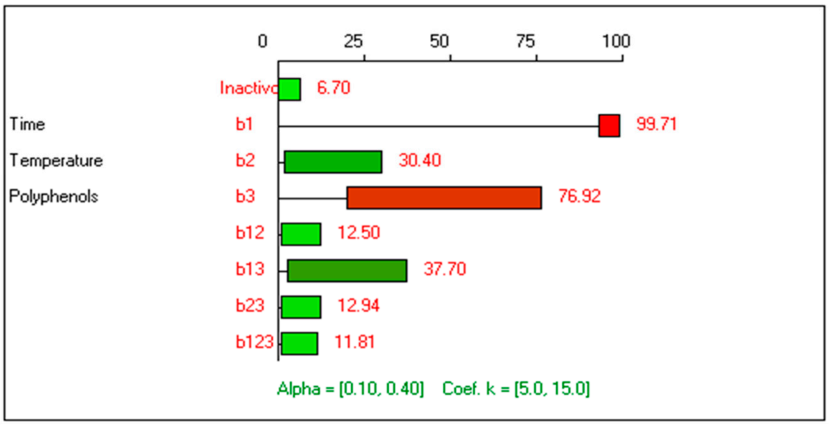

D

Figure S56. Combined interactions between the independent variables on a response variable (**K270**) in **Pomace** olive oil under D-F: (**A**) x_1 and x_2, (**B**) x_1 and x_3, (**C**) x_2 and x_3, and (**D**) results of variance analysis of regression equation model and the significance changes in each individual independent variable and interaction between the combined independent variables on K270; b represents a significant difference when be >b_{123}, while b represents no significant difference when be ≤b_{123}; b_1, b_2, b_3 are the main effects of the independent variables, while b_{12}, b_{13}, b_{23}, and b_{123} are the interaction effects of the independent variables. Moreover, x_1, x_2, and x_3 are coded variables (time, temperature, and polyphenols addition, respectively) for the experimental design in D-F process.

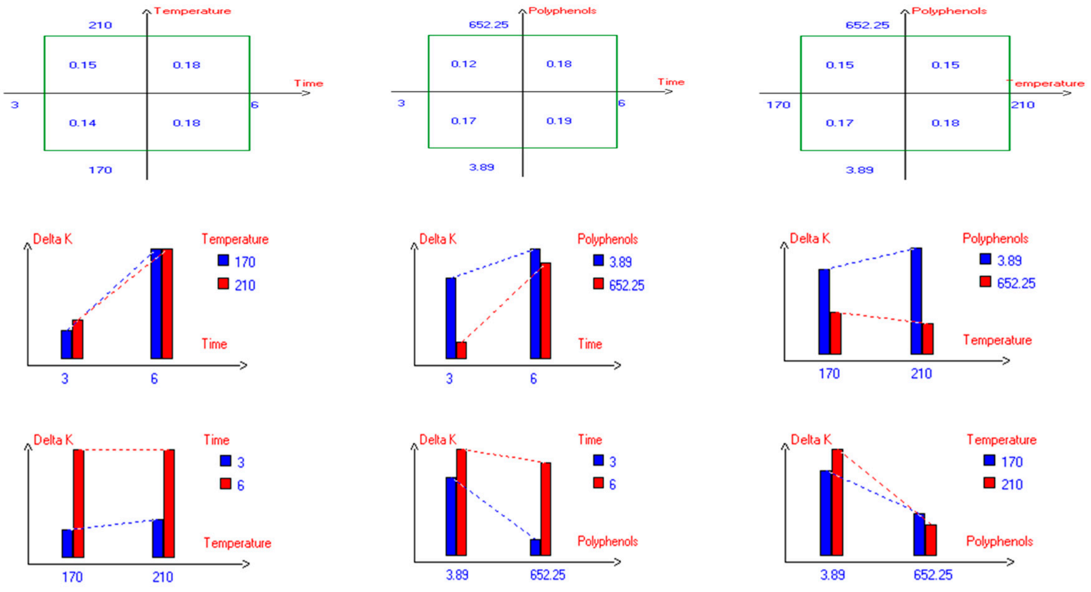

A B C

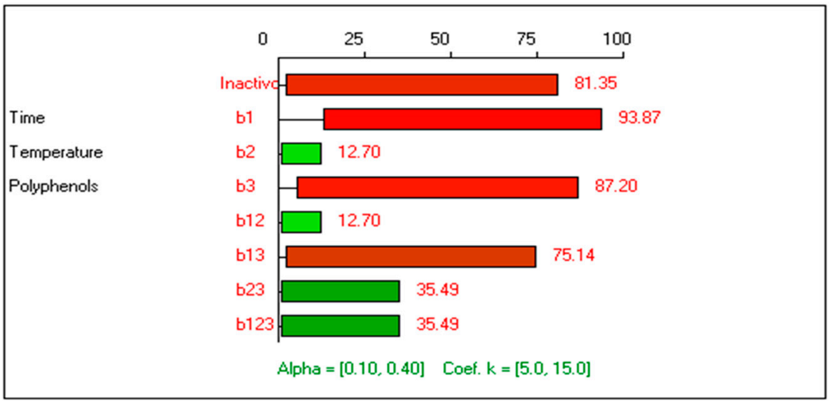

D

Figure S57. Combined interactions between the independent variables on a response variable (**ΔK**) in **Pomace** olive oil under D-F: (**A**) x_1 and x_2, (**B**) x_1 and x_3, (**C**) x_2 and x_3, and (**D**) results of variance analysis of regression equation model and the significance changes in each individual independent variable and interaction between the combined independent variables on ΔK; b represents a significant difference when be >b_{123}, while b represents no significant difference when be ≤b_{123}; b_1, b_2, b_3 are the main effects of the independent variables, while b_{12}, b_{13}, b_{23}, and b_{123} are the interaction effects of the independent variables. Moreover, x_l, x_2, and x_3 are coded variables (time, temperature, and polyphenols addition, respectively) for the experimental design in D-F process.

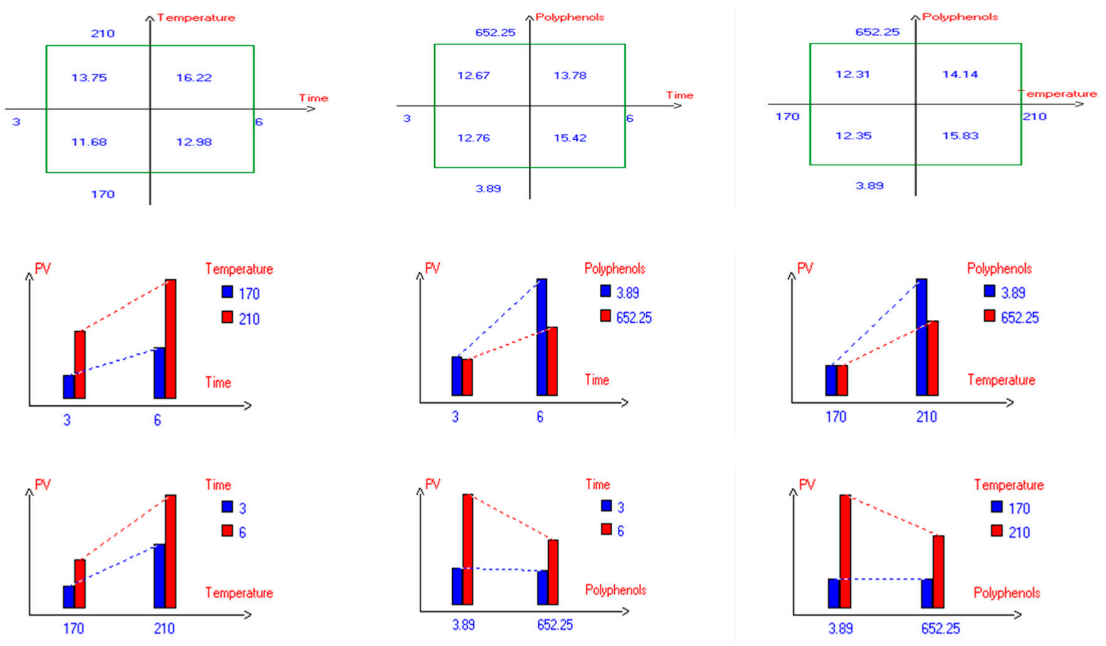

A **B** **C**

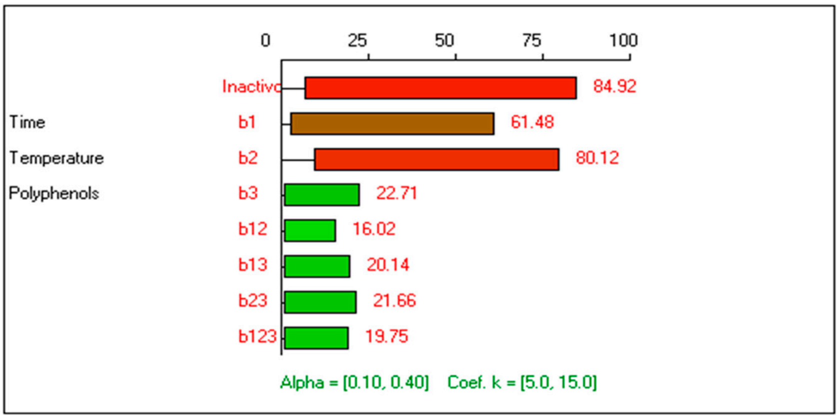

D

Figure S58. Combined interactions between the independent variables on a response variable (**peroxide value** ($mEqO_2/kg$)) in **Pomace** olive oil under D-F: (**A**) x_1 and x_2, (**B**) x_1 and x_3, (**C**) x_2 and x_3, and (**D**) results of variance analysis of regression equation model and the significance changes in each individual independent variable and interaction between the combined independent variables on peroxide value; b represents a significant difference when be $>b_{123}$, while b represents no significant difference when be $\leq b_{123}$; b_1, b_2, b_3 are the main effects of the independent variables, while b_{12}, b_{13}, b_{23}, and b_{123} are the interaction effects of the independent variables. Moreover, x_1, x_2, and x_3 are coded variables (time, temperature, and polyphenols addition, respectively) for the experimental design in D-F process.

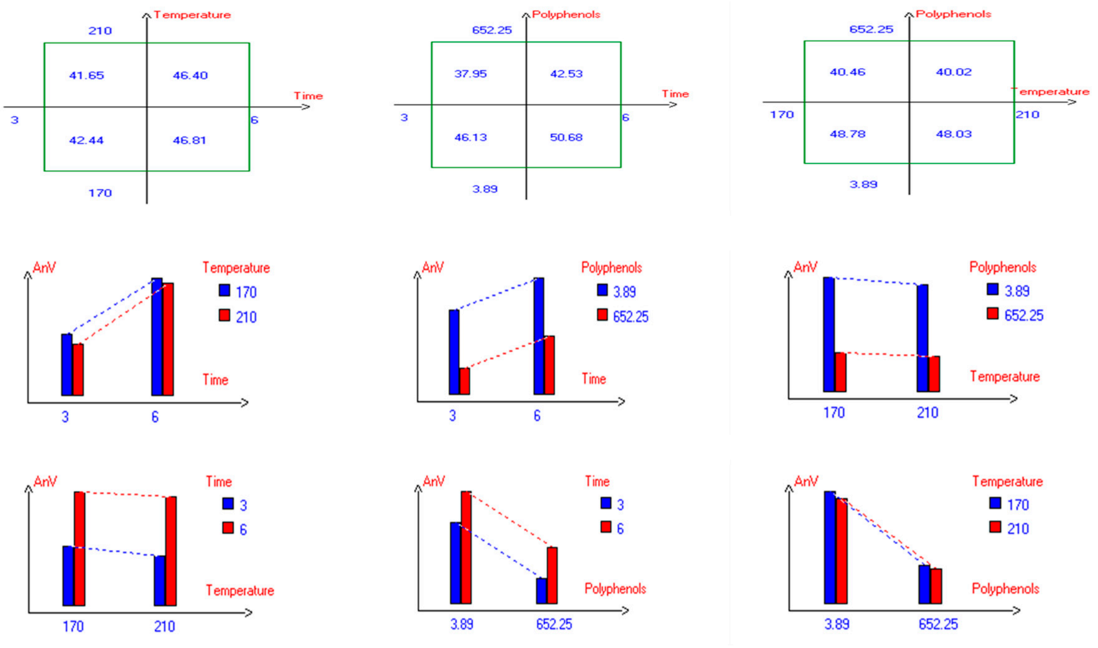

A **B** **C**

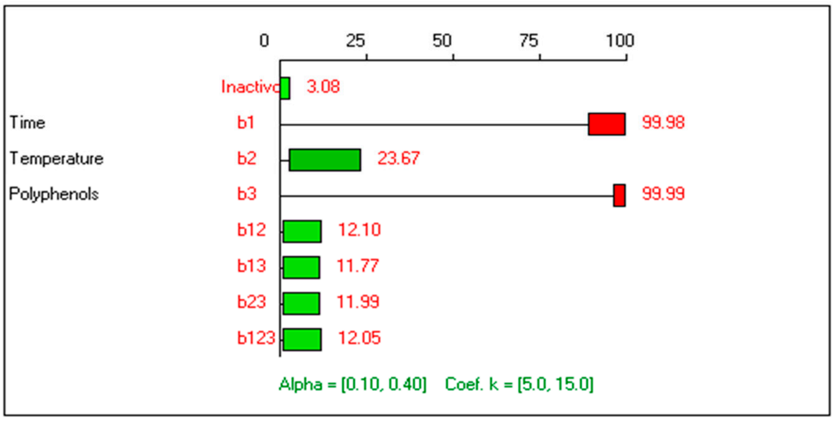

D

Figure S59. Combined interactions between the independent variables on a response variable (**anisidine value** (mg/kg)) in **Pomace** olive oil under D-F: (**A**) x_1 and x_2, (**B**) x_1 and x_3, (**C**) x_2 and x_3, and (**D**) results of variance analysis of regression equation model and the significance changes in each individual independent variable and interaction between the combined independent variables on anisidine value; b represents a significant difference when be $>b_{123}$, while b represents no significant difference when be $\leq b_{123}$; b_1, b_2, b_3 are the main effects of the independent variables, while b_{12}, b_{13}, b_{23}, and b_{123} are the interaction effects of the independent variables. Moreover, x_1, x_2, and x_3 are coded variables (time, temperature, and polyphenols addition, respectively) for the experimental design in D-F process.

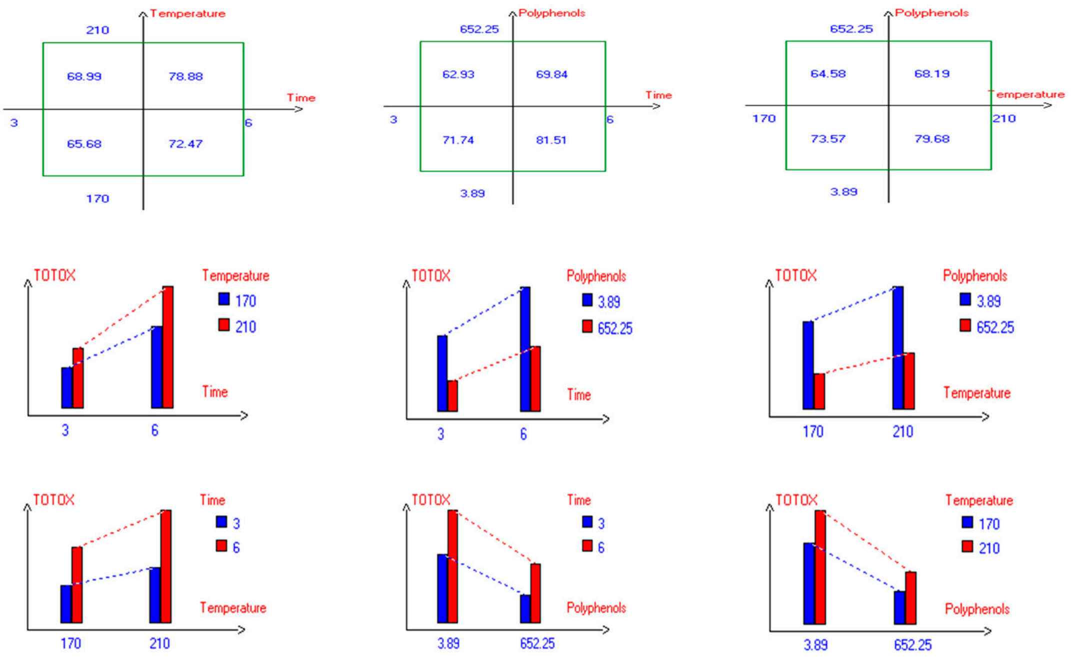

A **B** **C**

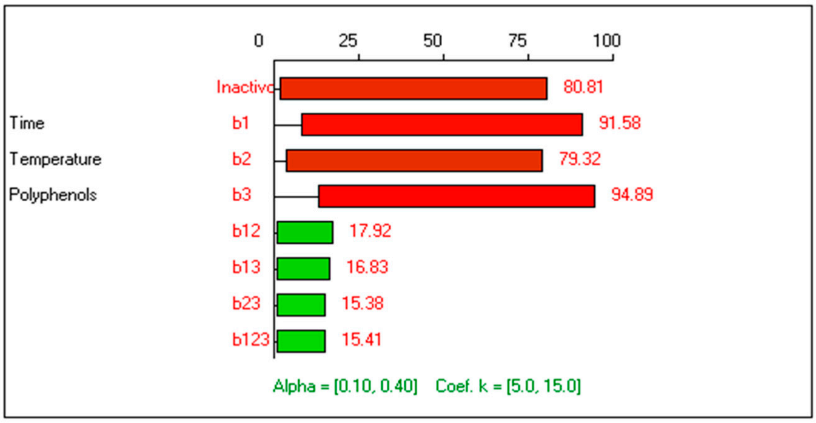

D

Figure S60. Combined interactions between the independent variables on a response variable (**TOTOX**) in **Pomace** olive oil under D-F: (**A**) x_1 and x_2, (**B**) x_1 and x_3, (**C**) x_2 and x_3, and (**D**) results of variance analysis of regression equation model and the significance changes in each individual independent variable and interaction between the combined independent variables on TOTOX; b represents a significant difference when be $>b_{123}$, while b represents no significant difference when be $\leq b_{123}$; b_1, b_2, b_3 are the main effects of the independent variables, while b_{12}, b_{13}, b_{23}, and b_{123} are the interaction effects of the independent variables. Moreover, x_1, x_2, and x_3 are coded variables (time, temperature, and polyphenols addition, respectively) for the experimental design in D-F process.

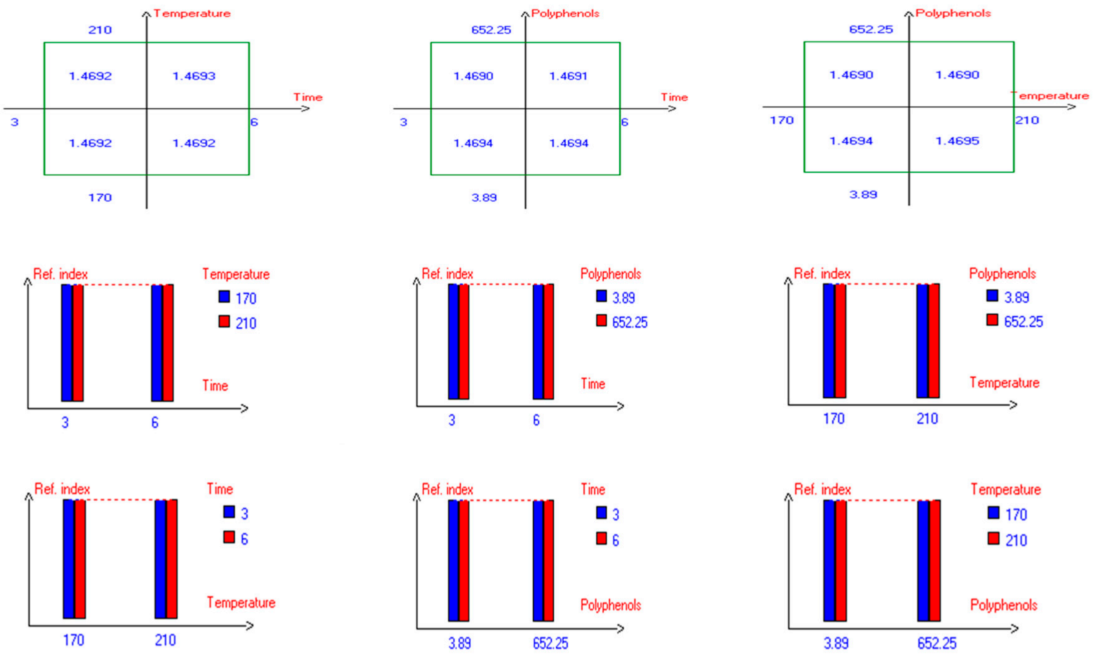

A **B** **C**

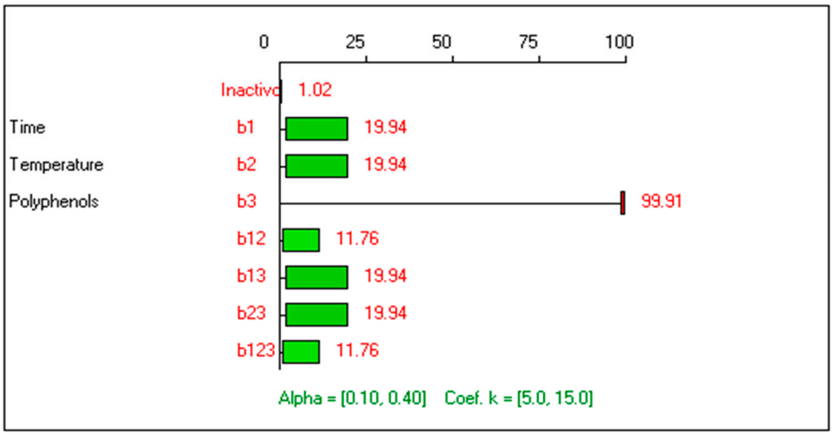

D

Figure S61. Combined interactions between the independent variables on a response variable (**refractive index**) in **Pomace** olive oil under D-F: (**A**) x_1 and x_2, (**B**) x_1 and x_3, (**C**) x_2 and x_3, and (**D**) results of variance analysis of regression equation model and the significance changes in each individual independent variable and interaction between the combined independent variables on refractive index; b represents a significant difference when be $>b_{123}$, while b represents no significant difference when be $\leq b_{123}$; b_1, b_2, b_3 are the main effects of the independent variables, while b_{12}, b_{13}, b_{23}, and b_{123} are the interaction effects of the independent variables. Moreover, x_1, x_2, and x_3 are coded variables (time, temperature, and polyphenols addition, respectively) for the experimental design in D-F process.

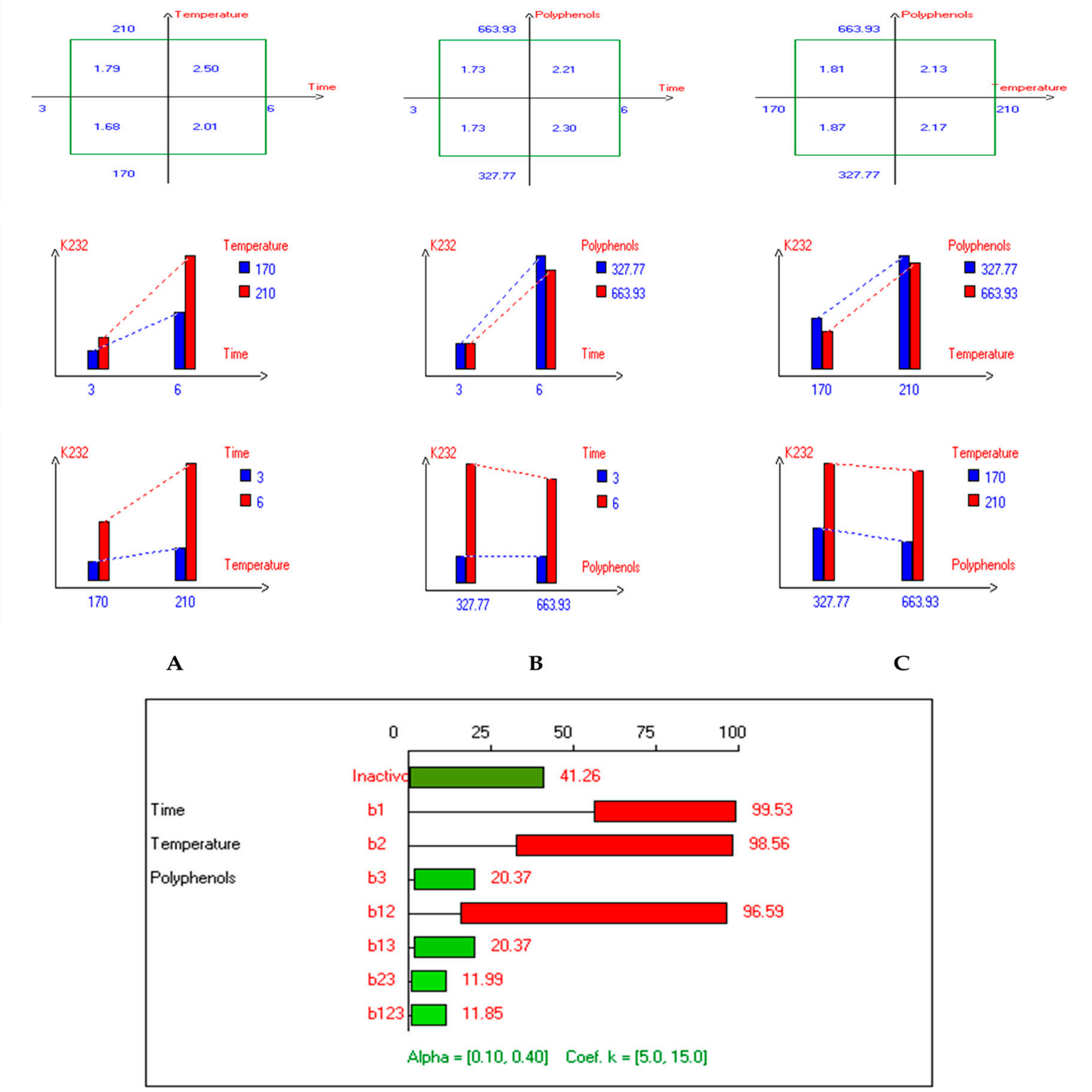

D

Figure S62. Combined interactions between the independent variables on a response variable (**K_{232}**) in EVOO **Koroneiki** under D-F: (**A**) x_1 and x_2, (**B**) x_1 and x_3, (**C**) x_2 and x_3, and (**D**) results of variance analysis of regression equation model and the significance changes in each individual independent variable and interaction between the combined independent variables on K_{232}; b represents a significant difference when be $>b_{123}$, while b represents no significant difference when be $\leq b_{123}$; b_1, b_2, b_3 are the main effects of the independent variables, while b_{12}, b_{13}, b_{23}, and b_{123} are the interaction effects of the independent variables. Moreover, x_1, x_2, and x_3 are coded variables (time, temperature, and polyphenols addition, respectively) for the experimental design in D-F process.

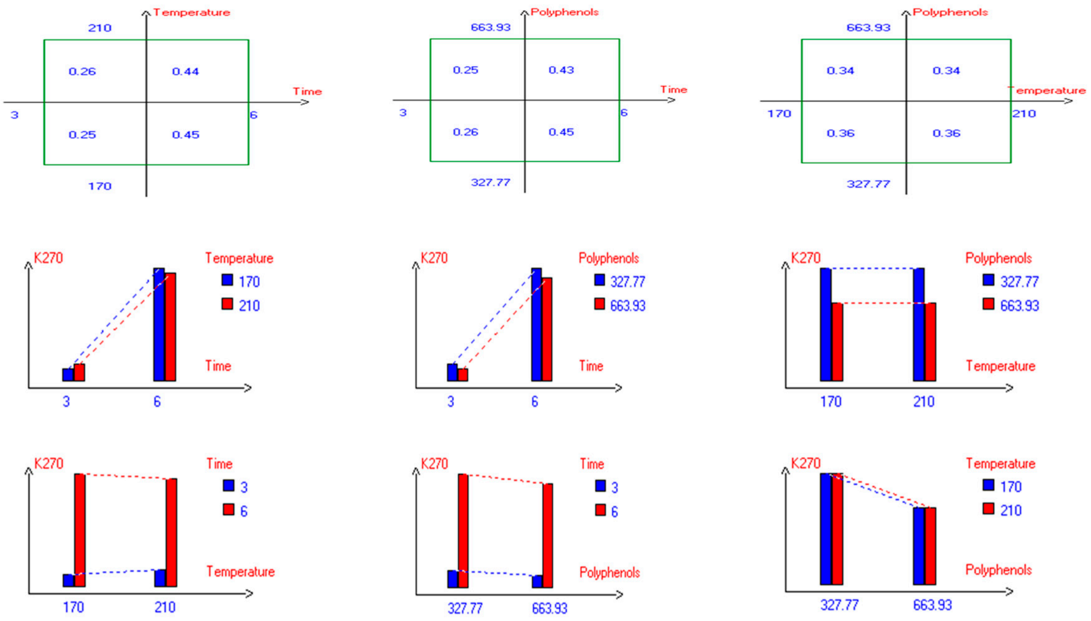

A **B** **C**

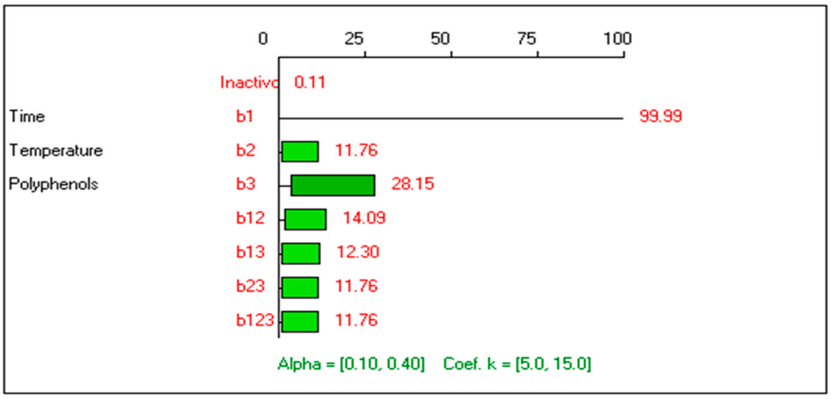

D

Figure S63. Combined interactions between the independent variables on a response variable (**K**$_{270}$) in EVOO **Koroneiki** under D-F: (**A**) x_1 and x_2, (**B**) x_1 and x_3, (**C**) x_2 and x_3, and (**D**) results of variance analysis of regression equation model and the significance changes in each individual independent variable and interaction between the combined independent variables on K$_{270}$; b represents a significant difference when be >b_{123}, while b represents no significant difference when be ≤b_{123}; b_1, b_2, b_3 are the main effects of the independent variables, while b_{12}, b_{13}, b_{23}, and b_{123} are the interaction effects of the independent variables. Moreover, x_I, x_2, and x_3 are coded variables (time, temperature, and polyphenols addition, respectively) for the experimental design in D-F process.

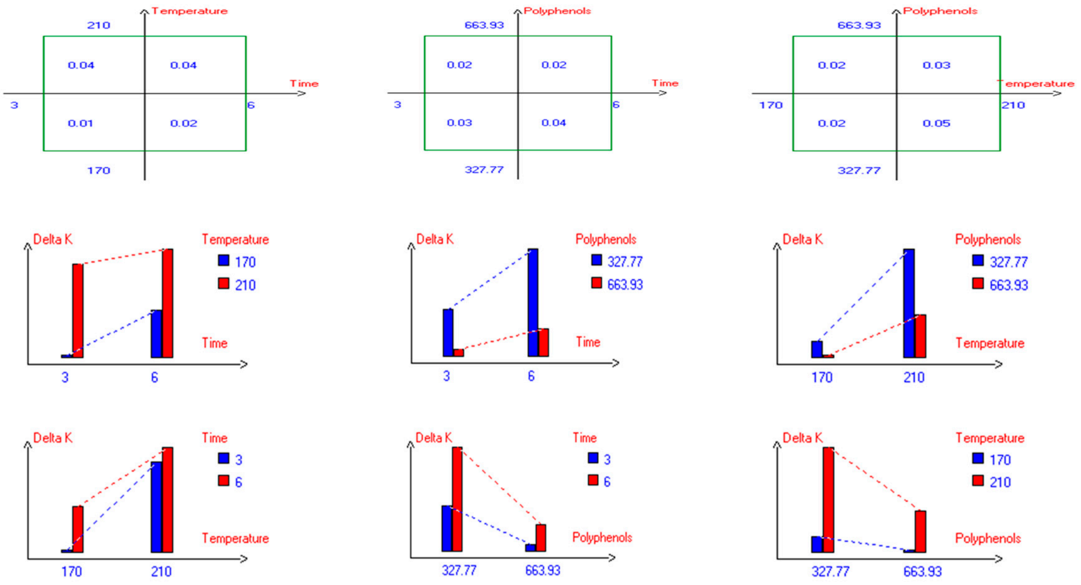

A **B** **C**

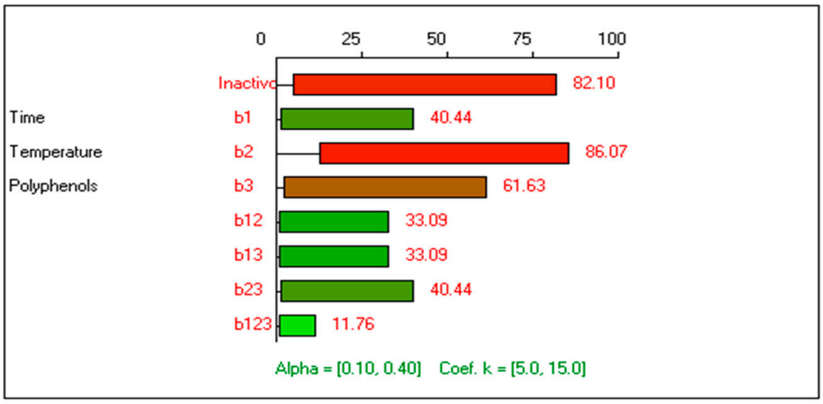

D

Figure S64. Combined interactions between the independent variables on a response variable (ΔK) in EVOO **Koroneiki** under D-F: (**A**) x_1 and x_2, (**B**) x_1 and x_3, (**C**) x_2 and x_3, and (**D**) results of variance analysis of regression equation model and the significance changes in each individual independent variable and interaction between the combined independent variables on ΔK; b represents a significant difference when be $>b_{123}$, while b represents no significant difference when be $\leq b_{123}$; b_1, b_2, b_3 are the main effects of the independent variables, while b_{12}, b_{13}, b_{23}, and b_{123} are the interaction effects of the independent variables. Moreover, x_1, x_2, and x_3 are coded variables (time, temperature, and polyphenols addition, respectively) for the experimental design in D-F process.

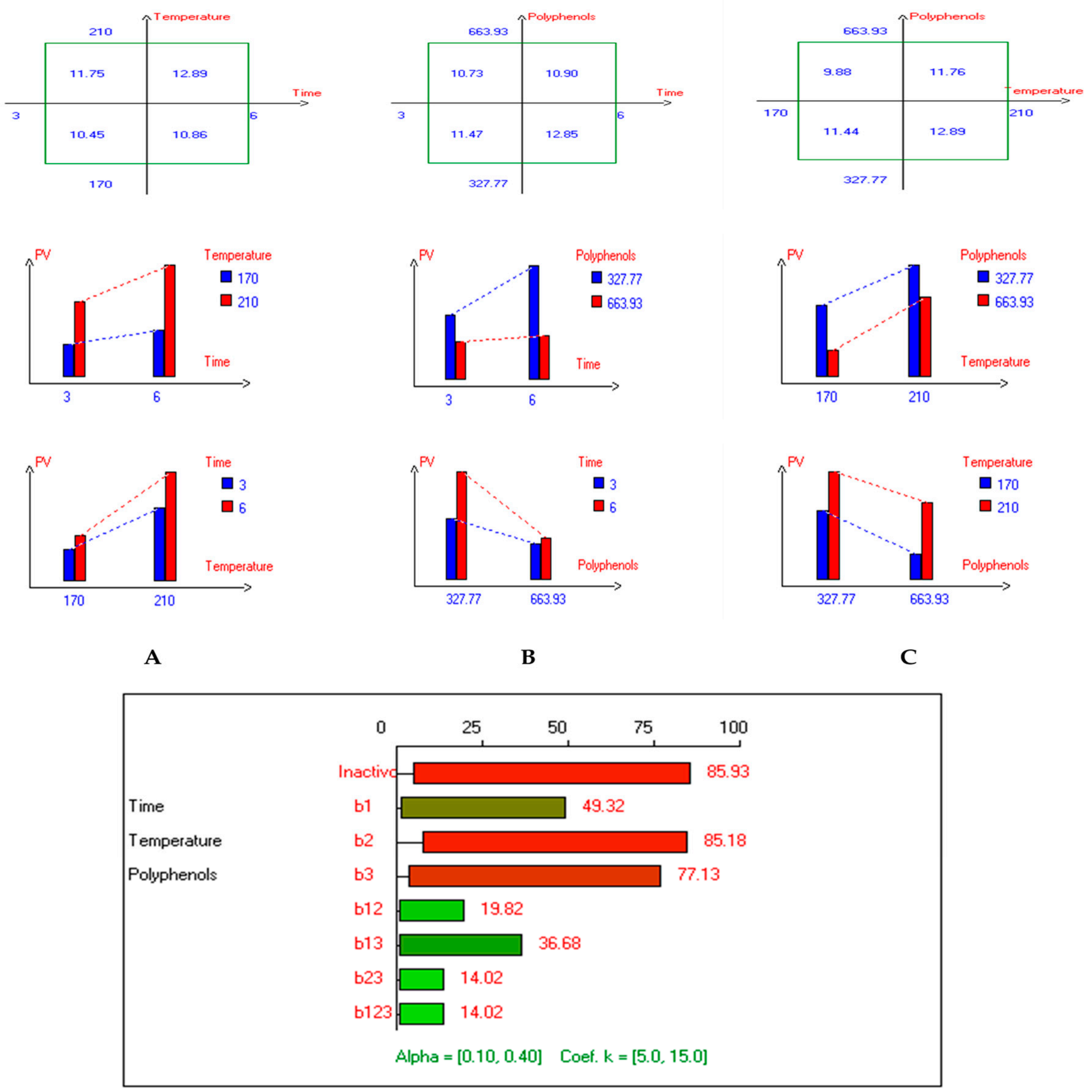

D

Figure S65. Combined interactions between the independent variables on a response variable (**peroxide value** (mEqO_2/kg)) in EVOO **Koroneiki** under D-F: (**A**) x_1 and x_2, (**B**) x_1 and x_3, (**C**) x_2 and x_3, and (**D**) results of variance analysis of regression equation model and the significance changes in each individual independent variable and interaction between the combined independent variables on peroxide value; b represents a significant difference when be >b_{123}, while b represents no significant difference when be ≤b_{123}; b_1, b_2, b_3 are the main effects of the independent variables, while b_{12}, b_{13}, b_{23}, and b_{123} are the interaction effects of the independent variables. Moreover, x_1, x_2, and x_3 are coded variables (time, temperature, and polyphenols addition, respectively) for the experimental design in D-F process.

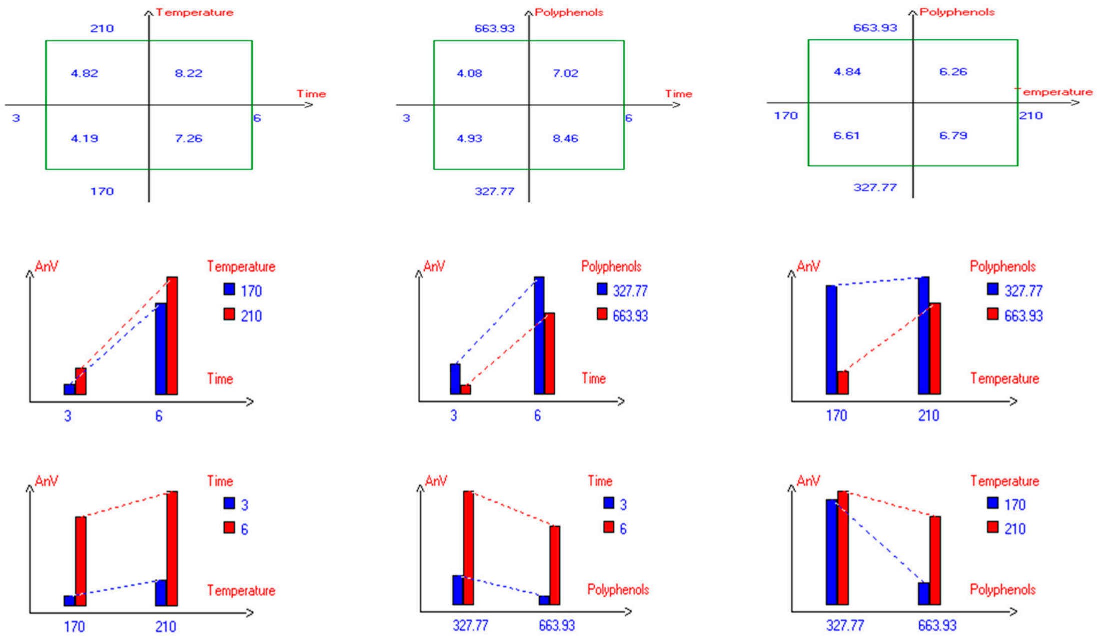

A **B** **C**

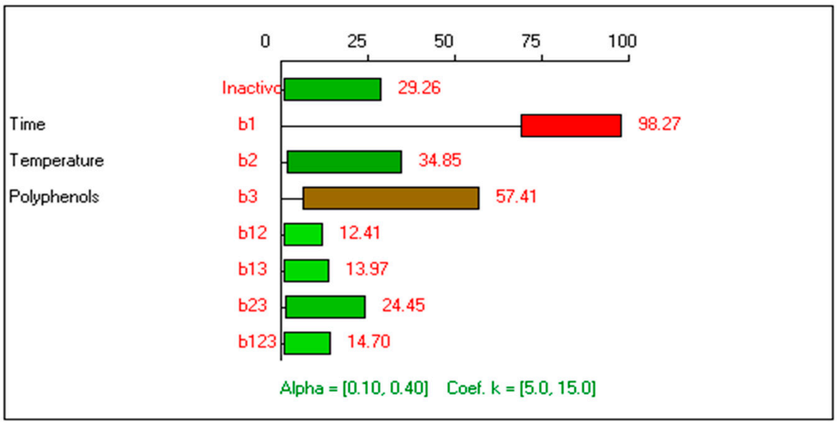

D

Figure S66. Combined interactions between the independent variables on a response variable (**anisidine value** (mg/kg)) in EVOO **Koroneiki** under D-F: (**A**) x_1 and x_2, (**B**) x_1 and x_3, (**C**) x_2 and x_3, and (**D**) results of variance analysis of regression equation model and the significance changes in each individual independent variable and interaction between the combined independent variables on anisidine value; b represents a significant difference when be $>b_{123}$, while b represents no significant difference when be $\leq b_{123}$; b_1, b_2, b_3 are the main effects of the independent variables, while b_{12}, b_{13}, b_{23}, and b_{123} are the interaction effects of the independent variables. Moreover, x_1, x_2, and x_3 are coded variables (time, temperature, and polyphenols addition, respectively) for the experimental design in D-F process.

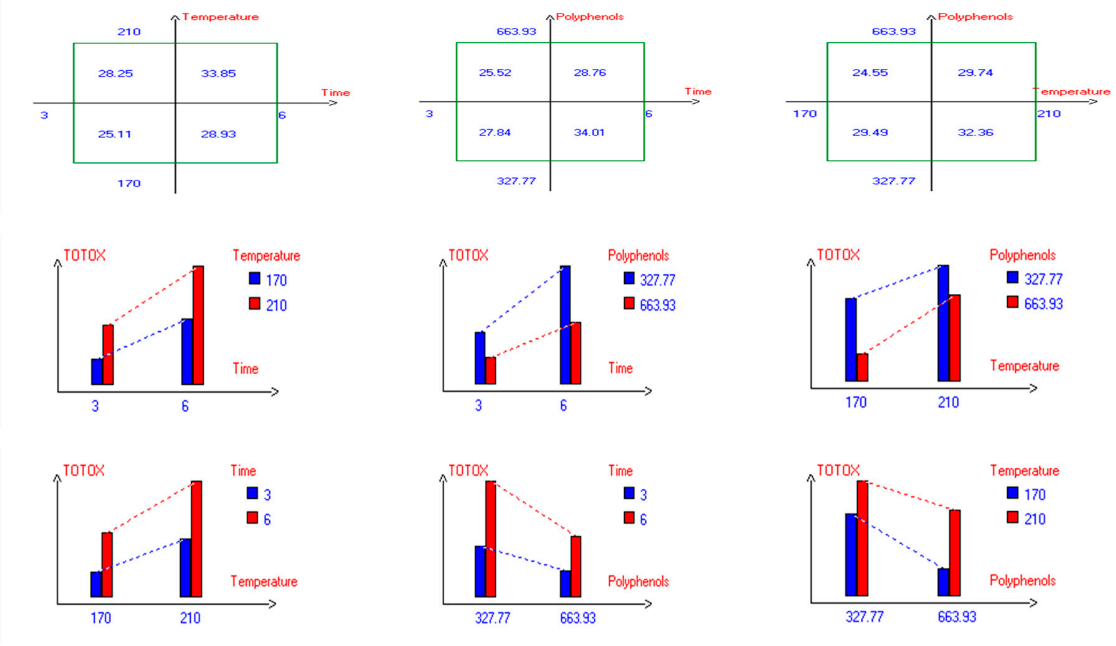

A **B** **C**

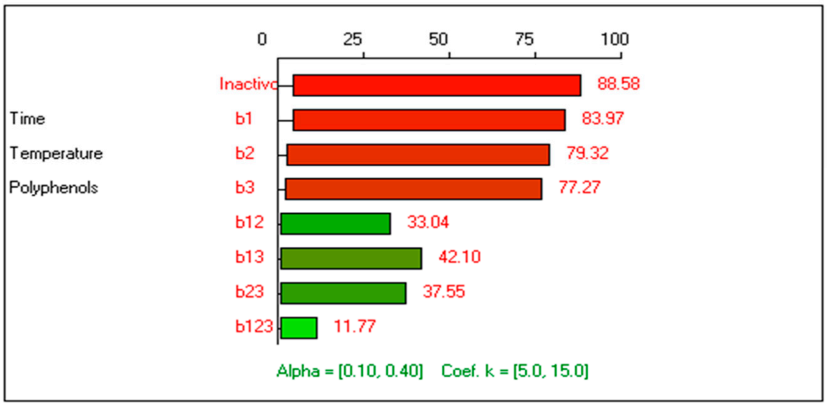

D

Figure S67. Combined interactions between the independent variables on a response variable (**TOTOX**) in EVOO **Koroneiki** under D-F: (**A**) x_1 and x_2, (**B**) x_1 and x_3, (**C**) x_2 and x_3, and (**D**) results of variance analysis of regression equation model and the significance changes in each individual independent variable and interaction between the combined independent variables on TOTOX; b represents a significant difference when be $>b_{123}$, while b represents no significant difference when be $\leq b_{123}$; b_1, b_2, b_3 are the main effects of the independent variables, while b_{12}, b_{13}, b_{23}, and b_{123} are the interaction effects of the independent variables. Moreover, x_1, x_2, and x_3 are coded variables (time, temperature, and polyphenols addition, respectively) for the experimental design in D-F process.

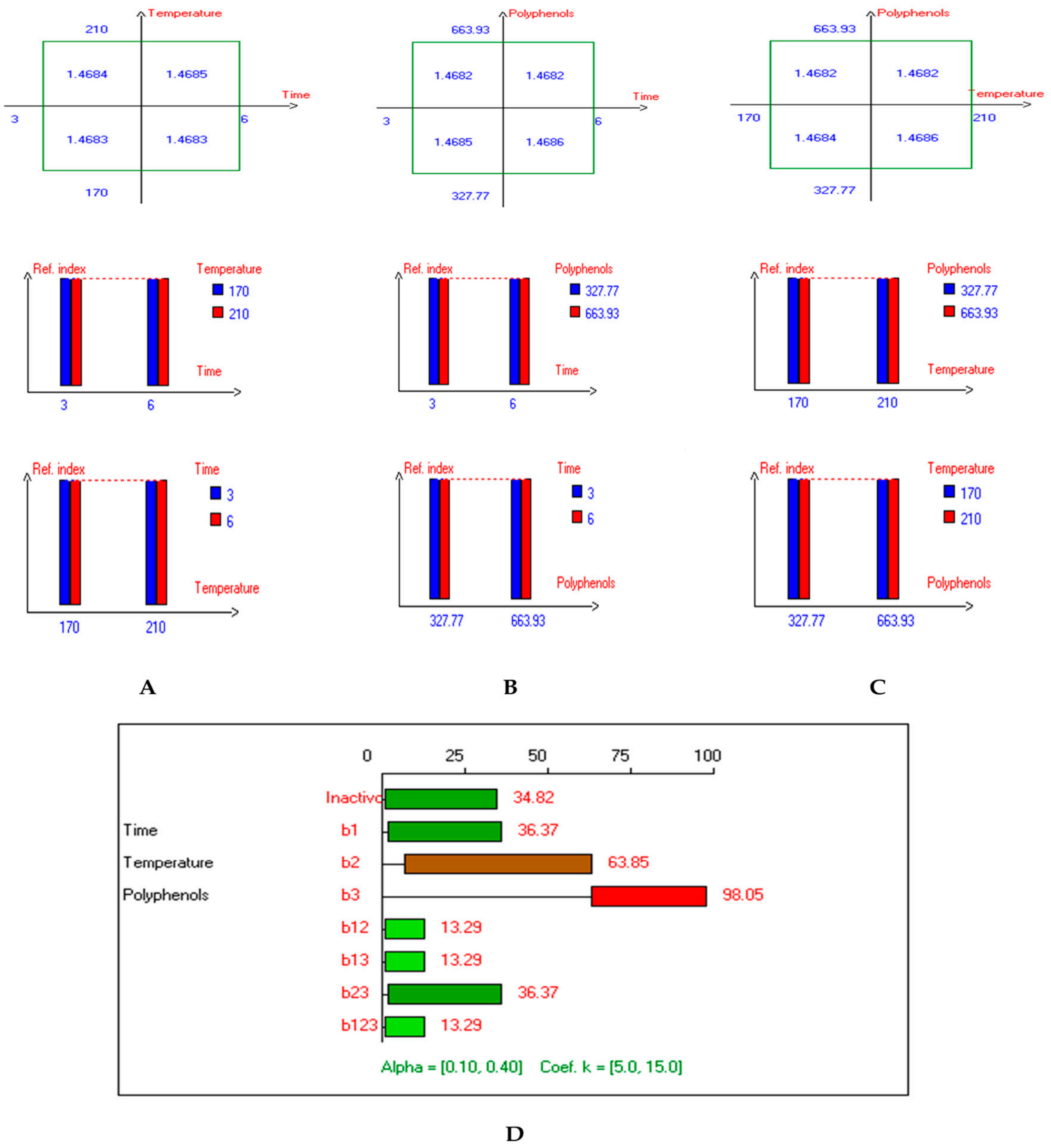

Figure S68. Combined interactions between the independent variables on a response variable (**refractive index**) in EVOO **Koroneiki** under D-F: (**A**) x_1 and x_2, (**B**) x_1 and x_3, (**C**) x_2 and x_3, and (**D**) results of variance analysis of regression equation model and the significance changes in each individual independent variable and interaction between the combined independent variables on refractive index; b represents a significant difference when be >b_{123}, while b represents no significant difference when be ≤b_{123}; b_1, b_2, b_3 are the main effects of the independent variables, while b_{12}, b_{13}, b_{23}, and b_{123} are the interaction effects of the independent variables. Moreover, x_1, x_2, and x_3 are coded variables (time, temperature, and polyphenols addition, respectively) for the experimental design in D-F process.

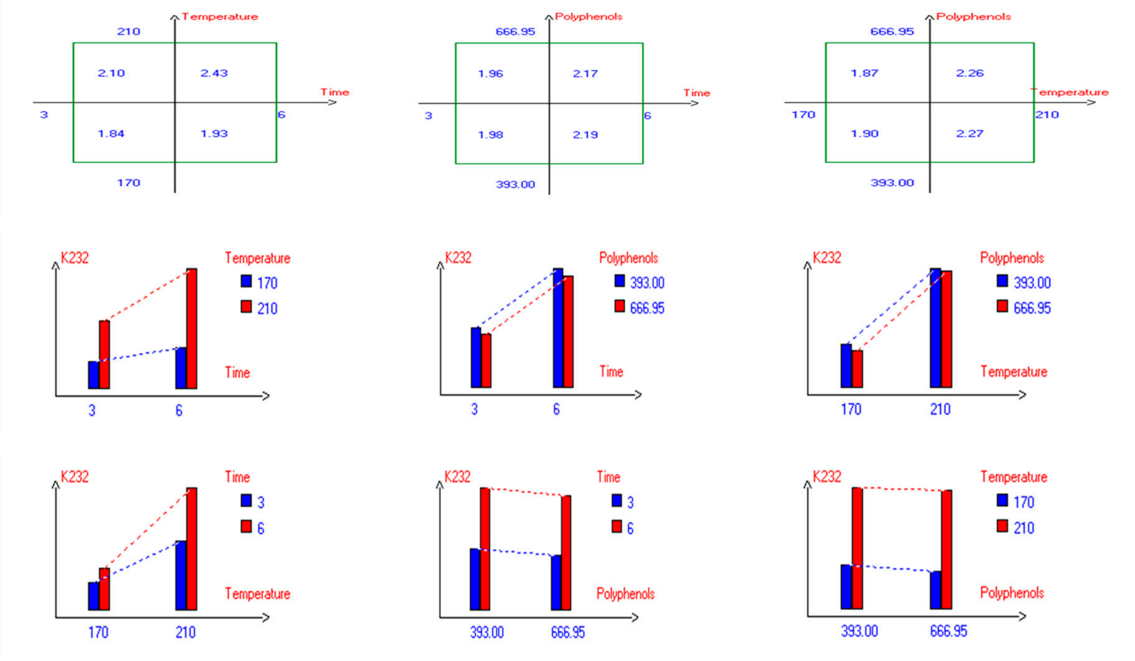

A **B** **C**

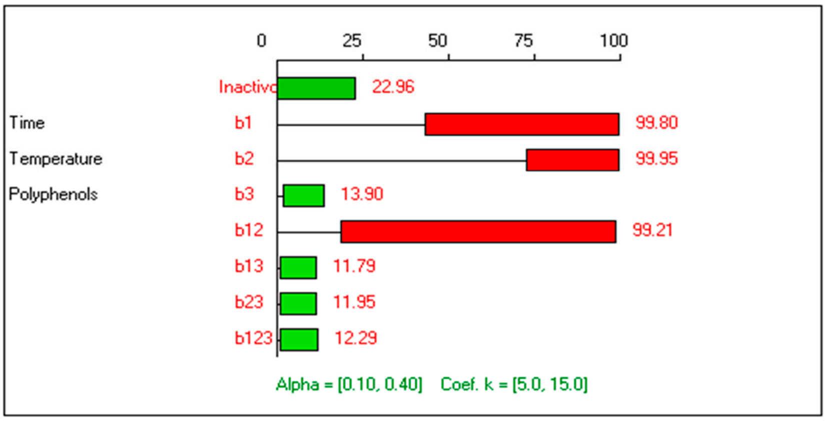

D

Figure S69. Combined interactions between the independent variables on a response variable (**K_{232}**) in EVOO **Arbosana** under D-F: (**A**) x_1 and x_2, (**B**) x_1 and x_3, (**C**) x_2 and x_3, and (**D**) results of variance analysis of regression equation model and the significance changes in each individual independent variable and interaction between the combined independent variables on K_{232}; b represents a significant difference when be $>b_{123}$, while b represents no significant difference when be $\leq b_{123}$; b_1, b_2, b_3 are the main effects of the independent variables, while b_{12}, b_{13}, b_{23}, and b_{123} are the interaction effects of the independent variables. Moreover, x_1, x_2, and x_3 are coded variables (time, temperature, and polyphenols addition, respectively) for the experimental design in D-F process.

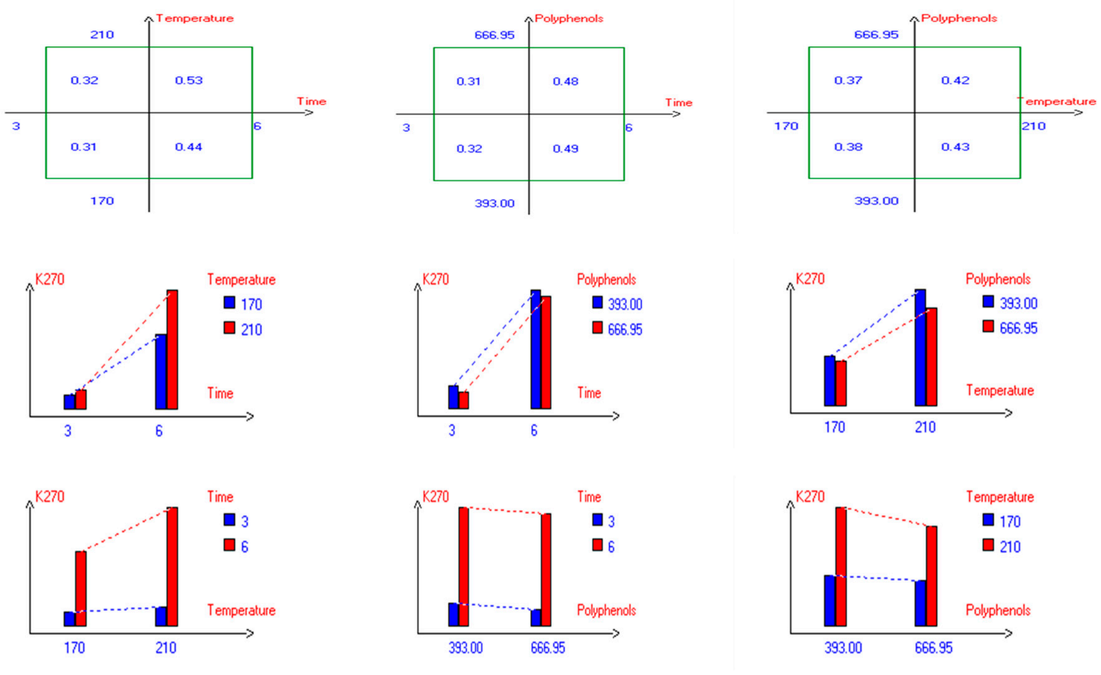

A **B** **C**

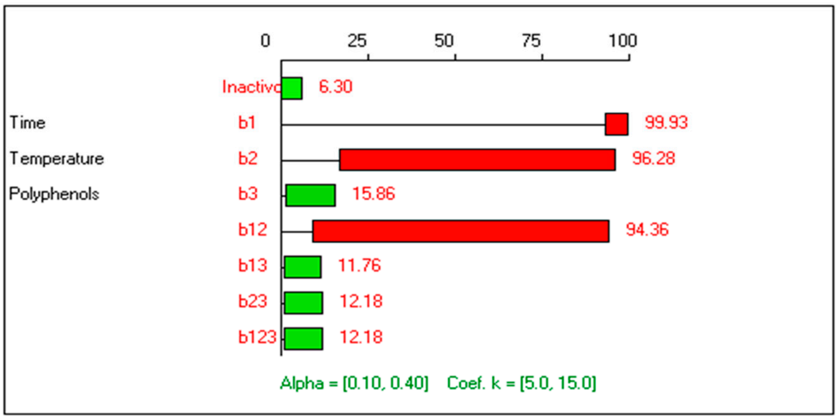

D

Figure S70. Combined interactions between the independent variables on a response variable (**K$_{270}$**) in EVOO **Arbosana** under D-F: (**A**) x_1 and x_2, (**B**) x_1 and x_3, (**C**) x_2 and x_3, and (**D**) results of variance analysis of regression equation model and the significance changes in each individual independent variable and interaction between the combined independent variables on K$_{270}$; b represents a significant difference when be >b_{123}, while b represents no significant difference when be ≤b_{123}; b_1, b_2, b_3 are the main effects of the independent variables, while b_{12}, b_{13}, b_{23}, and b_{123} are the interaction effects of the independent variables. Moreover, x_1, x_2, and x_3 are coded variables (time, temperature, and polyphenols addition, respectively) for the experimental design in D-F process.

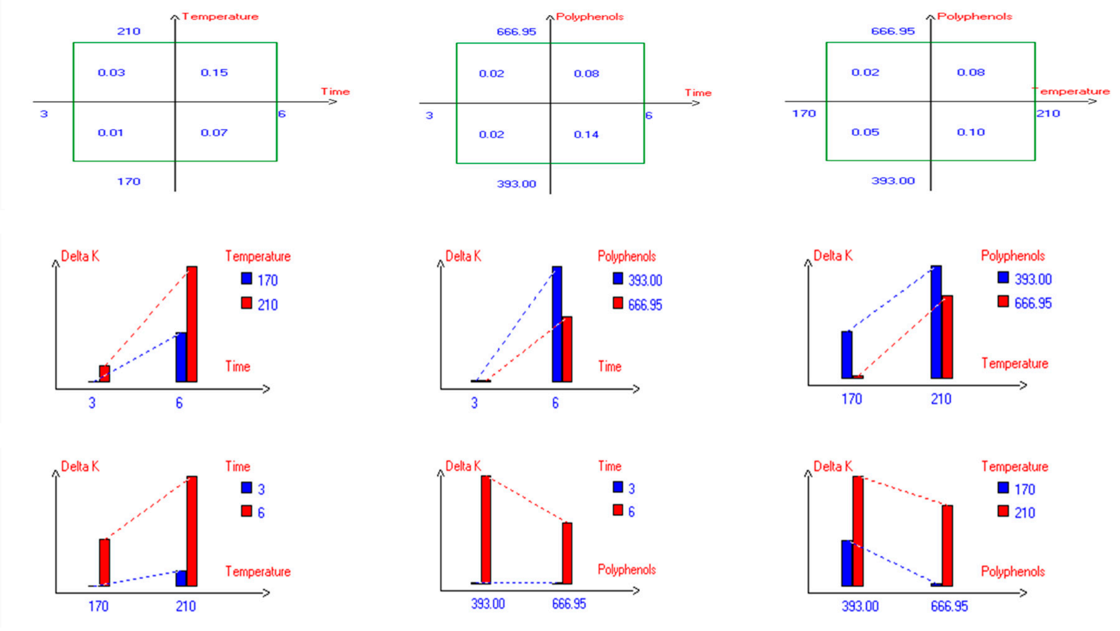

A **B** **C**

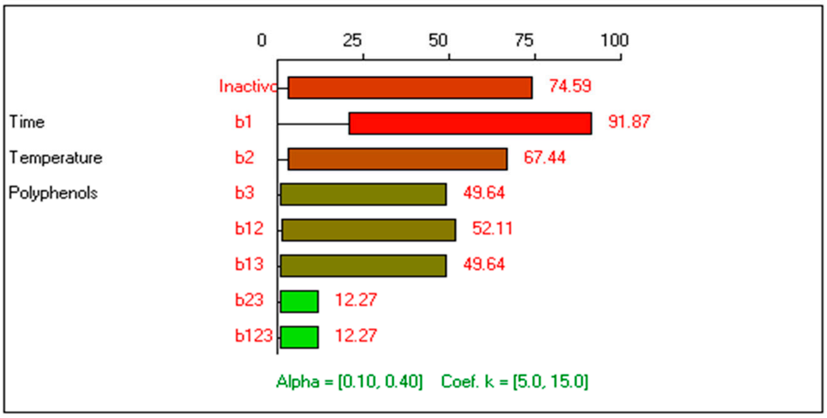

D

Figure S71. Combined interactions between the independent variables on a response variable (ΔK) in EVOO **Arbosana** under D-F: (**A**) x_1 and x_2, (**B**) x_1 and x_3, (**C**) x_2 and x_3, and (**D**) results of variance analysis of regression equation model and the significance changes in each individual independent variable and interaction between the combined independent variables on ΔK; b represents a significant difference when be $>b_{123}$, while b represents no significant difference when be $\leq b_{123}$; b_1, b_2, b_3 are the main effects of the independent variables, while b_{12}, b_{13}, b_{23}, and b_{123} are the interaction effects of the independent variables. Moreover, x_1, x_2, and x_3 are coded variables (time, temperature, and polyphenols addition, respectively) for the experimental design in D-F process.

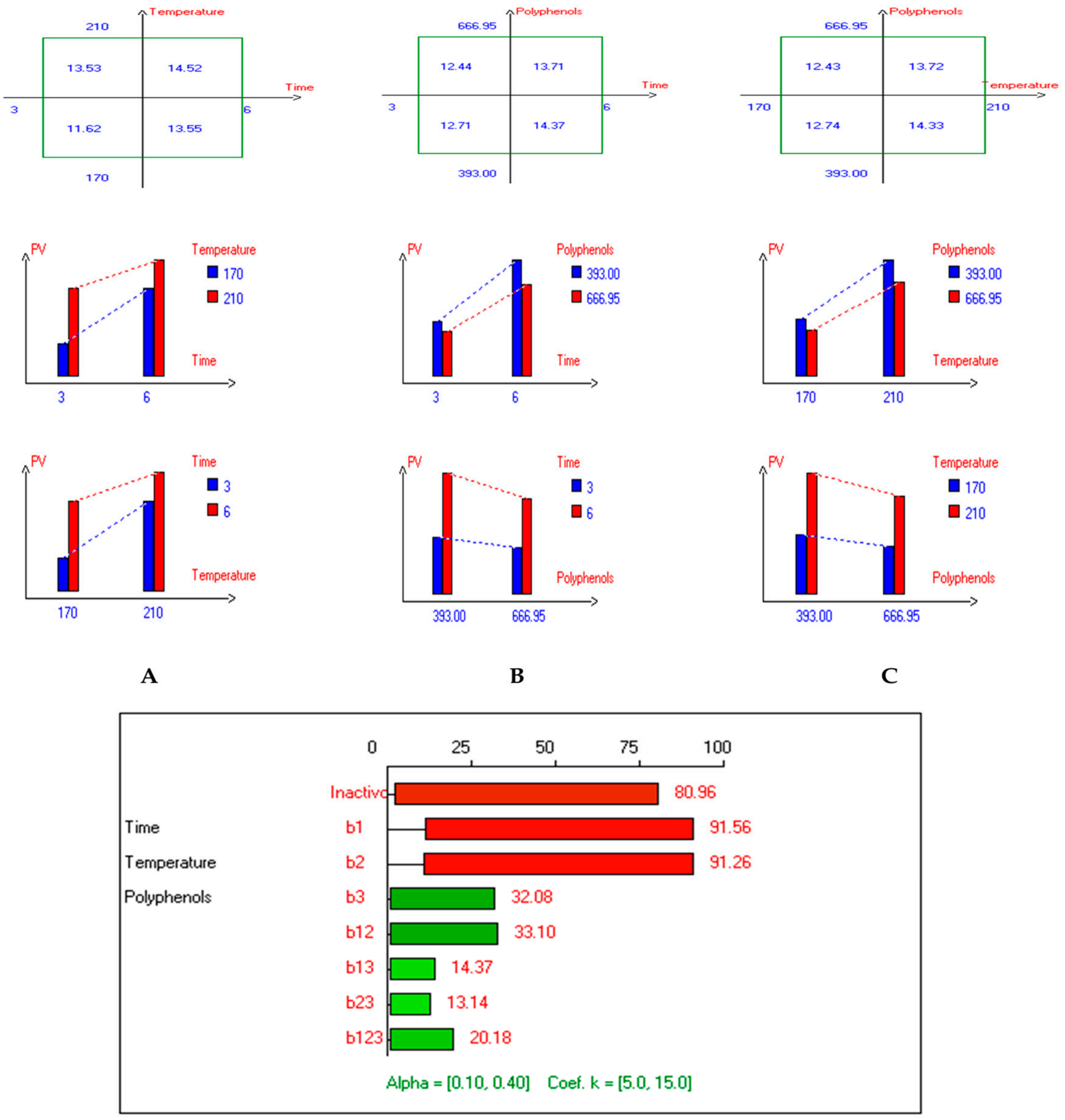

D

Figure S72. Combined interactions between the independent variables on a response variable (**peroxide value** ($mEqO_2$/kg)) in EVOO **Arbosana** under D-F: (**A**) x_1 and x_2, (**B**) x_1 and x_3, (**C**) x_2 and x_3, and (**D**) results of variance analysis of regression equation model and the significance changes in each individual independent variable and interaction between the combined independent variables on peroxide value; b represents a significant difference when be >b_{123}, while b represents no significant difference when be ≤b_{123}; b_1, b_2, b_3 are the main effects of the independent variables, while b_{12}, b_{13}, b_{23}, and b_{123} are the interaction effects of the independent variables. Moreover, x_1, x_2, and x_3 are coded variables (time, temperature, and polyphenols addition, respectively) for the experimental design in D-F process.

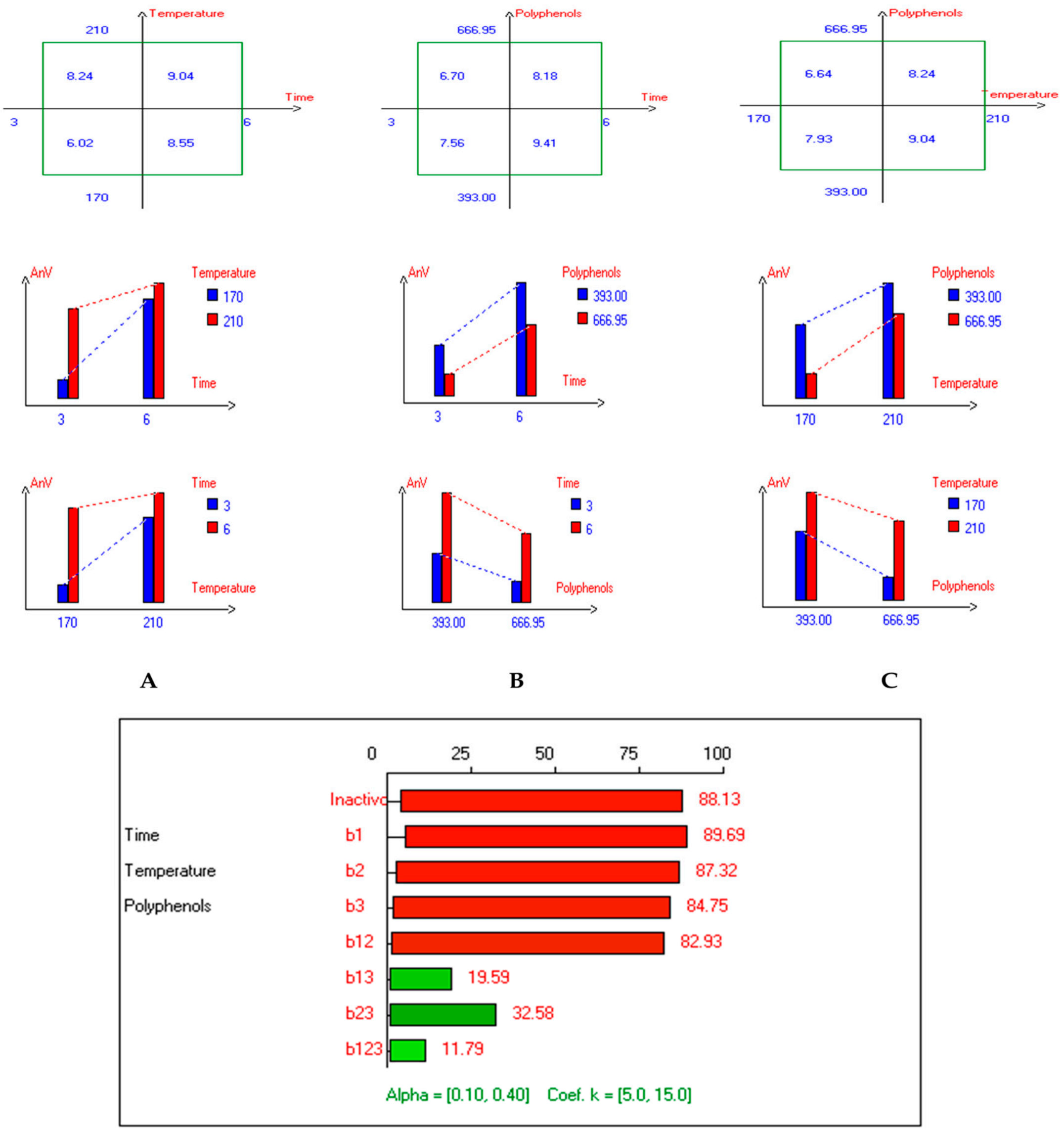

D

Figure S73. Combined interactions between the independent variables on a response variable (**anisidine value** (mg/kg)) in EVOO **Arbosana** under D-F: (**A**) x_1 and x_2, (**B**) x_1 and x_3, (**C**) x_2 and x_3, and (**D**) results of variance analysis of regression equation model and the significance changes in each individual independent variable and interaction between the combined independent variables on anisidine value; b represents a significant difference when be >b_{123}, while b represents no significant difference when be ≤b_{123}; b_1, b_2, b_3 are the main effects of the independent variables, while b_{12}, b_{13}, b_{23}, and b_{123} are the interaction effects of the independent variables. Moreover, x_1, x_2, and x_3 are coded variables (time, temperature, and polyphenols addition, respectively) for the experimental design in D-F process.

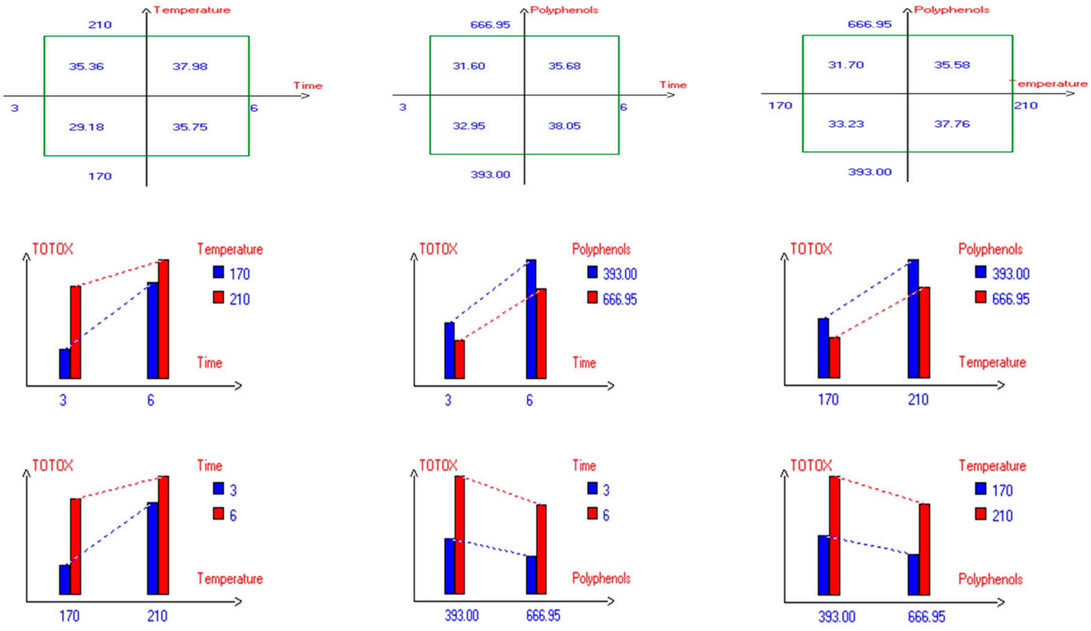

A **B** **C**

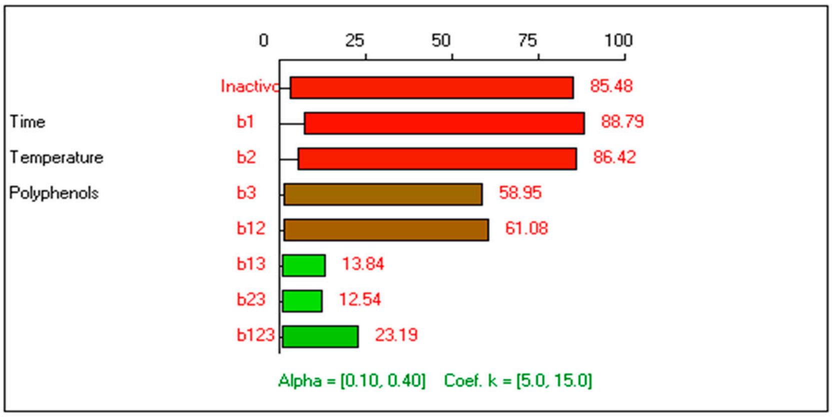

D

Figure S74. Combined interactions between the independent variables on a response variable (**TOTOX**) in EVOO **Arbosana** under D-F: (**A**) x_1 and x_2, (**B**) x_1 and x_3, (**C**) x_2 and x_3, and (**D**) results of variance analysis of regression equation model and the significance changes in each individual independent variable and interaction between the combined independent variables on TOTOX; b represents a significant difference when be $>b_{123}$, while b represents no significant difference when be $\leq b_{123}$; b_1, b_2, b_3 are the main effects of the independent variables, while b_{12}, b_{13}, b_{23}, and b_{123} are the interaction effects of the independent variables. Moreover, x_1, x_2, and x_3 are coded variables (time, temperature, and polyphenols addition, respectively) for the experimental design in D-F process.

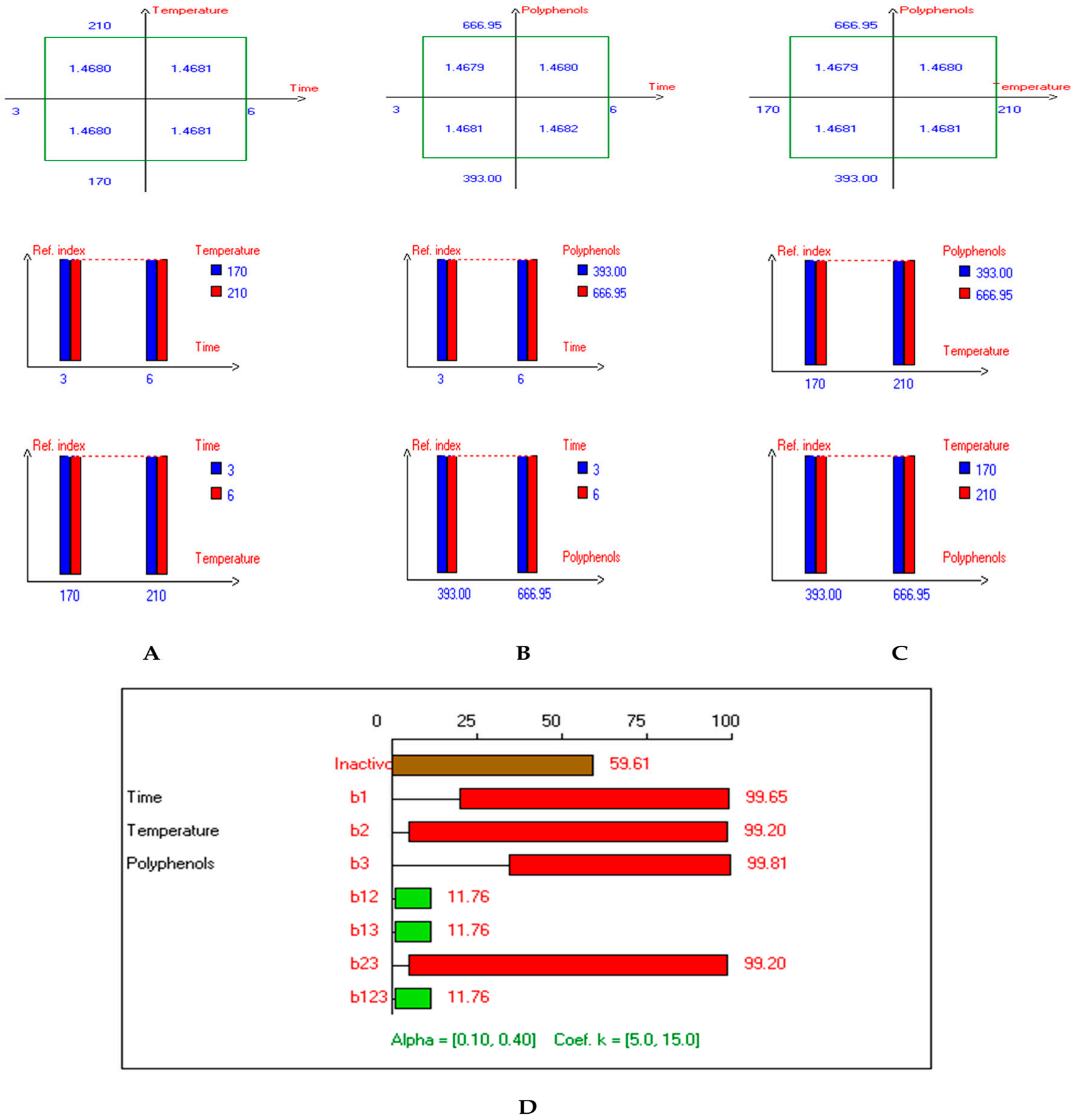

Figure S75. Combined interactions between the independent variables on a response variable (**refractive index**) in EVOO **Arbosana** under D-F: (**A**) x_1 and x_2, (**B**) x_1 and x_3, (**C**) x_2 and x_3, and (**D**) results of variance analysis of regression equation model and the significance changes in each individual independent variable and interaction between the combined independent variables on refractive index; b represents a significant difference when be >b_{123}, while b represents no significant difference when be ≤b_{123}; b_1, b_2, b_3 are the main effects of the independent variables, while b_{12}, b_{13}, b_{23}, and b_{123} are the interaction effects of the independent variables. Moreover, x_1, x_2, and x_3 are coded variables (time, temperature, and polyphenols addition, respectively) for the experimental design in D-F process.

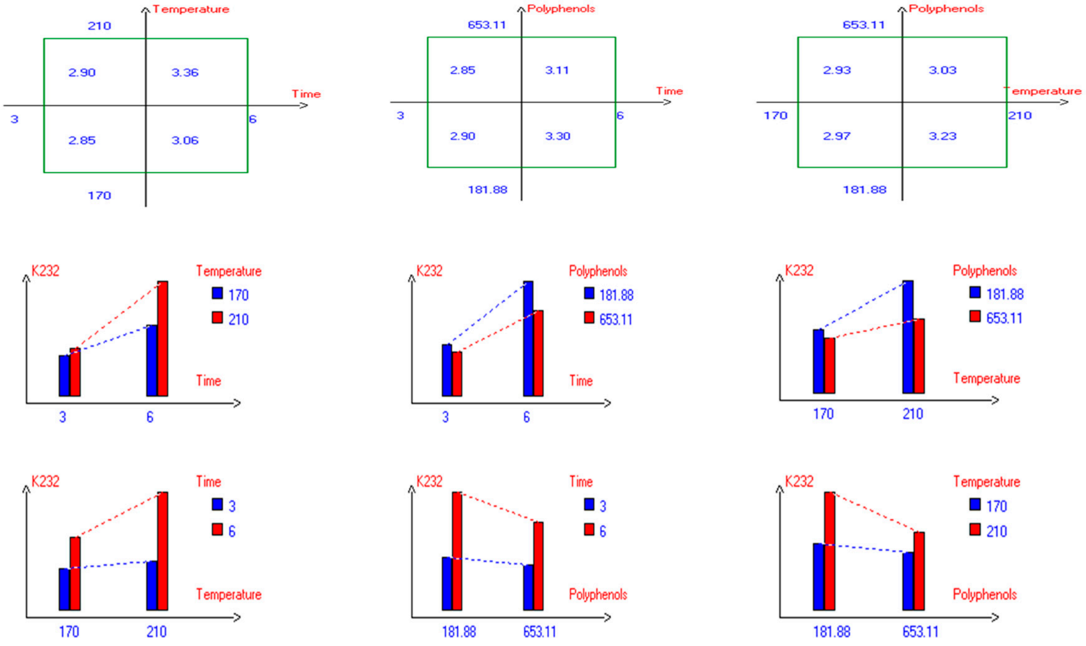

A **B** **C**

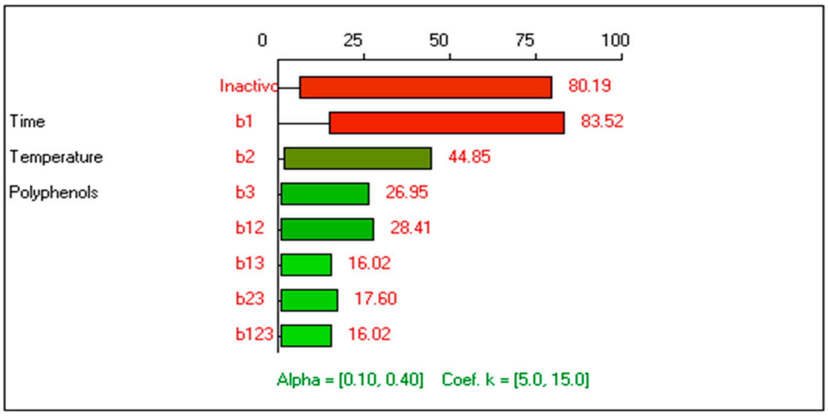

D

Figure S76. Combined interactions between the independent variables on a response variable (**K_{232}**) in **Olive oil 1°** under D-F: (**A**) x_1 and x_2, (**B**) x_1 and x_3, (**C**) x_2 and x_3, and (**D**) results of variance analysis of regression equation model and the significance changes in each individual independent variable and interaction between the combined independent variables on K_{232}; b represents a significant difference when be $>b_{123}$, while b represents no significant difference when be $\leq b_{123}$; b_1, b_2, b_3 are the main effects of the independent variables, while b_{12}, b_{13}, b_{23}, and b_{123} are the interaction effects of the independent variables. Moreover, x_1, x_2, and x_3 are coded variables (time, temperature, and polyphenols addition, respectively) for the experimental design in D-F process.

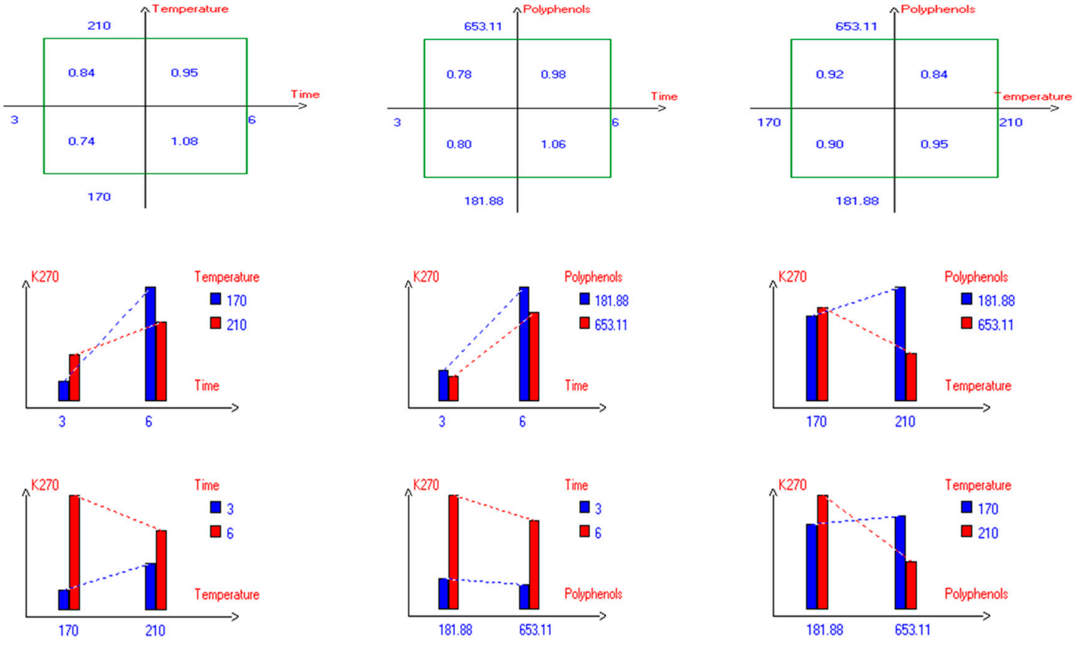

A **B** **C**

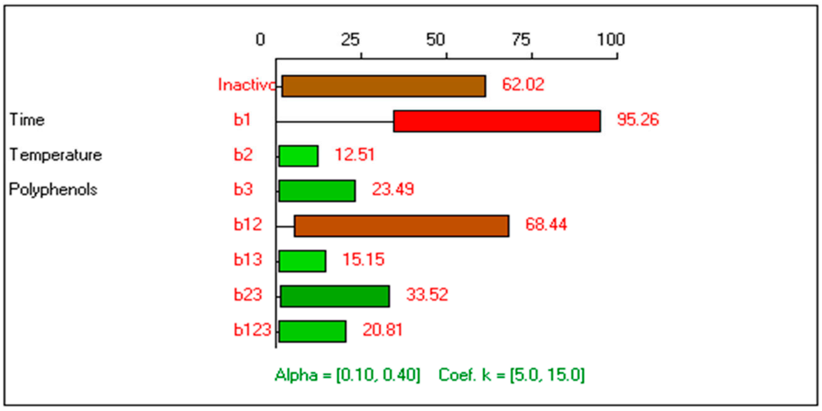

D

Figure S77. Combined interactions between the independent variables on a response variable (**K_{270}**) in **Olive oil 1°** under D-F: (**A**) x_1 and x_2, (**B**) x_1 and x_3, (**C**) x_2 and x_3, and (**D**) results of variance analysis of regression equation model and the significance changes in each individual independent variable and interaction between the combined independent variables on K_{270}; b represents a significant difference when be >b_{123}, while b represents no significant difference when be ≤b_{123}; b_1, b_2, b_3 are the main effects of the independent variables, while b_{12}, b_{13}, b_{23}, and b_{123} are the interaction effects of the independent variables. Moreover, x_I, x_2, and x_3 are coded variables (time, temperature, and polyphenols addition, respectively) for the experimental design in D-F process.

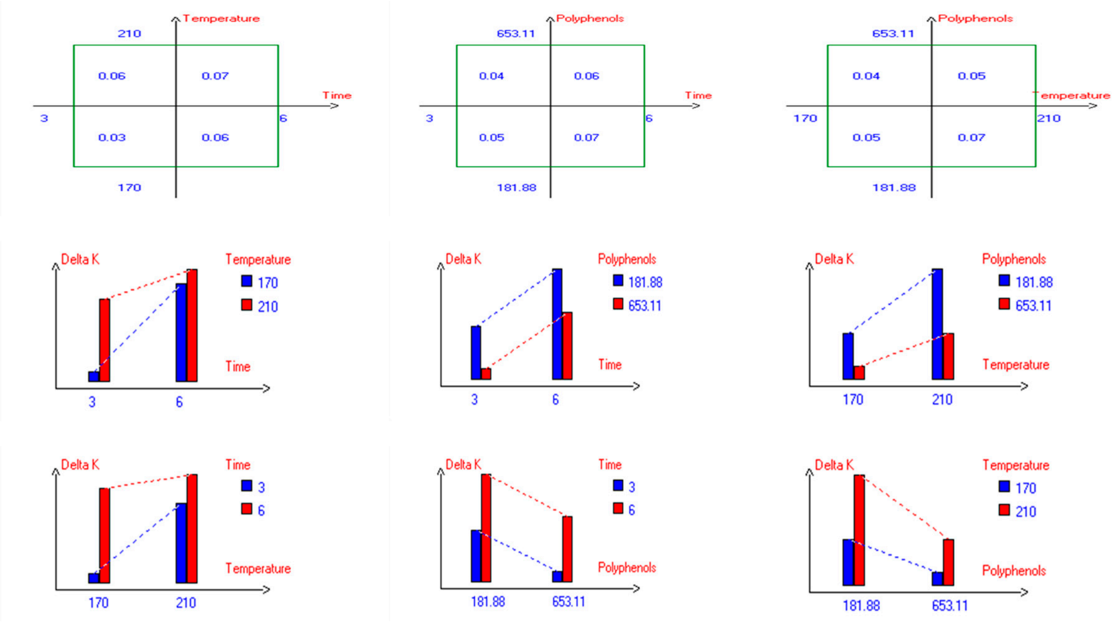

A **B** **C**

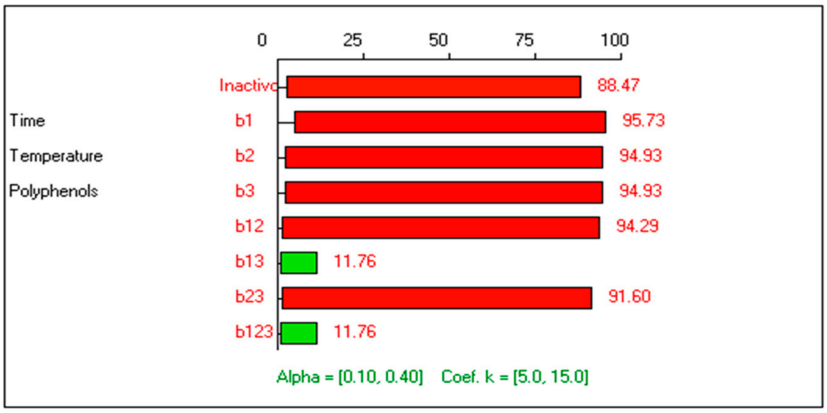

D

Figure S78. Combined interactions between the independent variables on a response variable (ΔK) in **Olive oil 1°** under D-F: (**A**) x_1 and x_2, (**B**) x_1 and x_3, (**C**) x_2 and x_3, and (**D**) results of variance analysis of regression equation model and the significance changes in each individual independent variable and interaction between the combined independent variables on ΔK; b represents a significant difference when be >b_{123}, while b represents no significant difference when be ≤b_{123}; b_1, b_2, b_3 are the main effects of the independent variables, while b_{12}, b_{13}, b_{23}, and b_{123} are the interaction effects of the independent variables. Moreover, x_1, x_2, and x_3 are coded variables (time, temperature, and polyphenols addition, respectively) for the experimental design in D-F process.

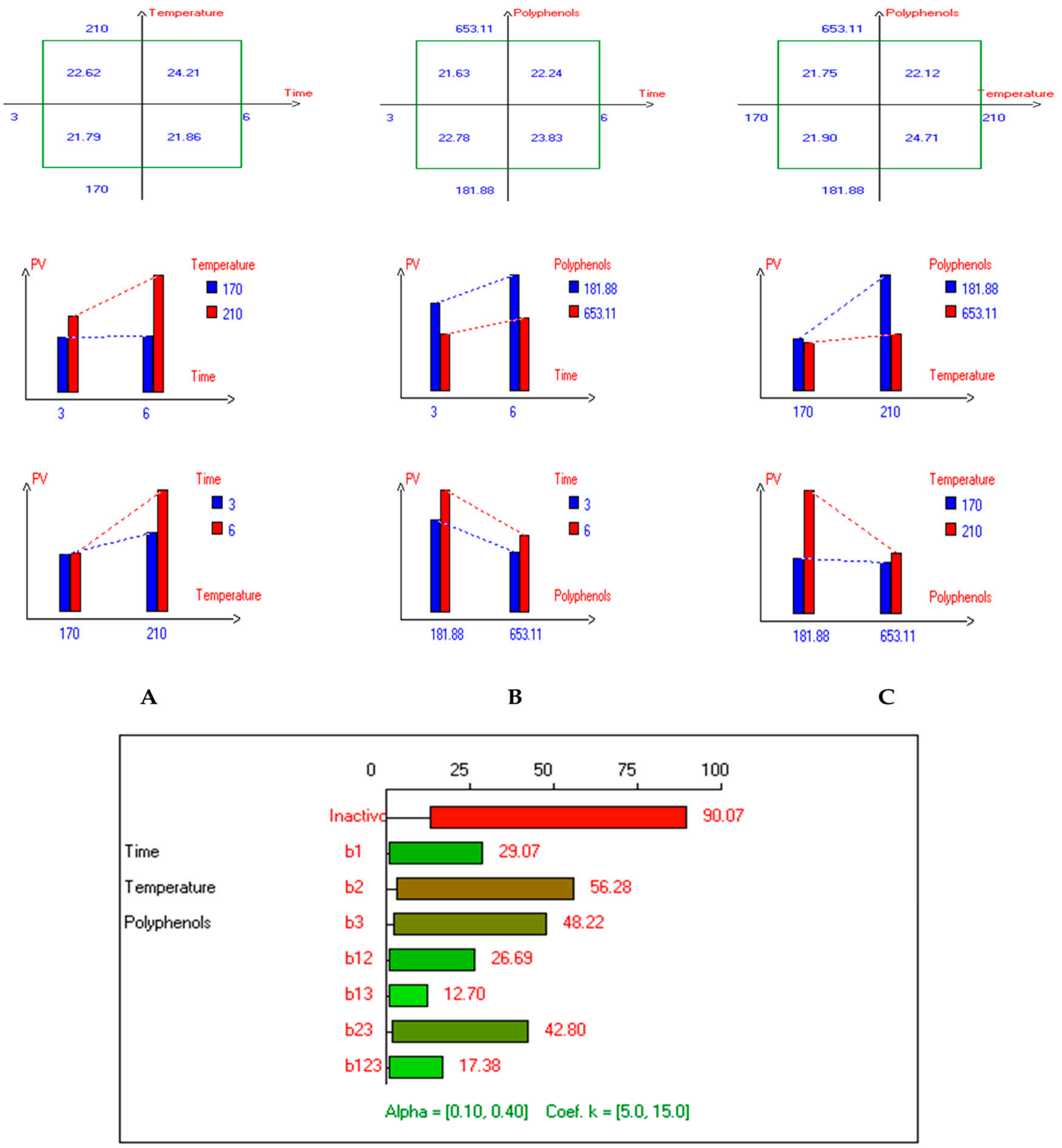

D

Figure S79. Combined interactions between the independent variables on a response variable (**peroxide value** (mEqO_2/kg)) in **Olive oil 1°** under D-F: (**A**) x_1 and x_2, (**B**) x_1 and x_3, (**C**) x_2 and x_3, and (**D**) results of variance analysis of regression equation model and the significance changes in each individual independent variable and interaction between the combined independent variables on peroxide value; b represents a significant difference when be >b_{123}, while b represents no significant difference when be ≤b_{123}; b_1, b_2, b_3 are the main effects of the independent variables, while b_{12}, b_{13}, b_{23}, and b_{123} are the interaction effects of the independent variables. Moreover, x_1, x_2, and x_3 are coded variables (time, temperature, and polyphenols addition, respectively) for the experimental design in D-F process.

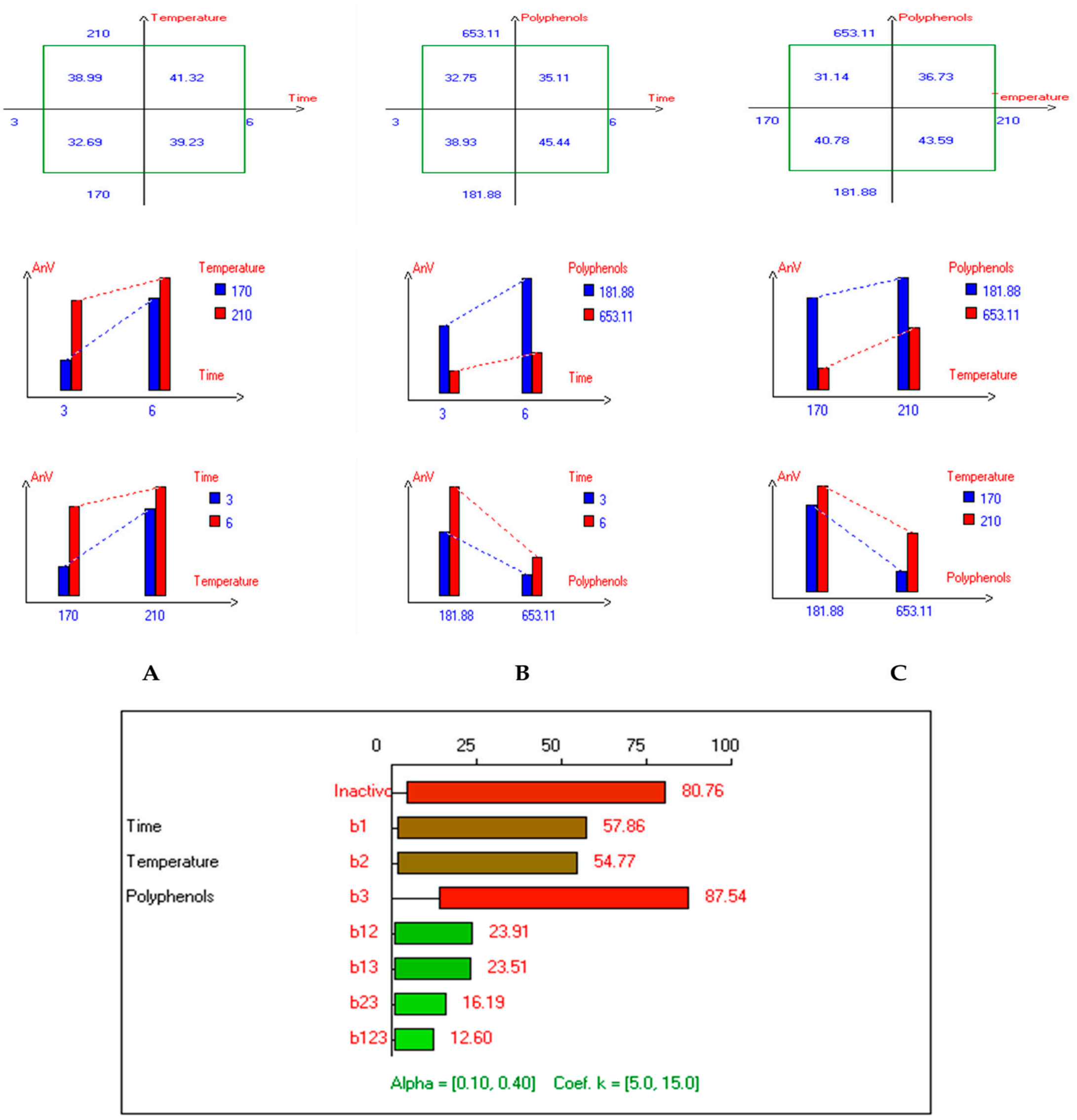

D

Figure S80. Combined interactions between the independent variables on a response variable (**anisidine value** (mg/kg)) in **Olive oil 1°** under D-F: (**A**) x_1 and x_2, (**B**) x_1 and x_3, (**C**) x_2 and x_3, and (**D**) results of variance analysis of regression equation model and the significance changes in each individual independent variable and interaction between the combined independent variables on anisidine value; b represents a significant difference when be $>b_{123}$, while b represents no significant difference when be $\leq b_{123}$; b_1, b_2, b_3 are the main effects of the independent variables, while b_{12}, b_{13}, b_{23}, and b_{123} are the interaction effects of the independent variables. Moreover, x_1, x_2, and x_3 are coded variables (time, temperature, and polyphenols addition, respectively) for the experimental design in D-F process.

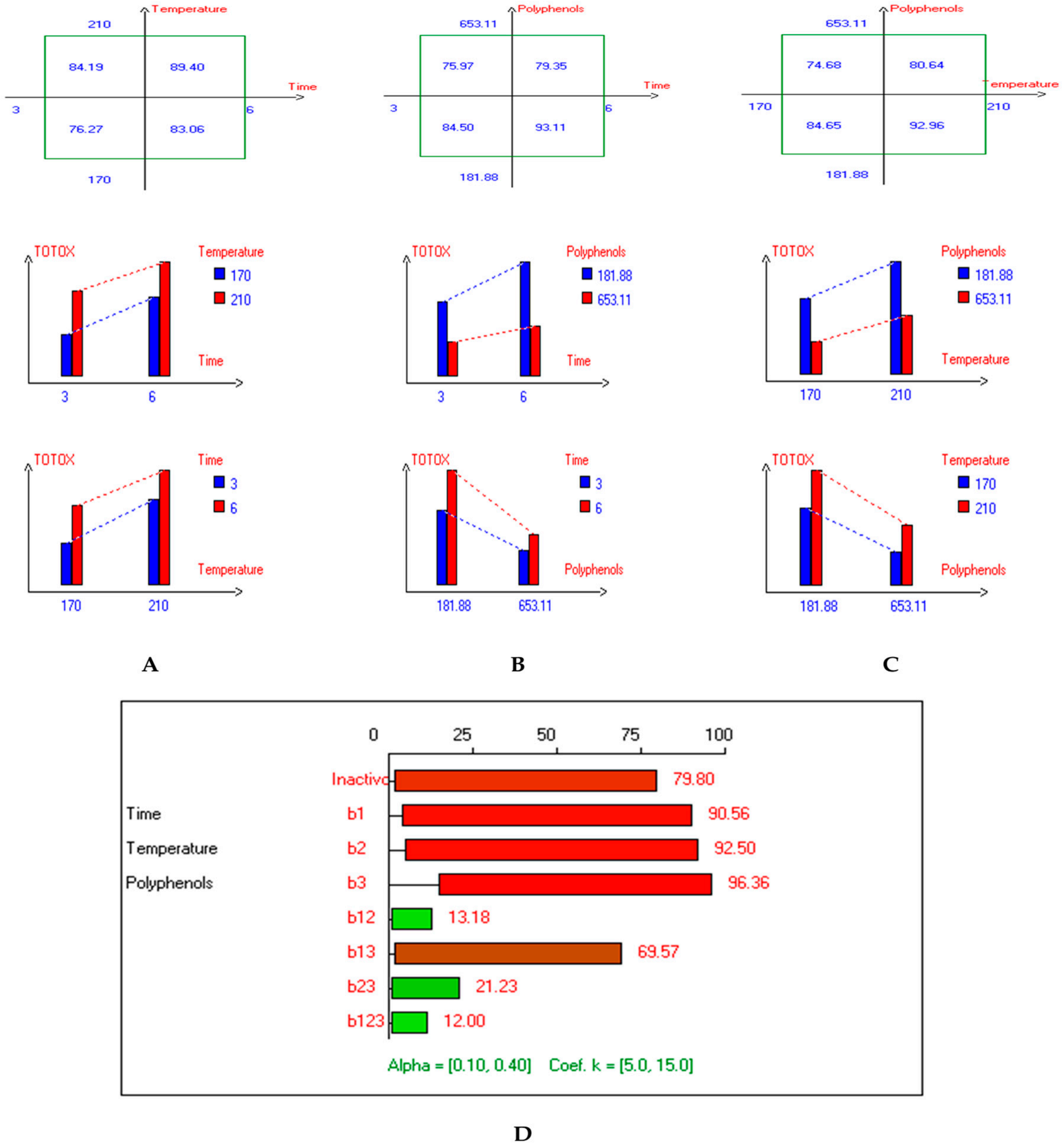

Figure S81. Combined interactions between the independent variables on a response variable (**TOTOX**) in **Olive oil 1°** under D-F: (**A**) x_1 and x_2, (**B**) x_1 and x_3, (**C**) x_2 and x_3, and (**D**) results of variance analysis of regression equation model and the significance changes in each individual independent variable and interaction between the combined independent variables on TOTOX; b represents a significant difference when be >b_{123}, while b represents no significant difference when be ≤b_{123}; b_1, b_2, b_3 are the main effects of the independent variables, while b_{12}, b_{13}, b_{23}, and b_{123} are the interaction effects of the independent variables. Moreover, x_1, x_2, and x_3 are coded variables (time, temperature, and polyphenols addition, respectively) for the experimental design in D-F process.

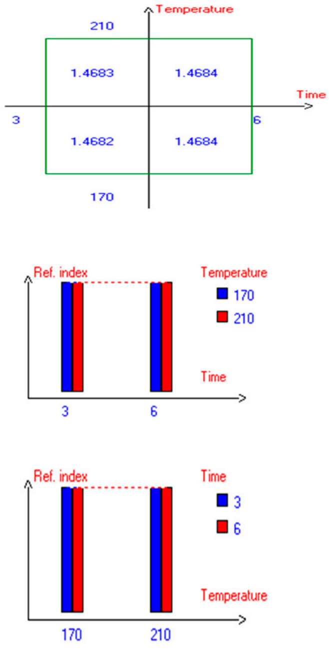

A

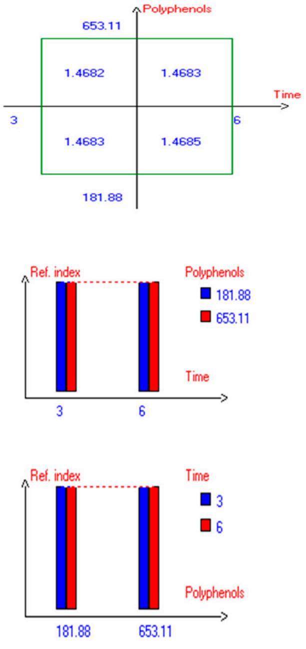

B

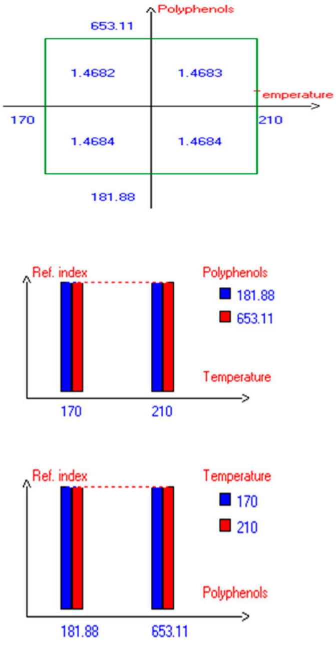

C

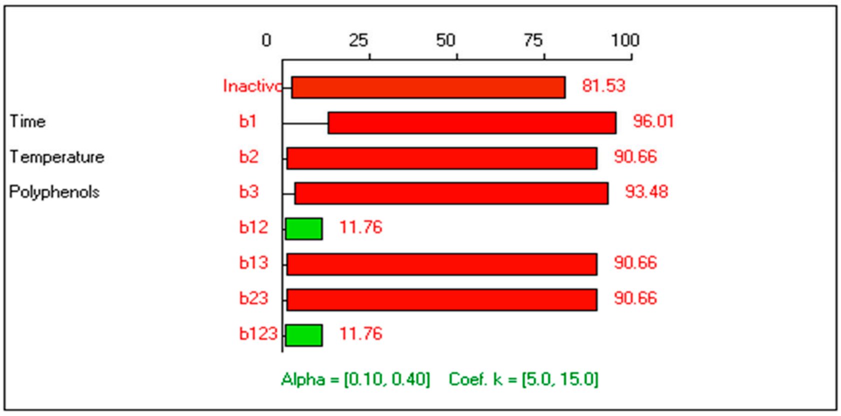

D

Figure S82. Combined interactions between the independent variables on a response variable (**refractive index**) in **Olive oil 1°** under D-F: (**A**) x_1 and x_2, (**B**) x_1 and x_3, (**C**) x_2 and x_3, and (**D**) results of variance analysis of regression equation model and the significance changes in each individual independent variable and interaction between the combined independent variables on refractive index; b represents a significant difference when be $>b_{123}$, while b represents no significant difference when be $\leq b_{123}$; b_1, b_2, b_3 are the main effects of the independent variables, while b_{12}, b_{13}, b_{23}, and b_{123} are the interaction effects of the independent variables. Moreover, x_1, x_2, and x_3 are coded variables (time, temperature, and polyphenols addition, respectively) for the experimental design in D-F process.

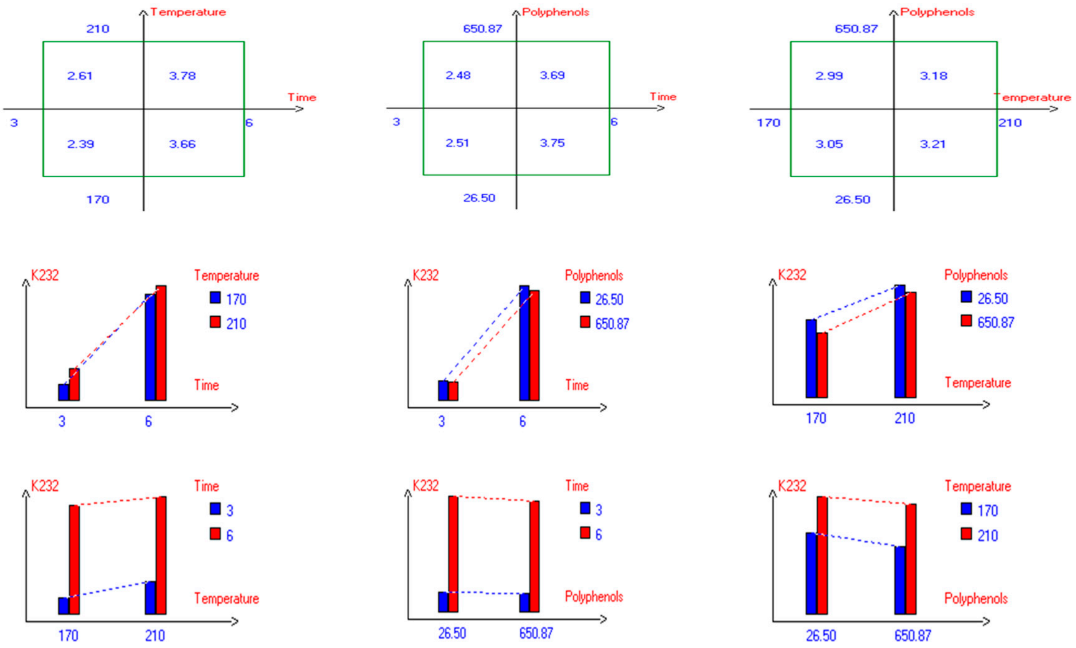

A **B** **C**

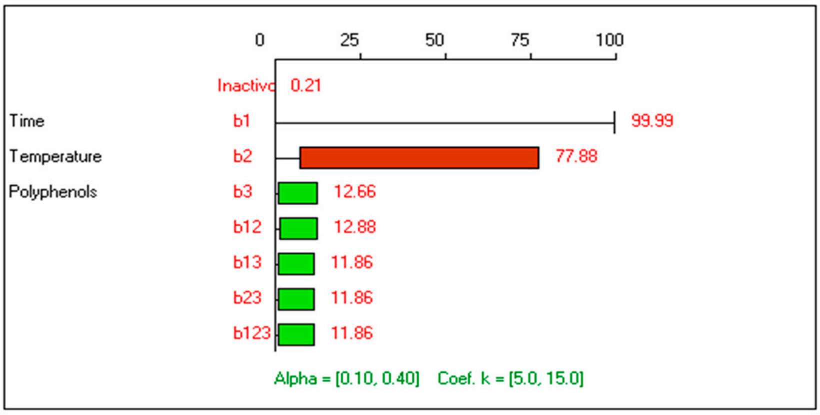

D

Figure S83. Combined interactions between the independent variables on a response variable (**K**$_{232}$) in **Olive oil 0.4°** under D-F: (**A**) x_1 and x_2, (**B**) x_1 and x_3, (**C**) x_2 and x_3, and (**D**) results of variance analysis of regression equation model and the significance changes in each individual independent variable and interaction between the combined independent variables on K$_{232}$; b represents a significant difference when be >b_{123}, while b represents no significant difference when be ≤b_{123}; b_1, b_2, b_3 are the main effects of the independent variables, while b_{12}, b_{13}, b_{23}, and b_{123} are the interaction effects of the independent variables. Moreover, x_1, x_2, and x_3 are coded variables (time, temperature, and polyphenols addition, respectively) for the experimental design in D-F process.

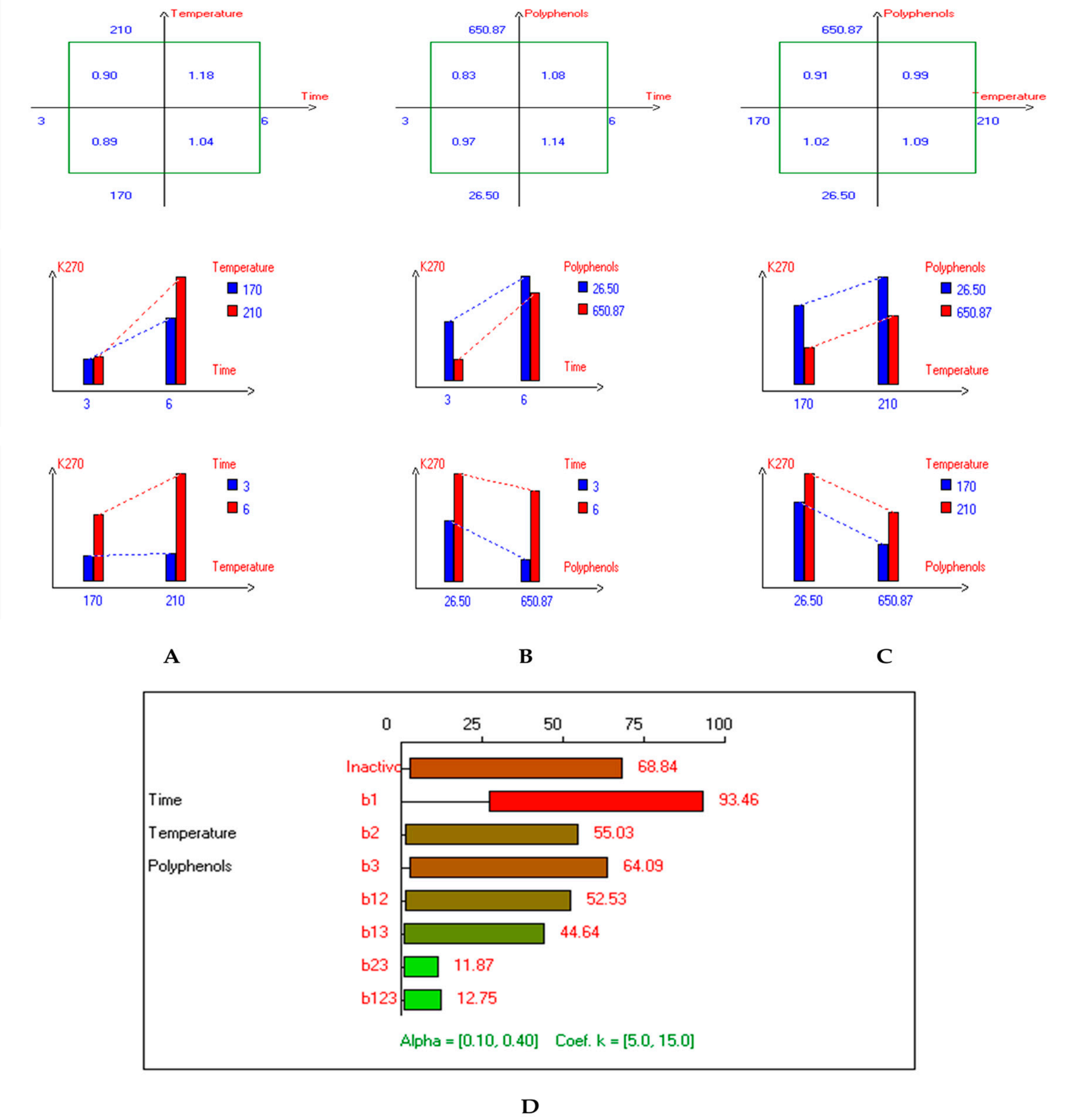

Figure S84. Combined interactions between the independent variables on a response variable (**K_{270}**) in **Olive oil 0.4°** under D-F: (**A**) x_1 and x_2, (**B**) x_1 and x_3, (**C**) x_2 and x_3, and (**D**) results of variance analysis of regression equation model and the significance changes in each individual independent variable and interaction between the combined independent variables on K_{270}; b represents a significant difference when be $>b_{123}$, while b represents no significant difference when be $\leq b_{123}$; b_1, b_2, b_3 are the main effects of the independent variables, while b_{12}, b_{13}, b_{23}, and b_{123} are the interaction effects of the independent variables. Moreover, x_1, x_2, and x_3 are coded variables (time, temperature, and polyphenols addition, respectively) for the experimental design in D-F process.

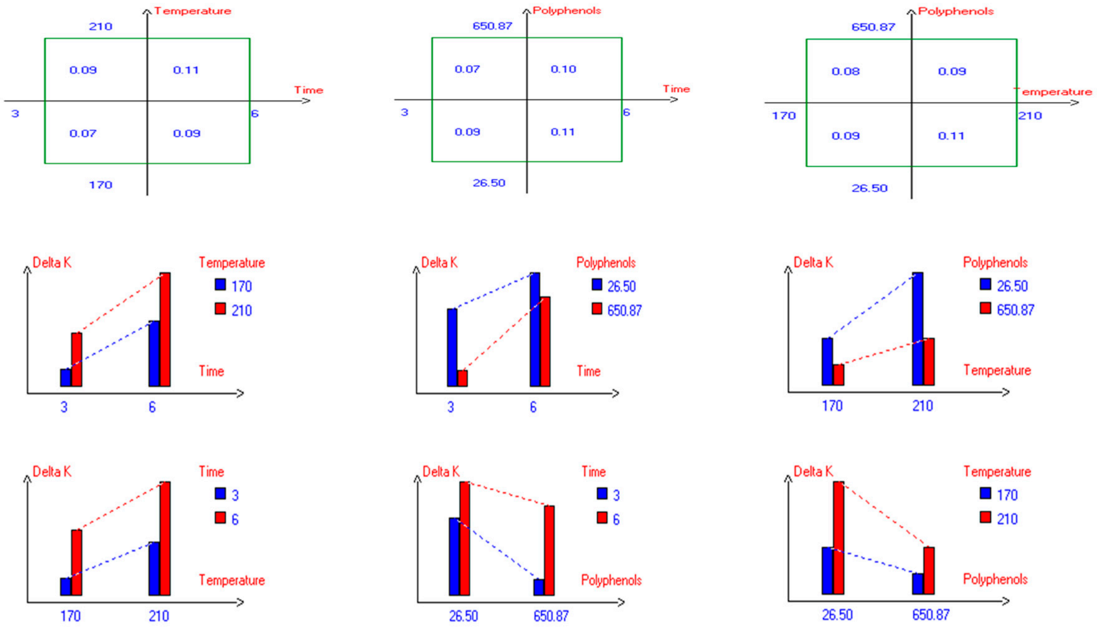

A B C

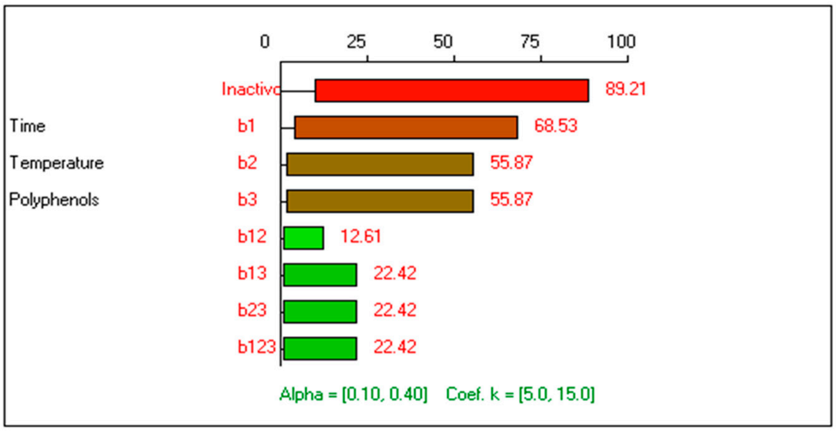

D

Figure S85. Combined interactions between the independent variables on a response variable (ΔK) in **Olive oil 0.4°** under D-F: (**A**) x_1 and x_2, (**B**) x_1 and x_3, (**C**) x_2 and x_3, and (**D**) results of variance analysis of regression equation model and the significance changes in each individual independent variable and interaction between the combined independent variables on ΔK; b represents a significant difference when be >b_{123}, while b represents no significant difference when be ≤b_{123}; b_1, b_2, b_3 are the main effects of the independent variables, while b_{12}, b_{13}, b_{23}, and b_{123} are the interaction effects of the independent variables. Moreover, x_l, x_2, and x_3 are coded variables (time, temperature, and polyphenols addition, respectively) for the experimental design in D-F process.

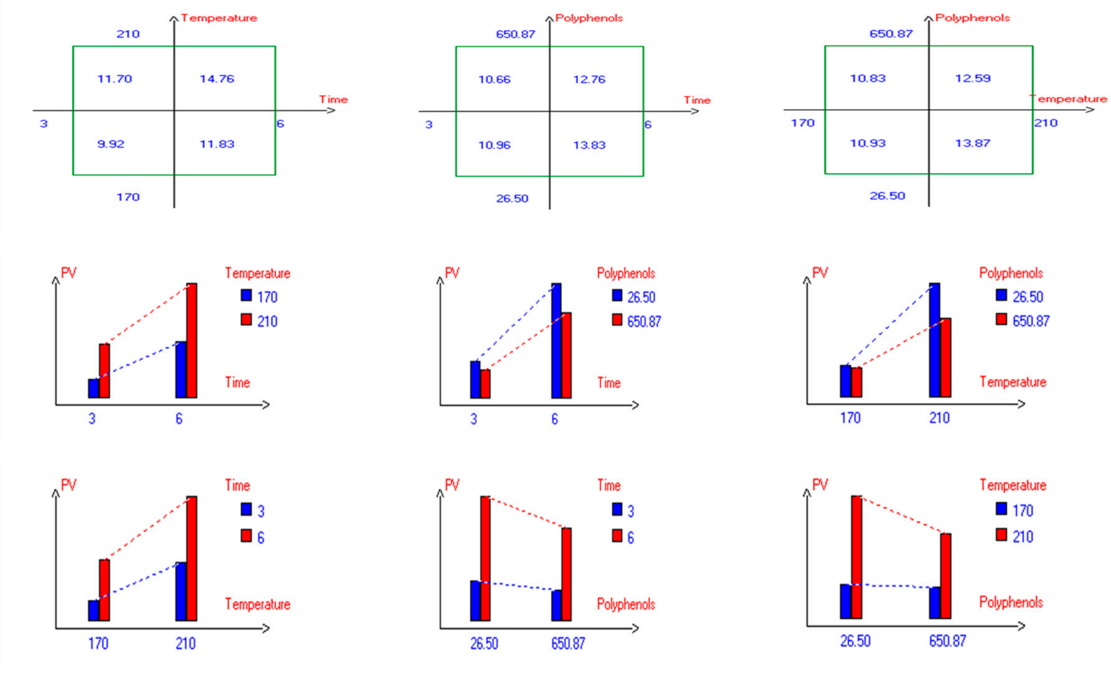

A **B** **C**

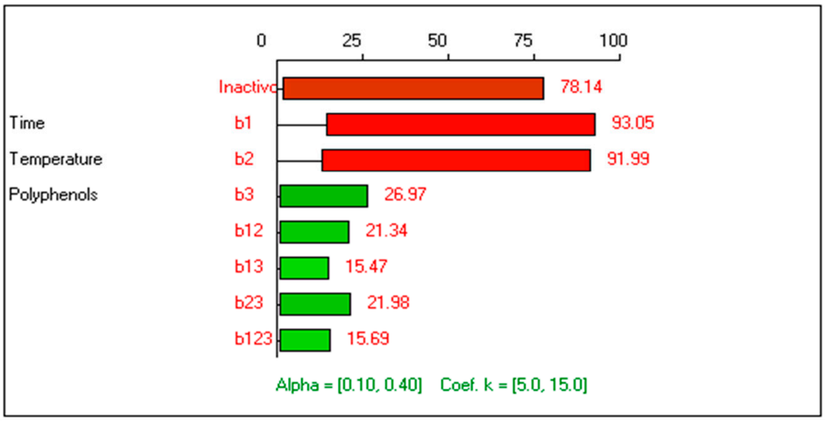

D

Figure S86. Combined interactions between the independent variables on a response variable (**peroxide value** ($mEqO_2$/kg)) in **Olive oil 0.4°** under D-F: (**A**) x_1 and x_2, (**B**) x_1 and x_3, (**C**) x_2 and x_3, and (**D**) results of variance analysis of regression equation model and the significance changes in each individual independent variable and interaction between the combined independent variables on peroxide value; b represents a significant difference when be $>b_{123}$, while b represents no significant difference when be $\leq b_{123}$; b_1, b_2, b_3 are the main effects of the independent variables, while b_{12}, b_{13}, b_{23}, and b_{123} are the interaction effects of the independent variables. Moreover, x_1, x_2, and x_3 are coded variables (time, temperature, and polyphenols addition, respectively) for the experimental design in D-F process.

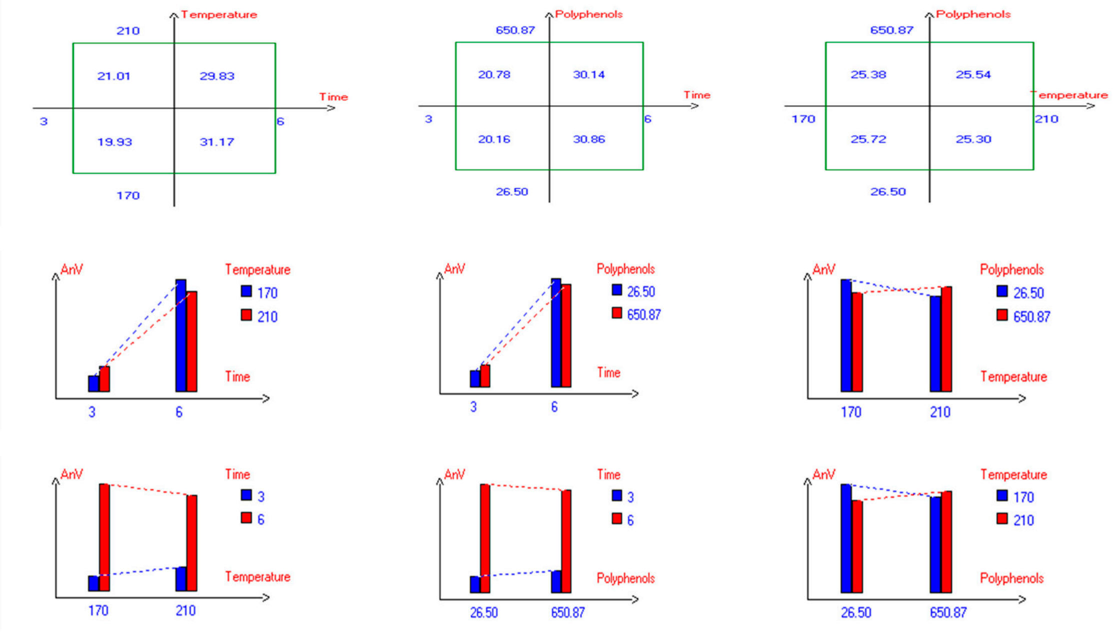

A **B** **C**

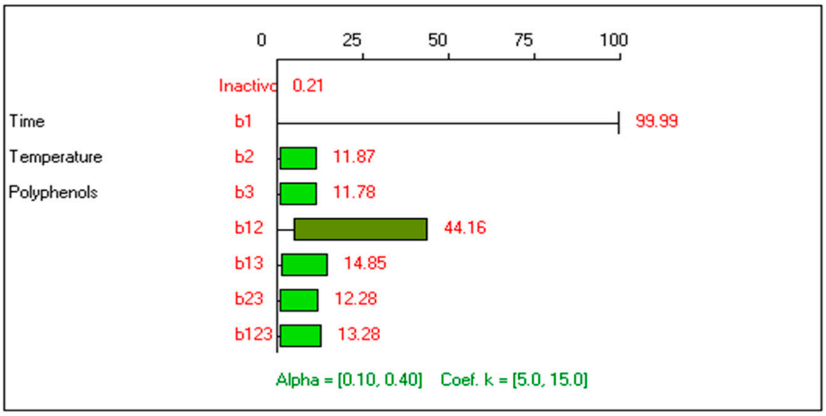

D

Figure S87. Combined interactions between the independent variables on a response variable (**anisidine value** (mg/kg)) in **Olive oil 0.4°** under D-F: (**A**) x_1 and x_2, (**B**) x_1 and x_3, (**C**) x_2 and x_3, and (**D**) results of variance analysis of regression equation model and the significance changes in each individual independent variable and interaction between the combined independent variables on anisidine value; b represents a significant difference when be $>b_{123}$, while b represents no significant difference when be $\leq b_{123}$; b_1, b_2, b_3 are the main effects of the independent variables, while b_{12}, b_{13}, b_{23}, and b_{123} are the interaction effects of the independent variables. Moreover, x_1, x_2, and x_3 are coded variables (time, temperature, and polyphenols addition, respectively) for the experimental design in D-F process.

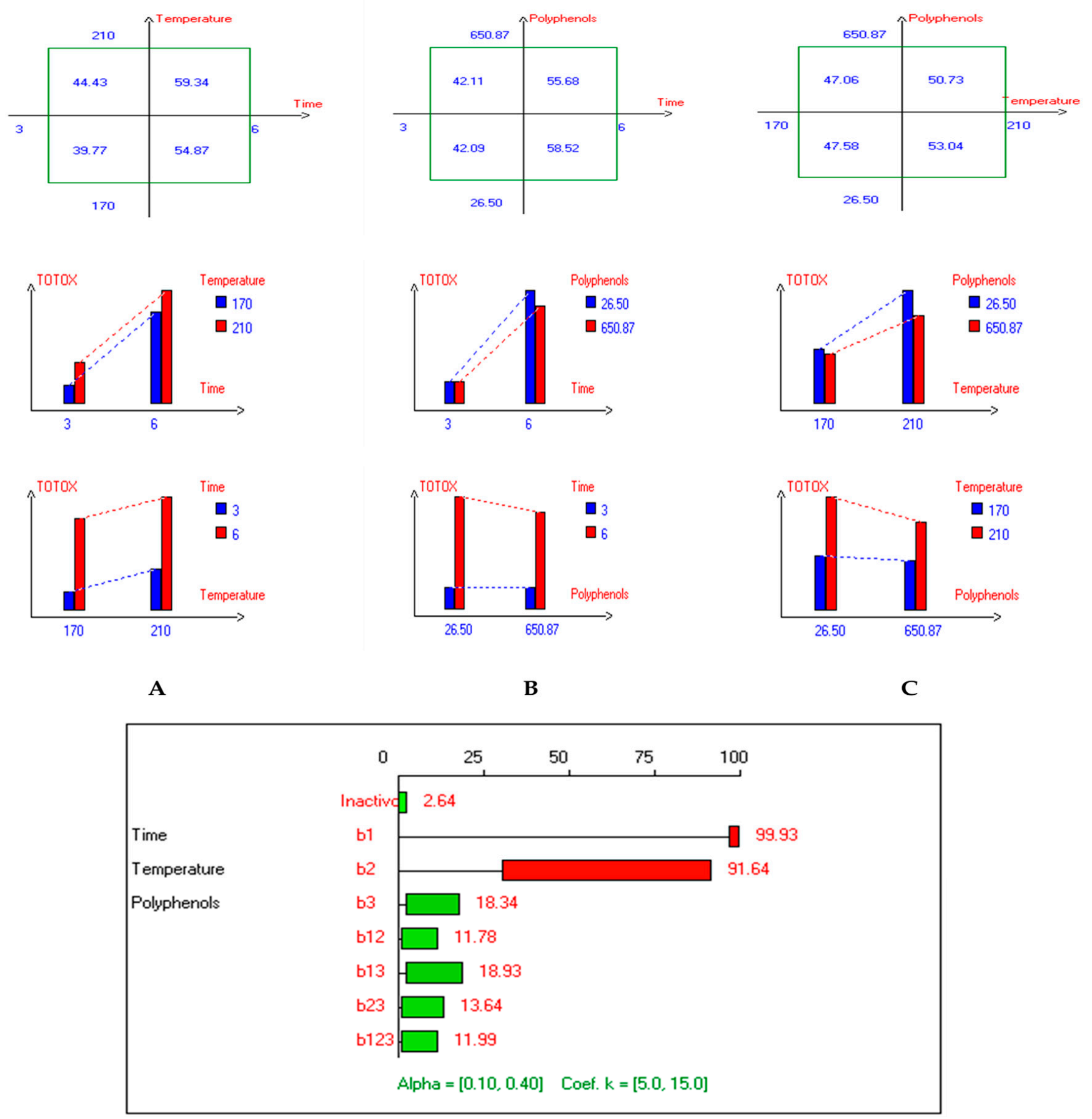

D

Figure S88. Combined interactions between the independent variables on a response variable (**TOTOX**) in **Olive oil 0.4°** under D-F: (**A**) x_1 and x_2, (**B**) x_1 and x_3, (**C**) x_2 and x_3, and (**D**) results of variance analysis of regression equation model and the significance changes in each individual independent variable and interaction between the combined independent variables on TOTOX; b represents a significant difference when be $>b_{123}$, while b represents no significant difference when be $\leq b_{123}$; b_1, b_2, b_3 are the main effects of the independent variables, while b_{12}, b_{13}, b_{23}, and b_{123} are the interaction effects of the independent variables. Moreover, x_1, x_2, and x_3 are coded variables (time, temperature, and polyphenols addition, respectively) for the experimental design in D-F process.

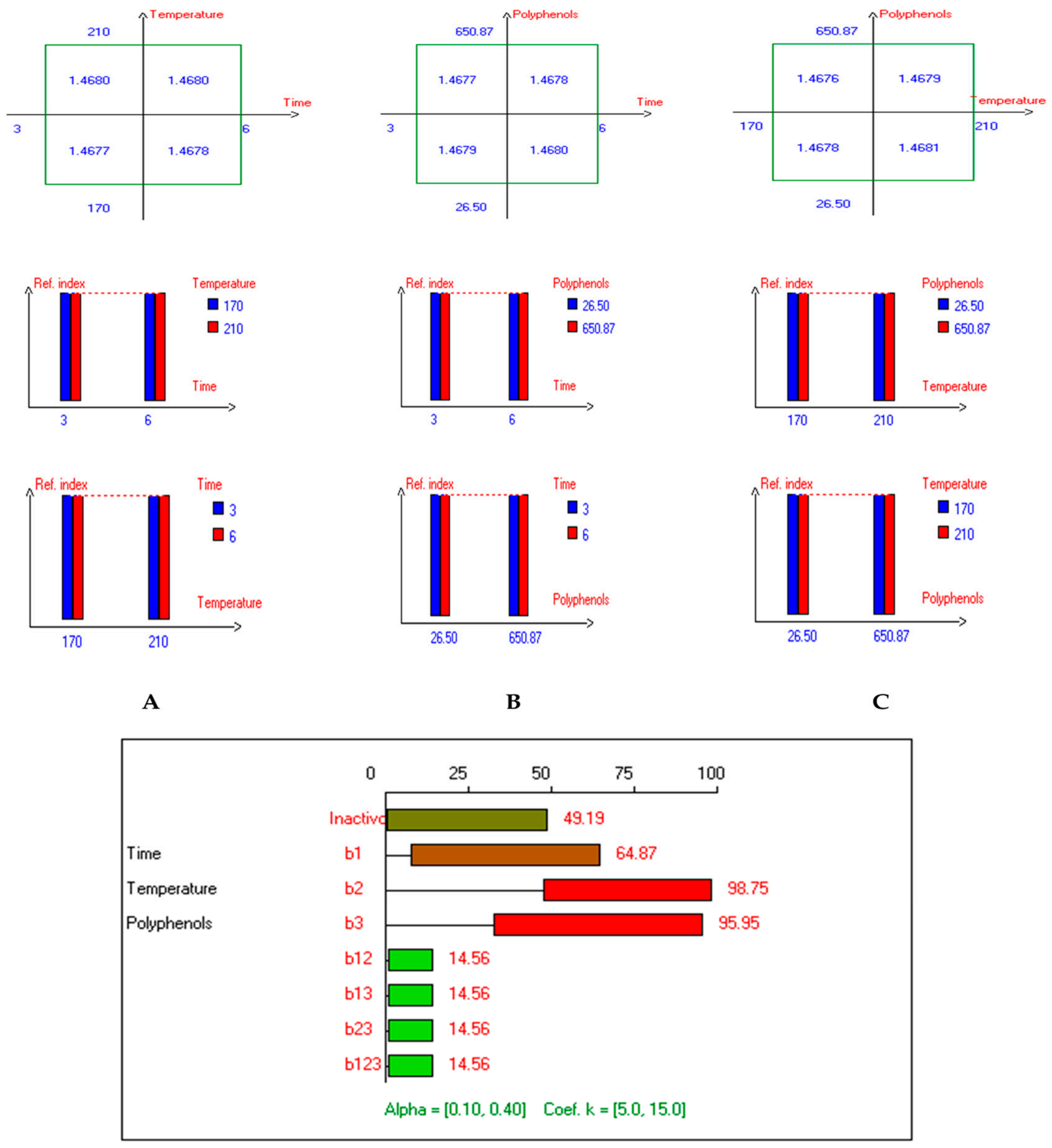

D

Figure S89. Combined interactions between the independent variables on a response variable (**refractive index**) in **Olive oil 0.4°** under D-F: (**A**) x_1 and x_2, (**B**) x_1 and x_3, (**C**) x_2 and x_3, and (**D**) results of variance analysis of regression equation model and the significance changes in each individual independent variable and interaction between the combined independent variables on refractive index; b represents a significant difference when be >b_{123}, while b represents no significant difference when be ≤b_{123}; b_1, b_2, b_3 are the main effects of the independent variables, while b_{12}, b_{13}, b_{23}, and b_{123} are the interaction effects of the independent variables. Moreover, x_I, x_2, and x_3 are coded variables (time, temperature, and polyphenols addition, respectively) for the experimental design in D-F process.

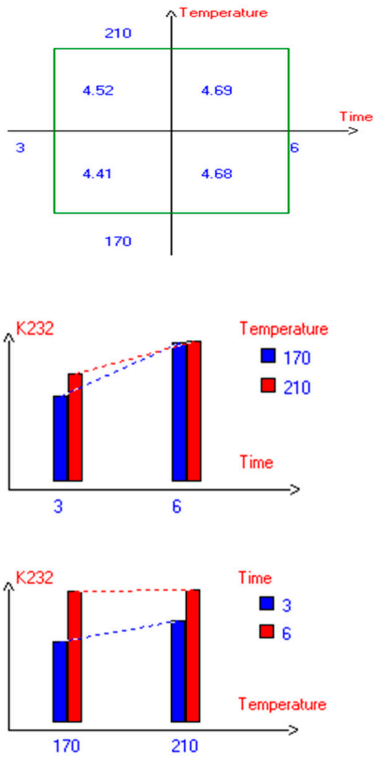

A

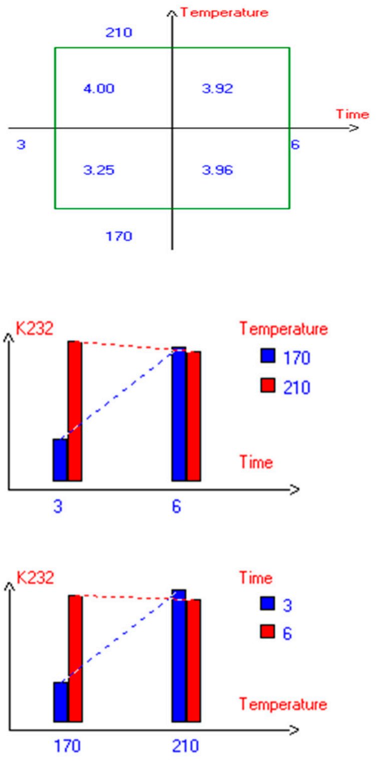

C

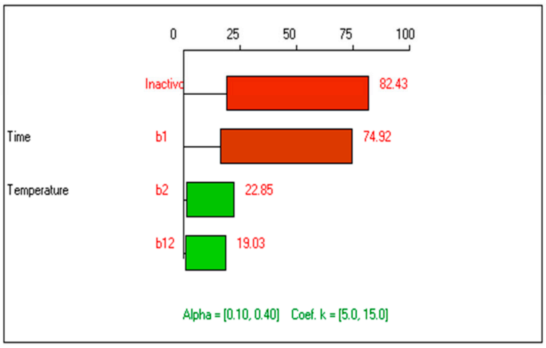

B

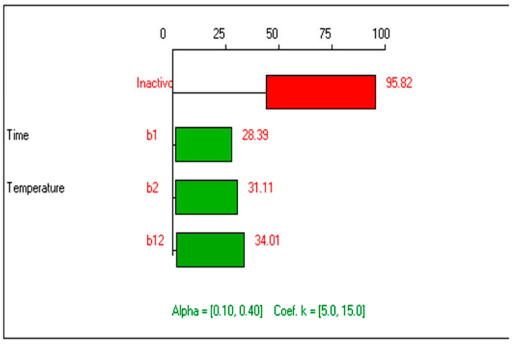

D

Figure S90. (A) Combined interactions between the independent variables (x_1 and x_2) on (**K_{232}**) in **sunflower oil**, **(B)** Results of variance analysis of regression equation model and the significance changes of each individual independent variable and interaction between the combined independent variables on acidity in sunflower oil. **(C)** Combined interactions between the independent variables (x_1 and x_2) on (**K_{232}**) in **sunflower oil-high oleic acid**, **(D)** Results of variance analysis of regression equation model and the significance changes of each individual independent variable and interaction between the combined independent variables on rancid score in sunflower oil-high oleic acid. Where, b represents significant difference when be $>b_{12}$; while b represents no significant difference when be $\leq b_{12}$. Moreover, b_1 and b_2 are the main effects of the independent variables, while b_{12} is the interaction effect of the independent variables. Where x_1: time, x_2: temperature.

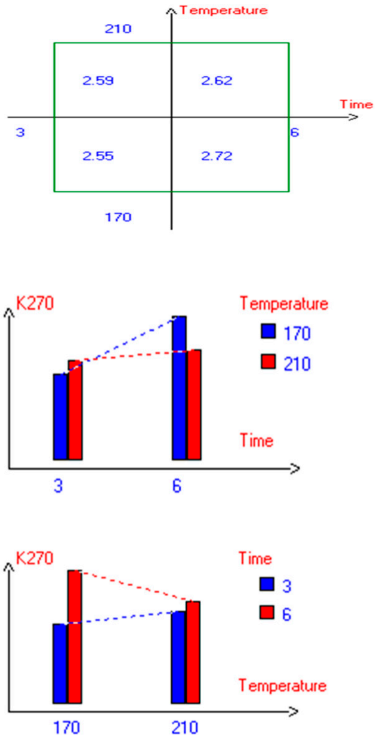

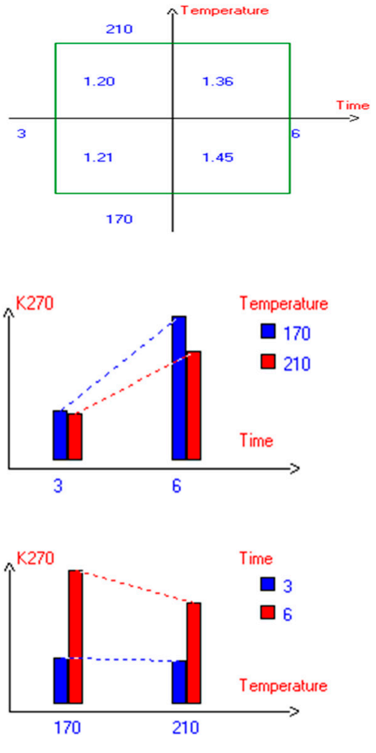

A **C**

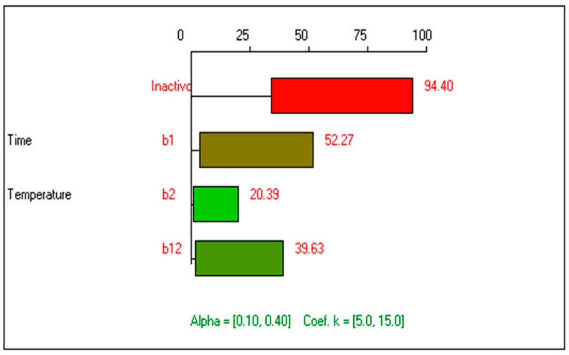

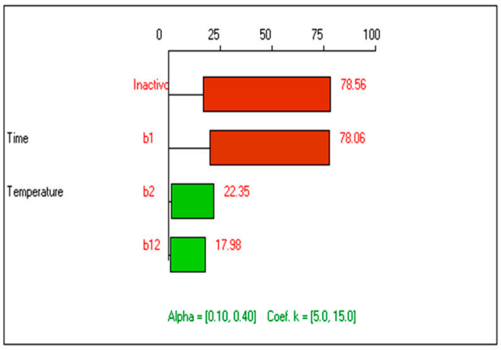

B **D**

Figure S91. (A) Combined interactions between the independent variables (x_1 and x_2) on (**K_{232}**) in **sunflower oil**, **(B)** Results of variance analysis of regression equation model and the significance changes of each individual independent variable and interaction between the combined independent variables on acidity in sunflower oil. **(C)** Combined interactions between the independent variables (x_1 and x_2) on (**K_{232}**) in **sunflower oil-high oleic acid**, **(D)** Results of variance analysis of regression equation model and the significance changes of each individual independent variable and interaction between the combined independent variables on rancid score in sunflower oil-high oleic acid. Where, b represents significant difference when be $>b_{12}$; while b represents no significant difference when be $\leq b_{12}$. Moreover, b_1 and b_2 are the main effects of the independent variables, while b_{12} is the interaction effect of the independent variables. Where x_1: time, x_2: temperature.

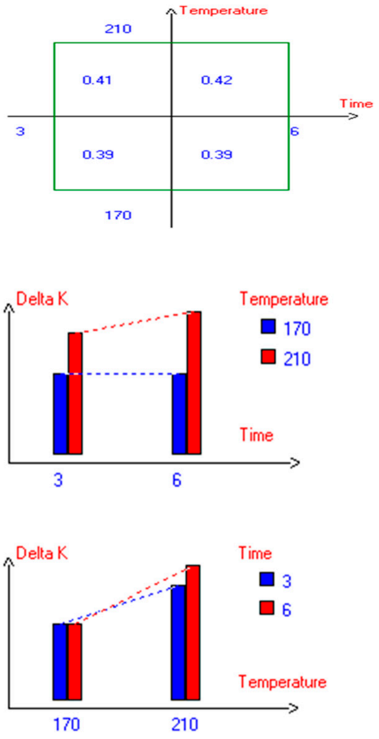

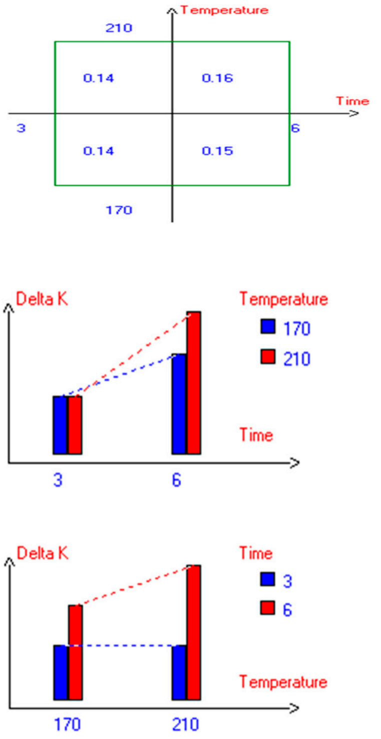

A **C**

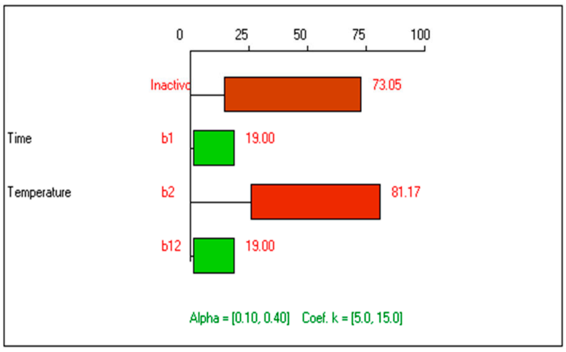

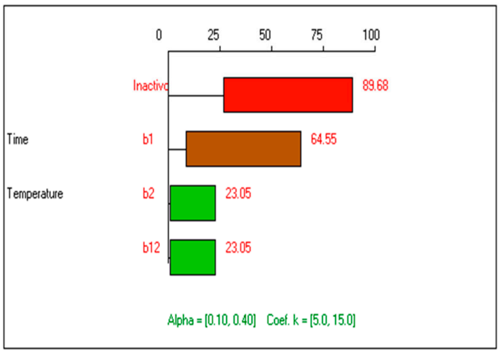

B **D**

Figure S92. (A) Combined interactions between the independent variables (x_1 and x_2) on (ΔK) in **sunflower oil**, **(B)** Results of variance analysis of regression equation model and the significance changes of each individual independent variable and interaction between the combined independent variables on acidity in sunflower oil. **(C)** Combined interactions between the independent variables (x_1 and x_2) on (ΔK) in **sunflower oil-high oleic acid**, **(D)** Results of variance analysis of regression equation model and the significance changes of each individual independent variable and interaction between the combined independent variables on rancid score in sunflower oil-high oleic acid. Where, b represents significant difference when be >b_{12}; while b represents no significant difference when be ≤b_{12}. Moreover, b_1 and b_2 are the main effects of the independent variables, while b_{12} is the interaction effect of the independent variables. Where x_1: time, x_2: temperature.

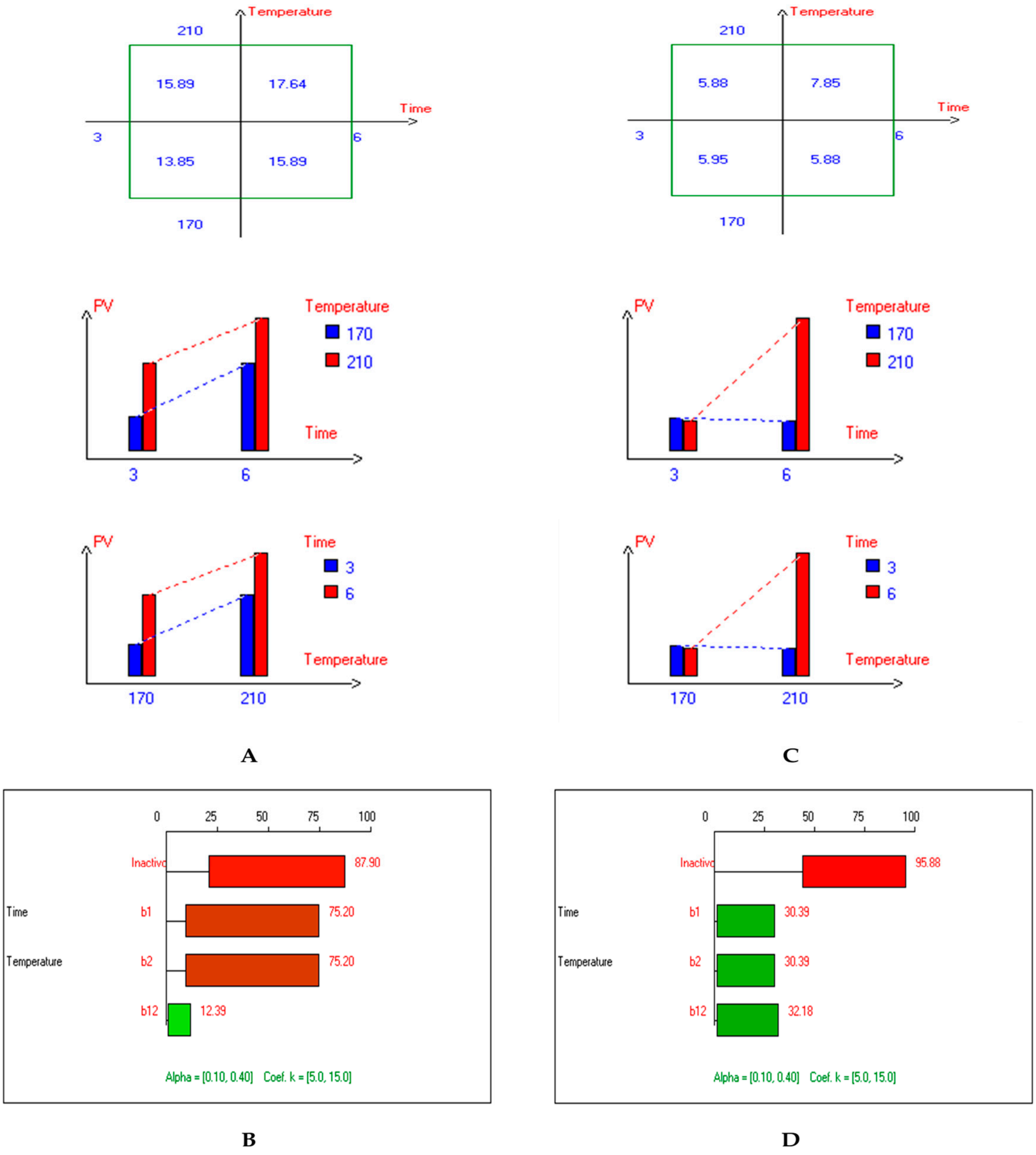

Figure S93. (A) Combined interactions between the independent variables (x_1 and x_2) on (**peroxide value** ($mEqO_2/kg$)) in **sunflower oil**, **(B)** Results of variance analysis of regression equation model and the significance changes of each individual independent variable and interaction between the combined independent variables on acidity in sunflower oil. **(C)** Combined interactions between the independent variables (x_1 and x_2) on (**peroxide value** ($mEqO_2/kg$)) in **sunflower oil-high oleic acid**, **(D)** Results of variance analysis of regression equation model and the significance changes of each individual independent variable and interaction between the combined independent variables on rancid score in sunflower oil-high oleic acid. Where, b represents significant difference when be $>b_{12}$; while b represents no significant difference when be $\leq b_{12}$. Moreover, b_1 and b_2 are the main effects of the independent variables, while b_{12} is the interaction effect of the independent variables. Where x_1: time, x_2: temperature.

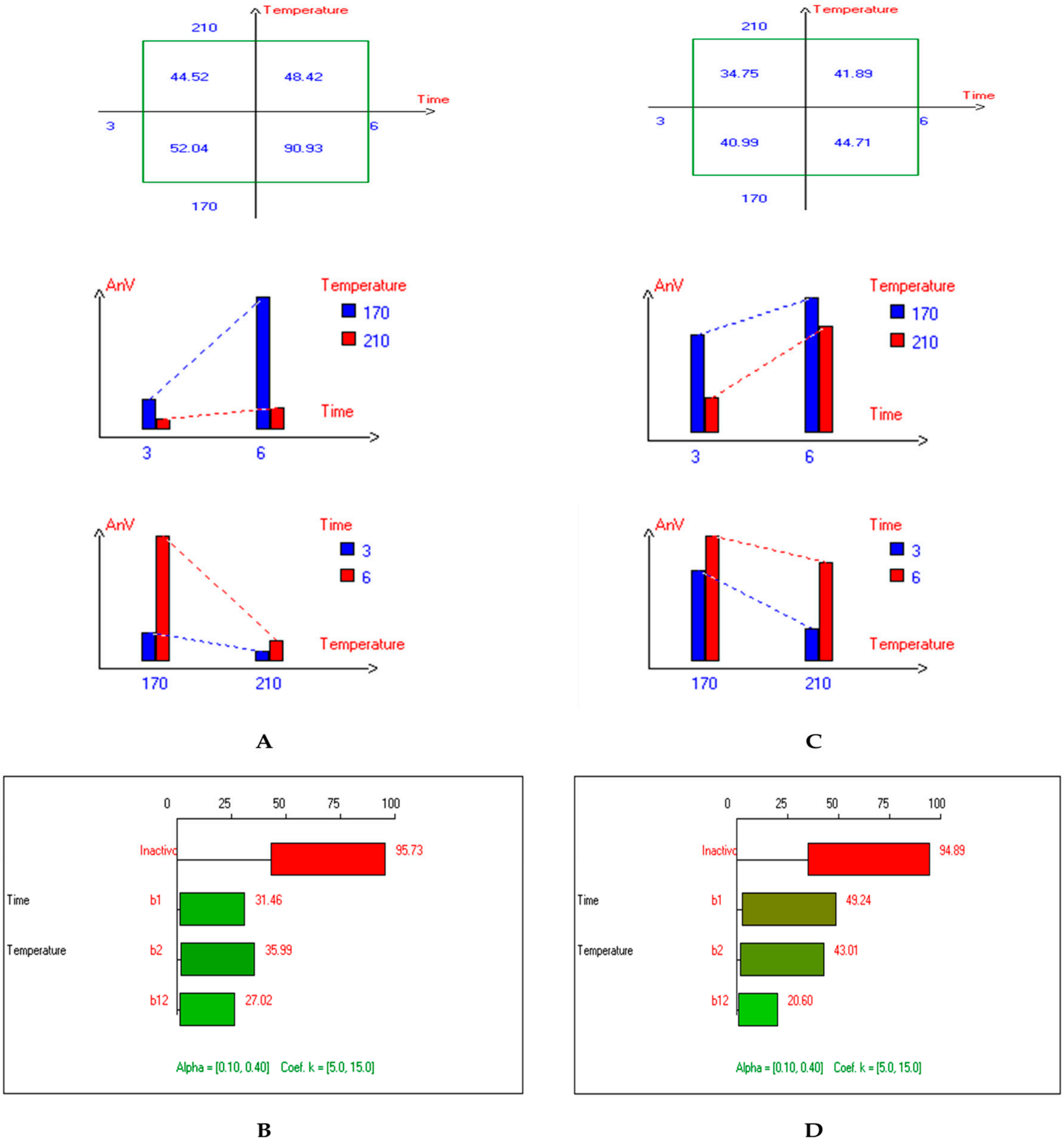

Figure S94. (A) Combined interactions between the independent variables (x_1 and x_2) on (**anisidine value** (mg/kg)) in **sunflower oil**, **(B)** Results of variance analysis of regression equation model and the significance changes of each individual independent variable and interaction between the combined independent variables on acidity in sunflower oil. **(C)** Combined interactions between the independent variables (x_1 and x_2) on (**anisidine value** (mg/kg)) in **sunflower oil-high oleic acid**, **(D)** Results of variance analysis of regression equation model and the significance changes of each individual independent variable and interaction between the combined independent variables on rancid score in sunflower oil-high oleic acid. Where, b represents significant difference when be $>b_{12}$; while b represents no significant difference when be $\leq b_{12}$. Moreover, b_1 and b_2 are the main effects of the independent variables, while b_{12} is the interaction effect of the independent variables. Where x_1: time, x_2: temperature.

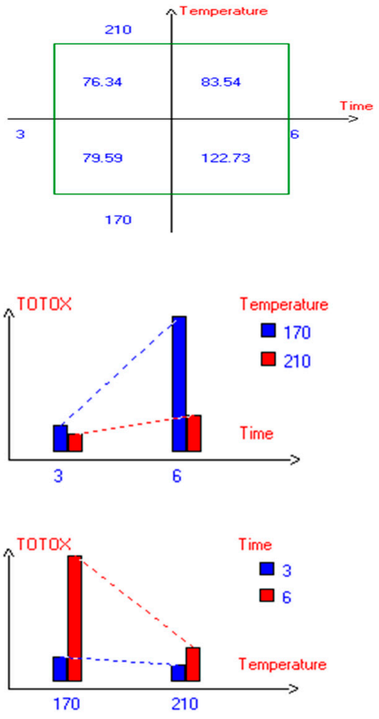

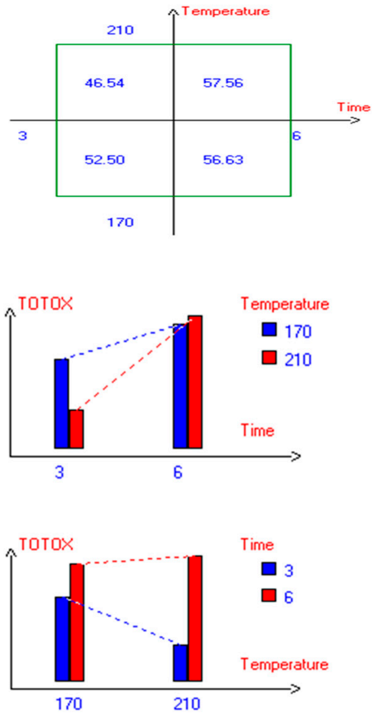

A

C

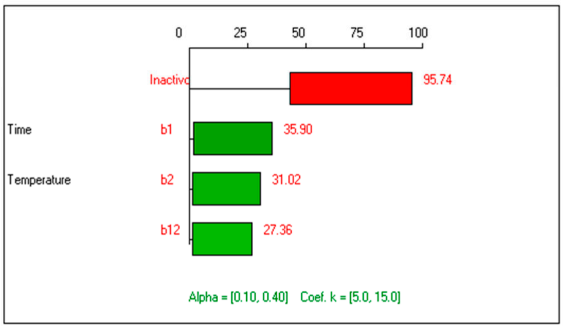

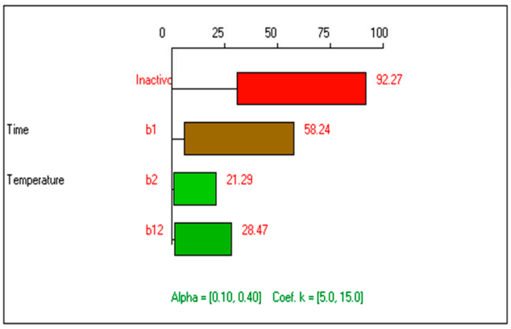

B

D

Figure S95. (A) Combined interactions between the independent variables (x_1 and x_2) on (**TOTOX**) in **sunflower oil**, **(B)** Results of variance analysis of regression equation model and the significance changes of each individual independent variable and interaction between the combined independent variables on acidity in sunflower oil. **(C)** Combined interactions between the independent variables (x_1 and x_2) on (**TOTOX**) in **sunflower oil-high oleic acid**, **(D)** Results of variance analysis of regression equation model and the significance changes of each individual independent variable and interaction between the combined independent variables on rancid score in sunflower oil-high oleic acid. Where, b represents significant difference when be $>b_{12}$; while b represents no significant difference when be $\leq b_{12}$. Moreover, b_1 and b_2 are the main effects of the independent variables, while b_{12} is the interaction effect of the independent variables. Where x_1: time, x_2: temperature.

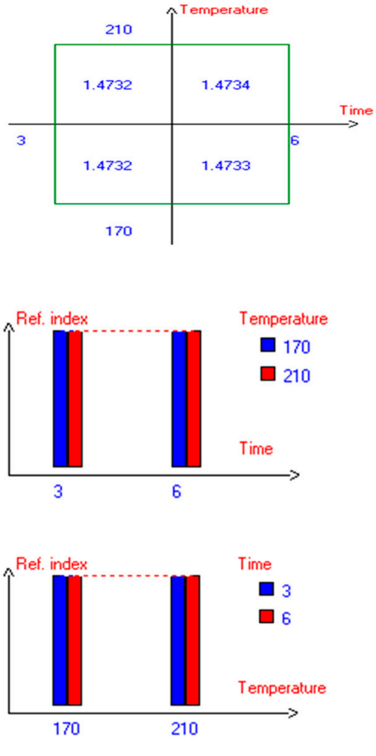

A

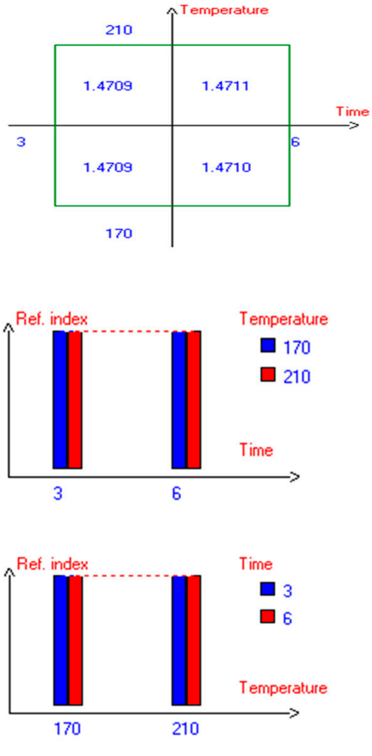

C

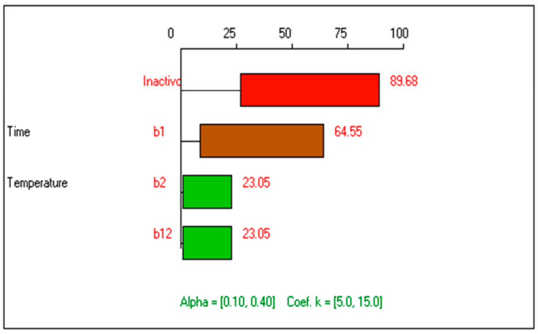

B

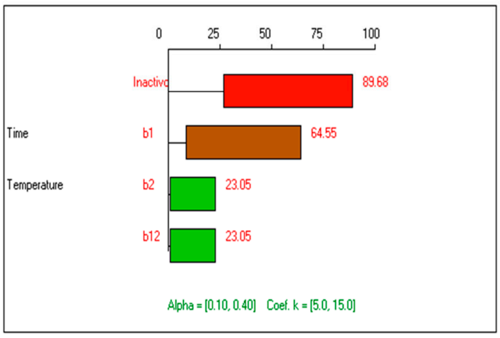

D

Figure S96. (A) Combined interactions between the independent variables (x_1 and x_2) on (**refractive index**) in **sunflower oil**, **(B)** Results of variance analysis of regression equation model and the significance changes of each individual independent variable and interaction between the combined independent variables on acidity in sunflower oil. **(C)** Combined interactions between the independent variables (x_1 and x_2) on (**refractive index**) in **sunflower oil-high oleic acid**, **(D)** Results of variance analysis of regression equation model and the significance changes of each individual independent variable and interaction between the combined independent variables on rancid score in sunflower oil-high oleic acid. Where, b represents significant difference when be $>b_{12}$; while b represents no significant difference when be $\leq b_{12}$. Moreover, b_1 and b_2 are the main effects of the independent variables, while b_{12} is the interaction effect of the independent variables. Where x_1: time, x_2: temperature.

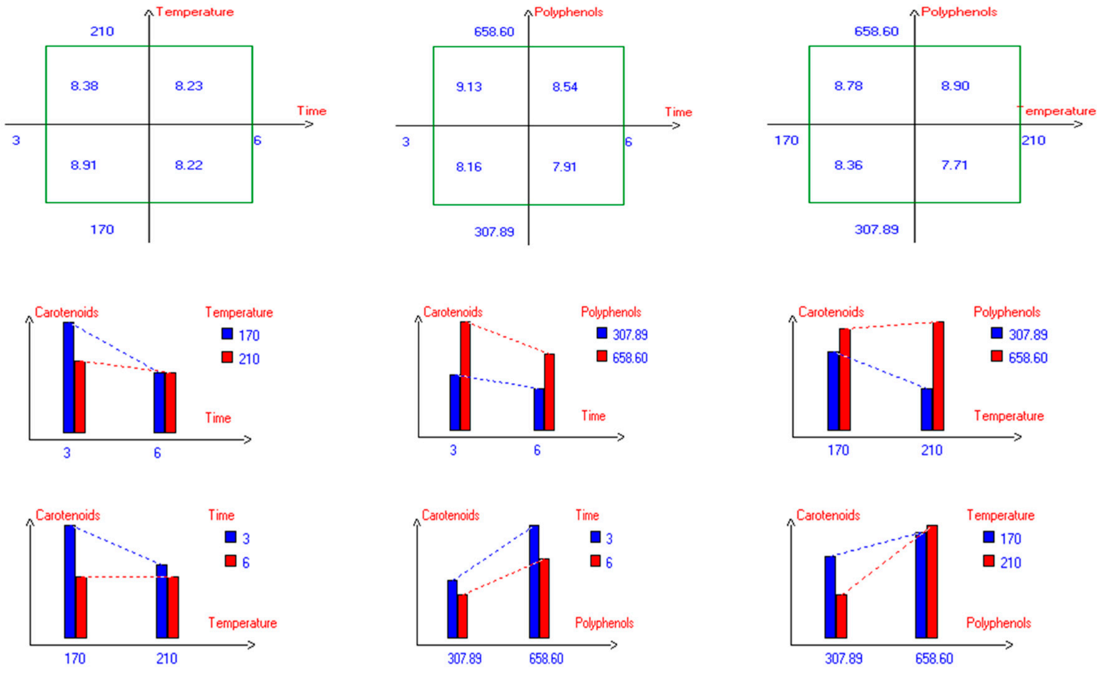

A **B** **C**

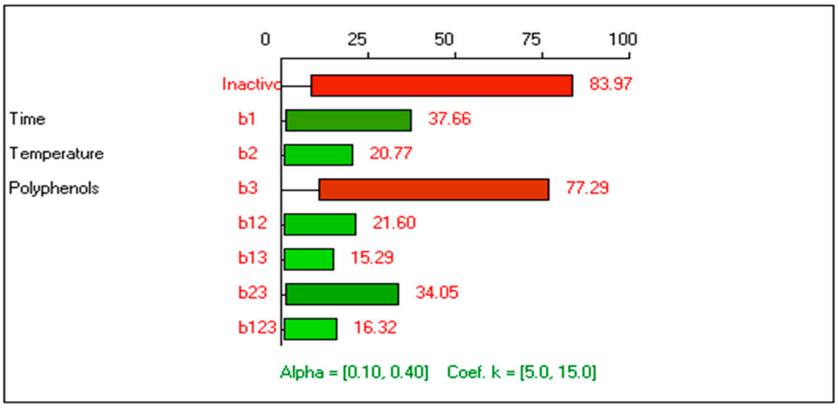

D

Figure S97. Combined interactions between the independent variables on a response variable (**carotenoids** (mg/kg)) in EVOO **Picual** under D-F: (**A**) x_1 and x_2, (**B**) x_1 and x_3, (**C**) x_2 and x_3, and (**D**) results of variance analysis of regression equation model and the significance changes in each individual independent variable and interaction between the combined independent variables on carotenoids; b represents a significant difference when be $>b_{123}$, while b represents no significant difference when be $\leq b_{123}$; b_1, b_2, b_3 are the main effects of the independent variables, while b_{12}, b_{13}, b_{23}, and b_{123} are the interaction effects of the independent variables. Moreover, x_1, x_2, and x_3 are coded variables (time, temperature, and polyphenols addition, respectively) for the experimental design in D-F process.

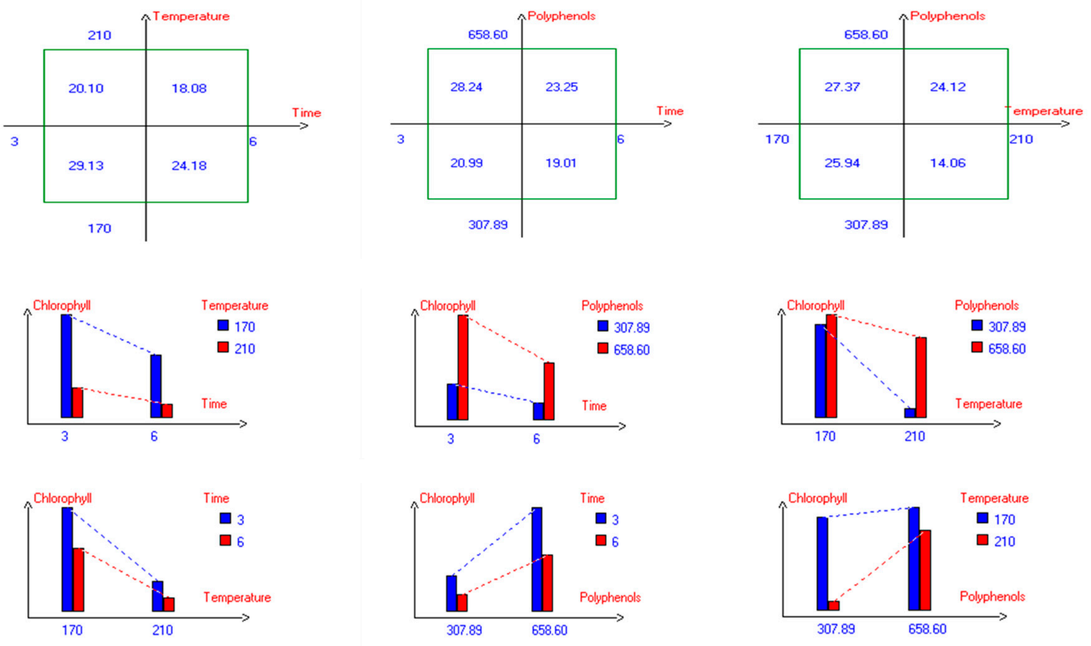

A **B** **C**

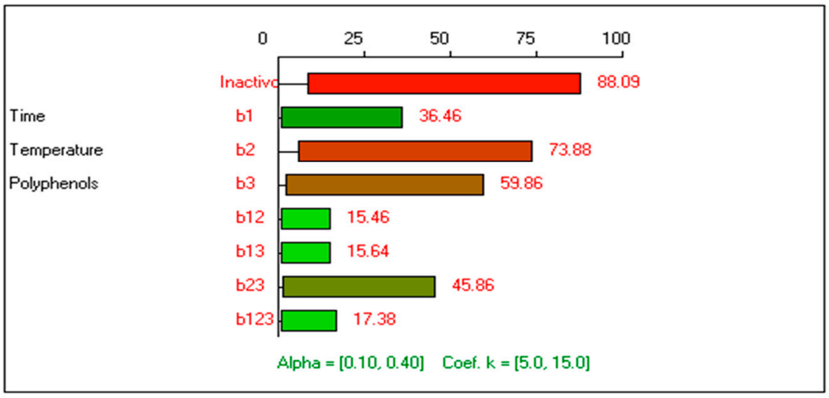

D

Figure S98. Combined interactions between the independent variables on a response variable (**chlorophyll** (mg/kg)) in EVOO **Picual** under D-F: (**A**) x_1 and x_2, (**B**) x_1 and x_3, (**C**) x_2 and x_3, and (**D**) results of variance analysis of regression equation model and the significance changes in each individual independent variable and interaction between the combined independent variables on chlorophyll; b represents a significant difference when be $>b_{123}$, while b represents no significant difference when be $\leq b_{123}$; b_1, b_2, b_3 are the main effects of the independent variables, while b_{12}, b_{13}, b_{23}, and b_{123} are the interaction effects of the independent variables. Moreover, x_I, x_2, and x_3 are coded variables (time, temperature, and polyphenols addition, respectively) for the experimental design in D-F process.

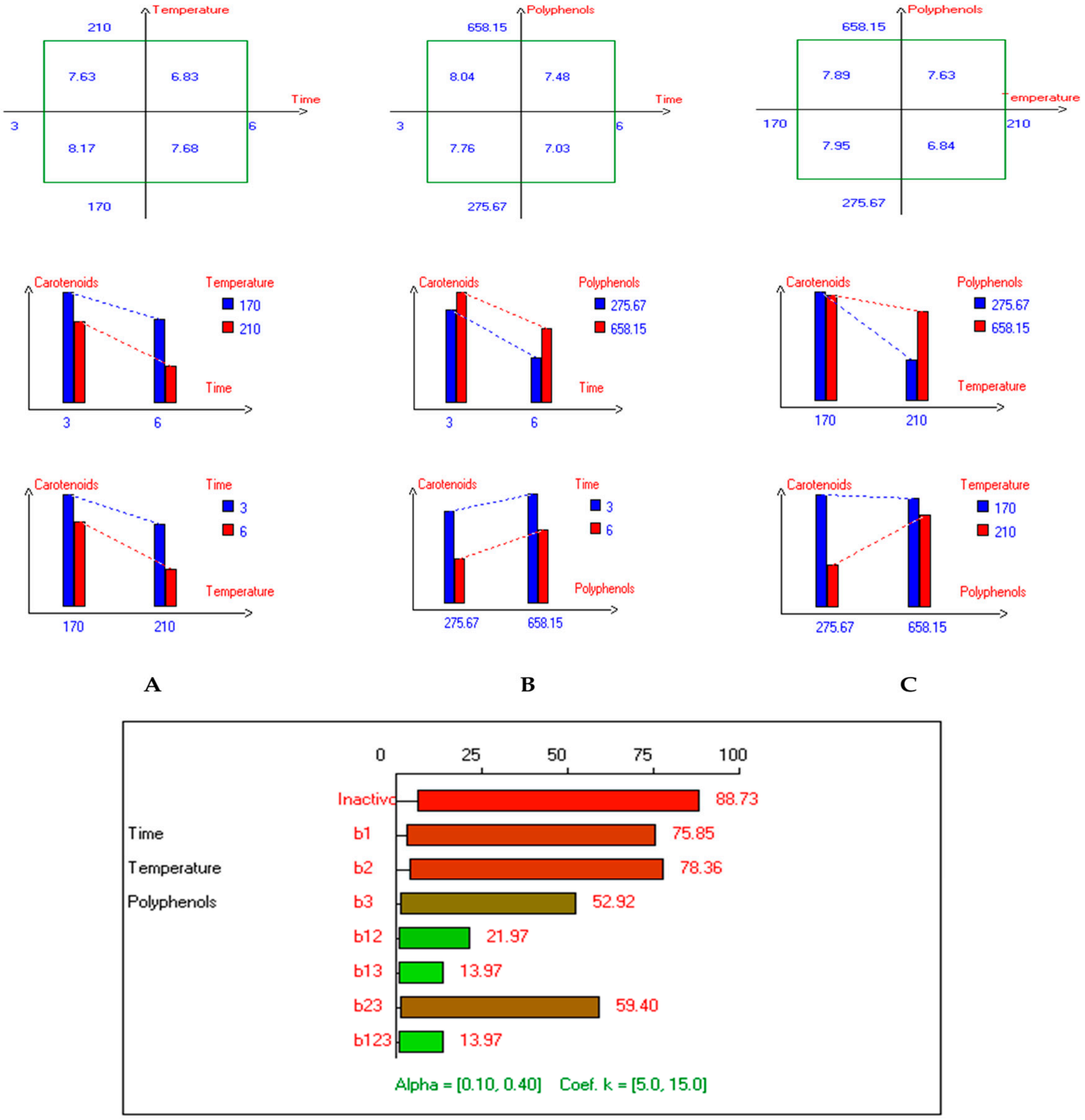

D

Figure S99. Combined interactions between the independent variables on a response variable (**carotenoids** (mg/kg)) in EVOO **Cornicabra** under D-F: (**A**) x_1 and x_2, (**B**) x_1 and x_3, (**C**) x_2 and x_3, and (**D**) results of variance analysis of regression equation model and the significance changes in each individual independent variable and interaction between the combined independent variables on carotenoids; b represents a significant difference when be $>b_{123}$, while b represents no significant difference when be $\leq b_{123}$; b_1, b_2, b_3 are the main effects of the independent variables, while b_{12}, b_{13}, b_{23}, and b_{123} are the interaction effects of the independent variables. Moreover, x_1, x_2, and x_3 are coded variables (time, temperature, and polyphenols addition, respectively) for the experimental design in D-F process.

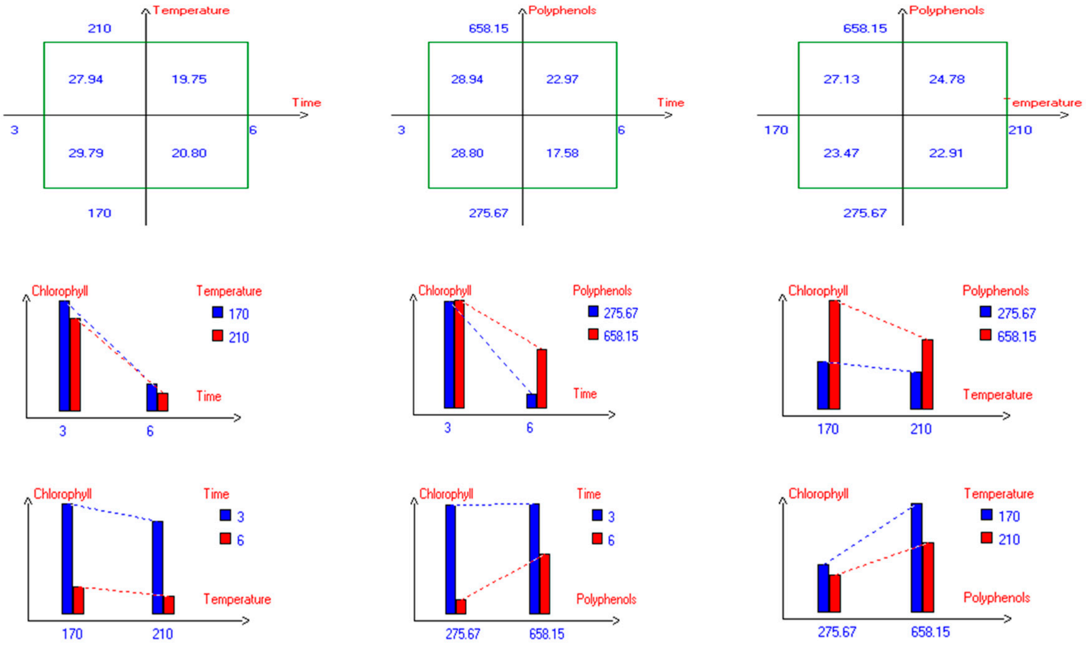

A **B** **C**

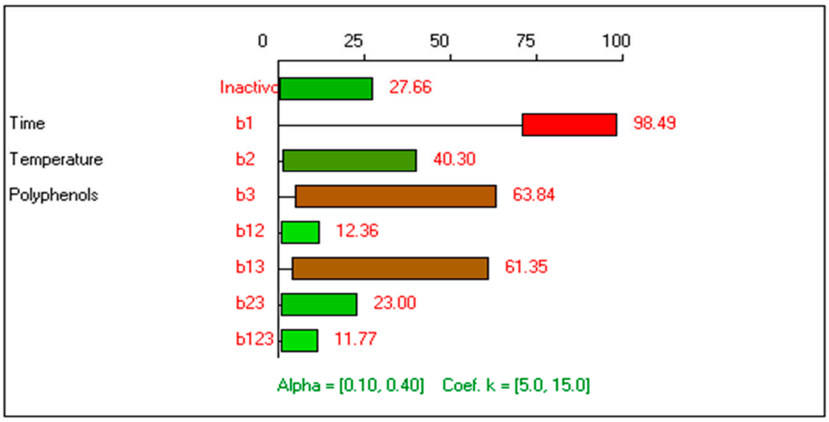

D

Figure S100. Combined interactions between the independent variables on a response variable (**chlorophyll** (mg/kg)) in EVOO **Cornicabra** under D-F: (**A**) x_1 and x_2, (**B**) x_1 and x_3, (**C**) x_2 and x_3, and (**D**) results of variance analysis of regression equation model and the significance changes in each individual independent variable and interaction between the combined independent variables on chlorophyll; b represents a significant difference when be $>b_{123}$, while b represents no significant difference when be $\leq b_{123}$; b_1, b_2, b_3 are the main effects of the independent variables, while b_{12}, b_{13}, b_{23}, and b_{123} are the interaction effects of the independent variables. Moreover, x_1, x_2, and x_3 are coded variables (time, temperature, and polyphenols addition, respectively) for the experimental design in D-F process.

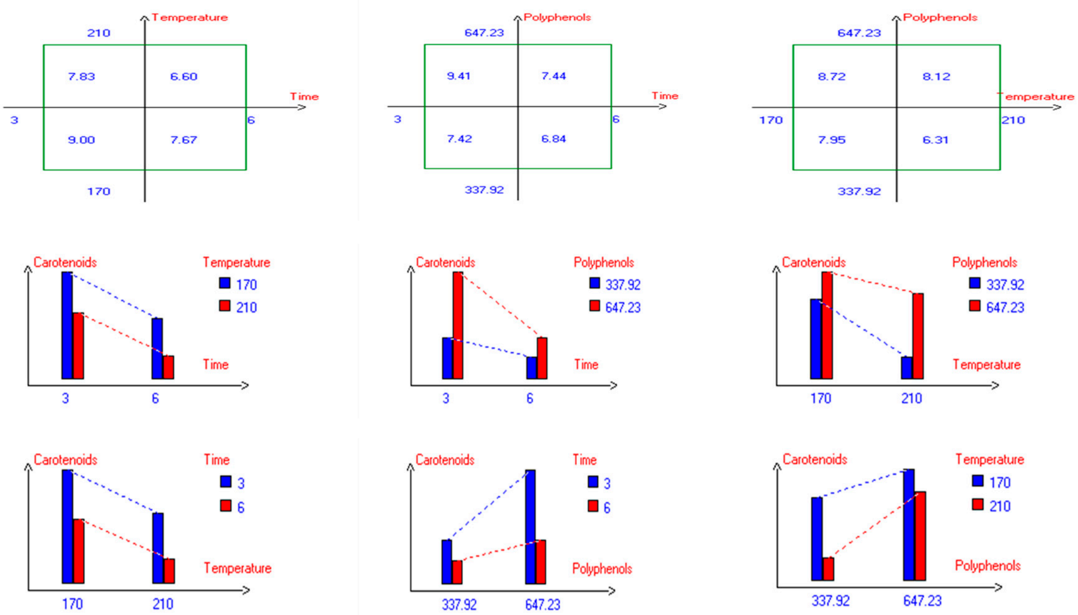

A **B** **C**

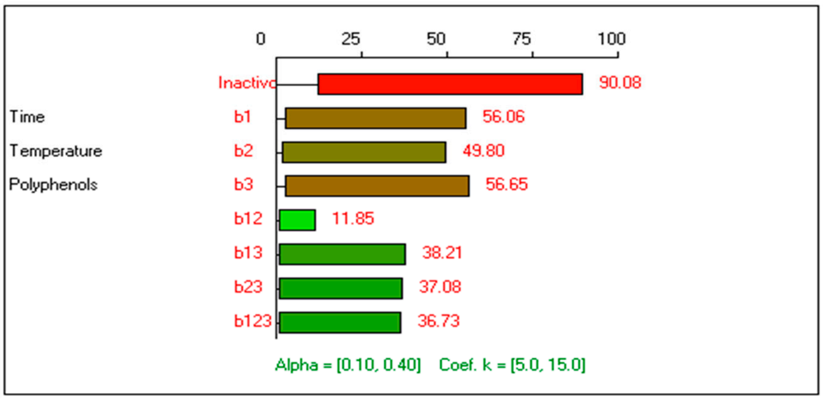

D

Figure S101. Combined interactions between the independent variables on a response variable (**carotenoids** (mg/kg)) in EVOO **Empeltre** under D-F: (**A**) x_1 and x_2, (**B**) x_1 and x_3, (**C**) x_2 and x_3, and (**D**) results of variance analysis of regression equation model and the significance changes in each individual independent variable and interaction between the combined independent variables on carotenoids; b represents a significant difference when be $>b_{123}$, while b represents no significant difference when be $\leq b_{123}$; b_1, b_2, b_3 are the main effects of the independent variables, while b_{12}, b_{13}, b_{23}, and b_{123} are the interaction effects of the independent variables. Moreover, x_1, x_2, and x_3 are coded variables (time, temperature, and polyphenols addition, respectively) for the experimental design in D-F process.

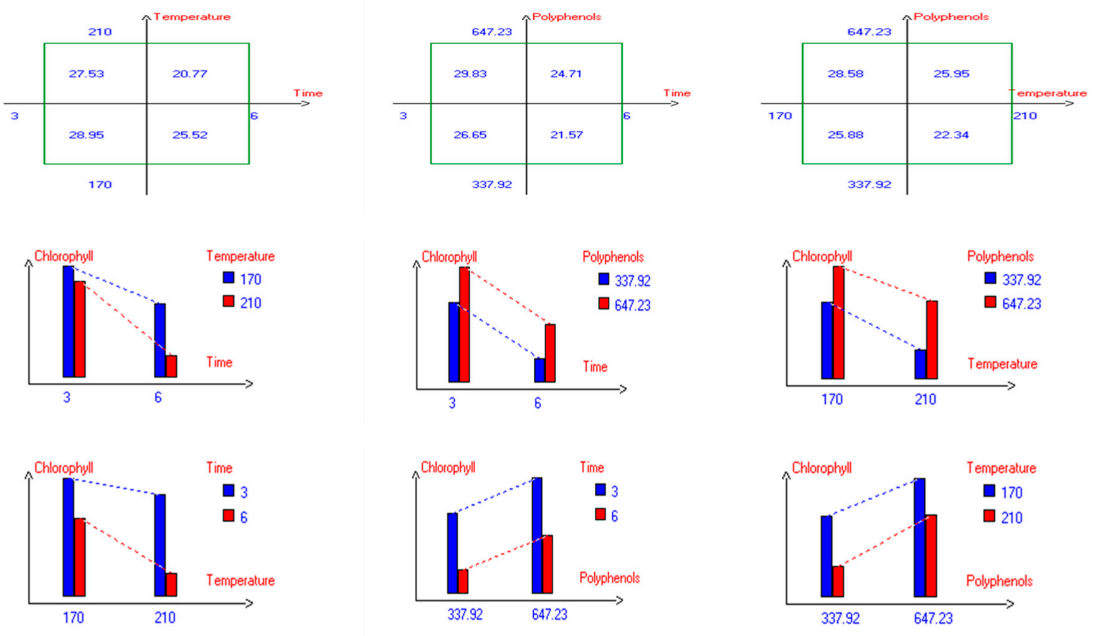

A **B** **C**

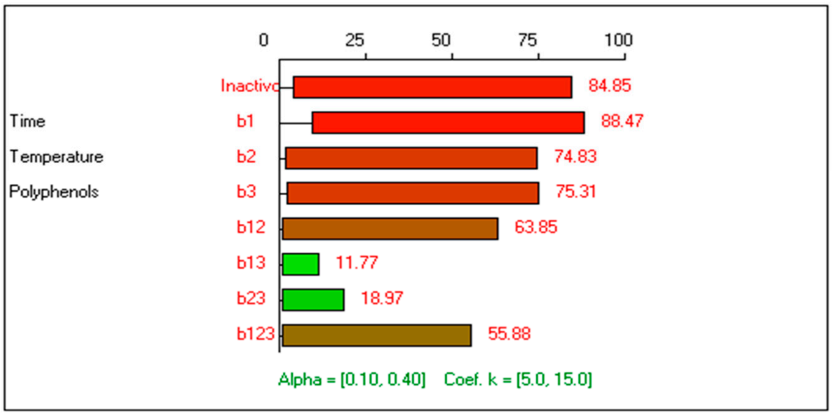

D

Figure S102. Combined interactions between the independent variables on a response variable (**chlorophyll** (mg/kg)) in EVOO **Empeltre** under D-F: (**A**) x_1 and x_2, (**B**) x_1 and x_3, (**C**) x_2 and x_3, and (**D**) results of variance analysis of regression equation model and the significance changes in each individual independent variable and interaction between the combined independent variables on chlorophyll; b represents a significant difference when be $>b_{123}$, while b represents no significant difference when be $\leq b_{123}$; b_1, b_2, b_3 are the main effects of the independent variables, while b_{12}, b_{13}, b_{23}, and b_{123} are the interaction effects of the independent variables. Moreover, x_1, x_2, and x_3 are coded variables (time, temperature, and polyphenols addition, respectively) for the experimental design in D-F process.

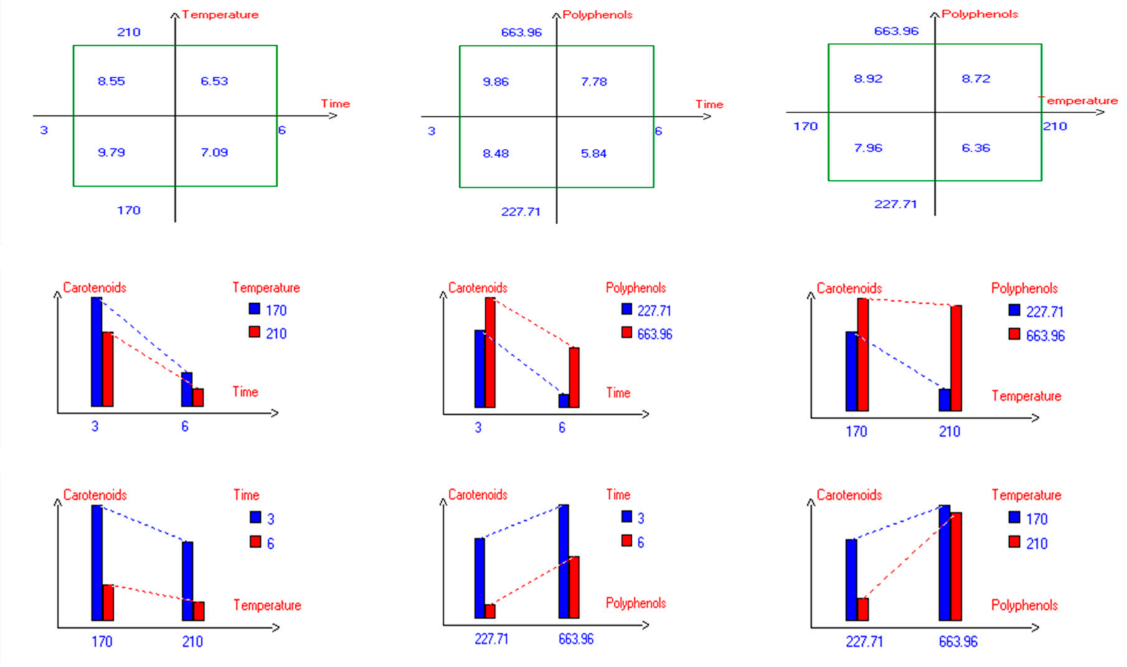

A **B** **C**

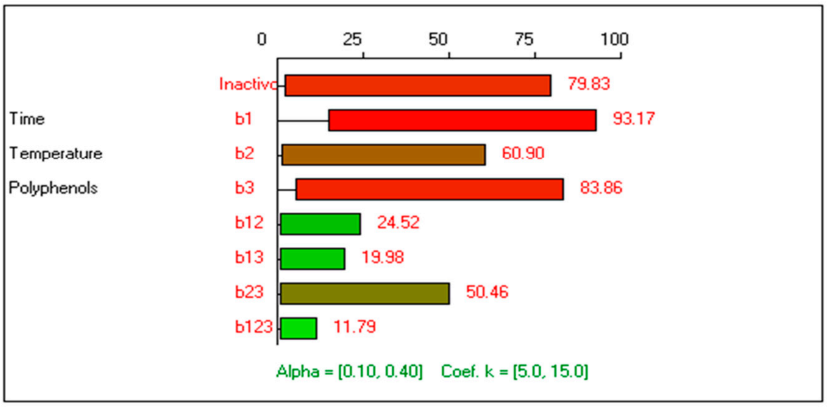

D

Figure S103. Combined interactions between the independent variables on a response variable (**carotenoids** (mg/kg)) in EVOO **Arbequina** under D-F: (**A**) x_1 and x_2, (**B**) x_1 and x_3, (**C**) x_2 and x_3, and (**D**) results of variance analysis of regression equation model and the significance changes in each individual independent variable and interaction between the combined independent variables on carotenoids; b represents a significant difference when be >b_{123}, while b represents no significant difference when be ≤b_{123}; b_1, b_2, b_3 are the main effects of the independent variables, while b_{12}, b_{13}, b_{23}, and b_{123} are the interaction effects of the independent variables. Moreover, x_I, x_2, and x_3 are coded variables (time, temperature, and polyphenols addition, respectively) for the experimental design in D-F process.

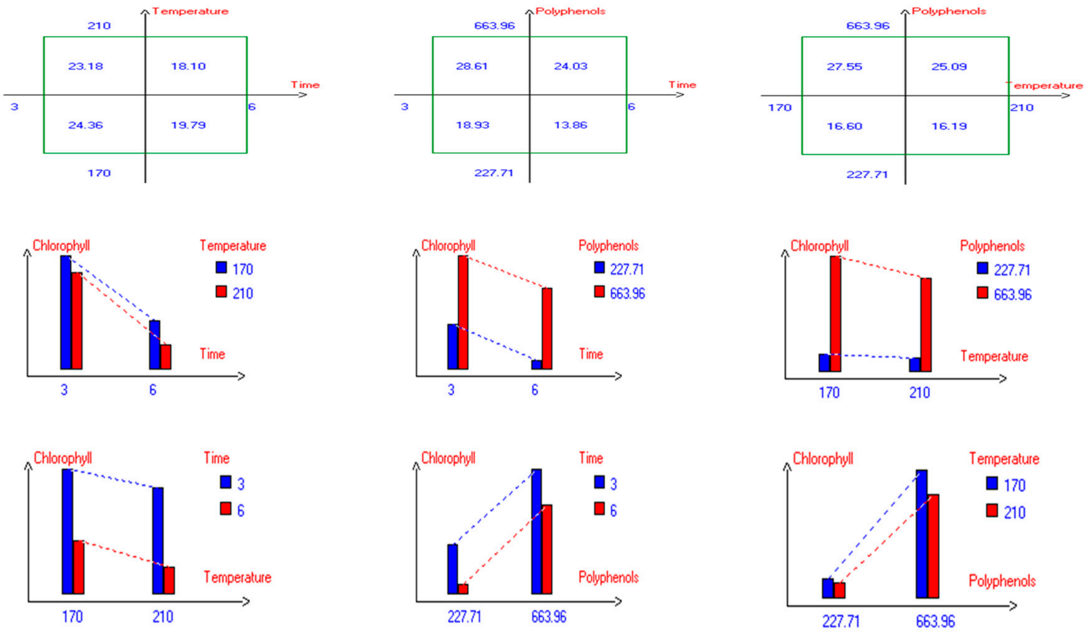

A B C

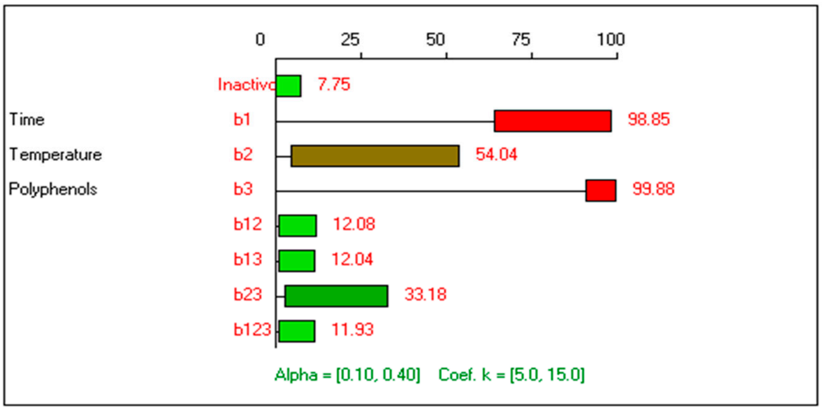

D

Figure S104. Combined interactions between the independent variables on a response variable (**chlorophyll** (mg/kg)) in EVOO **Arbequina** under D-F: (**A**) x_1 and x_2, (**B**) x_1 and x_3, (**C**) x_2 and x_3, and (**D**) results of variance analysis of regression equation model and the significance changes in each individual independent variable and interaction between the combined independent variables on chlorophyll; b represents a significant difference when be $>b_{123}$, while b represents no significant difference when be $\leq b_{123}$; b_1, b_2, b_3 are the main effects of the independent variables, while b_{12}, b_{13}, b_{23}, and b_{123} are the interaction effects of the independent variables. Moreover, x_1, x_2, and x_3 are coded variables (time, temperature, and polyphenols addition, respectively) for the experimental design in D-F process.

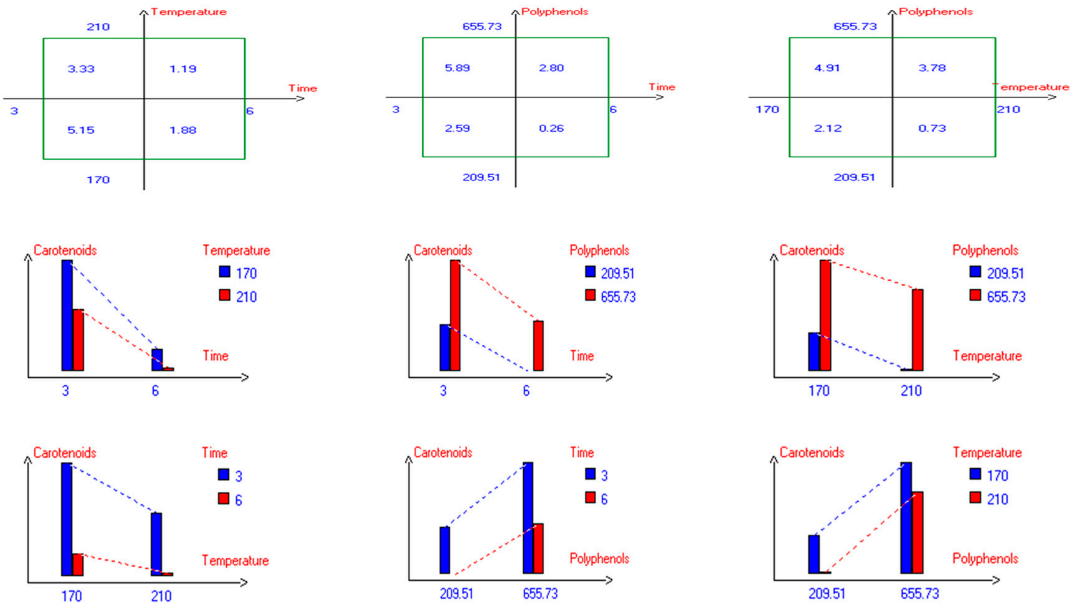

A **B** **C**

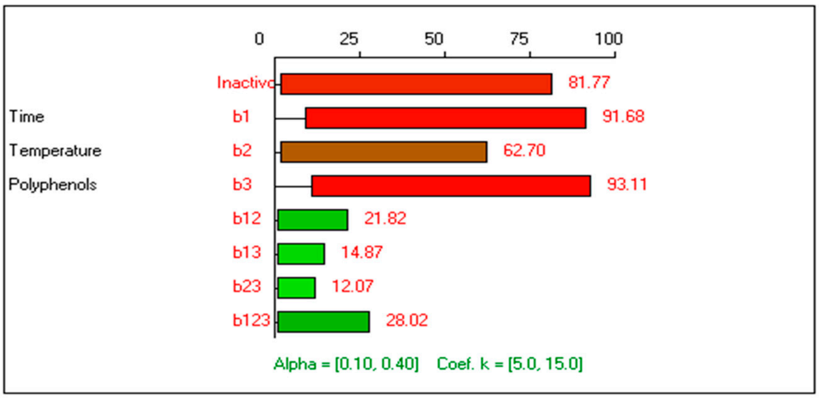

D

Figure S105. Combined interactions between the independent variables on a response variable (**carotenoids** (mg/kg)) in EVOO **Hojiblanca** under D-F: (**A**) x_1 and x_2, (**B**) x_1 and x_3, (**C**) x_2 and x_3, and (**D**) results of variance analysis of regression equation model and the significance changes in each individual independent variable and interaction between the combined independent variables on carotenoids; b represents a significant difference when be $>b_{123}$, while b represents no significant difference when be $\leq b_{123}$; b_1, b_2, b_3 are the main effects of the independent variables, while b_{12}, b_{13}, b_{23}, and b_{123} are the interaction effects of the independent variables. Moreover, x_1, x_2, and x_3 are coded variables (time, temperature, and polyphenols addition, respectively) for the experimental design in D-F process.

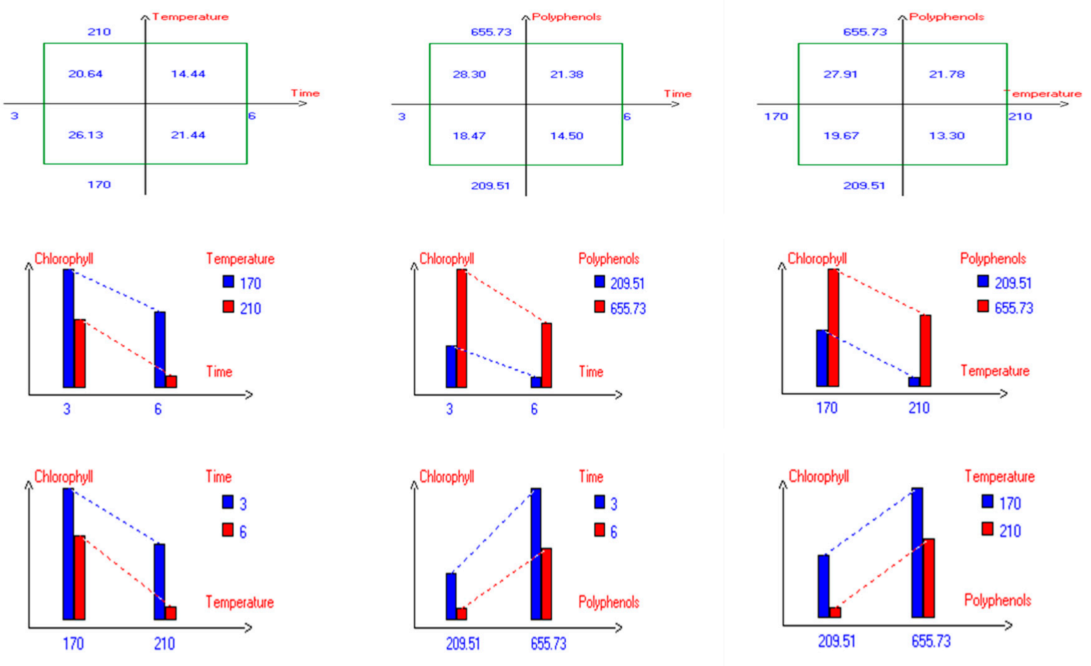

A **B** **C**

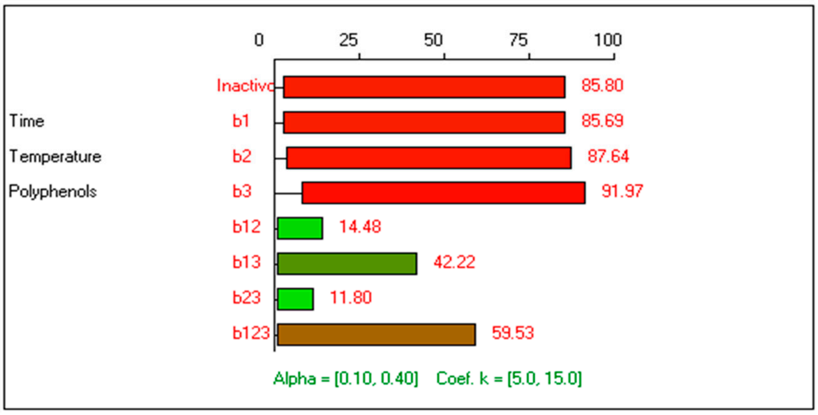

D

Figure S106. Combined interactions between the independent variables on a response variable (**chlorophyll** (mg/kg)) in EVOO **Hojiblanca** under D-F: (**A**) x_1 and x_2, (**B**) x_1 and x_3, (**C**) x_2 and x_3, and (**D**) results of variance analysis of regression equation model and the significance changes in each individual independent variable and interaction between the combined independent variables on chlorophyll; b represents a significant difference when be >b_{123}, while b represents no significant difference when be ≤b_{123}; b_1, b_2, b_3 are the main effects of the independent variables, while b_{12}, b_{13}, b_{23}, and b_{123} are the interaction effects of the independent variables. Moreover, x_1, x_2, and x_3 are coded variables (time, temperature, and polyphenols addition, respectively) for the experimental design in D-F process.

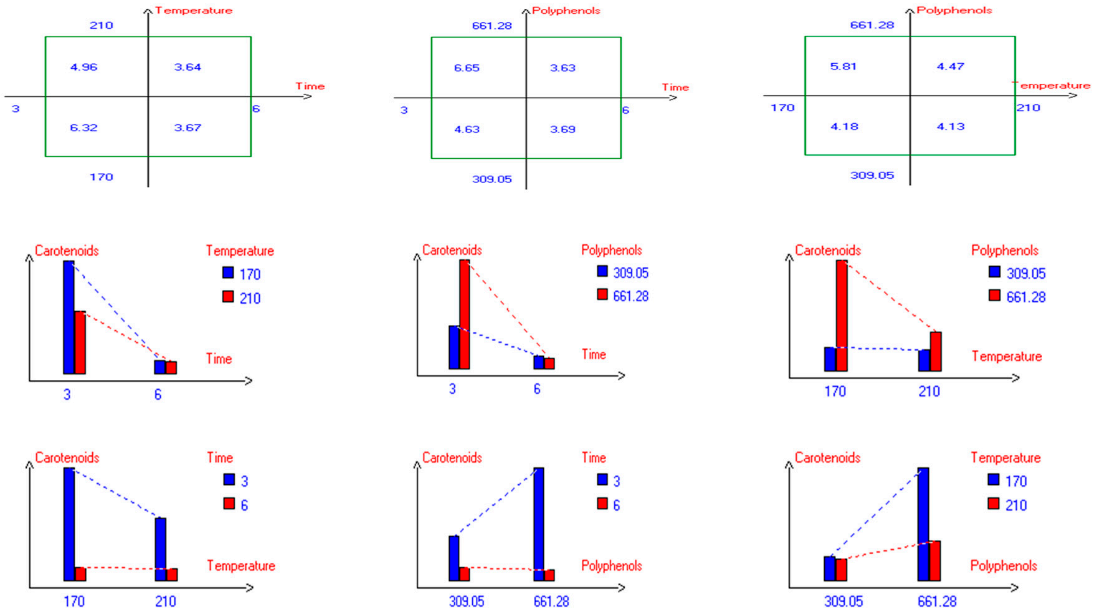

A **B** **C**

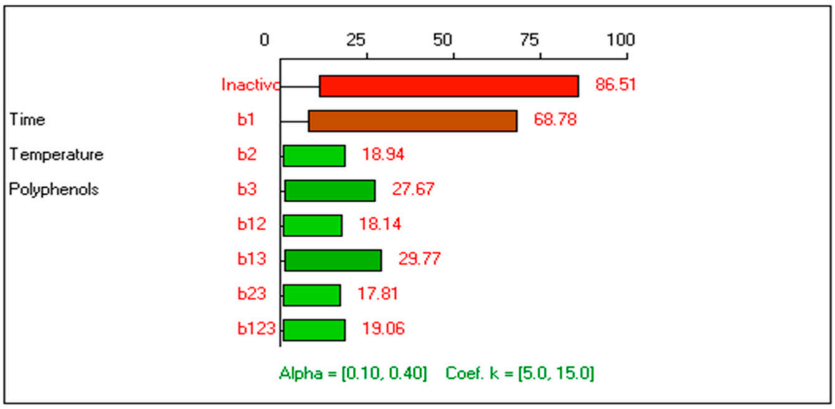

D

Figure S107. Combined interactions between the independent variables on a response variable (**carotenoids** (mg/kg)) in EVOO **Manzanilla** under D-F: (**A**) x_1 and x_2, (**B**) x_1 and x_3, (**C**) x_2 and x_3, and (**D**) results of variance analysis of regression equation model and the significance changes in each individual independent variable and interaction between the combined independent variables on carotenoids; b represents a significant difference when be >b_{123}, while b represents no significant difference when be ≤b_{123}; b_1, b_2, b_3 are the main effects of the independent variables, while b_{12}, b_{13}, b_{23}, and b_{123} are the interaction effects of the independent variables. Moreover, x_1, x_2, and x_3 are coded variables (time, temperature, and polyphenols addition, respectively) for the experimental design in D-F process.

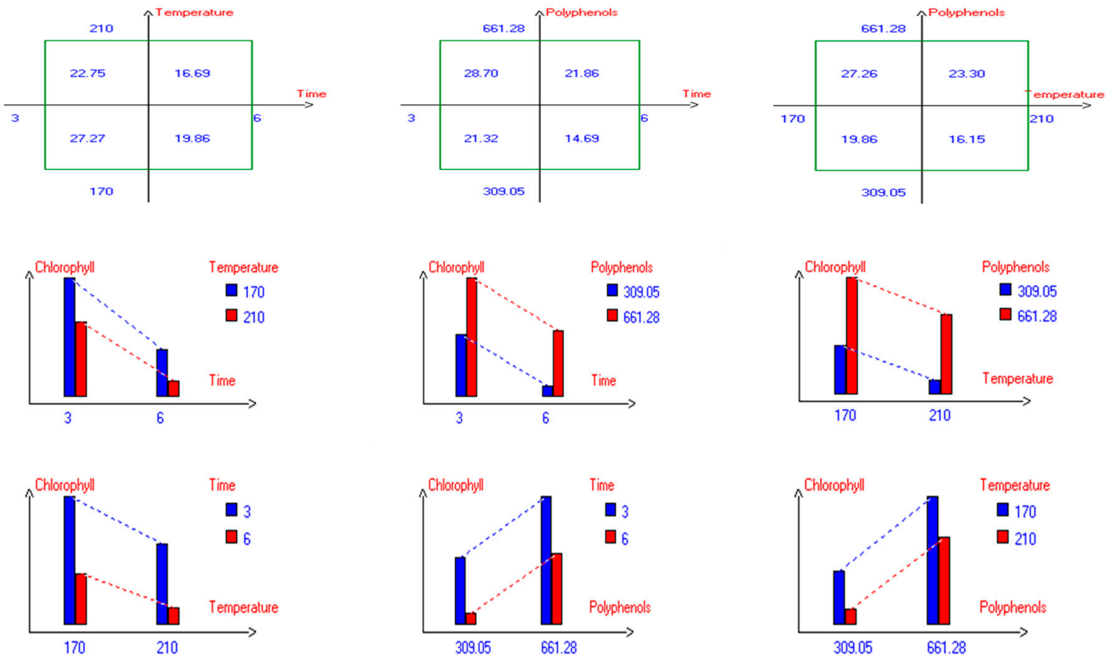

A B C

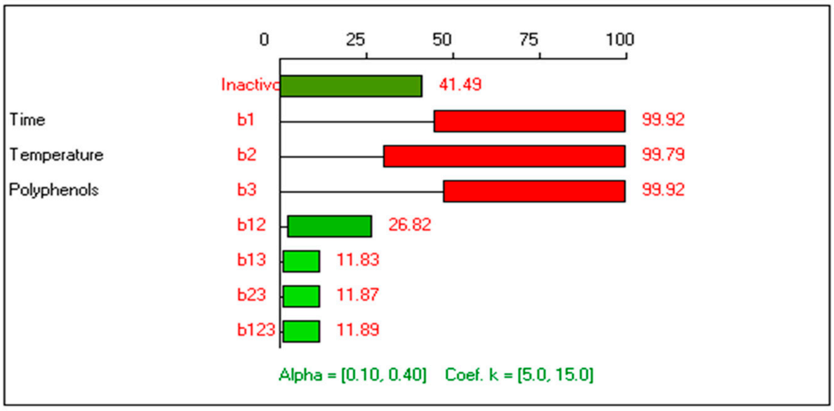

D

Figure S108. Combined interactions between the independent variables on a response variable (**chlorophyll** (mg/kg)) in EVOO **Manzanilla** under D-F: (**A**) x_1 and x_2, (**B**) x_1 and x_3, (**C**) x_2 and x_3, and (**D**) results of variance analysis of regression equation model and the significance changes in each individual independent variable and interaction between the combined independent variables on chlorophyll; b represents a significant difference when be $>b_{123}$, while b represents no significant difference when be $\leq b_{123}$; b_1, b_2, b_3 are the main effects of the independent variables, while b_{12}, b_{13}, b_{23}, and b_{123} are the interaction effects of the independent variables. Moreover, x_1, x_2, and x_3 are coded variables (time, temperature, and polyphenols addition, respectively) for the experimental design in D-F process.

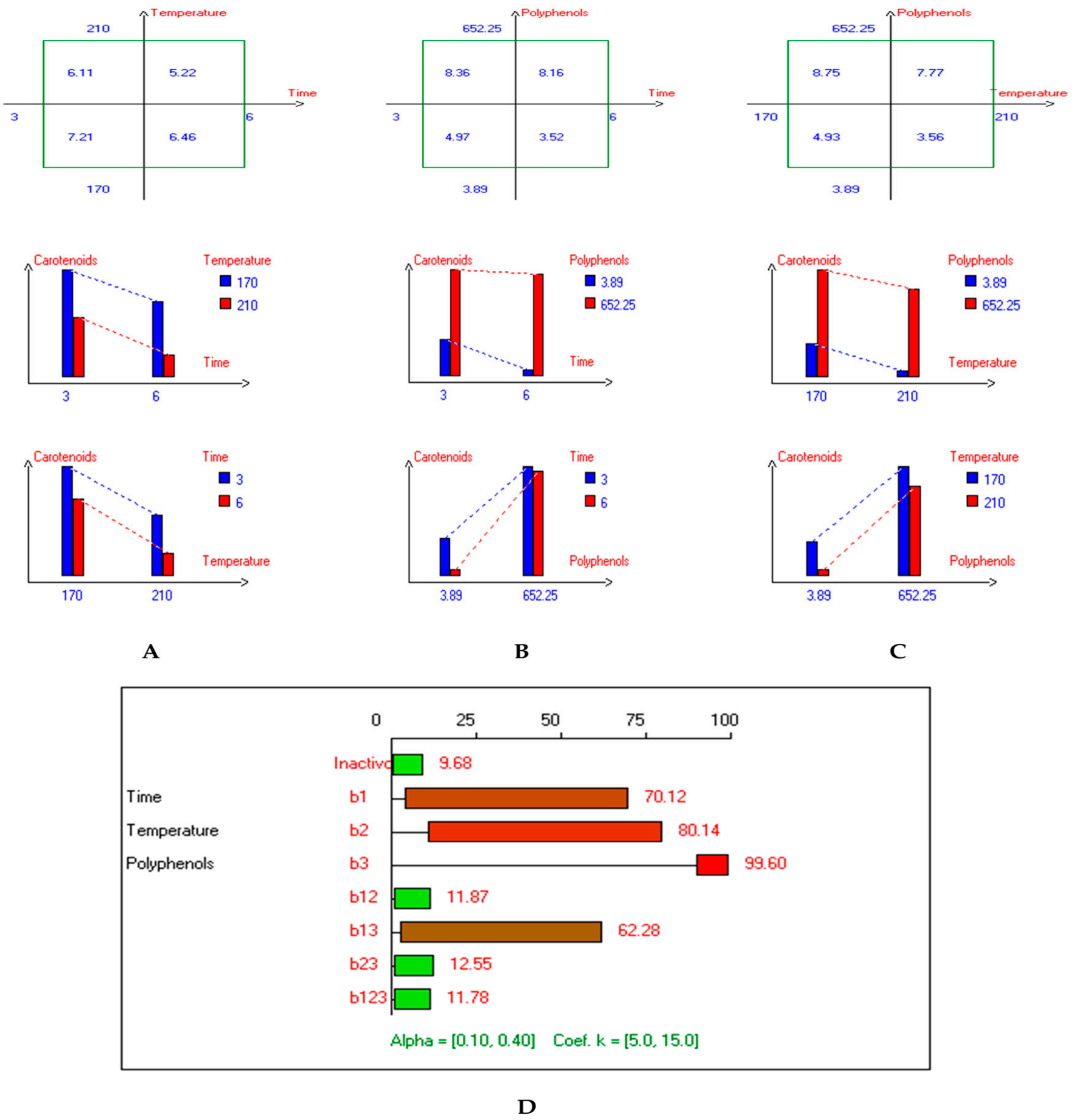

Figure S109. Combined interactions between the independent variables on a response variable (**carotenoids** (mg/kg)) in **Pomace** olive oil under D-F: (**A**) x_1 and x_2, (**B**) x_1 and x_3, (**C**) x_2 and x_3, and (**D**) results of variance analysis of regression equation model and the significance changes in each individual independent variable and interaction between the combined independent variables on carotenoids; b represents a significant difference when be $>b_{123}$, while b represents no significant difference when be $\leq b_{123}$; b_1, b_2, b_3 are the main effects of the independent variables, while b_{12}, b_{13}, b_{23}, and b_{123} are the interaction effects of the independent variables. Moreover, x_1, x_2, and x_3 are coded variables (time, temperature, and polyphenols addition, respectively) for the experimental design in D-F process.

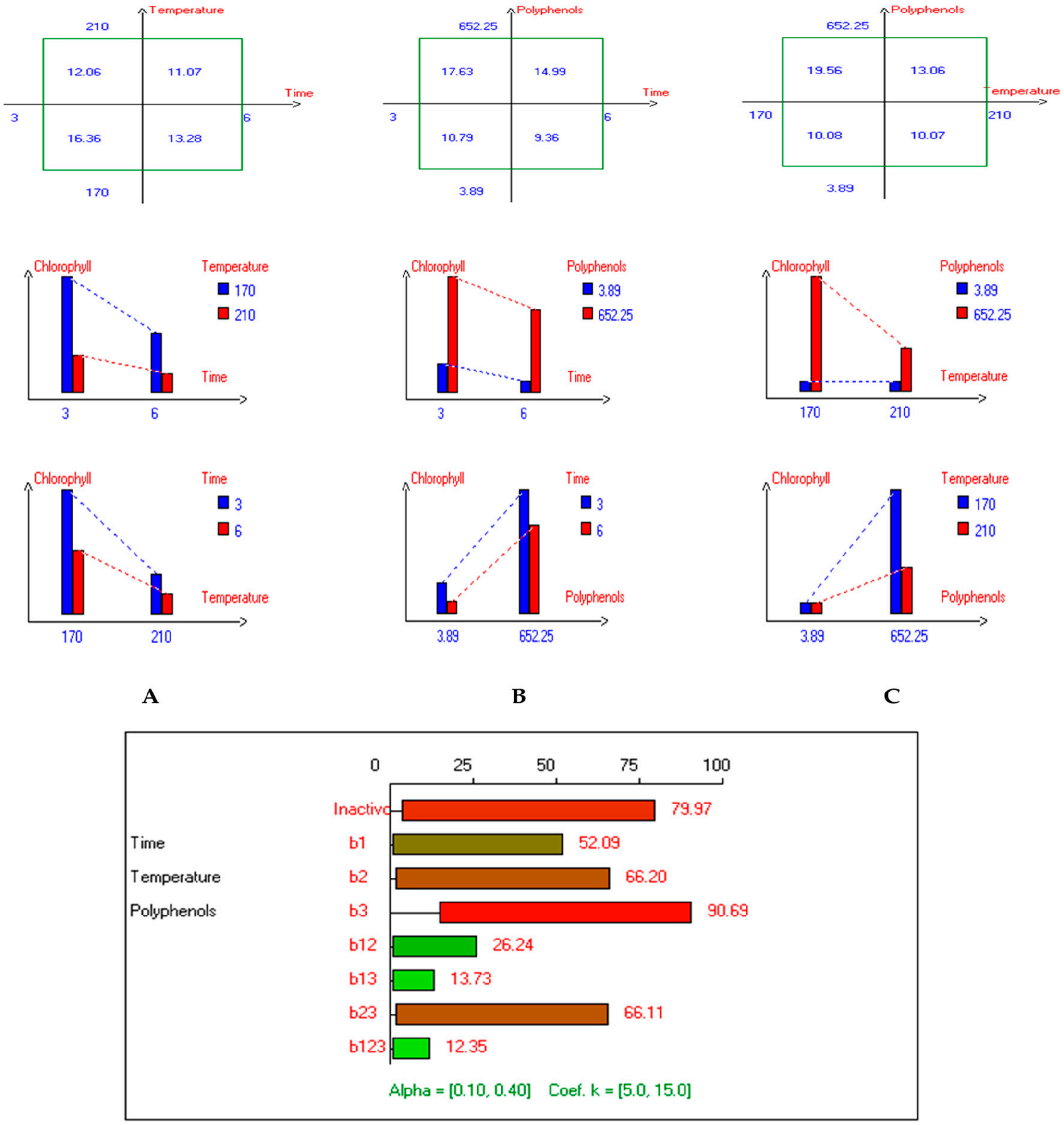

D

Figure S110. Combined interactions between the independent variables on a response variable (**chlorophyll** (mg/kg)) in **Pomace** olive oil under D-F: (**A**) x_1 and x_2, (**B**) x_1 and x_3, (**C**) x_2 and x_3, and (**D**) results of variance analysis of regression equation model and the significance changes in each individual independent variable and interaction between the combined independent variables on chlorophyll; b represents a significant difference when be $>b_{123}$, while b represents no significant difference when be $\leq b_{123}$; b_1, b_2, b_3 are the main effects of the independent variables, while b_{12}, b_{13}, b_{23}, and b_{123} are the interaction effects of the independent variables. Moreover, x_1, x_2, and x_3 are coded variables (time, temperature, and polyphenols addition, respectively) for the experimental design in D-F process.

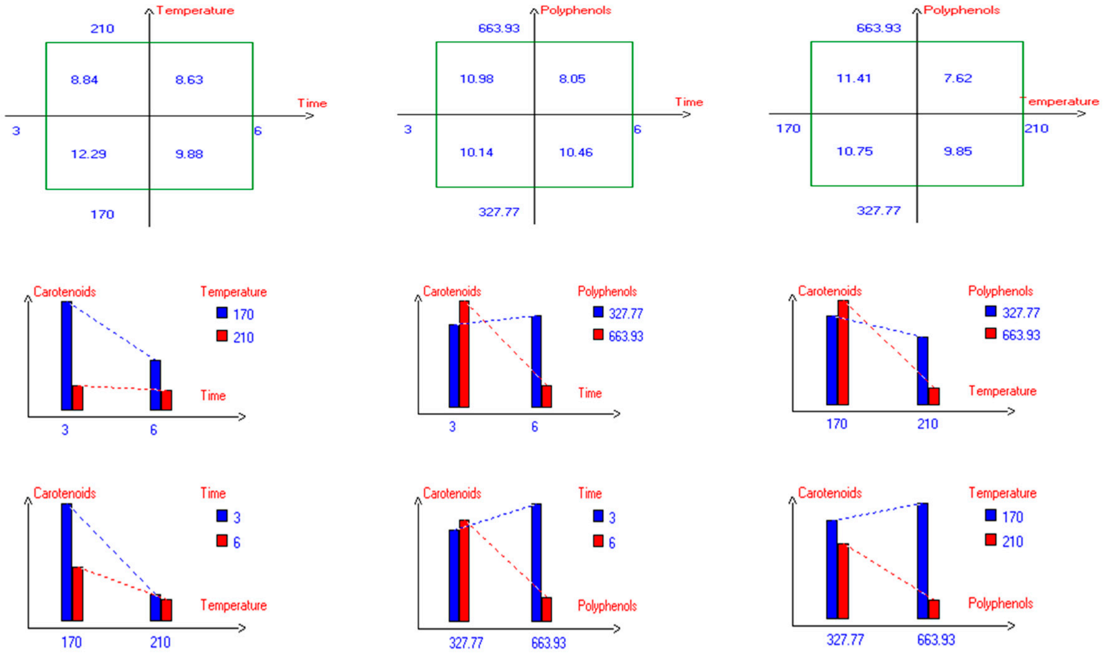

A **B** **C**

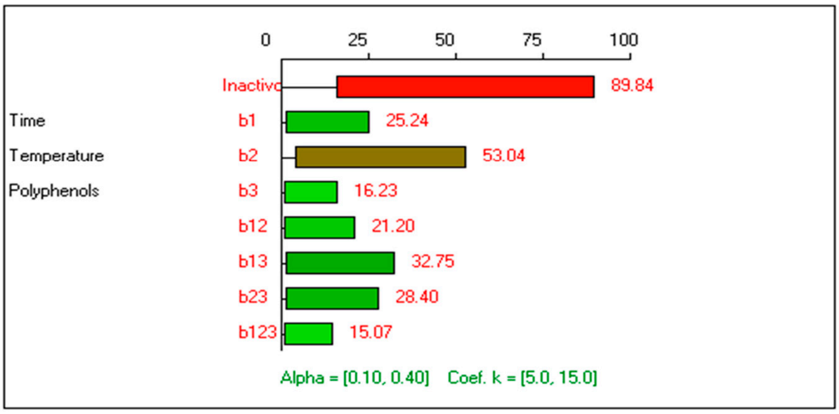

D

Figure S111. Combined interactions between the independent variables on a response variable (**carotenoids** (mg/kg)) in EVOO **Koroneiki** under D-F: (**A**) x_1 and x_2, (**B**) x_1 and x_3, (**C**) x_2 and x_3, and (**D**) results of variance analysis of regression equation model and the significance changes in each individual independent variable and interaction between the combined independent variables on carotenoids; b represents a significant difference when be $>b_{123}$, while b represents no significant difference when be $\leq b_{123}$; b_1, b_2, b_3 are the main effects of the independent variables, while b_{12}, b_{13}, b_{23}, and b_{123} are the interaction effects of the independent variables. Moreover, x_1, x_2, and x_3 are coded variables (time, temperature, and polyphenols addition, respectively) for the experimental design in D-F process.

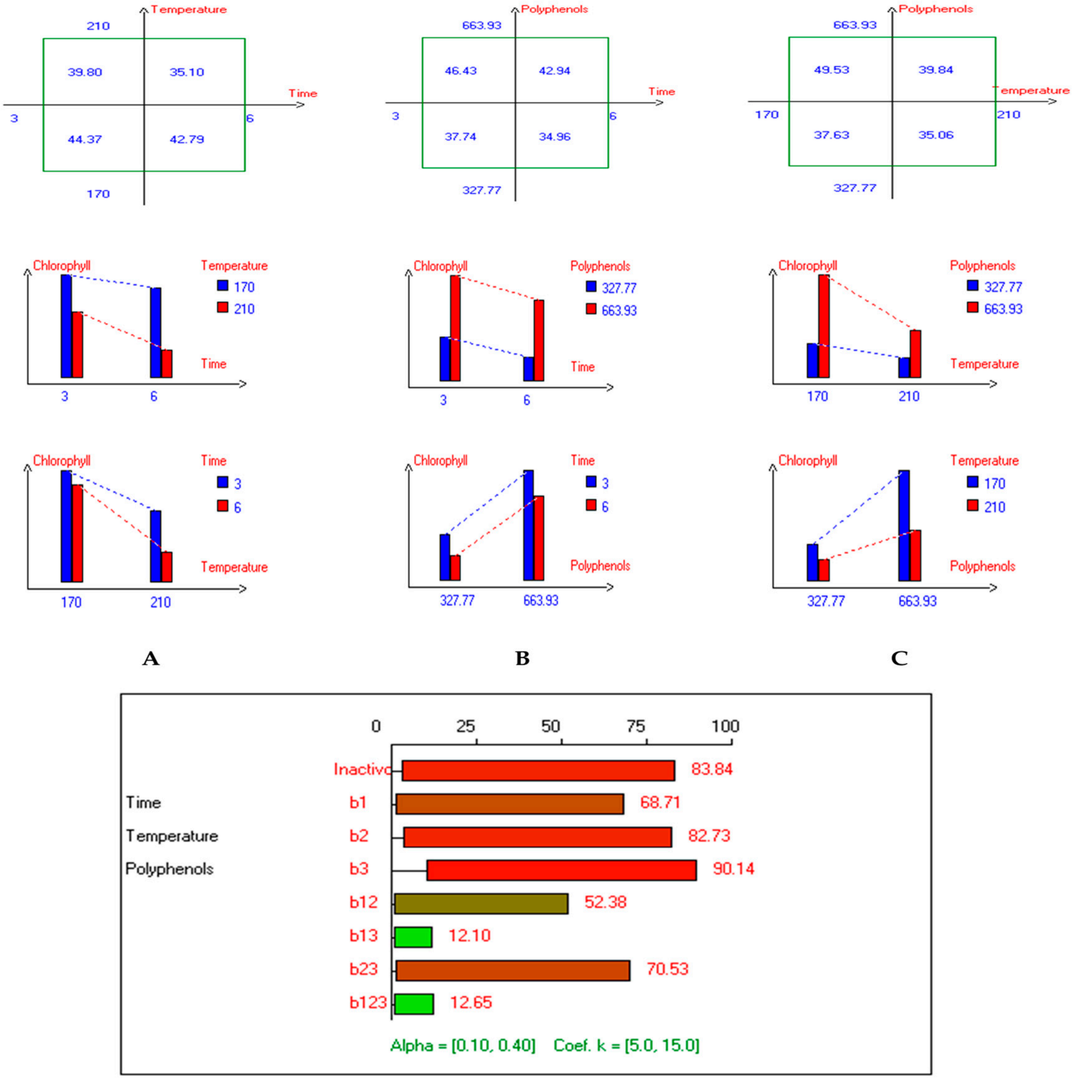

D

Figure S112. Combined interactions between the independent variables on a response variable (**chlorophyll** (mg/kg)) in EVOO **Koroneiki** under D-F: (**A**) x_1 and x_2, (**B**) x_1 and x_3, (**C**) x_2 and x_3, and (**D**) results of variance analysis of regression equation model and the significance changes in each individual independent variable and interaction between the combined independent variables on chlorophyll; b represents a significant difference when be $>b_{123}$, while b represents no significant difference when be $\leq b_{123}$; b_1, b_2, b_3 are the main effects of the independent variables, while b_{12}, b_{13}, b_{23}, and b_{123} are the interaction effects of the independent variables. Moreover, x_l, x_2, and x_3 are coded variables (time, temperature, and polyphenols addition, respectively) for the experimental design in D-F process.

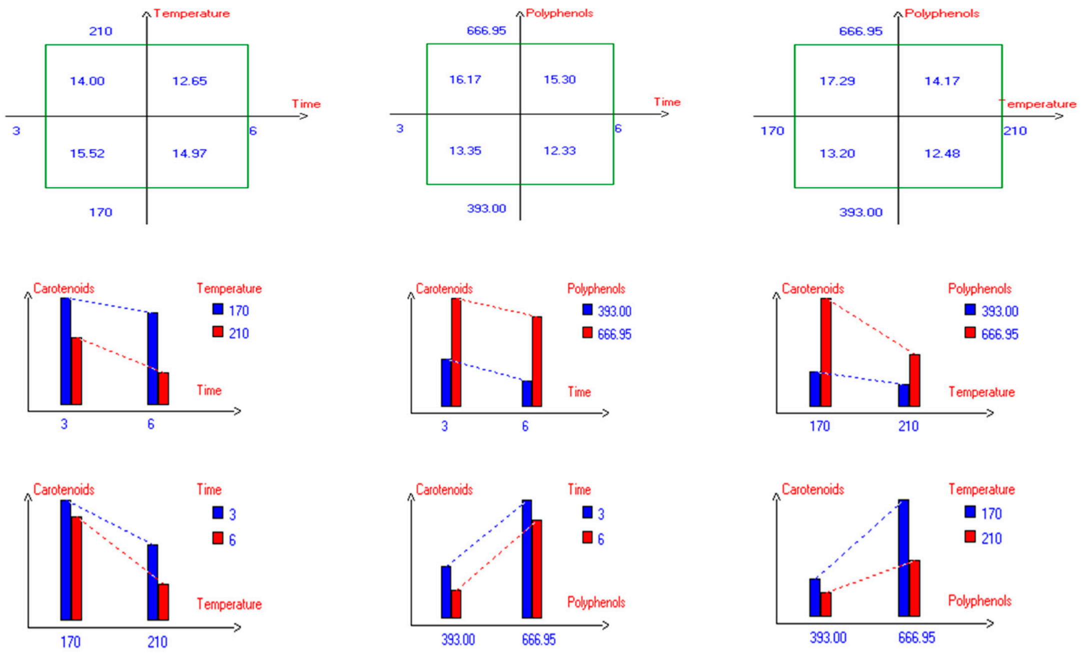

A **B** **C**

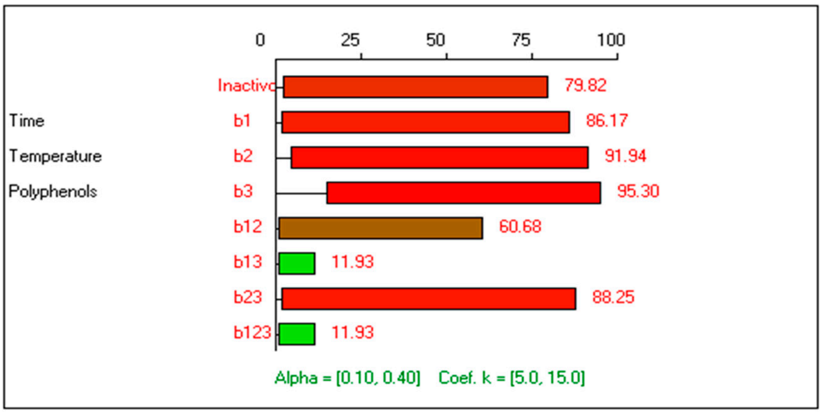

D

Figure S113. Combined interactions between the independent variables on a response variable (**carotenoids** (mg/kg)) in EVOO **Arbosana** under D-F: (**A**) x_1 and x_2, (**B**) x_1 and x_3, (**C**) x_2 and x_3, and (**D**) results of variance analysis of regression equation model and the significance changes in each individual independent variable and interaction between the combined independent variables on carotenoids; b represents a significant difference when be $>b_{123}$, while b represents no significant difference when be $\leq b_{123}$; b_1, b_2, b_3 are the main effects of the independent variables, while b_{12}, b_{13}, b_{23}, and b_{123} are the interaction effects of the independent variables. Moreover, x_1, x_2, and x_3 are coded variables (time, temperature, and polyphenols addition, respectively) for the experimental design in D-F process.

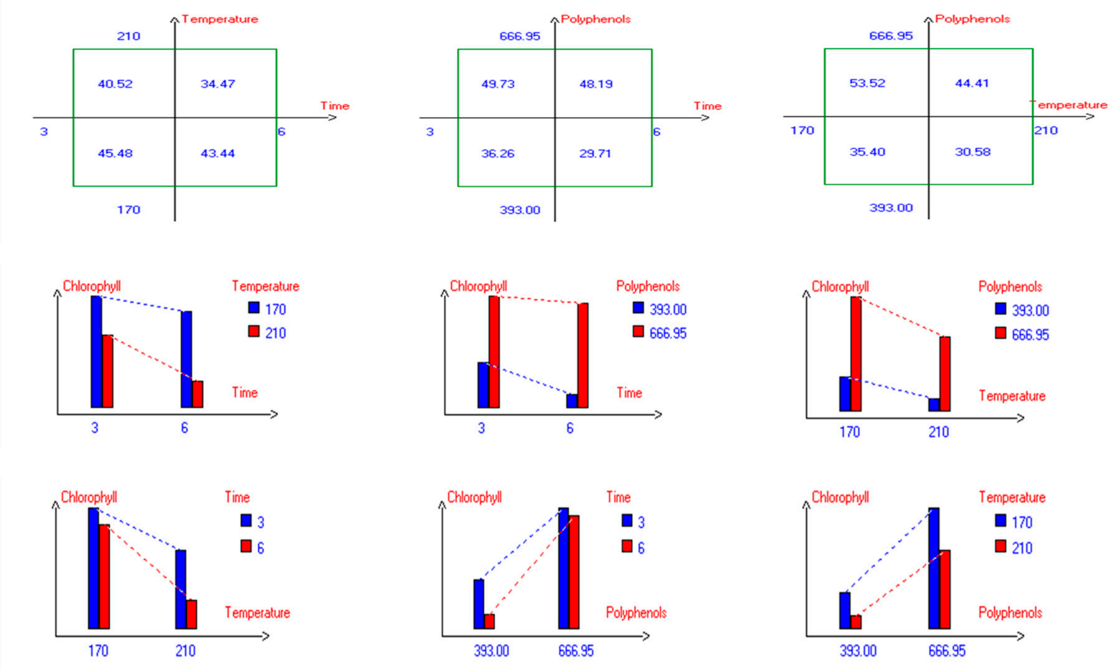

A **B** **C**

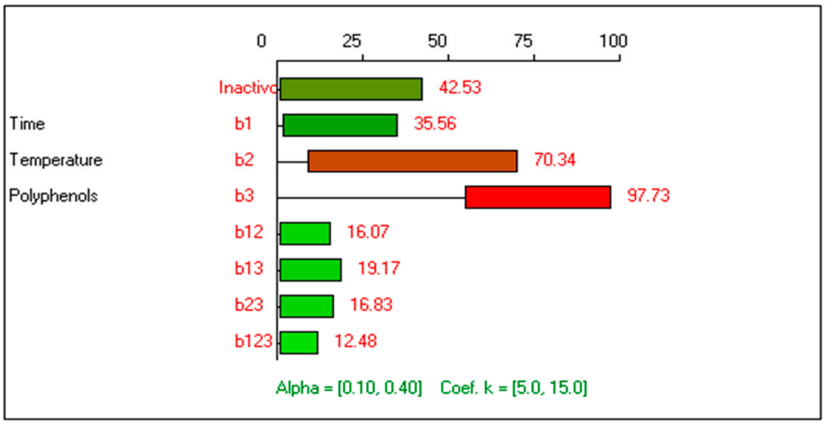

D

Figure S114. Combined interactions between the independent variables on a response variable (**chlorophyll** (mg/kg)) in EVOO **Arbosana** under D-F: (**A**) x_1 and x_2, (**B**) x_1 and x_3, (**C**) x_2 and x_3, and (**D**) results of variance analysis of regression equation model and the significance changes in each individual independent variable and interaction between the combined independent variables on chlorophyll; b represents a significant difference when be $>b_{123}$, while b represents no significant difference when be $\leq b_{123}$; b_1, b_2, b_3 are the main effects of the independent variables, while b_{12}, b_{13}, b_{23}, and b_{123} are the interaction effects of the independent variables. Moreover, x_1, x_2, and x_3 are coded variables (time, temperature, and polyphenols addition, respectively) for the experimental design in D-F process.

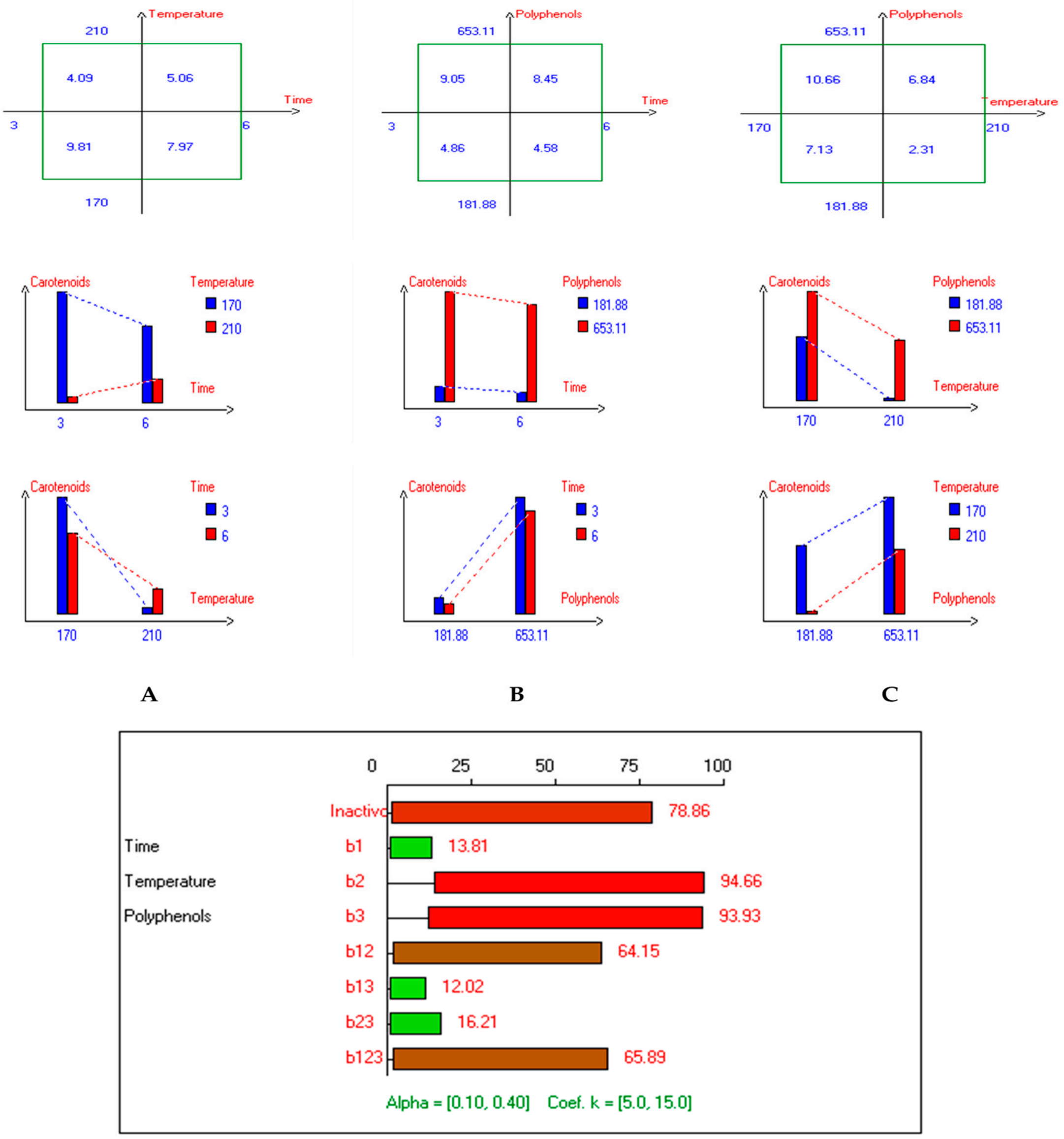

D

Figure S115. Combined interactions between the independent variables on a response variable (**carotenoids** (mg/kg)) in **Olive oil 1°** under D-F: (**A**) x_1 and x_2, (**B**) x_1 and x_3, (**C**) x_2 and x_3, and (**D**) results of variance analysis of regression equation model and the significance changes in each individual independent variable and interaction between the combined independent variables on carotenoids; b represents a significant difference when be $>b_{123}$, while b represents no significant difference when be $\leq b_{123}$; b_1, b_2, b_3 are the main effects of the independent variables, while b_{12}, b_{13}, b_{23}, and b_{123} are the interaction effects of the independent variables. Moreover, x_1, x_2, and x_3 are coded variables (time, temperature, and polyphenols addition, respectively) for the experimental design in D-F process.

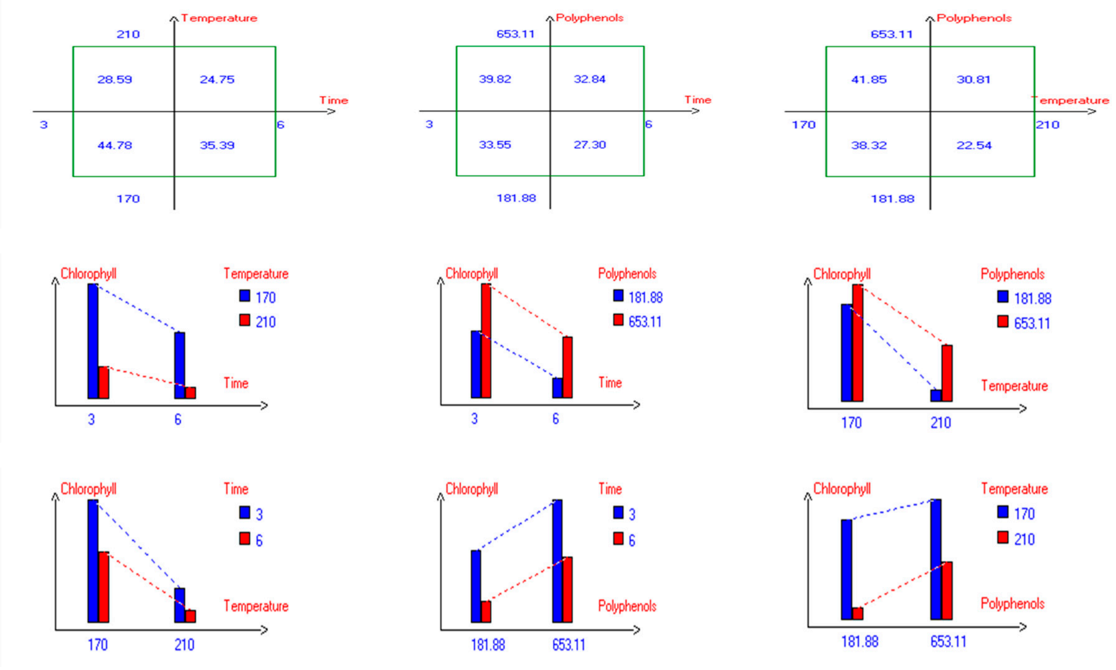

A **B** **C**

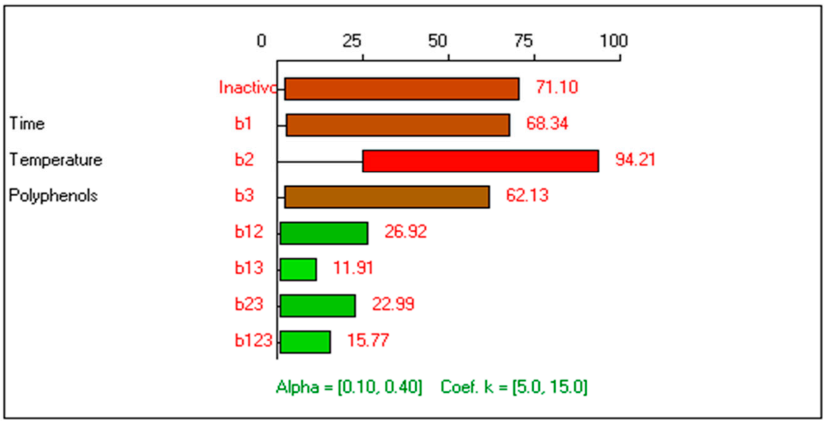

D

Figure S116. Combined interactions between the independent variables on a response variable (**chlorophyll** (mg/kg)) in **Olive oil 1°** under D-F: (**A**) x_1 and x_2, (**B**) x_1 and x_3, (**C**) x_2 and x_3, and (**D**) results of variance analysis of regression equation model and the significance changes in each individual independent variable and interaction between the combined independent variables on chlorophyll; b represents a significant difference when be $>b_{123}$, while b represents no significant difference when be $\leq b_{123}$; b_1, b_2, b_3 are the main effects of the independent variables, while b_{12}, b_{13}, b_{23}, and b_{123} are the interaction effects of the independent variables. Moreover, x_1, x_2, and x_3 are coded variables (time, temperature, and polyphenols addition, respectively) for the experimental design in D-F process.

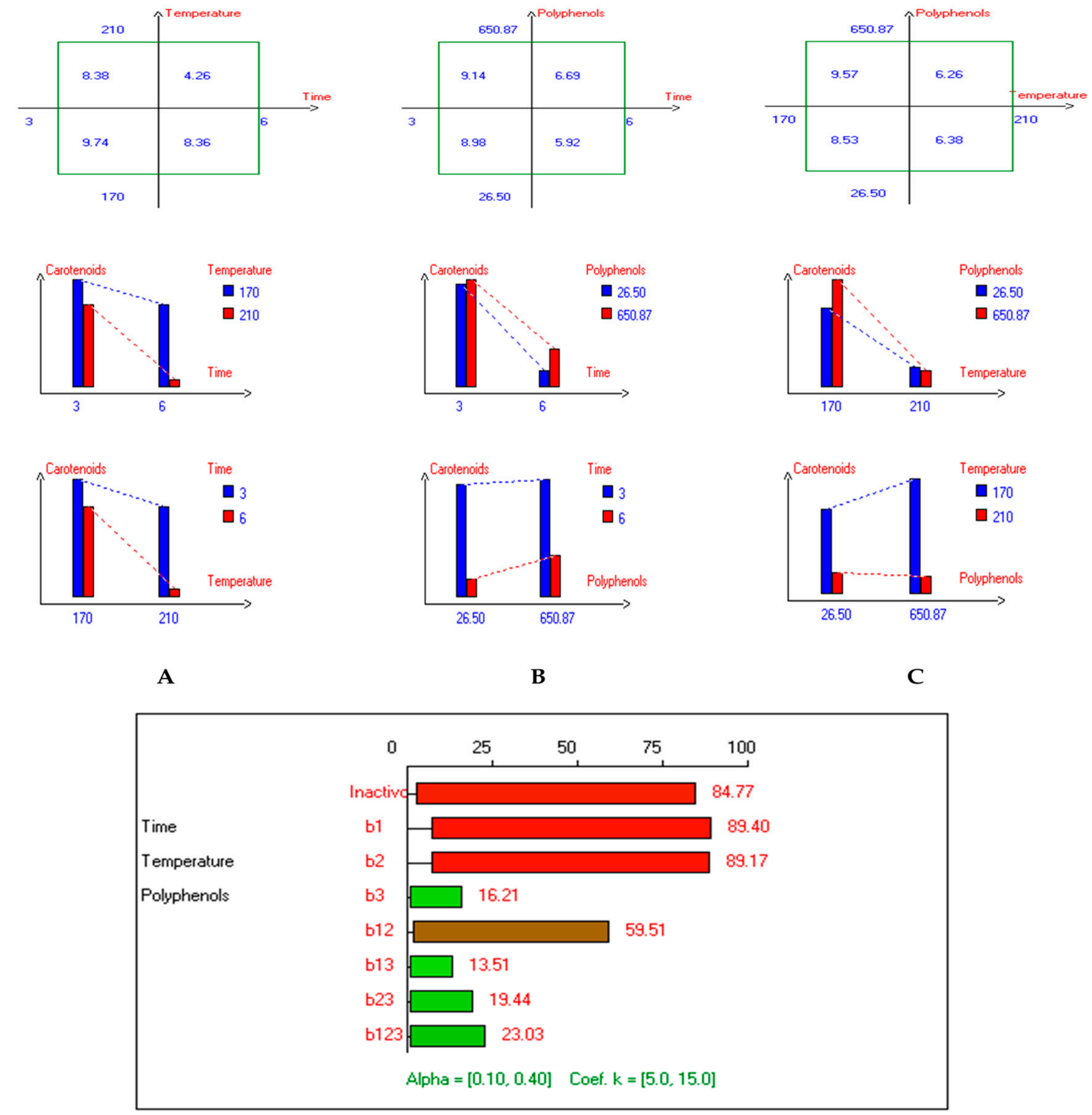

D

Figure S117. Combined interactions between the independent variables on a response variable (**carotenoids** (mg/kg)) in **Olive oil 0.4°** under D-F: (**A**) x_1 and x_2, (**B**) x_1 and x_3, (**C**) x_2 and x_3, and (**D**) results of variance analysis of regression equation model and the significance changes in each individual independent variable and interaction between the combined independent variables on carotenoids; b represents a significant difference when be $>b_{123}$, while b represents no significant difference when be $\leq b_{123}$; b_1, b_2, b_3 are the main effects of the independent variables, while b_{12}, b_{13}, b_{23}, and b_{123} are the interaction effects of the independent variables. Moreover, x_1, x_2, and x_3 are coded variables (time, temperature, and polyphenols addition, respectively) for the experimental design in D-F process.

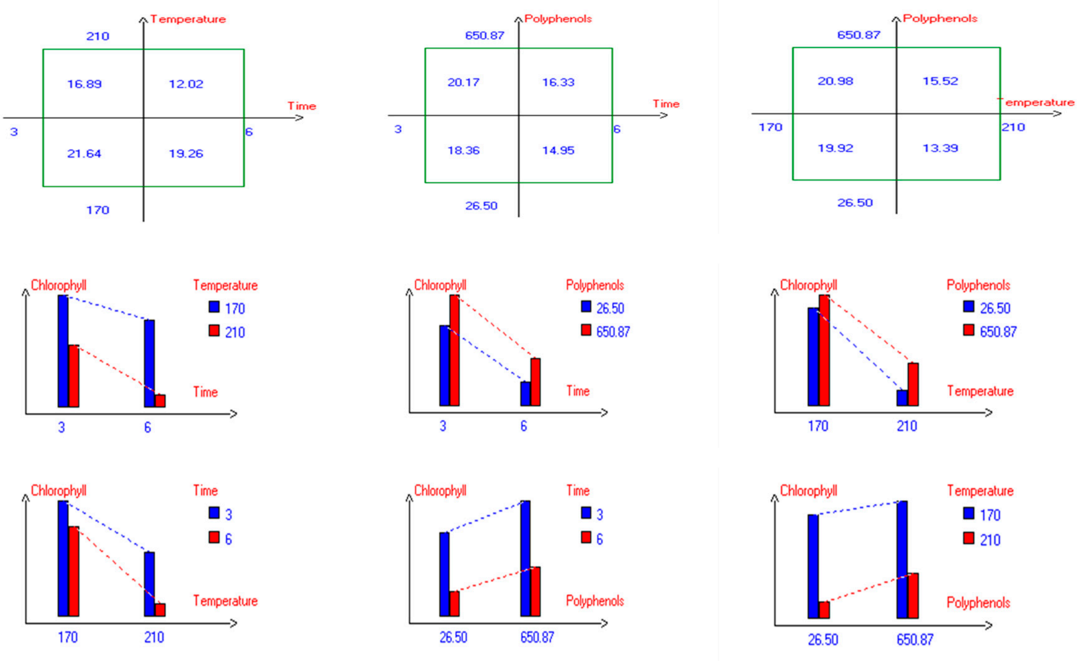

A **B** **C**

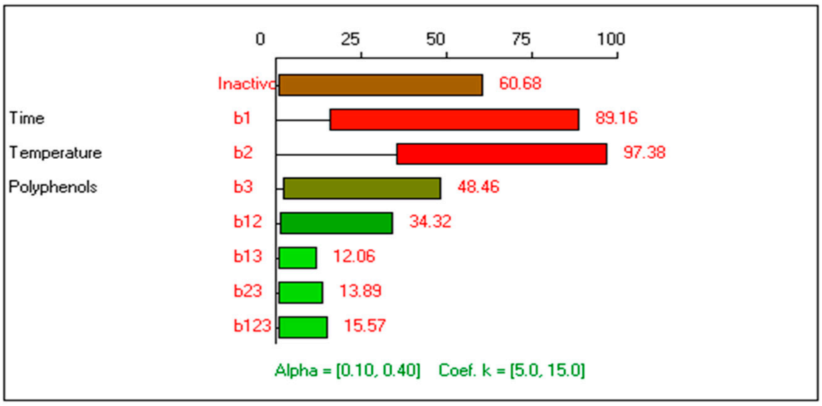

D

Figure S118. Combined interactions between the independent variables on a response variable (**chlorophyll** (mg/kg)) in **Olive oil 0.4°** under D-F: (**A**) x_1 and x_2, (**B**) x_1 and x_3, (**C**) x_2 and x_3, and (**D**) results of variance analysis of regression equation model and the significance changes in each individual independent variable and interaction between the combined independent variables on chlorophyll; b represents a significant difference when be $>b_{123}$, while b represents no significant difference when be $\leq b_{123}$; b_1, b_2, b_3 are the main effects of the independent variables, while b_{12}, b_{13}, b_{23}, and b_{123} are the interaction effects of the independent variables. Moreover, x_1, x_2, and x_3 are coded variables (time, temperature, and polyphenols addition, respectively) for the experimental design in D-F process.

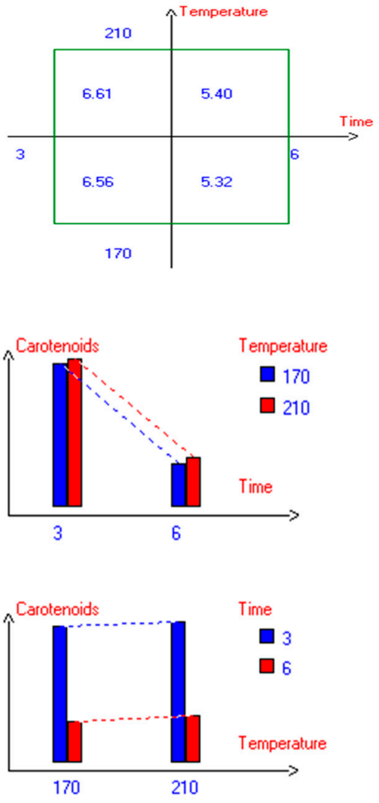

A

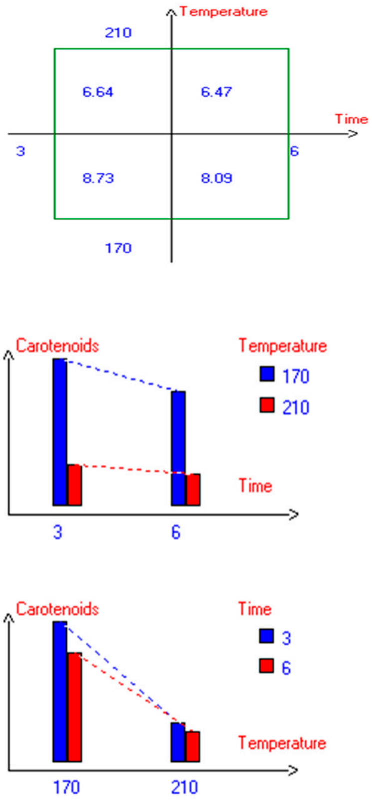

C

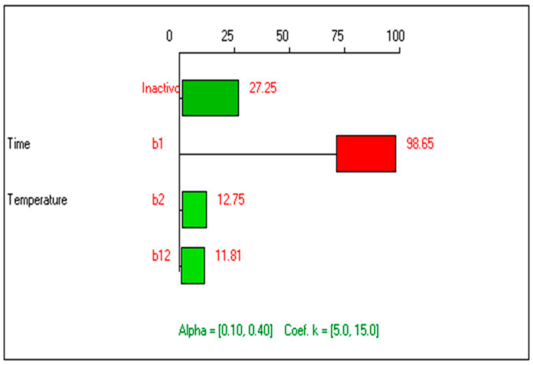

B

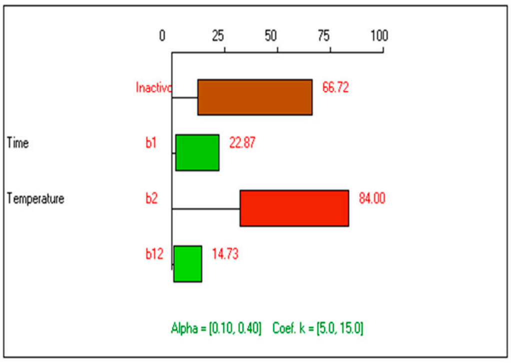

D

Figure S119. (A) Combined interactions between the independent variables (x_1 and x_2) on (**carotenoids mg/kg**) in **sunflower oil**, **(B)** Results of variance analysis of regression equation model and the significance changes of each individual independent variable and interaction between the combined independent variables on acidity in sunflower oil. **(C)** Combined interactions between the independent variables (x_1 and x_2) on (**carotenoids (mg/kg)**) in **sunflower oil-high oleic acid**, **(D)** Results of variance analysis of regression equation model and the significance changes of each individual independent variable and interaction between the combined independent variables on rancid score in sunflower oil-high oleic acid. Where, b represents significant difference when be $>b_{12}$; while b represents no significant difference when be $\leq b_{12}$. Moreover, b_1 and b_2 are the main effects of the independent variables, while b_{12} is the interaction effect of the independent variables. Where x_1: time, x_2: temperature.

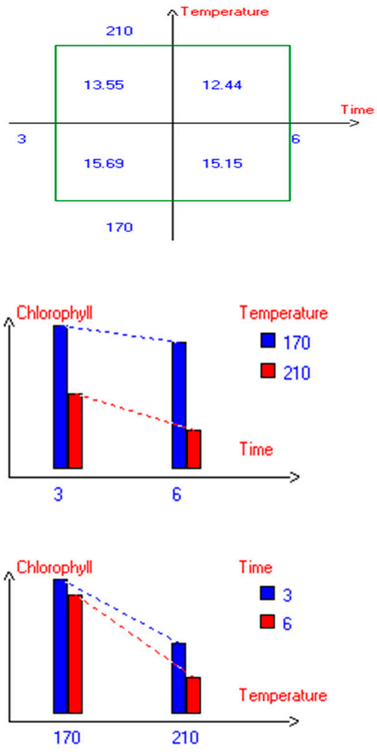

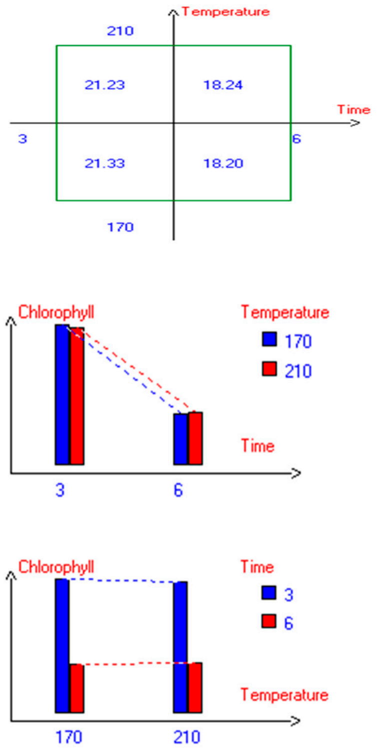

A **C**

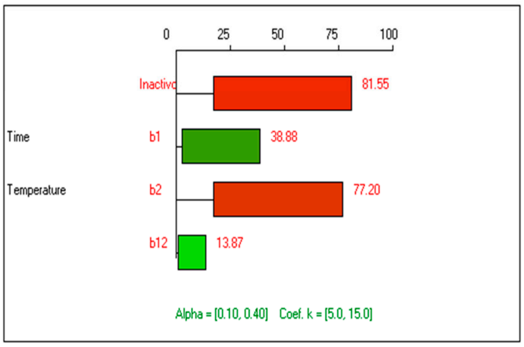

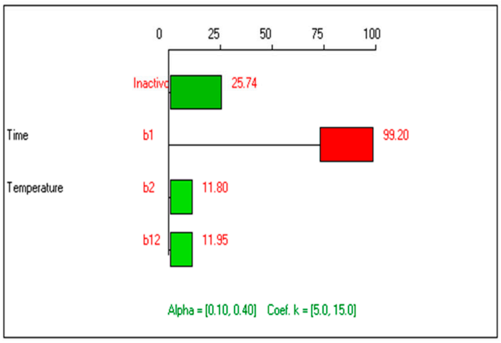

B **D**

Figure S120. (A) Combined interactions between the independent variables (x_1 and x_2) on (**Chlorophyll (mg/kg)**) in **sunflower oil**, **(B)** Results of variance analysis of regression equation model and the significance changes of each individual independent variable and interaction between the combined independent variables on acidity in sunflower oil. **(C)** Combined interactions between the independent variables (x_1 and x_2) on (**Chlorophyll** (**mg/kg**)) in **sunflower oil-high oleic acid**, **(D)** Results of variance analysis of regression equation model and the significance changes of each individual independent variable and interaction between the combined independent variables on rancid score in sunflower oil-high oleic acid. Where, b represents significant difference when be $>b_{12}$; while b represents no significant difference when be $\leq b_{12}$. Moreover, b_1 and b_2 are the main effects of the independent variables, while b_{12} is the interaction effect of the independent variables. Where x_1: time, x_2: temperature.

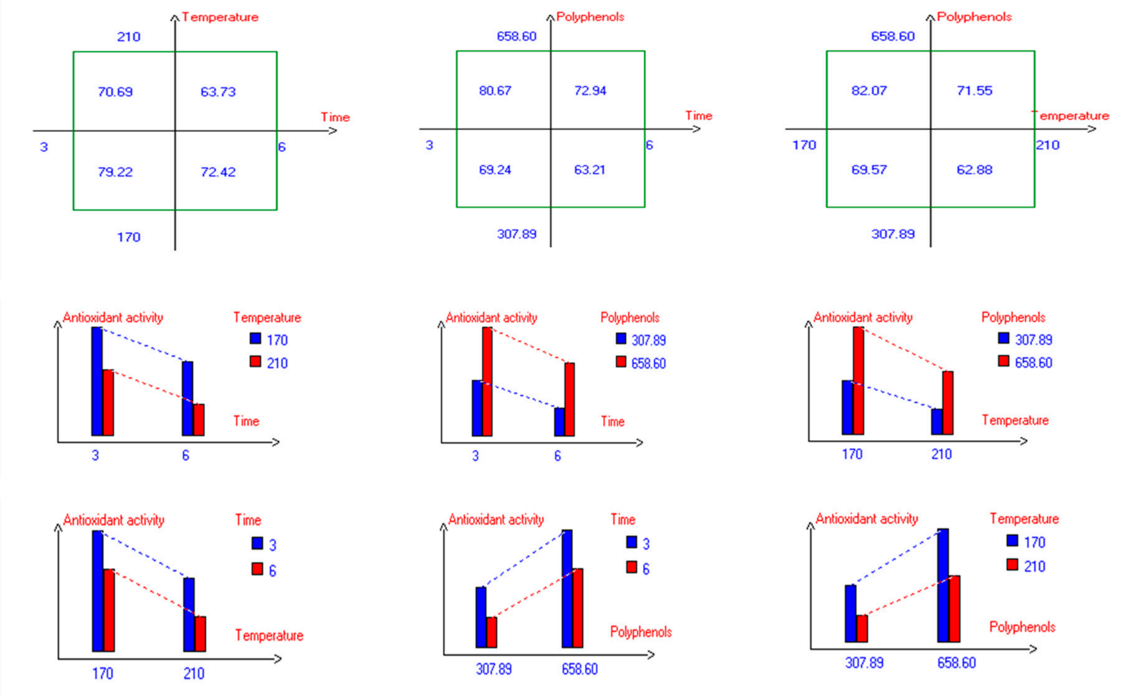

A **B** **C**

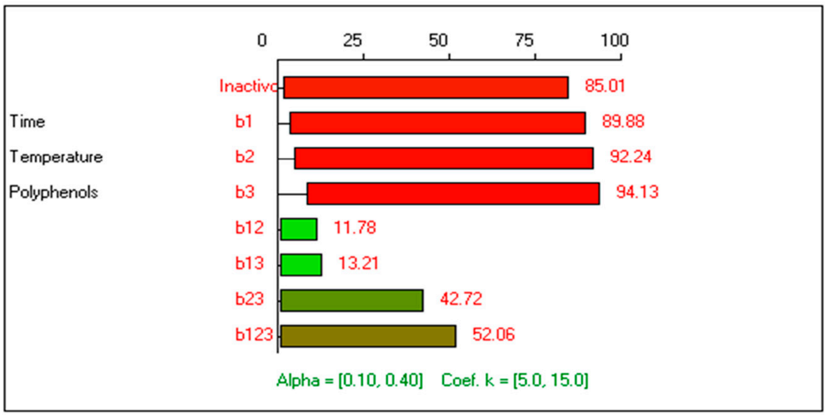

D

Figure S121. Combined interactions between the independent variables on a response variable (**antioxidant activity %**) in EVOO **Picual** under D-F: (**A**) x_1 and x_2, (**B**) x_1 and x_3, (**C**) x_2 and x_3, and (**D**) results of variance analysis of regression equation model and the significance changes in each individual independent variable and interaction between the combined independent variables on antioxidant activity; b represents a significant difference when be >b_{123}, while b represents no significant difference when be ≤b_{123}; b_1, b_2, b_3 are the main effects of the independent variables, while b_{12}, b_{13}, b_{23}, and b_{123} are the interaction effects of the independent variables. Moreover, x_l, x_2, and x_3 are coded variables (time, temperature, and polyphenols addition, respectively) for the experimental design in D-F process.

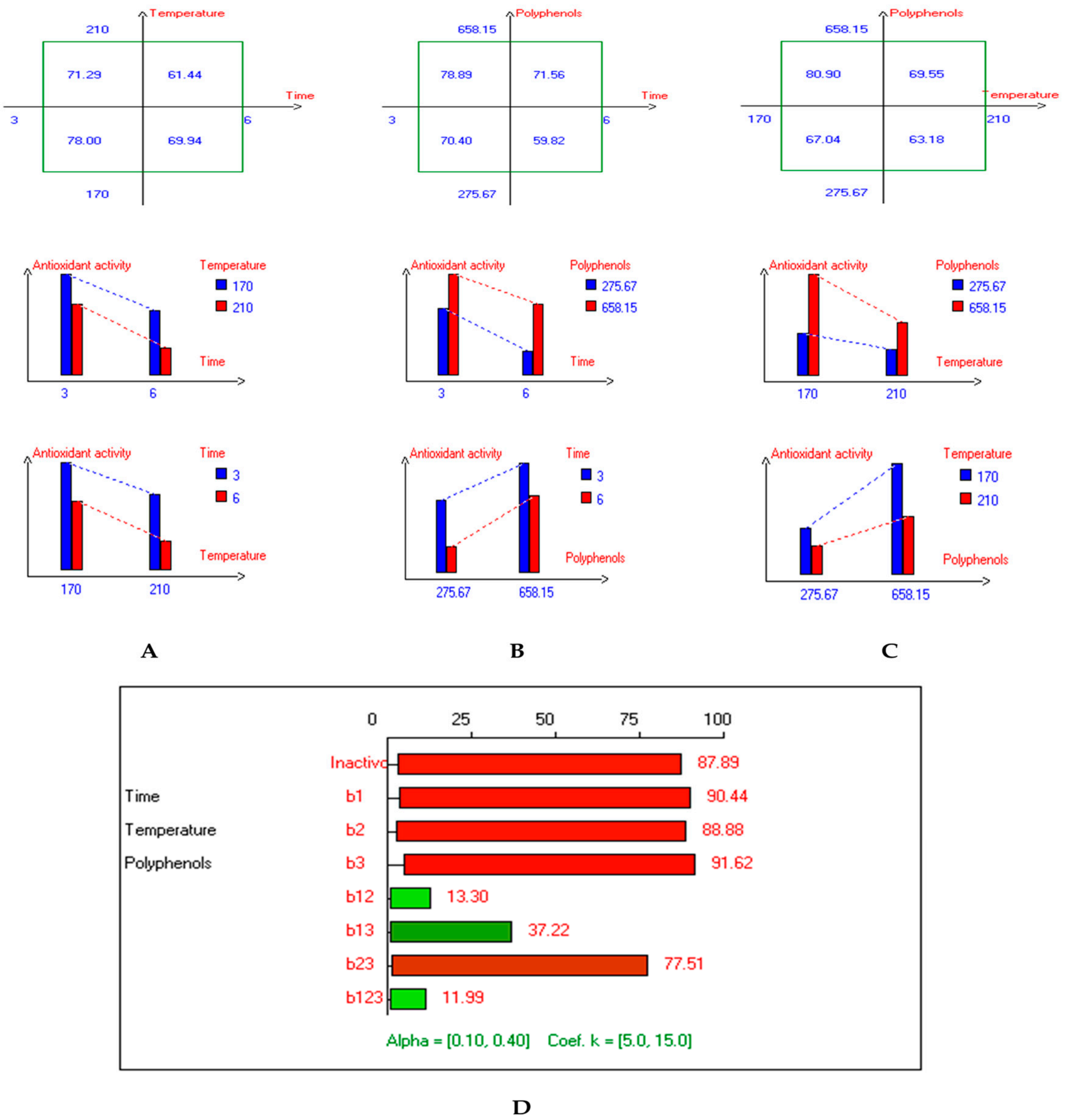

Figure S122. Combined interactions between the independent variables on a response variable (**antioxidant activity %**) in EVOO **Cornicabra** under D-F: (**A**) x_1 and x_2, (**B**) x_1 and x_3, (**C**) x_2 and x_3, and (**D**) results of variance analysis of regression equation model and the significance changes in each individual independent variable and interaction between the combined independent variables on antioxidant activity; b represents a significant difference when be >b_{123}, while b represents no significant difference when be ≤b_{123}; b_1, b_2, b_3 are the main effects of the independent variables, while b_{12}, b_{13}, b_{23}, and b_{123} are the interaction effects of the independent variables. Moreover, x_1, x_2, and x_3 are coded variables (time, temperature, and polyphenols addition, respectively) for the experimental design in D-F process.

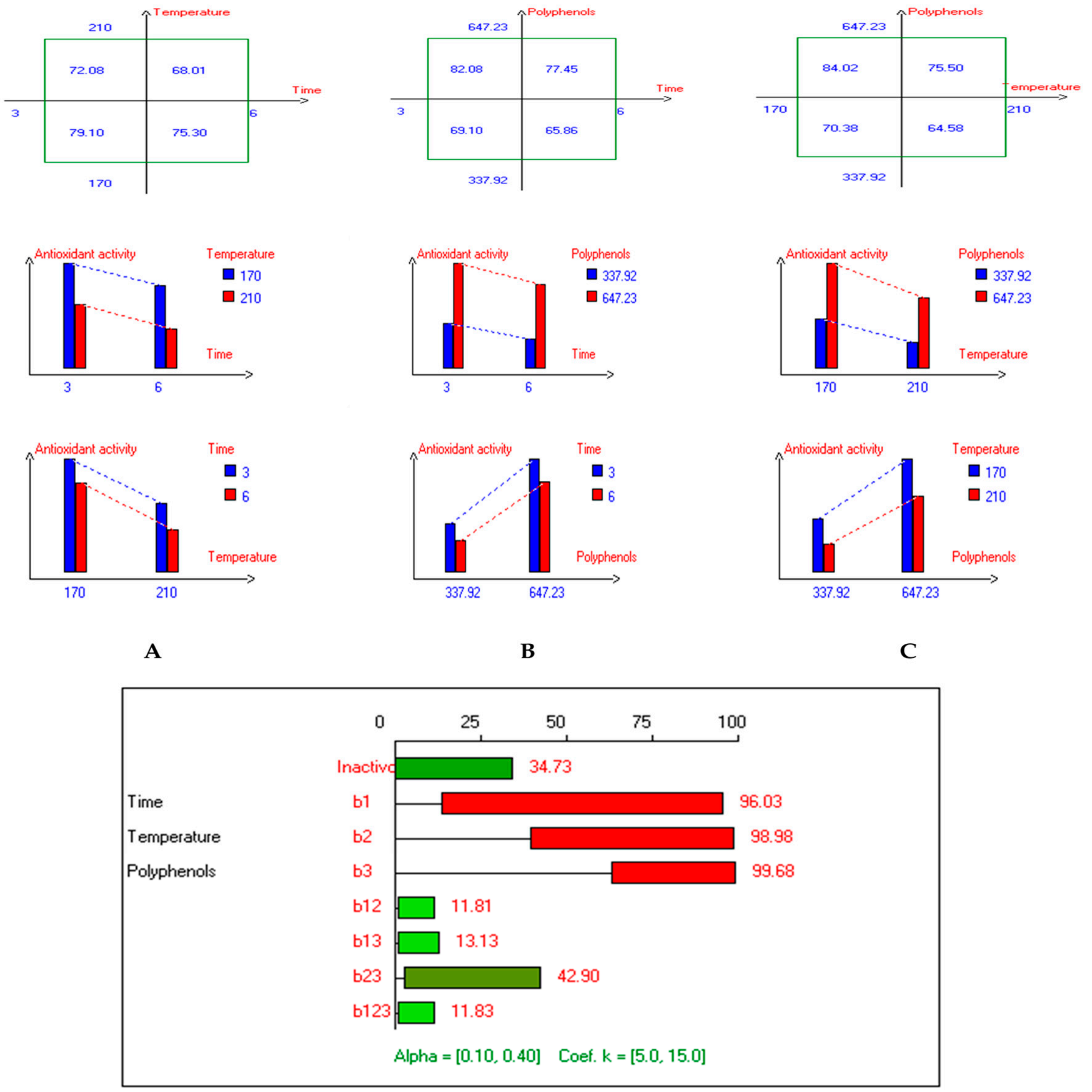

D

Figure S123. Combined interactions between the independent variables on a response variable (**antioxidant activity %**) in EVOO **Empeltre** under D-F: (**A**) x_1 and x_2, (**B**) x_1 and x_3, (**C**) x_2 and x_3, and (**D**) results of variance analysis of regression equation model and the significance changes in each individual independent variable and interaction between the combined independent variables on antioxidant activity; b represents a significant difference when be >b_{123}, while b represents no significant difference when be ≤b_{123}; b_1, b_2, b_3 are the main effects of the independent variables, while b_{12}, b_{13}, b_{23}, and b_{123} are the interaction effects of the independent variables. Moreover, x_I, x_2, and x_3 are coded variables (time, temperature, and polyphenols addition, respectively) for the experimental design in D-F process.

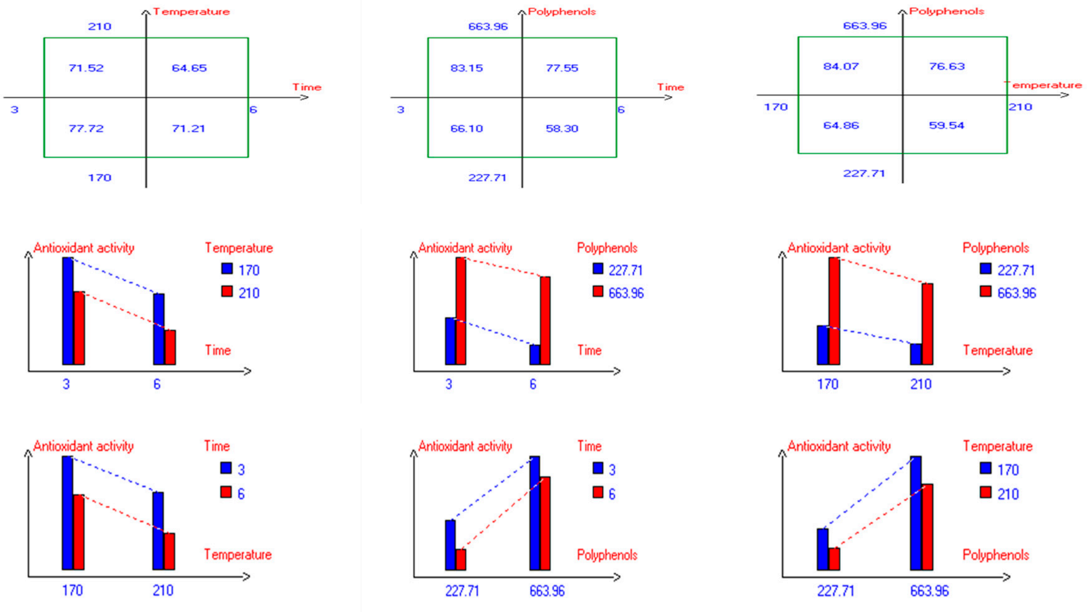

A **B** **C**

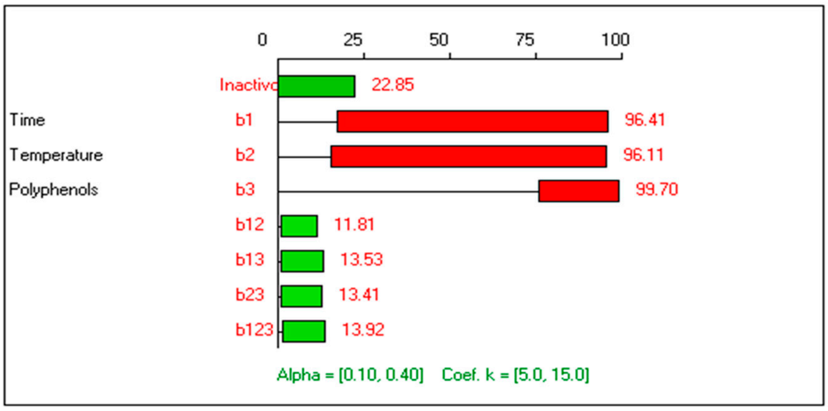

D

Figure S124. Combined interactions between the independent variables on a response variable (**antioxidant activity** %) in EVOO **Arbequina** under D-F: (**A**) x_1 and x_2, (**B**) x_1 and x_3, (**C**) x_2 and x_3, and (**D**) results of variance analysis of regression equation model and the significance changes in each individual independent variable and interaction between the combined independent variables on antioxidant activity; b represents a significant difference when be >b_{123}, while b represents no significant difference when be ≤b_{123}; b_1, b_2, b_3 are the main effects of the independent variables, while b_{12}, b_{13}, b_{23}, and b_{123} are the interaction effects of the independent variables. Moreover, x_1, x_2, and x_3 are coded variables (time, temperature, and polyphenols addition, respectively) for the experimental design in D-F process.

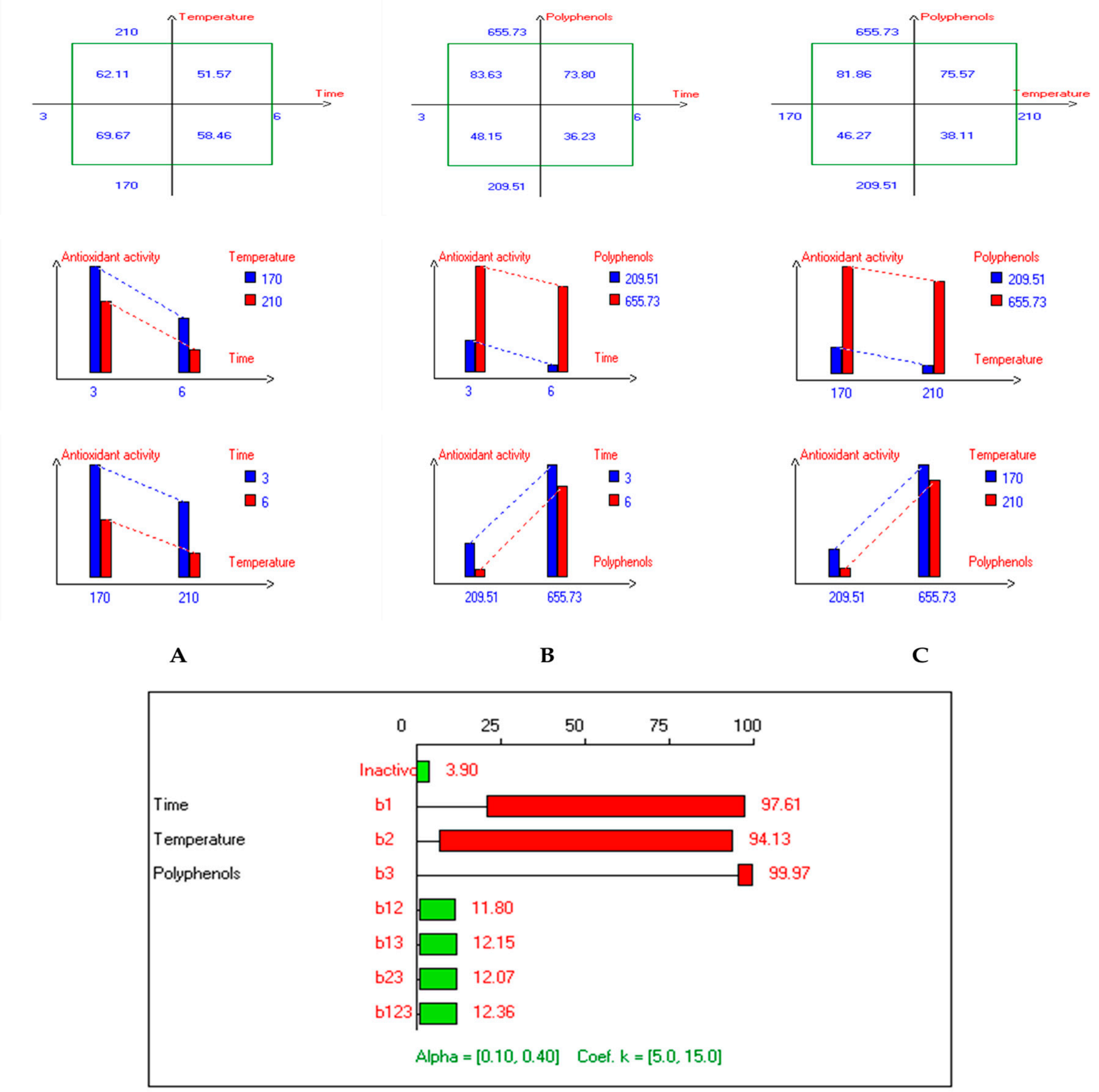

D

Figure S125. Combined interactions between the independent variables on a response variable (**antioxidant activity %**) in EVOO **Hojiblanca** under D-F: (**A**) x_1 and x_2, (**B**) x_1 and x_3, (**C**) x_2 and x_3, and (**D**) results of variance analysis of regression equation model and the significance changes in each individual independent variable and interaction between the combined independent variables on antioxidant activity; b represents a significant difference when be $>b_{123}$, while b represents no significant difference when be $\leq b_{123}$; b_1, b_2, b_3 are the main effects of the independent variables, while b_{12}, b_{13}, b_{23}, and b_{123} are the interaction effects of the independent variables. Moreover, x_1, x_2, and x_3 are coded variables (time, temperature, and polyphenols addition, respectively) for the experimental design in D-F process.

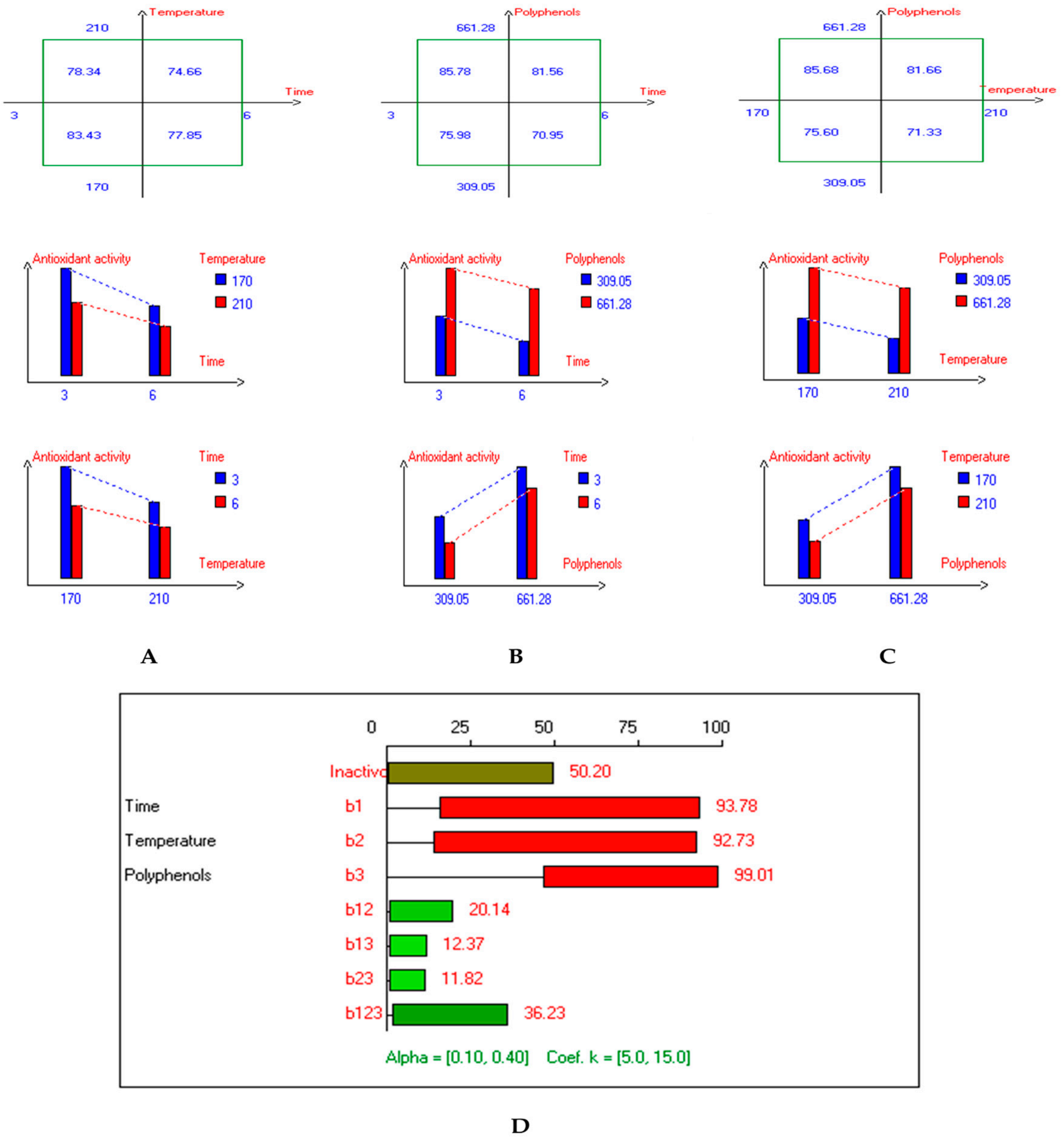

Figure S126. Combined interactions between the independent variables on a response variable (**antioxidant activity** %) in EVOO **Manzanilla** under D-F: (**A**) x_1 and x_2, (**B**) x_1 and x_3, (**C**) x_2 and x_3, and (**D**) results of variance analysis of regression equation model and the significance changes in each individual independent variable and interaction between the combined independent variables on antioxidant activity; b represents a significant difference when be $>b_{123}$, while b represents no significant difference when be $\leq b_{123}$; b_1, b_2, b_3 are the main effects of the independent variables, while b_{12}, b_{13}, b_{23}, and b_{123} are the interaction effects of the independent variables. Moreover, x_1, x_2, and x_3 are coded variables (time, temperature, and polyphenols addition, respectively) for the experimental design in D-F process.

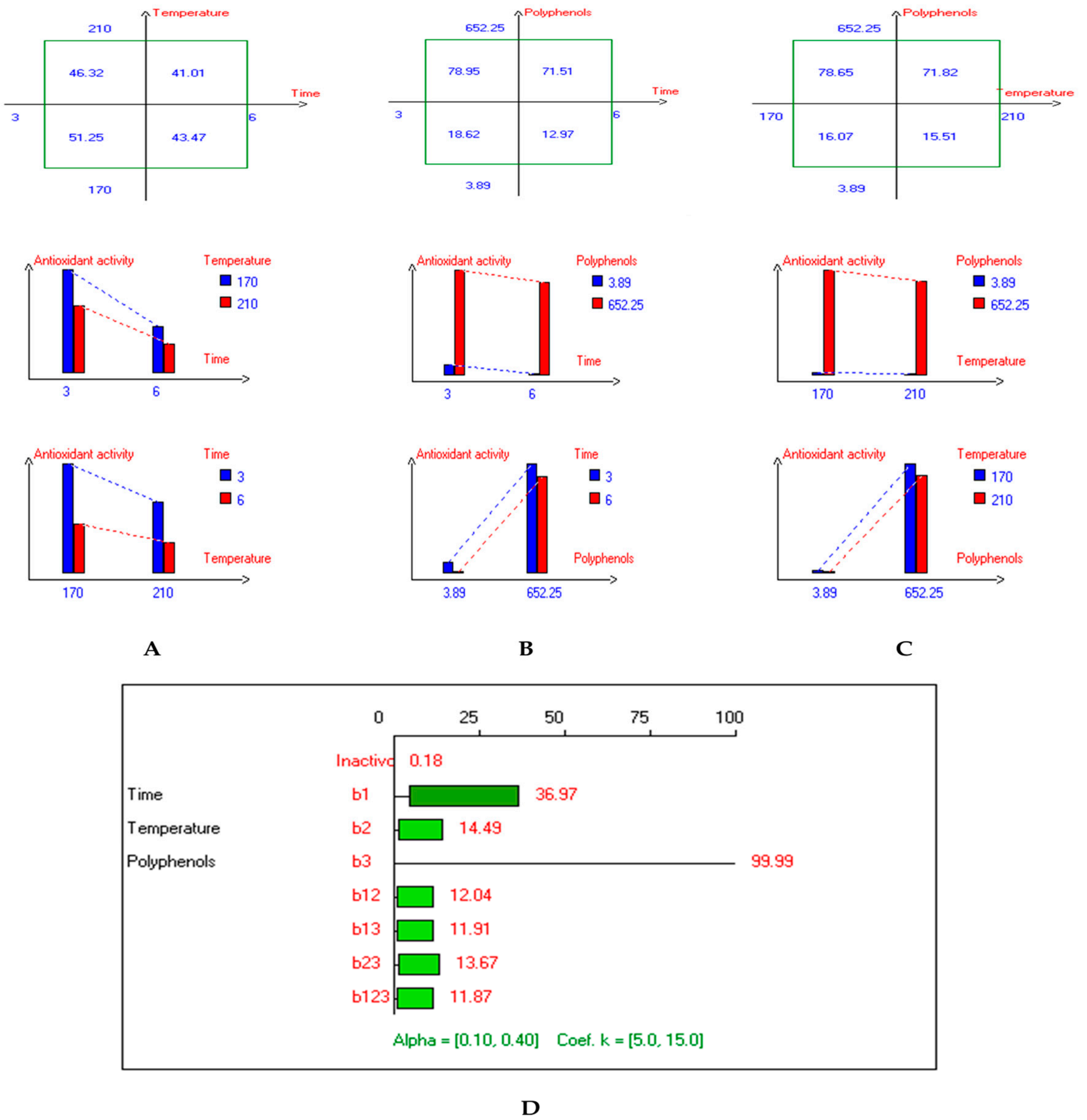

Figure S127. Combined interactions between the independent variables on a response variable (**antioxidant activity** %) in **Pomace** olive oil under D-F: (**A**) x_1 and x_2, (**B**) x_1 and x_3, (**C**) x_2 and x_3, and (**D**) results of variance analysis of regression equation model and the significance changes in each individual independent variable and interaction between the combined independent variables on antioxidant activity; b represents a significant difference when be $>b_{123}$, while b represents no significant difference when be $\leq b_{123}$; b_1, b_2, b_3 are the main effects of the independent variables, while b_{12}, b_{13}, b_{23}, and b_{123} are the interaction effects of the independent variables. Moreover, x_1, x_2, and x_3 are coded variables (time, temperature, and polyphenols addition, respectively) for the experimental design in D-F process.

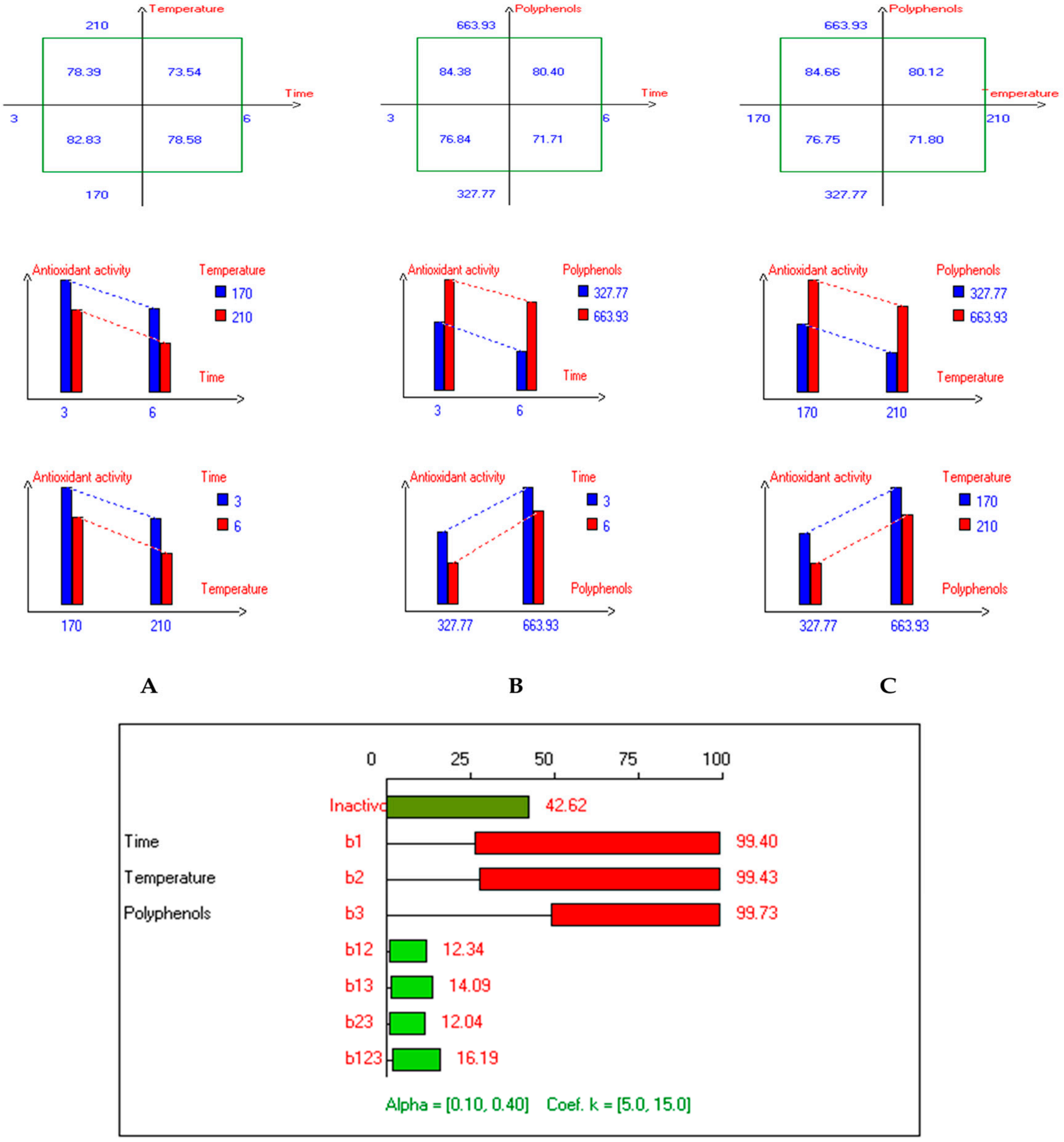

D

Figure S128. Combined interactions between the independent variables on a response variable (**antioxidant activity%**) in EVOO **Koroneiki** under D-F: (**A**) x_1 and x_2, (**B**) x_1 and x_3, (**C**) x_2 and x_3, and (**D**) results of variance analysis of regression equation model and the significance changes in each individual independent variable and interaction between the combined independent variables on antioxidant activity; b represents a significant difference when be $>b_{123}$, while b represents no significant difference when be $\leq b_{123}$; b_1, b_2, b_3 are the main effects of the independent variables, while b_{12}, b_{13}, b_{23}, and b_{123} are the interaction effects of the independent variables. Moreover, x_I, x_2, and x_3 are coded variables (time, temperature, and polyphenols addition, respectively) for the experimental design in D-F process.

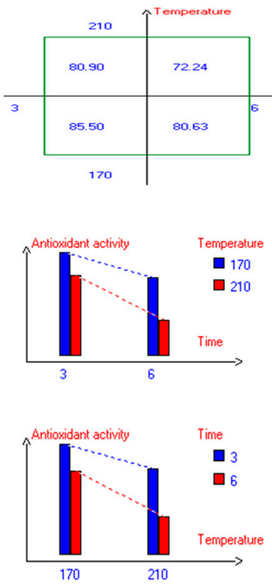

A

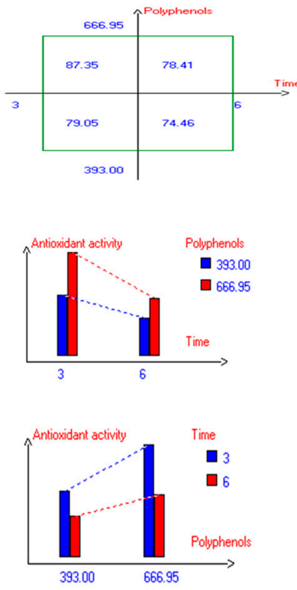

B

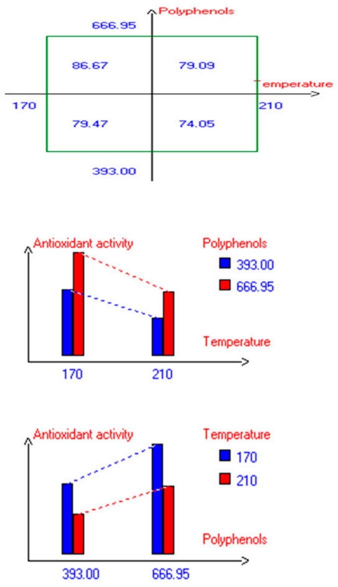

C

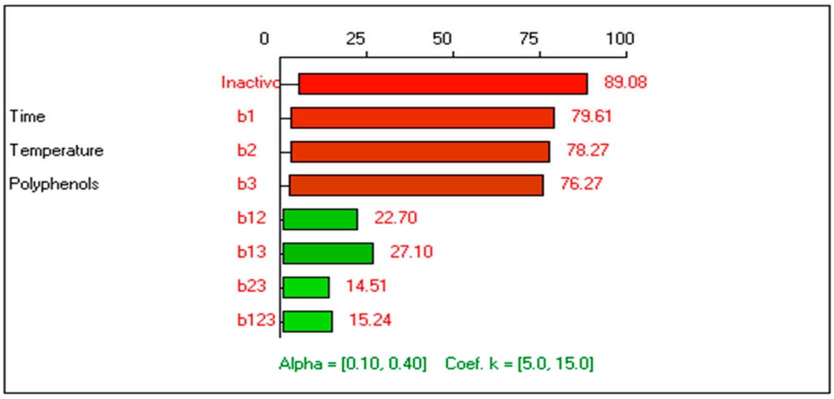

D

Figure S129. Combined interactions between the independent variables on a response variable (**antioxidant activity%**) in EVOO **Arbosana** under D-F: (**A**) x_1 and x_2, (**B**) x_1 and x_3, (**C**) x_2 and x_3, and (**D**) results of variance analysis of regression equation model and the significance changes in each individual independent variable and interaction between the combined independent variables on antioxidant activity; b represents a significant difference when be $>b_{123}$, while b represents no significant difference when be $\leq b_{123}$; b_1, b_2, b_3 are the main effects of the independent variables, while b_{12}, b_{13}, b_{23}, and b_{123} are the interaction effects of the independent variables. Moreover, x_l, x_2, and x_3 are coded variables (time, temperature, and polyphenols addition, respectively) for the experimental design in D-F process.

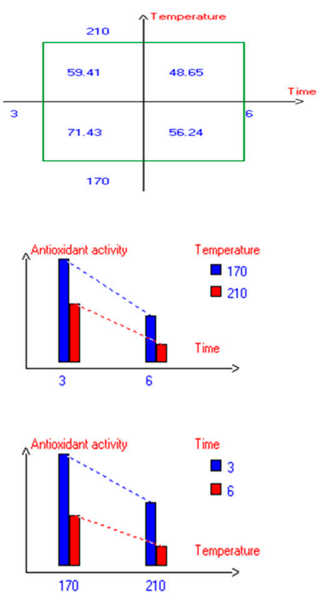

A

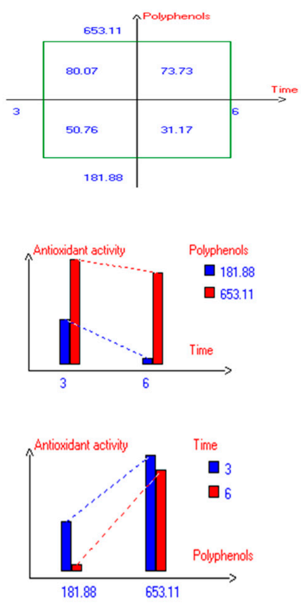

B

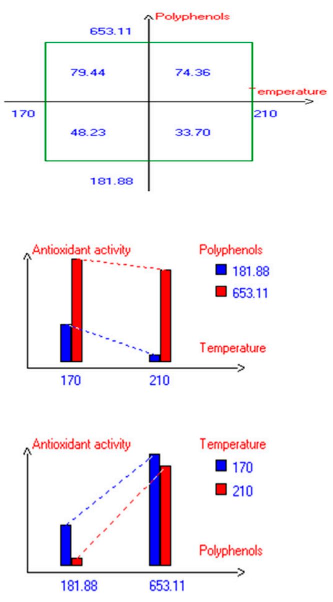

C

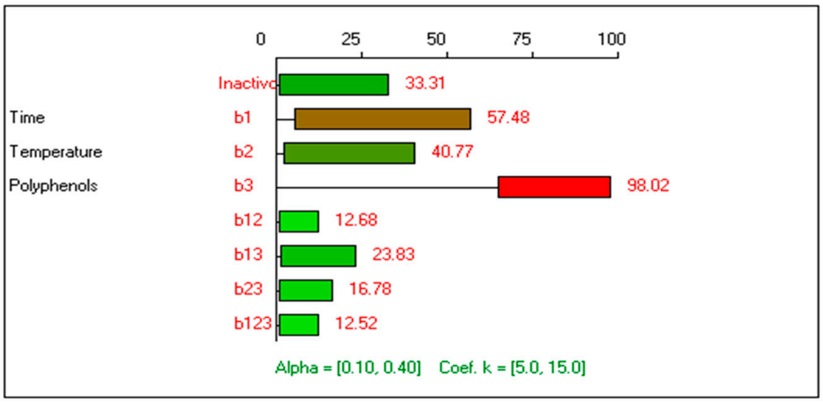

D

Figure S130. Combined interactions between the independent variables on a response variable (**antioxidant activity%**) in **Olive oil 1°** under D-F: (**A**) x_1 and x_2, (**B**) x_1 and x_3, (**C**) x_2 and x_3, and (**D**) results of variance analysis of regression equation model and the significance changes in each individual independent variable and interaction between the combined independent variables on antioxidant activity; b represents a significant difference when be $>b_{123}$, while b represents no significant difference when be $\leq b_{123}$; b_1, b_2, b_3 are the main effects of the independent variables, while b_{12}, b_{13}, b_{23}, and b_{123} are the interaction effects of the independent variables. Moreover, x_l, x_2, and x_3 are coded variables (time, temperature, and polyphenols addition, respectively) for the experimental design in D-F process.

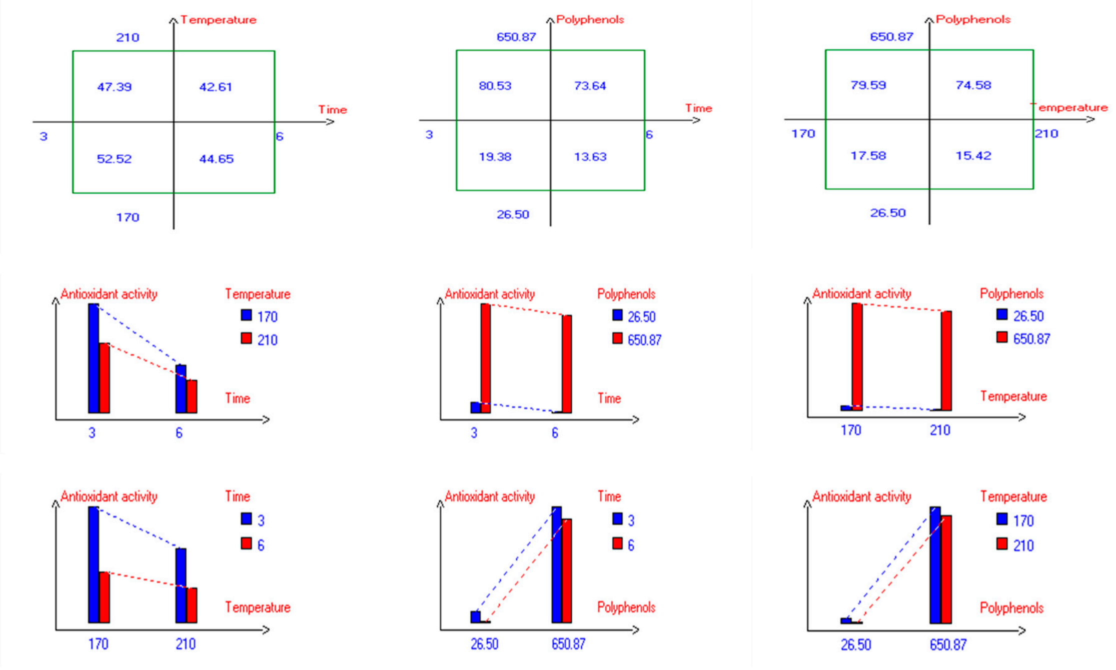

A **B** **C**

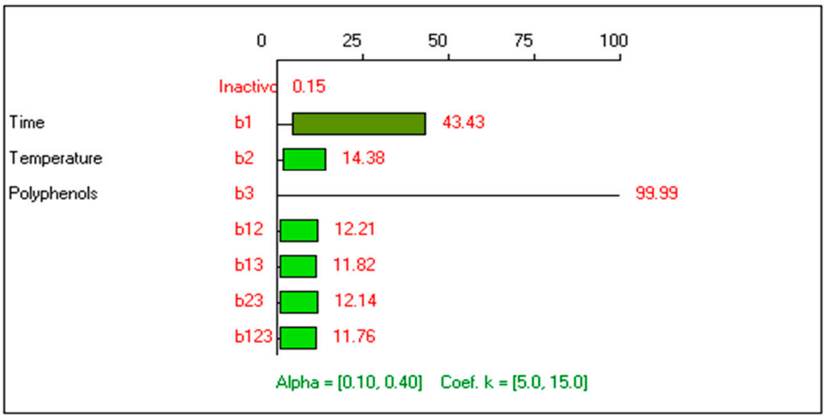

D

Figure S131. Combined interactions between the independent variables on a response variable (**antioxidant activity%**) in **Olive oil 0.4°** under D-F: (**A**) x_1 and x_2, (**B**) x_1 and x_3, (**C**) x_2 and x_3, and (**D**) results of variance analysis of regression equation model and the significance changes in each individual independent variable and interaction between the combined independent variables on antioxidant activity; b represents a significant difference when be >b_{123}, while b represents no significant difference when be ≤b_{123}; b_1, b_2, b_3 are the main effects of the independent variables, while b_{12}, b_{13}, b_{23}, and b_{123} are the interaction effects of the independent variables. Moreover, x_1, x_2, and x_3 are coded variables (time, temperature, and polyphenols addition, respectively) for the experimental design in D-F process.

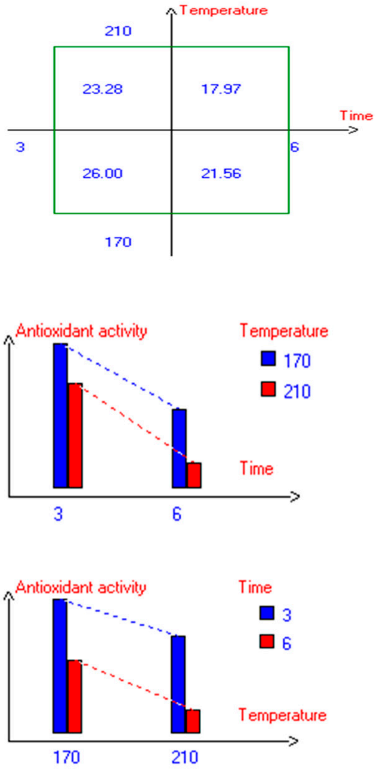

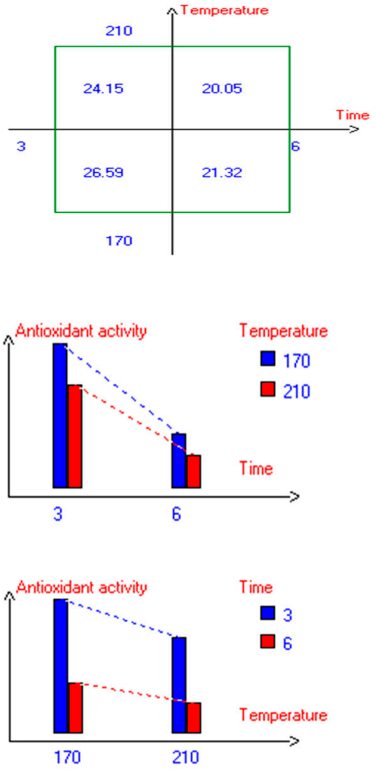

A **C**

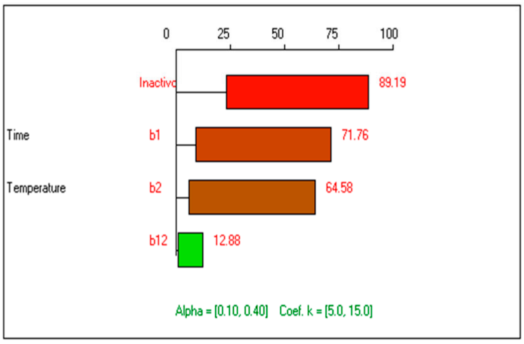

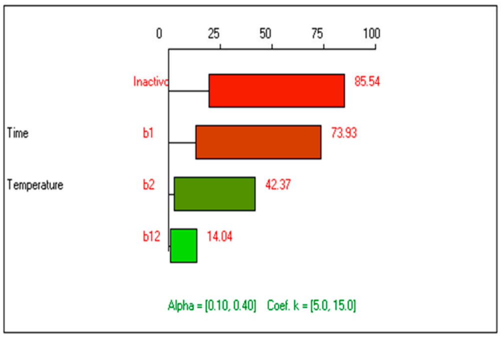

B **D**

Figure S132. (A) Combined interactions between the independent variables (x_1 and x_2) on (**antioxidant activity%**) in **sunflower oil**, **(B)** Results of variance analysis of regression equation model and the significance changes of each individual independent variable and interaction between the combined independent variables on acidity in sunflower oil. **(C)** Combined interactions between the independent variables (x_1 and x_2) on (**antioxidant activity%**) in **sunflower oil-high oleic acid**, **(D)** Results of variance analysis of regression equation model and the significance changes of each individual independent variable and interaction between the combined independent variables on rancid score in sunflower oil-high oleic acid. Where, b represents significant difference when be $>b_{12}$; while b represents no significant difference when be $\leq b_{12}$. Moreover, b_1 and b_2 are the main effects of the independent variables, while b_{12} is the interaction effect of the independent variables. Where x_1: time, x_2: temperature.
